# Network pharmacology and molecular docking technology-based predictive study of the active ingredients and potential targets of rhubarb for the treatment of diabetic nephropathy

**DOI:** 10.1186/s12906-022-03662-6

**Published:** 2022-08-06

**Authors:** Shaojie Fu, Yena Zhou, Cong Hu, Zhonggao Xu, Jie Hou

**Affiliations:** 1 Department of Nephrology, the First Hospital of Jilin University, Changchun, 130021 Jilin China; 2 Center for Reproductive Medicine, Center for Prenatal Diagnosis, The First Hospital of Jilin University, Changchun, 130021 Jilin China

**Keywords:** Diabetic nephropathy, Rhubarb, Network pharmacology, Chinese traditional medicine, Molecular docking

## Abstract

Diabetic nephropathy (DN) is one of the most serious complications of diabetes and the main cause of end-stage renal failure. Rhubarb is a widely used traditional Chinese herb, and it has exhibited efficacy in reducing proteinuria, lowering blood sugar levels and improving kidney function in patients with DN. However, the exact pharmacological mechanism by rhubarb improves DN remain unclear due to the complexity of its ingredients. Hence, we systematically explored the underlying mechanisms of rhubarb in the treatment of DN. We adopted a network pharmacology approach, focusing on the identification of active ingredients, drug target prediction, gene collection, Gene Ontology enrichment and Kyoto Encyclopedia of Genes and Genomes enrichment. Molecular docking technology was used to verify the binding ability between the main active compounds and central therapeutic targets, and screen out the core active ingredients in rhubarb for the treatment of DN. Finally, molecular dynamics simulation was performed for the optimal core protein-ligand obtained by molecular docking using GROMACS software. The network analysis identified 16 active compounds in rhubarb that were linked to 37 possible therapeutic targets related to DN. Through protein–protein interaction analysis, TP53, CASP8, CASP3, MYC, JUN and PTGS2 were identified as the key therapeutic targets. By validation of molecular docking, finding that the central therapeutic targets have good affinities with the main active compounds of rhubarb, and rhein, beta-sitosterol and aloe-emodin were identified as the core active ingredients in rhubarb for the treatment of DN. Results from molecular dynamics simulations showed that TP53 and aloe-emodin bound very stably with a binding free energy of − 26.98 kcal/mol between the two. The results of the gene enrichment analysis revealed that the PI3K-Akt signalling pathway, p53 signalling pathway, AGE-RAGE signalling pathway and MAPK signalling pathway might be the key pathways for the treatment of DN, and these pathways were involved in podocyte apoptosis, glomerular mesangial cell proliferation, inflammation and renal fibrosis. Based on the network pharmacology approach and molecular docking technology, we successfully predicted the active compounds and their respective targets. In addition, we illustrated the molecular mechanisms that mediate the therapeutic effects of rhubarb against DN. These findings provided an important scientific basis for further research of the mechanism of rhubarb in the treatment of DN.

**Supplementary Information:**

The online version contains supplementary material available at 10.1186/s12906-022-03662-6.

## Introduction

Because of the improvement of living conditions, increasingly sedentary lifestyles, increased rates of obesity and an ageing population, the global prevalence of diabetes is increasing at an alarming rate [1]. Diabetic nephropathy (DN) is one of the most serious complications of diabetes and the main cause of end-stage renal failure [2]. The incidence of DN in patients with diabetes is approximately 20–40% [3]. Once DN enters the clinical phase, kidney damage progresses rapidly, which seriously affects patient quality of life [4]. However, current treatments for DN are limited, and they mainly aim to control blood sugar, reduce blood pressure and regulate lipids, thereby improving hypercoagulability to delay the occurrence and development of proteinuria and protect kidney function [5, 6].

In recent years, some hospitals have assessed the efficacy of traditional rhubarb (Radix Rhei Et Rhizome), a widely used traditional Chinese herb, as a long-term treatment for DN [7, 8]. Clinically, rhubarb and its ingredients have been widely used in the treatment of DN with significant efficacy [9, 10]. Oral rhubarb reduced proteinuria, lowered blood sugar levels and improved kidney function in patients with DN [11]. Rhein purified from rhubarb can decrease lipid levels and improve renal lesions in db/db mice with DN, an ideal animal model for type 2 diabetes research [12]. However, the mechanism by which rhubarb alleviates DN has not yet been elucidated. Nevertheless, the integration of bioinformatics and network pharmacology provides a practical approach to explore the mechanisms of action [13]. Network pharmacology can systematically reveal the active components in drugs and predict the relationships between drug components and gene targets [14, 15]. Molecular docking could verify the interaction between the active compounds and central therapeutic targets [16]. Molecular dynamics relies on Newtonian mechanics to assess the binding stability and flexibility of ligand and receptor by simulating their movement [17]. This study is the first to apply network pharmacology, molecular docking and molecular dynamics simulation technology in combination to fully reveal the underlying mechanisms by which rhubarb produces therapeutic effects for DN patients, and to predict the main active compounds and key targets of rhubarb for the treatment of DN, as well as the signalling pathways involved.

## Materials and methods

### Screening of the active compounds of rhubarb

The Traditional Chinese Medicine Systems Pharmacology (TCMSP) database is an authoritative Chinese herbal medicine database that captures the relationships among drugs, targets and diseases [18], whereas Traditional Chinese Medicines Integrated Database (TCMID) is a large repository of information pertaining to herbs and herbal ingredients [19]. ‘Radix Rhei Et Rhizome’ was used as the keyword to retrieve all chemical compounds of rhubarb from the TCMSP (http://tcmspw.com/tcmsp.php, Ver.2.3) and TCMID databases (http://www.megabionet.org/tcmid/, Ver.1.0). Oral bioavailability (OB) refers to the percentage of an oral drug that reaches the systemic circulation. It reflects the degree of absorption and utilisation of drugs in the body [20]. Drug similarity (DL) reflects the structural similarity between the compound and drug molecule. Thus, compounds with a high DL value are more likely to exhibit appropriate pharmacodynamic and pharmacokinetic properties [21]. Therefore, OB and DL provide important references for screening active compounds and as the TCMSP database suggested OB ≥ 30% and DL ≥ 0.18 were used as the screening criteria to select the active compounds of rhubarb [22].

### Prediction of the targets of rhubarb

DrugBank (https://www.drugbank.ca/) is a comprehensive bioinformatics and cheminformatics database that provides detailed information on drug types, chemical structures, drug targets and other information [23]. The related targets of the active compounds of rhubarb were gathered from DrugBank under the condition of *Homo sapiens*. UniProt (http://www.uniprot.org/) is the most informative and extensive protein database, and it is used to standardise gene names [24]. We obtained the target gene names corresponding to the target protein names of active chemical compounds using UniProt.

### Collection of gene targets in DN

The human genes associated with DN were gathered from four databases, namely Online Mendelian Inheritance in Man (OMIM), GeneCards, Pharmacogenomics Knowledge Base (PharmGkb) and DrugBank. OMIM (https://omim.org/) is an authoritative and comprehensive database of human genes and genetic phenotypes [25]. GeneCards (https://www.genecards.org/) is an integrative database that provides information on all predicted and annotated human genes [26]. PharmGKB (https://www.pharmgkb.org/) is an integrated resource about how variation in human genetics leads to variation in responses to drugs [27]. DrugBank (https://www.drugbank.ca/), as a comprehensive bioinformatics database, also provides relevant information on disease targets [28]. The search term ‘diabetic nephropathy’ was used to retrieve the DN targets from the four databases. R package-VennDiagram was used to plot the venn diagram to visualize the relationship between the search results for each database [29].

### Therapeutic targets of rhubarb for treating DN

We screened the active compounds of rhubarb and identified their target genes. We also gathered the DN-related genes. Taking the intersection of the aforementioned two groups of genes, the overlapped genes were considered potential therapeutic targets of rhubarb against DN.

### Protein–protein interaction (PPI)

The PPIs of the therapeutic targets of rhubarb in the treatment of DN were gathered using STRING (https://string-db.org/, version 11.0), a database of known and predicted PPI that uses bioinformatic strategies to collect information [30]. In this study, we limited the species to ‘*Homo sapiens*’, collected the PPIs with confidence scores > 0.4 and hid the disconnected nodes in the network.

### Network construction

The active compounds–therapeutic targets network was built by linking the active compounds with the therapeutic targets of rhubarb in the treatment of DN. The PPI network of therapeutic targets was established by connecting the therapeutic targets to their interacting targets. The two networks were visualised using Cytoscape (http://www.cytoscape.org/, version 3.7.2), which can graphically display and analyse networks [31].

In this study, to screen the central targets, we used the NetworkAnalyzer tool of Cytoscape to calculate three topological parameters for each node in the PPI network of therapeutic targets, including degree, closeness centrality and betweenness centrality [32, 33]. For each variable, higher values indicate greater importance of the node in the network [34]. Nodes with values of all three topological parameters exceeding the median were selected to build sub-network, in which another selection was performed to finally obtain the central targets.

### Molecular docking

Molecular docking is the most widely used technique in drug design because it can predict the ability of ligands and proteins to bind, as well as the location of binding [35]. In this study, to obtain the core active ingredients in rhubarb for the treatment of DN, molecular docking was performed on the key targets and the active compounds acting on them. The two-dimensional structures of the active compounds of rhubarb were gathered from PubChem. Then their three-dimensional structures were converted using ChemBio3D Ultra 17.0 and optimised by MMFF94 force field to obtain the three-dimensional conformation of the minimum free energy of each compound [36]. The crystal structures of the key targets TP53, PTGS2, MYC, CASP3, CASP8, and JUN were all downloaded from PDB database (Protein Data Bank: www.rcsb.org), and their corresponding PDB IDs were as follows: 3DCY, 5IKR, 5I4Z, 6X8I, 5H31 and 5FV8 [37–43]. These crystal structures were imported into PyMOL 1.7.2.1 software (https://pymol.org/2/) for various optimisation operations such as dehydration, hydrogenation and isolation of original ligands, and the optimised targets were entered into AutoDockTools − 1.5.6 to construct a docking grid box. The active site of molecular docking was determined using the ligand coordinate in the target protein complex, and Autodock vina 1.1.2 was used for molecular docking research [44]. Twenty conformations were generated for each molecular docking. The best affinity conformation was selected as the final docking conformation. The compound-target interactions in the docking conformation were analyzed by ligplot 2.2 and visualised in Pymol 2.3.

### Molecular dynamics simulation

To further investigate the dynamic interaction process and the stability of binding between proteins and small ligands, molecular dynamics simulation was also performed. Molecular dynamics simulation is a popular technique to study protein motion by tracking their conformational changes over time [45]. The GROMACS software package was used to perform molecular dynamics simulation in this study [46]. The protein used the AMBER14SB force field parameter, while the ligand atomic charges were calculated by B3LYP/6-31G* basis set and the ligand topology was built by GAFF2 force field parameter. TIP3P water model was used to add the antagonist sodium ions and hydrogen atoms to neutralize the charge. Electrostatic interactions were treated separately using the Particle Mesh Ewald (PME) and Verlet algorithms. The heavy atoms of proteins were confined, and the steepest descent method was applied to minimize the energy at 50000 steps. The simulation system was equilibrated for 100 ps using canonical ensembles (NVT) and isothermal isobaric ensembles (NPT). Both Van-der-Waals and Coulomb interactions were calculated using a cut-off of 1.4 nm. Finally, the system performed a 100 ns molecular dynamics simulation at constant temperature (300 K) and constant pressure (1 bar) with a time step of 2 fs and the trajectory data was saved every 5 ps.

### Gene ontology (GO) and Kyoto encyclopedia of genes and genomes (KEGG) pathway enrichment

We used the GO database (http://geneontology.org/) to clarify the possible biological mechanisms of therapeutic targets, including biological process (BP), cell component (CC) and molecular function (MF) terms deemed significant at *P* < 0.05 [47]. We used the KEGG database (https://www.kegg.jp/) to further explore the vital biological relevance of therapeutic targets and verify the reliability of the integrated results [48]. The enrichment analysis of GO and KEGG was performed using R package-Bioconductor clusterProfiler, which is widely used for gene clusters enrichment analysis [49].

## Results

**Fig. 1:**
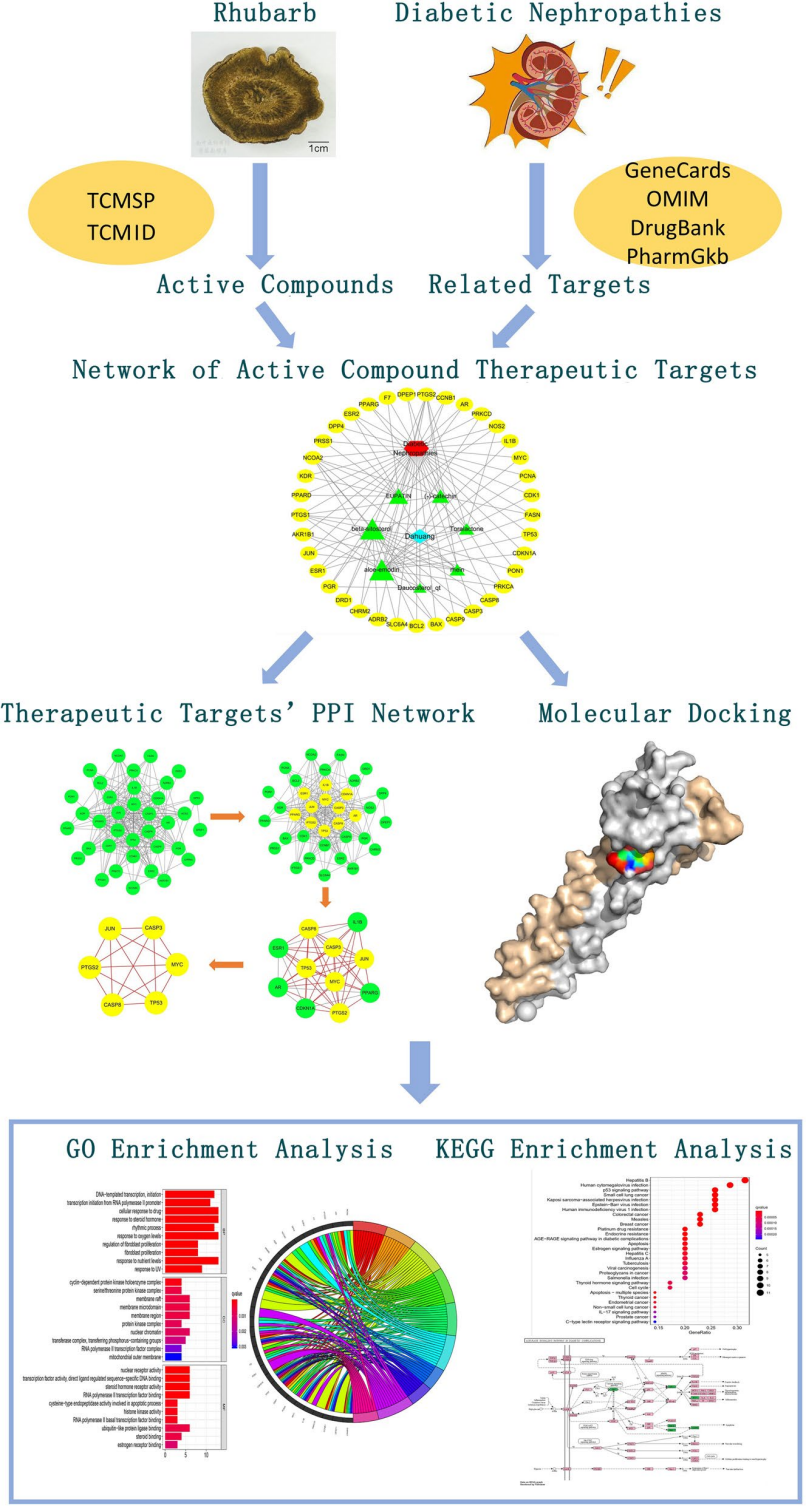
Schematic illustration of this network pharmacology study of rhubarb for the treatment of diabetic nephropathy. GO, Gene Ontology; KEGG, Kyoto Encyclopedia of Genes and Genomes; PPI, protein–protein interaction

The flowchart of the study based on network pharmacology is presented in Fig. 1.

### Active compounds of rhubarb

In the search, 92 compounds were obtained from the TCMSP and TCMID databases, including flavonoids, steroids, alkaloids, glycosides and triterpenes. According to the OB and DL of the ingredients, 16 compounds were selected as potential active ingredients, and their characteristics are listed in Table 1.

**Table 1:**
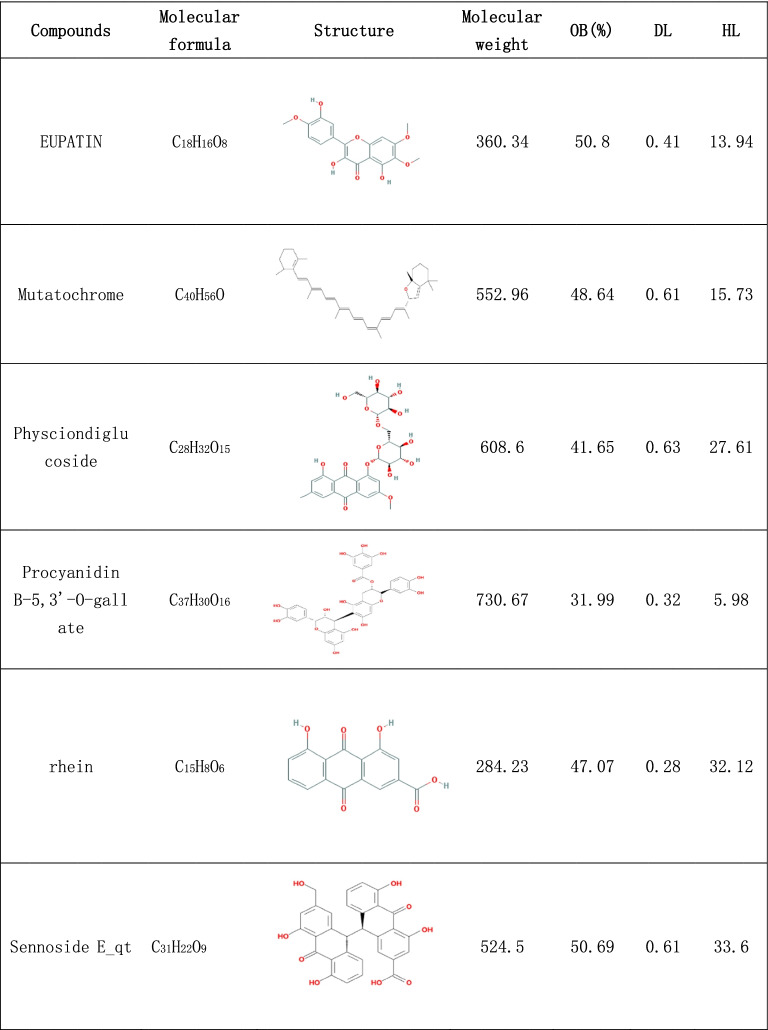

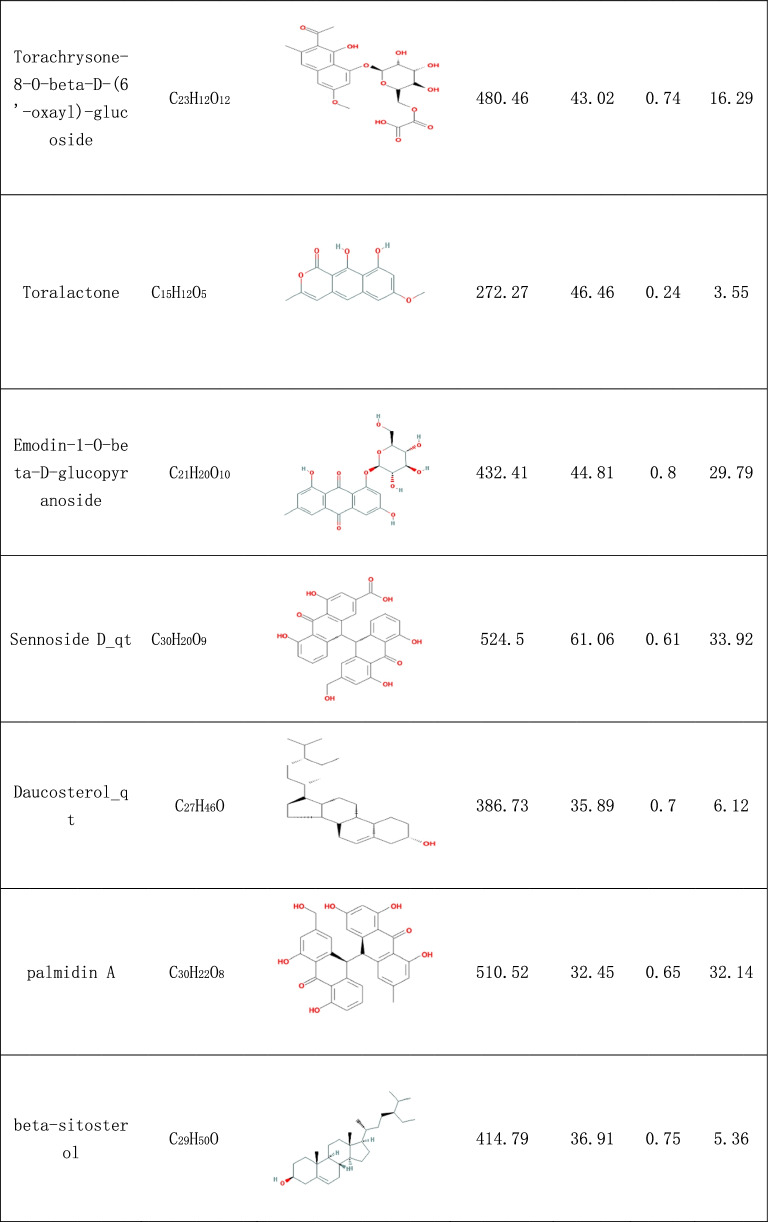

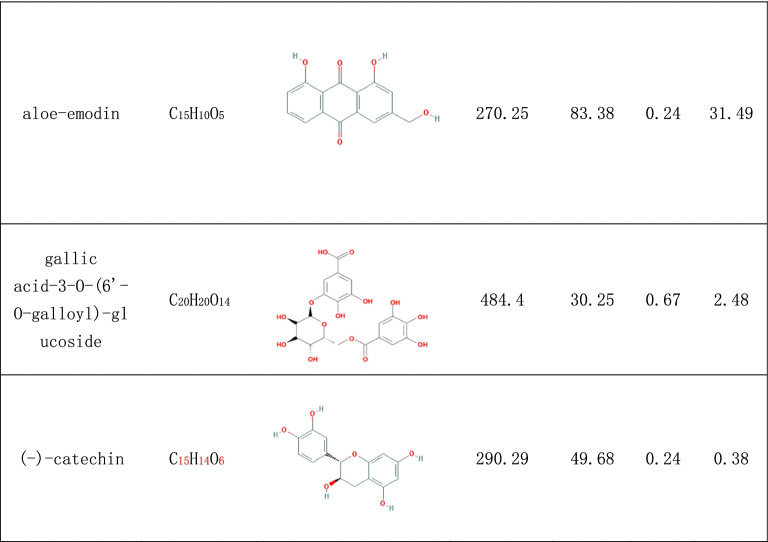
The characteristics of potential active ingredients

### Prediction of the targets of rhubarb

The targets of active compounds were predicted using the DrugBank database, and the target protein names were converted into target gene names using the UniProt database. Finally, 88 targets were predicted for these active compounds of rhubarb, and they were regarded as potential targets (Supplementary Table 1).

### Collection of gene targets in DN

Regarding genes related to DN, 3319, 68, 79 and 43 genes were retrieved from GeneCards, OMIM, PharmGkb and DrugBank, respectively, (Supplementary Table 2). The genes commonly predicted by the four databases were considered the genes associated with DN, and the details are depicted in Supplementary Figure 1.

### Therapeutic targets of rhubarb for DN

The target genes of rhubarb were predicted, and the genes associated with DN were obtained. Using the aforementioned commonly predicted genes, 37 possible therapeutic targets were gathered, and their features are listed in Table 2.

**Table 2:** The characteristics of therapeutic targets

Gene Symbol	Protein names	UniPort ID	Degree	Betweenness	Closeness
TP53	Cellular tumor antigen p53	P04637	26	119.80086	0.744681
CASP3	Caspase-3	P42574	24	89.589681	0.729167
MYC	Myc proto-oncogene protein	P01106	24	41.276849	0.714286
ESR1	Estrogen receptor	P03372	24	102.85391	0.729167
JUN	Transcription factor AP-1	P05412	24	53.369635	0.714286
PTGS2	Prostaglandin G/H synthase 2	P35354	23	72.477952	0.729167
PPARG	Peroxisome proliferator-activated receptor gamma	P37231	21	158.33969	0.714286
CASP8	Caspase-8	Q14790	19	12.393883	0.648148
AR	Androgen receptor	P10275	18	12.240176	0.636364
CDKN1A	Cyclin-dependent kinase inhibitor 1	P38936	18	13.525691	0.636364
CASP9	Caspase-9	P55211	17	8.2618119	0.625
IL1B	Interleukin-1 beta	P01584	17	52.104864	0.648148
CCNB1	G2/mitotic-specific cyclin-B1	P14635	15	4.9289352	0.59322
CDK1	Cyclin-dependent kinase 1	P06493	14	4.7454907	0.57377
PGR	Progesterone receptor	P06401	14	3.452711	0.583333
KDR	Vascular endothelial growth factor receptor 2	P35968	14	1.6679282	0.59322
BAX	Apoptosis regulator BAX	Q07812	13	1.8816711	0.57377
NOS2	Nitric oxide synthase, inducible	P35228	12	11.292795	0.57377
PRKCA	Protein kinase C alpha type	P17252	12	15.963106	0.59322
BCL2	Apoptosis regulator Bcl-2	P10415	10	0.9061896	0.538462
PRKCD	Protein kinase C delta type	Q05655	10	8.6580533	0.564516
ESR2	Estrogen receptor beta	Q92731	10	1.1598973	0.555556
NCOA2	Nuclear receptor coactivator 2	Q15596	9	6.10359	0.546875
ADRB2	Beta-2 adrenergic receptor	P07550	9	98.01746	0.538462
AKR1B1	Aldo-keto reductase family 1 member B1	P15121	9	22.285304	0.57377
FASN	Fatty acid synthase	P49327	7	5.0822475	0.530303
PCNA	Proliferating cell nuclear antigen	P12004	6	0	0.486111
PTGS1	Prostaglandin G/H synthase 1	P23219	6	0.2352941	0.514706
SLC6A4	Sodium-dependent serotonin transporter	P31645	5	43.590673	0.492958
PPARD	Peroxisome proliferator-activated receptor delta	Q03181	5	1.7936508	0.466667
DPP4	Dipeptidyl peptidase 4	P27487	4	68	0.486111
CHRM2	Muscarinic acetylcholine receptor M2	P08172	2	0	0.368421
DRD1	D(1A) dopamine receptor	P21728	2	0	0.368421
PON1	Serum paraoxonase/arylesterase 1	P27169	1	0	0.421687
DPEP1	Dipeptidase 1	P16444	1	0	0.330189
PRSS1	Trypsin-1	P07477	1	0	0.432099
F7	Coagulation factor VII	P08709	0	0	0

### Active compounds–therapeutic targets network

**Fig. 2:**
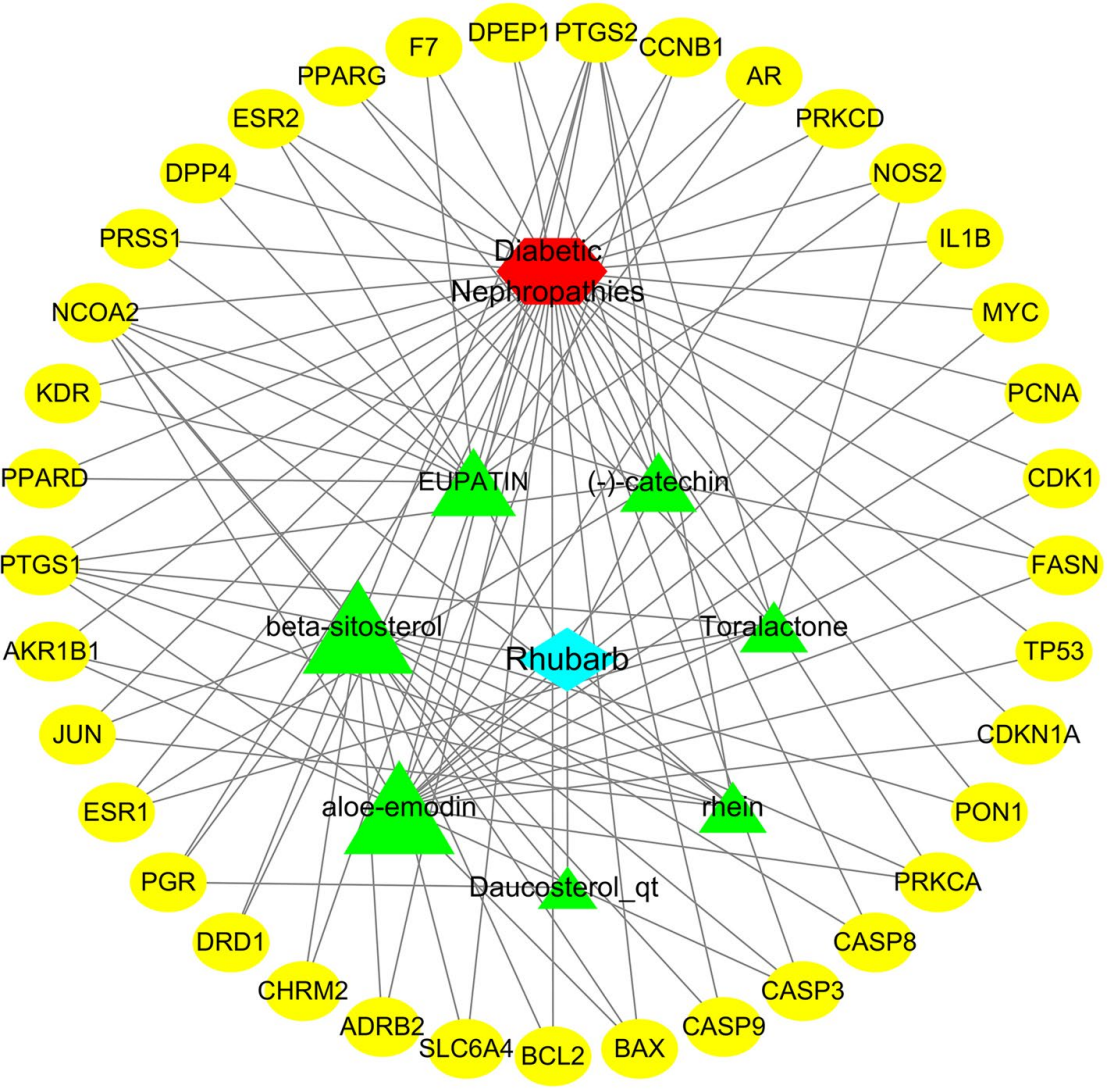
Active compounds-therapeutic targets network. The blue rhombus represents rhubarb, the red hexagon represents DN, yellow ovals represent the therapeutic targets and green triangles represent active compounds. The sizes of compound nodes are proportional to their degree

The active compounds–therapeutic targets network is depicted in Fig. 2, and it revealed the relationships between the active compounds and therapeutic targets, including 46 total nodes (7 compound nodes, 37 therapeutic target nodes, 1 rhubarb node and 1 DN node) and 105 edges. This network fully reveals the characteristics of rhubarb for the treatment of DN through multi-components and multi-targets. The larger the Degree value of the compound node in the network, the more therapeutic targets this compound acts on. As presented in Fig. 2, the two nodes with the largest degree values were aloe-emodin and beta-sitosterol (degree = 17), and their degree values were much larger than those of the other nodes (mean, 6.8), indicating that aloe-emodin and beta-sitosterol may play significant roles in the effects of rhubarb against DN.

### Therapeutic target PPI network

**Fig. 3:**
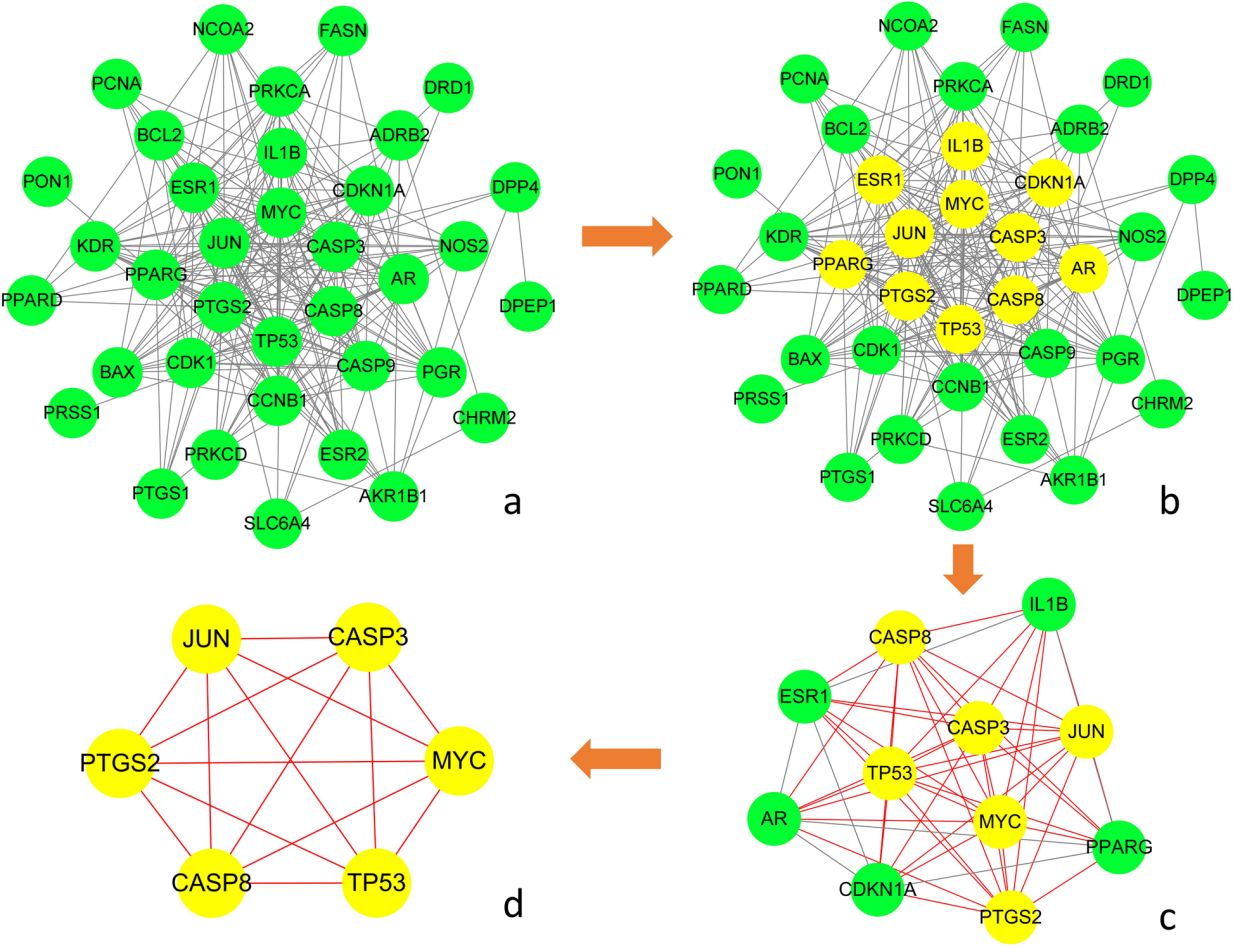
Protein–protein interaction network of therapeutic targets. **a** Is the original PPI network of therapeutic targets and all nodes in the network are green. In **b**, the core nodes in this network screened by their topological features are yellow, and the other nodes are green. **c** Is the sub-network constructed from the core nodes of (**b**), the core nodes in this network screened by their topological features are yellow, and the other nodes are green. **d** Is the sub-network constructed from the core nodes of (**c**), all nodes in the network are yellow and represent the final central therapeutic targets obtained through screening

To identify the central therapeutic targets of the effects of rhubarb against DN, the PPI network of therapeutic targets is depicted in Fig. 3a, including 37 nodes and 223 edges. NetworkAnalyzer was used to calculate three topological features of the 36 targets, and the details are listed in Table 2. The median degree, betweenness and closeness values of these nodes were 12, 8.45993 and 0.57377, respectively. As shown in Fig. 3b and c, nodes with values of all three topological parameters exceeding the median were selected to build sub-network. In sub-network another selection was performed to finally obtain the central targets (Fig. 3c and d). Finally, 6 genes were identified as central therapeutic targets of the effects of rhubarb against DN, including TP53, CASP8, CASP3, MYC, JUN and PTGS2.

### Results of molecular docking

**Fig. 4:**
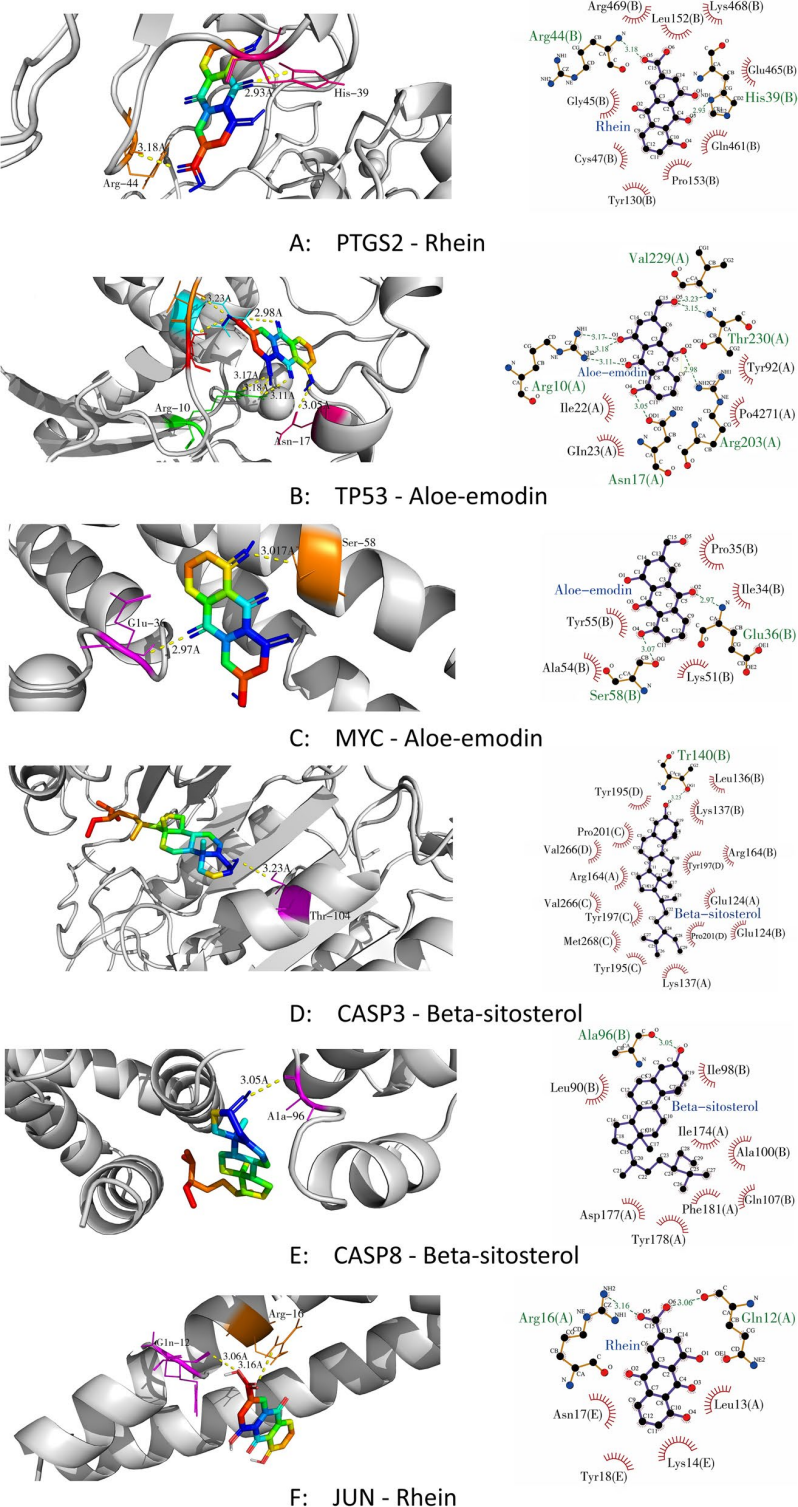
Molecular docking of the key therapeutic targets and active compounds of rhubarb. In 3D interactions, the colorful ring structures represent the active ingredients; the colorful segments on the protein structure represent the amino acids forming hydrogen bonds with the active ingredient; the yellow dashed lines represent the hydrogen bonds formed between the ingredient and the protein. In 2D interactions, the purple ring structures represent the active ingredients; the orange chain structures represent the amino acids forming hydrogen bonds with the active ingredient; the red arc structures represent the amino acids forming hydrophobic interaction with the active ingredient; the green dashed lines represent the hydrogen bonds formed between the ingredient and the protein

In this study, molecular docking technology was used to obtain the core active ingredients in rhubarb for the treatment of DN. Based on the result of therapeutic target PPI network, the central therapeutic targets were identified, and they were selected for molecular docking with the active compounds of rhubarb acting on them, respectively. According to the receptor-ligand docking theory, docking energy is inversely correlated with binding affinity, and a more negative docking energy indicates stronger binding affinity between the protein and the ligand. The docking details are shown in Table 3. The six pairs of compounds and proteins with the best binding affinity were PTGS2 and rhein, TP53 and aloe-emodin, MYC and aloe-emodin, CASP3 and beta-sitosterol, CASP8 and beta-sitosterol, and JUN and rhein, respectively. The docking data of above six pairs of compounds and proteins were imported into Ligplot software to analyze the compound and protein interactions and visualised in Pymol software, which is depicted in Fig. 4. The binding energy values of the 6 central therapeutic targets with the active compounds acting on them were all less than − 5 kcal/mol, and at least one hydrogen bond formed between them, indicating these compounds have good binding affinity to the key therapeutic targets and can fully play the role of anti-MN. The screening criteria for core active ingredients were as follows: ① Compounds with the highest affinity for each key therapeutic target; ② Compounds that act on the key therapeutic targets must have a docking energy of less than − 5 kcal/mol. The core active ingredients in rhubarb for the treatment of DN were obtained by the above screening criteria were rhein, beta-sitosterol and aloe-emodin.

**Table 3:** The molecular docking details

Target	PDB IDs	Active compounds	Docking energy(KJ/mol)
PTGS2	5IKR	EUPATIN	−9.4
PTGS2	5IKR	Rhein	−9.7
PTGS2	5IKR	Toralactone	−9.3
PTGS2	5IKR	beta-sitosterol	−9.2
PTGS2	5IKR	aloe-emodin	−9.1
PTGS2	5IKR	(−)-catechin	−9.3
PTGS2	5IKR	Daucosterol_qt	−8.8
TP53	3DCY	EUPATIN	−7.9
TP53	3DCY	Rhein	−8.0
TP53	3DCY	Toralactone	−7.5
TP53	3DCY	beta-sitosterol	−7.9
TP53	3DCY	aloe-emodin	−8.3
TP53	3DCY	(−)-catechin	−7.8
TP53	3DCY	Daucosterol_qt	−8.2
MYC	5I4Z	EUPATIN	−6.1
MYC	5I4Z	Rhein	−6.6
MYC	5I4Z	Toralactone	−6.1
MYC	5I4Z	beta-sitosterol	−6.2
MYC	5I4Z	aloe-emodin	−6.7
MYC	5I4Z	(−)-catechin	−6.3
MYC	5I4Z	Daucosterol_qt	−6.6
CASP3	6X8I	EUPATIN	−7.5
CASP3	6X8I	Rhein	−8.1
CASP3	6X8I	Toralactone	−7.8
CASP3	6X8I	beta-sitosterol	−8.3
CASP3	6X8I	aloe-emodin	−7.5
CASP3	6X8I	(−)-catechin	−7.7
CASP3	6X8I	Daucosterol_qt	−8.2
CASP8	5H31	EUPATIN	−7.7
CASP8	5H31	Rhein	−7.9
CASP8	5H31	Toralactone	−7.2
CASP8	5H31	beta-sitosterol	−8.0
CASP8	5H31	aloe-emodin	−7.7
CASP8	5H31	(−)-catechin	−7.8
CASP8	5H31	Daucosterol_qt	−7.9
JUN	5FV8	EUPATIN	−6.3
JUN	5FV8	Rhein	−6.7
JUN	5FV8	Toralactone	−6.6
JUN	5FV8	beta-sitosterol	−6.4
JUN	5FV8	aloe-emodin	−6.4
JUN	5FV8	(−)-catechin	−6.4
JUN	5FV8	Daucosterol_qt	−6.6

### Results of molecular dynamics simulation

**Fig. 5:**
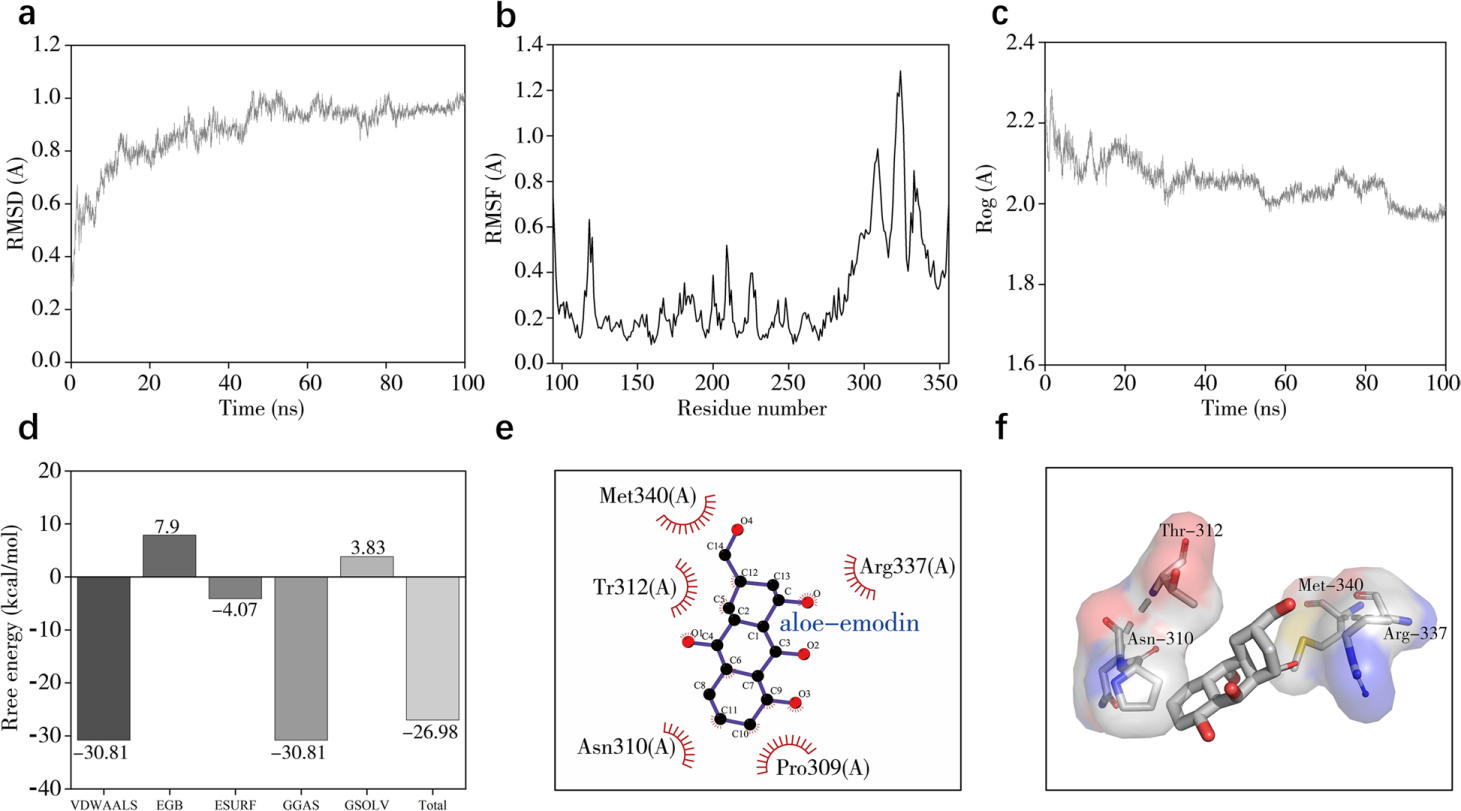
Molecular dynamics simulation of TP53 and aloe-emodin. **a** Shows the RMSD plot during molecular dynamics simulation of TP53 and aloe-emodin. **b** Shows the RMSF plot during molecular dynamics simulation of TP53 and aloe-emodin. **c** Shows the Rog plot during molecular dynamics simulation of TP53 and aloe-emodin. **d** Shows the binding free energy between TP53 and aloe-emodin. **e** and **f** show the 2D and 3D diagram of TP53 and aloe-emodin interaction

Since proteins and small ligands are in absolute motion, and the conformation involved in the molecular docking is only a relatively stationary state for them. Therefore, to get more confidence in the binding stability, we performed molecular dynamics simulation of TP53 and aloe-emodin, where TP53 was the most central therapeutic target with the highest Degree value in the PPI network, and aloe-emodin was the active ingredient of rhubarb that molecular docking results showed to bind most tightly to TP53. The RMSD curve reflects the fluctuation in protein conformation. As shown in Fig. 5a, the RMSD has some fluctuations in the early stage. However, the RMSD fluctuations of TP53 and aloe-emodin system stabilized after 55 nanoseconds (ns), indicating that the conformation of TP53 did not change significantly after the binding of aloe-emodin to it and the binding of the two was relatively stable. The RMSF curve reflects the fluctuation of the protein amino acid residues. As shown in Fig. 5b, the result of TP53 and aloe-emodin shows that the amino acids 114–134 and 287–354 of TP53 are highly volatile and has greater residue flexibility than other regions. The gyration radius curve reflects the compactness of the overall structure of protein. As shown in Fig. 5c, after a brief fluctuation at the beginning, the gyration radius curve gradually tends to balance, indicating that TP53 conformation is stable and is compactly folded. In addition, we also obtained the trajectory of the RMSD stationary phase (50–100 ns) of TP53 and aloe-emodin and calculated the binding free energy using gmx_MMPBSA. As shown in Fig. 5d, the binding free energy between the two is − 26.98 kcal/mol. Van der Waals energy (VDWAALS, − 30.81 kcal/mol), non-polar solvation energy (ESURF, − 4.07 kcal/mol) and total gas phase free energy (GGAS, − 30.81 kcal/mol) are conducive to the combination of both, while the polar solvation energy (EGB, 7.9 kcal/mol) and total solvation free energy (GSOLV, 3.83 kcal/mol) is not conducive to the interaction of both. As shown in Fig. 5e and f, the binding site of TP53 and aloe-emodin forms a relatively hydrophobic environment with strong hydrophobicity. The residues that interact with aloe-emodin are mainly hydrophobic residues, such as Thr312, Pro309, and Asn310.

### GO and KEGG pathway enrichment

**Fig. 6:**
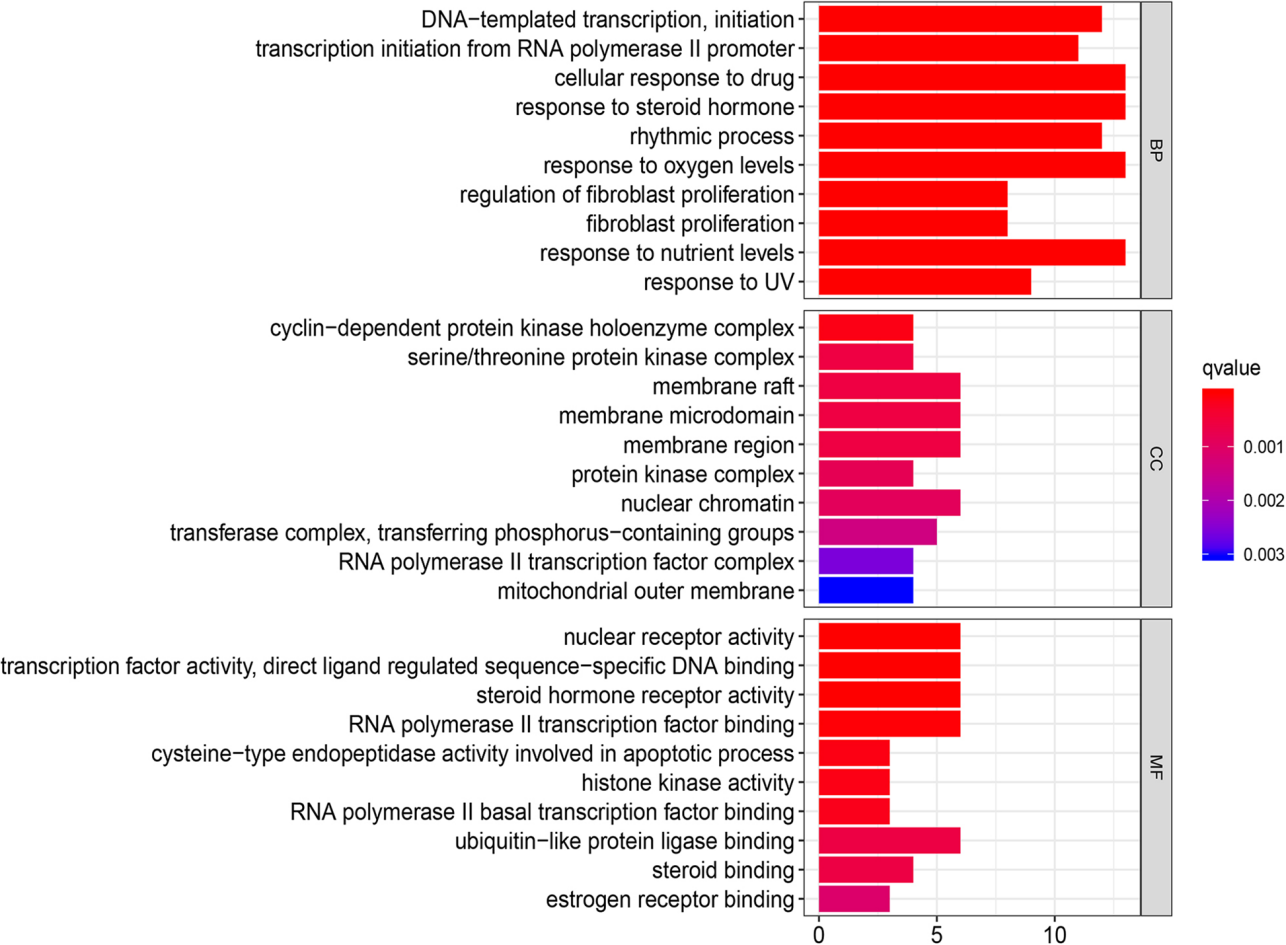
Top ten significant biological process, cell component and molecular function terms in Gene Ontology enrichment analysis

**Fig. 7:**
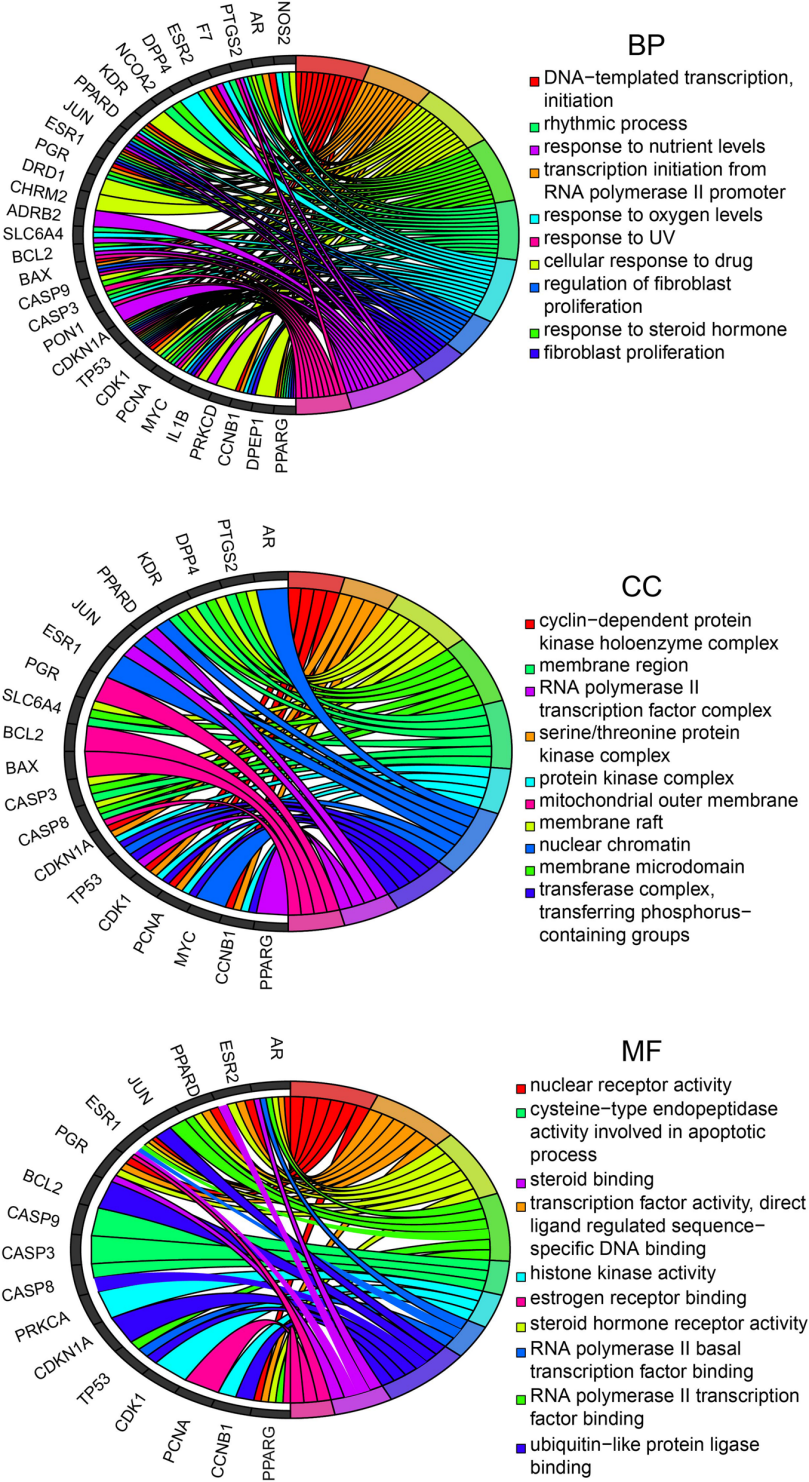
Relationships of therapeutic targets with the top 10 most significant biological process, cell component and molecular function terms in Gene Ontology enrichment analysis

To illuminate the complex mechanisms of the effects of rhubarb against DN, we conducted GO analysis of BP, CC and MF for the 37 therapeutic targets. The top 10 most significant BP, CC and MF terms, as determined using *p*-values, are listed in Fig. 6 and the relationships between the therapeutic targets and the aforementioned entries are depicted in Fig. 7. The specific entries of GO enrichment analysis for BP, CC and MF are listed in Supplementary Table 3.

**Fig. 8:**
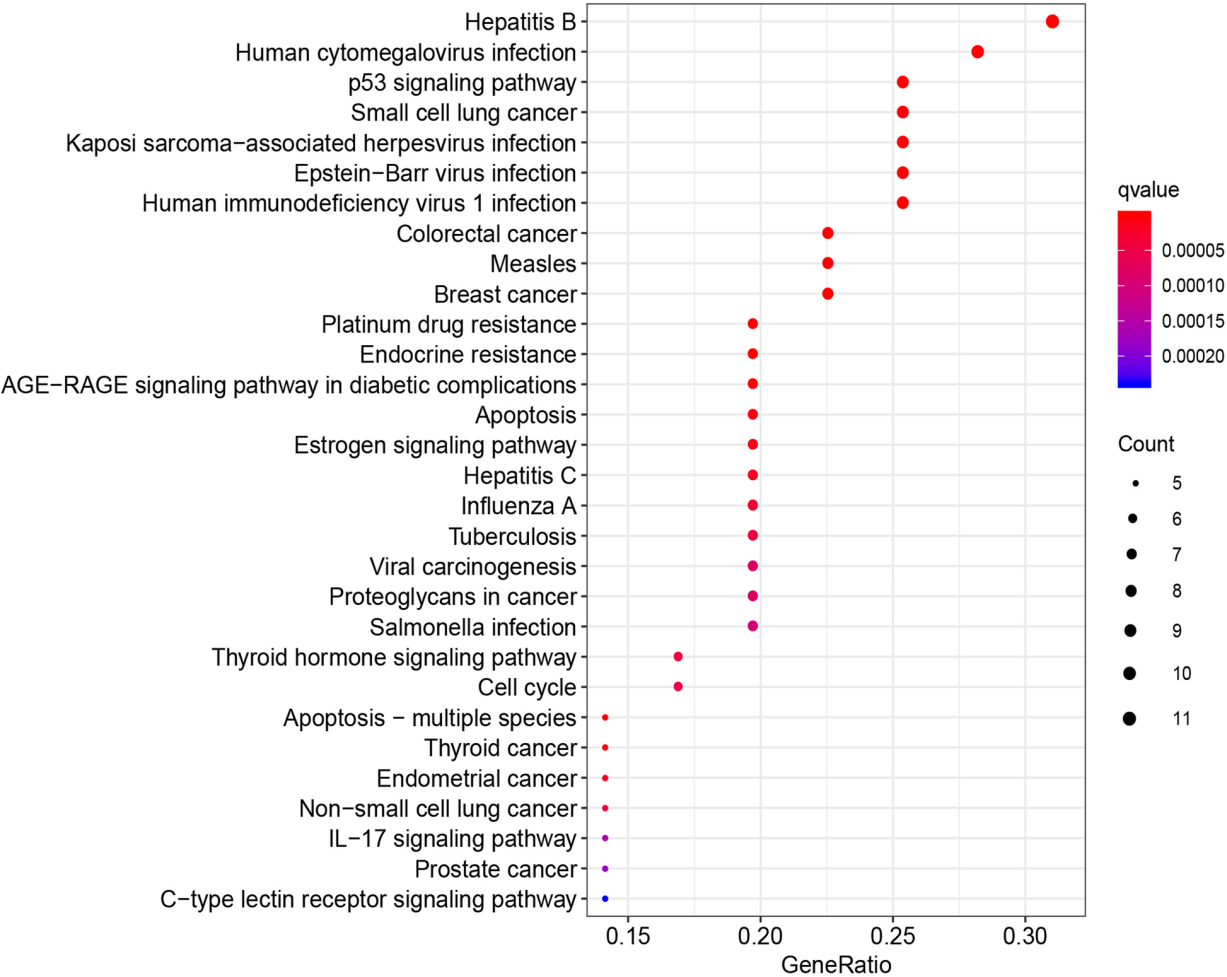
Top 30 most significant Kyoto Encyclopedia of Genes and Genomes terms

KEGG pathway enrichment analysis was performed to further clarify the underlying mechanisms of rhubarb in the treatment of DN. As presented in Supplementary Table 4, the 37 therapeutic targets were mapped into 106 KEGG pathways at *P* < 0.05. The top 30 matched KEGG pathways are depicted in Fig. 8. These 106 KEGG pathways involve human diseases, signalling pathways and pathophysiological mechanisms. The top 10 significant signalling pathways based on p-values were p53 signalling pathway, AGE-RAGE signalling pathway in diabetic complications, oestrogen signalling pathway, thyroid hormone signalling pathway, IL-17 signalling pathway, PI3K-Akt signalling pathway, TNF signalling pathway, VEGF signalling pathway, MAPK signalling pathway, ErbB signalling pathway and Wnt signalling pathway. The therapeutic targets of rhubarb and the associated DN genes in the AGE-RAGE signalling pathway are depicted in Supplementary Figure 2. These findings indicated that rhubarb may play a role in the treatment of DN by regulating the key targets in these signalling pathways, and most therapeutic targets participate in multiple signalling pathways.

## Discussion

DN is the main microvascular complication of diabetes. It significantly increases the morbidity and mortality of cardiovascular diseases, and it is one of the main complications prompting renal replacement therapy worldwide [50, 51]. Rhubarb is a well-known herbal medicine, and medicine preparations based on rhubarb have produced good results in the treatment of DN [52, 53]. However, the specific pharmacological mechanism of action of rhubarb is unclear. In this study, network pharmacology was used to explore the potential active compounds and underlying mechanisms of the effects of rhubarb against DN.

Through screening, we identified 16 active compounds in rhubarb, and after further molecular docking, rhein, beta-sitosterol and aloe-emodin were identified as the core active ingredients in rhubarb for the treatment of DN. Some of these compounds have been reported to have anti-DN effects. Rhein is a monomer of anthraquinone compounds mainly extracted from rhubarb [54]. Many studies revealed that rhein has beneficial anti-oxidant and anti-inflammatory effects on DN [12, 55], and it can alleviate renal fibrosis [56]. Emodin is a crystalline phenolic compound extracted from the root of *Rheum palmatum* [57]. It can mitigate podocyte apoptosis [58], inhibit the proliferation of fibroblasts [59], and suppress inflammation to ameliorate renal dysfunction in DN [60]. Catechin belongs to a group of polyphenolic compounds called flavanols, and it is widely distributed in plant foods [61, 62]. Some studies reported that catechin could ameliorate renal dysfunction in DN through its anti-oxidant and anti-inflammatory properties [63, 64]. These studies highlight the need for further research on the biological functions of the active compounds isolated from rhubarb.

From the therapeutic target PPI network, TP53, CASP8, CASP3, MYC, JUN and PTGS2 were selected as the core targets of rhubarb. These targets may play a major role in the therapeutic effects of rhubarb against DN. Caspases comprise a family of cysteine aspartate-specific proteases that mediate renal apoptosis [65]. Active CASP3 degrades cell-stabilising proteins and other DNA repair enzymes, resulting in apoptotic cell death [66], and attenuation of CASP3 activity in db/db mice can inhibit the progression of DN [67]. PPAR is a nuclear receptor that plays important roles in intermediary metabolism [68], and many studies suggested that the Pro12Ala polymorphism of the PPARG gene is a significant independent risk factor for DN [69, 70]. The transcription factor AP-1 (JUN) is widely involved in the transcriptional regulation of multiple genes involved in cell survival, proliferation and apoptosis [71]. JUN activation is crucial for mesangial cell proliferation and extracellular matrix production, and mesangial expansion is a key pathologic feature of DN [72, 73]. These findings indicate that the therapeutic effect of rhubarb against DN is primarily mediated through the regulation of glucose and lipid metabolism, inhibition of glomerular mesangial cell infiltration and suppression of renal fibrosis.

Next, we performed GO and KEGG enrichment analyses of the therapeutic targets. According to the GO analysis, the therapeutic targets displayed strong correlations with BP (e.g., response to steroid hormone, transcription initiation from RNA polymerase II promoter, cellular response to drugs), CC (e.g., cyclin-dependent protein kinase holoenzyme complex, serine/threonine protein kinase complex, membrane raft) and MF terms (e.g., nuclear receptor activity, steroid hormone receptor activity, histone kinase activity). Hence, rhubarb may function through the aforementioned pathways. For example, aldosterone is an endogenous steroid hormone that is involved in the formation and development of DN [74]. Aldosterone breakthrough is a clinical phenomenon that occurs upon treatment with the renin–angiotensin system (RAS) inhibitors angiotensin-converting enzyme inhibitors and angiotensin II receptor antagonists [75], and this phenomenon attenuates the organ-protective effects of RAS inhibitors against DN through the actions of mineralocorticoid receptors [76]. Several preclinical studies revealed that mineralocorticoid receptor antagonists could ameliorate or cure kidney injury and dysfunction [77, 78]. Therefore, we speculate that suppressing the response to steroid hormone and the activity of steroid hormone receptor is beneficial for the treatment of DN. Tamadher et al. found that histone H3 serine 10 phosphorylation facilitated endothelial activation in DN [79], suggesting that histone kinase activity is related to gene activation in DN.

According to the KEGG terms, the therapeutic targets of rhubarb against DN were mainly associated with the PI3K-Akt signalling pathway, p53 signalling pathway, AGE-RAGE signalling pathway in diabetic complications and MAPK signalling pathway. Podocytes maintain the glomerular filtration barrier composed of the basement membrane and slit diaphragm [80], and podocyte apoptosis was associated with increased macroalbuminuria in DN [81]. The PI3K-Akt signalling pathway is an apoptotic signalling transduction pathways, and decreased phosphorylation could give rise to podocyte apoptosis [82]. Huang et al. reported that notoginsenoside R1 could activate the PI3K-Akt signalling pathway to exert anti-apoptotic and renal-protective effects in DN mice [83]. p53 is a transcription factor, and the main outcomes of its activation are cell cycle arrest and apoptosis [84]. Evidence suggests that p53 overexpression is associated with the progression of DN, as p53 mediates podocyte apoptosis related to DN and promotes the expression of pro-fibrotic genes such as plasminogen activator inhibitor-1 [85–87]. Inflammation is associated with the development and progression of DN, and many studies support that the MAPK signalling pathway plays a central role in high glucose-induced cell damage and the activation of inflammation [88, 89]. Inhibiting the MAPK signalling pathway can reduce the inflammatory response of DN and protect the kidneys [90, 91]. Studies illustrated that p38 MAPK might play an important role in high glucose-induced epithelial–mesenchymal transition in cultured human renal tubular epithelial cells [92]. Zhong et al. reported that *Cordyceps sinensis* could mitigate epithelial–mesenchymal transition and the subsequent extracellular matrix deposition to exert a therapeutic effect on experimental diabetic renal fibrosis by inhibiting the p38 MAPK signalling pathway [1]. These findings suggest that the MAPK signalling pathway plays a role in the fibrosis associated with DN. KEGG enrichment analysis demonstrated that the therapeutic effect mechanism of the effects of rhubarb against DN may be closely related to PI3K, P53 and MAPK, suggesting that the therapeutic effect of rhubarb may be mediated through the regulation of several important factors in these signalling pathways, and most therapeutic targets participate in multiple signalling pathways.

Finally, in order to explore the accurate therapeutic mechanism of rhubarb, molecular docking was performed. The results showed that the main active compounds of rhubarb have different affinities with the central therapeutic targets. These interactions are the main forces between proteins and compounds, which form stable complexes of proteins and compounds. Molecular docking studies provide an explanation of the protein-compound interactions, which laid the foundation for further research on the therapeutic mechanism of active compounds. Interestingly, Albersmeyer et al. reported a case of AKI induced by excessive ingestion of rhubarb in the treatment of DN [93]. However, it has not been reported that regular consumption of Rhubarb caused secondary hyperoxaluria and renal failure. In addition, Liu et al. reported that the main active component of rhubarb, emodin, may provide clinical benefits in the treatment of AKI [94]. Therefore, more clinical evidence needs to be collected to further investigate the relationship between Rhubarb and AKI.

Our study still has some limitations. Our study relied on data mining and analysis, and further validation using experimental evidence is required. More databases on the composition and targets information of TCM should be included, which can make the results of our results more reliable. In summary, a comprehensive understanding of rhubarb for the treatment of DN still depends on the common development of multi-disciplines.

## Conclusion

We used network pharmacology analysis and molecular docking technology to explore the potential mechanism of the effects of rhubarb against DN, clarifying the core active ingredients in rhubarb were rhein, beta-sitosterol and aloe-emodin, and the key therapeutic targets were TP53, CASP8, CASP3, MYC, JUN and PTGS2. The therapeutic properties of rhubarb against DN arise from the regulation of biological pathways involved in podocyte apoptosis, inflammation and renal interstitial fibrosis. These findings demonstrate the importance of understanding traditional Chinese medicines and establish a base for further research on the pathogenesis of DN, as well as the development of new drugs.

## Supplementary Information

**Additional file 1.**

**Additional file 2.**

**Additional file 3.**

**Additional file 4.**

**Additional file 5.**

**Additional file 6.**

## Data Availability

The datasets are included in this published article and its supplementary files. The algorithms used to process the data are available from the corresponding author on reasonable request. Please send the permissions document to evaxiaohouzi@jlu.edu.cn.
