# Supplementary figures and images for: Network pharmacology and molecular docking technology-based predictive study of the active ingredients and potential targets of rhubarb for the treatment of diabetic nephropathy
Source: BMC Complement Med Ther. 2022 Aug 6;22:210. doi: 10.1186/s12906-022-03662-6 (PMC9356435; doi:10.1186/s12906-022-03662-6)

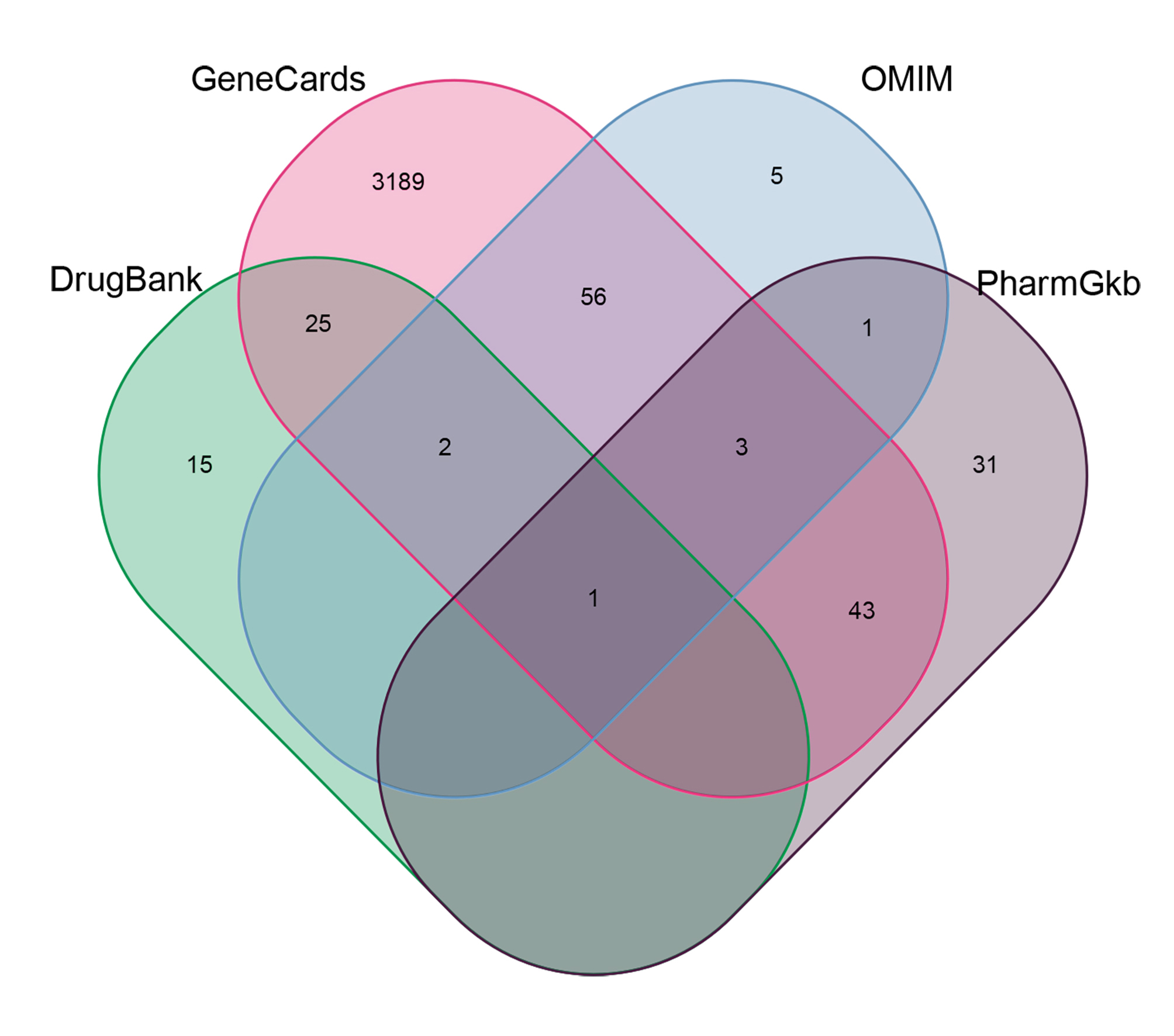

Supplement: Supplementary file 1 — Additional file 1. [file 12906_2022_3662_MOESM1_ESM.jpg]

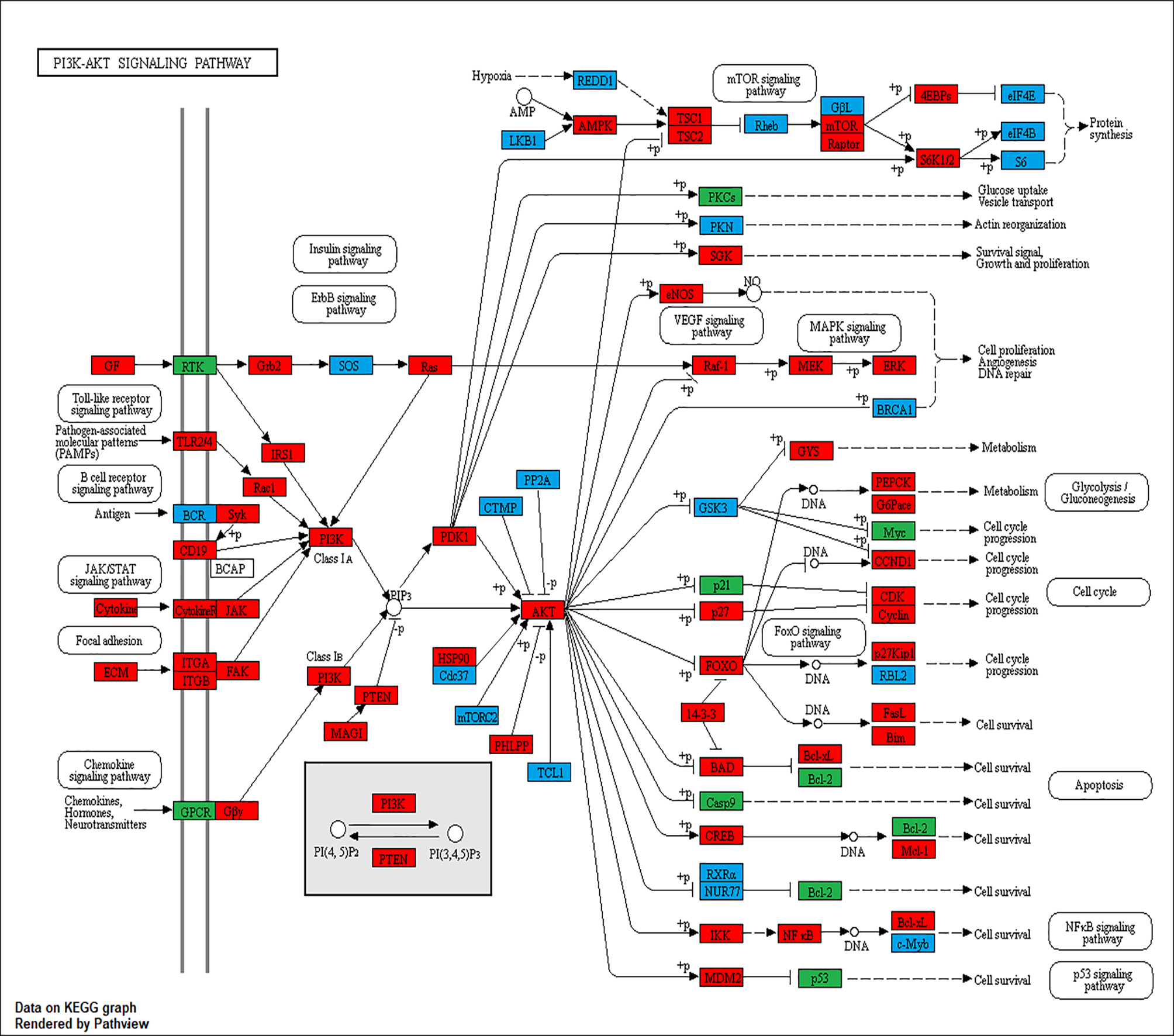

Supplement: Supplementary file 2 — Additional file 2. [file 12906_2022_3662_MOESM2_ESM.jpg]
