# Supplementary material for: Network pharmacology and molecular docking technology-based predictive study of the active ingredients and potential targets of rhubarb for the treatment of diabetic nephropathy
Source: BMC Complement Med Ther. 2022 Aug 6;22:210. doi: 10.1186/s12906-022-03662-6 (PMC9356435; doi:10.1186/s12906-022-03662-6)
Supplement: Supplementary file 3 — Additional file 3. [file 12906_2022_3662_MOESM3_ESM.docx]

| **Prediction of Targets of Dahuang** | | | | |
| --- | --- | --- | --- | --- |
| MolId | MolName | Protein names | Symbol | Database |
| MOL002235 | EUPATIN | Nitric oxide synthase, inducible | NOS2 | DrugBank |
| MOL002235 | EUPATIN | Androgen receptor | AR | DrugBank |
| MOL002235 | EUPATIN | Prostaglandin G/H synthase 2 | PTGS2 | DrugBank |
| MOL002235 | EUPATIN | Coagulation factor VII | F7 | DrugBank |
| MOL002235 | EUPATIN | Estrogen receptor beta | ESR2 | DrugBank |
| MOL002235 | EUPATIN | Dipeptidyl peptidase 4 | DPP4 | DrugBank |
| MOL002235 | EUPATIN | Heat shock protein HSP 90-beta | HSP90AB1 | DrugBank |
| MOL002235 | EUPATIN | Trypsin-1 | PRSS1 | DrugBank |
| MOL002235 | EUPATIN | Nuclear receptor coactivator 2 | NCOA2 | DrugBank |
| MOL002235 | EUPATIN | Calmodulin-lysine N-methyltransferase | CAMKMT | DrugBank |
| MOL002235 | EUPATIN | Sodium channel protein type 5 subunit alpha | SCN5A | DrugBank |
| MOL002235 | EUPATIN | Vascular endothelial growth factor receptor 2 | KDR | DrugBank |
| MOL002235 | EUPATIN | Peroxisome proliferator-activated receptor delta | PPARD | DrugBank |
| MOL002268 | rhein | Prostaglandin G/H synthase 1 | PTGS1 | DrugBank |
| MOL002268 | rhein | Prostaglandin G/H synthase 2 | PTGS2 | DrugBank |
| MOL002268 | rhein | Heat shock protein HSP 90-beta | HSP90AB1 | DrugBank |
| MOL002268 | rhein | Nuclear receptor coactivator 2 | NCOA2 | DrugBank |
| MOL002268 | rhein | Aldo-keto reductase family 1 member B1 | AKR1B1 | DrugBank |
| MOL002268 | rhein | Transcription factor AP-1 | JUN | DrugBank |
| MOL002281 | Toralactone | Nitric oxide synthase, inducible | NOS2 | DrugBank |
| MOL002281 | Toralactone | Prostaglandin G/H synthase 1 | PTGS1 | DrugBank |
| MOL002281 | Toralactone | Estrogen receptor | ESR1 | DrugBank |
| MOL002281 | Toralactone | Prostaglandin G/H synthase 2 | PTGS2 | DrugBank |
| MOL002281 | Toralactone | Estrogen receptor beta | ESR2 | DrugBank |
| MOL002281 | Toralactone | Heat shock protein HSP 90-beta | HSP90AB1 | DrugBank |
| MOL002281 | Toralactone | Serine/threonine-protein kinase Chk1 | CHEK1 | DrugBank |
| MOL002297 | Daucosterol_qt | Progesterone receptor | PGR | DrugBank |
| MOL002297 | Daucosterol_qt | Nuclear receptor coactivator 2 | NCOA2 | DrugBank |
| MOL000358 | beta-sitosterol | Progesterone receptor | PGR | DrugBank |
| MOL000358 | beta-sitosterol | Nuclear receptor coactivator 2 | NCOA2 | DrugBank |
| MOL000358 | beta-sitosterol | Prostaglandin G/H synthase 1 | PTGS1 | DrugBank |
| MOL000358 | beta-sitosterol | Prostaglandin G/H synthase 2 | PTGS2 | DrugBank |
| MOL000358 | beta-sitosterol | Heat shock protein HSP 90-beta | HSP90AB1 | DrugBank |
| MOL000358 | beta-sitosterol | Potassium voltage-gated channel subfamily H member 2 | KCNH2 | DrugBank |
| MOL000358 | beta-sitosterol | D(1A) dopamine receptor | DRD1 | DrugBank |
| MOL000358 | beta-sitosterol | Muscarinic acetylcholine receptor M3 | CHRM3 | DrugBank |
| MOL000358 | beta-sitosterol | Muscarinic acetylcholine receptor M1 | CHRM1 | DrugBank |
| MOL000358 | beta-sitosterol | Sodium channel protein type 5 subunit alpha | SCN5A | DrugBank |
| MOL000358 | beta-sitosterol | Muscarinic acetylcholine receptor M4 | CHRM4 | DrugBank |
| MOL000358 | beta-sitosterol | Alpha-1A adrenergic receptor | ADRA1A | DrugBank |
| MOL000358 | beta-sitosterol | Muscarinic acetylcholine receptor M2 | CHRM2 | DrugBank |
| MOL000358 | beta-sitosterol | Alpha-1B adrenergic receptor | ADRA1B | DrugBank |
| MOL000358 | beta-sitosterol | Beta-2 adrenergic receptor | ADRB2 | DrugBank |
| MOL000358 | beta-sitosterol | Neuronal acetylcholine receptor subunit alpha-2 | CHRNA2 | DrugBank |
| MOL000358 | beta-sitosterol | Sodium-dependent serotonin transporter | SLC6A4 | DrugBank |
| MOL000358 | beta-sitosterol | Mu-type opioid receptor | OPRM1 | DrugBank |
| MOL000358 | beta-sitosterol | Gamma-aminobutyric acid receptor subunit alpha-1 | GABRA1 | DrugBank |
| MOL000358 | beta-sitosterol | Apoptosis regulator Bcl-2 | BCL2 | DrugBank |
| MOL000358 | beta-sitosterol | Apoptosis regulator BAX | BAX | DrugBank |
| MOL000358 | beta-sitosterol | Caspase-9 | CASP9 | DrugBank |
| MOL000358 | beta-sitosterol | Transcription factor AP-1 | JUN | DrugBank |
| MOL000358 | beta-sitosterol | Caspase-3 | CASP3 | DrugBank |
| MOL000358 | beta-sitosterol | Caspase-8 | CASP8 | DrugBank |
| MOL000358 | beta-sitosterol | Protein kinase C alpha type | PRKCA | DrugBank |
| MOL000358 | beta-sitosterol | Serum paraoxonase/arylesterase 1 | PON1 | DrugBank |
| MOL000358 | beta-sitosterol | Microtubule-associated protein 2 | MAP2 | DrugBank |
| MOL000471 | aloe-emodin | Prostaglandin G/H synthase 1 | PTGS1 | DrugBank |
| MOL000471 | aloe-emodin | Prostaglandin G/H synthase 2 | PTGS2 | DrugBank |
| MOL000471 | aloe-emodin | Heat shock protein HSP 90-beta | HSP90AB1 | DrugBank |
| MOL000471 | aloe-emodin | Nuclear receptor coactivator 2 | NCOA2 | DrugBank |
| MOL000471 | aloe-emodin | cAMP-dependent protein kinase inhibitor alpha | PKIA | DrugBank |
| MOL000471 | aloe-emodin | Aldo-keto reductase family 1 member B1 | AKR1B1 | DrugBank |
| MOL000471 | aloe-emodin | Immunoglobulin heavy constant gamma 1 | IGHG1 | DrugBank |
| MOL000471 | aloe-emodin | Cyclin-dependent kinase inhibitor 1 | CDKN1A | DrugBank |
| MOL000471 | aloe-emodin | Eukaryotic translation initiation factor 6 | EIF6 | DrugBank |
| MOL000471 | aloe-emodin | Apoptosis regulator BAX | BAX | DrugBank |
| MOL000471 | aloe-emodin | Tumor necrosis factor-inducible gene 6 protein | TNFAIP6 | DrugBank |
| MOL000471 | aloe-emodin | Caspase-3 | CASP3 | DrugBank |
| MOL000471 | aloe-emodin | Cellular tumor antigen p53 | TP53 | DrugBank |
| MOL000471 | aloe-emodin | Fatty acid synthase | FASN | DrugBank |
| MOL000471 | aloe-emodin | Protein kinase C alpha type | PRKCA | DrugBank |
| MOL000471 | aloe-emodin | Protein kinase C epsilon type | PRKCE | DrugBank |
| MOL000471 | aloe-emodin | Cyclin-dependent kinase 1 | CDK1 | DrugBank |
| MOL000471 | aloe-emodin | Proliferating cell nuclear antigen | PCNA | DrugBank |
| MOL000471 | aloe-emodin | Myc proto-oncogene protein | MYC | DrugBank |
| MOL000471 | aloe-emodin | Interleukin-1 beta | IL1B | DrugBank |
| MOL000471 | aloe-emodin | Protein kinase C delta type | PRKCD | DrugBank |
| MOL000471 | aloe-emodin | G2/mitotic-specific cyclin-B1 | CCNB1 | DrugBank |
| MOL000096 | (-)-catechin | Prostaglandin G/H synthase 1 | PTGS1 | DrugBank |
| MOL000096 | (-)-catechin | Estrogen receptor | ESR1 | DrugBank |
| MOL000096 | (-)-catechin | Prostaglandin G/H synthase 2 | PTGS2 | DrugBank |
| MOL000096 | (-)-catechin | Heat shock protein HSP 90-beta | HSP90AB1 | DrugBank |
| MOL000096 | (-)-catechin | Dipeptidase 1 | DPEP1 | DrugBank |
| MOL000096 | (-)-catechin | Nuclear receptor coactivator 2 | NCOA2 | DrugBank |
| MOL000096 | (-)-catechin | Calmodulin-lysine N-methyltransferase | CAMKMT | DrugBank |
| MOL000096 | (-)-catechin | Fatty acid synthase | FASN | DrugBank |
| MOL000096 | (-)-catechin | Peroxisome proliferator-activated receptor gamma | PPARG | DrugBank |
| MOL000096 | (-)-catechin | Krueppel-like factor 7 | KLF7 | DrugBank |
