# Supplementary material for: Network pharmacology and molecular docking technology-based predictive study of the active ingredients and potential targets of rhubarb for the treatment of diabetic nephropathy
Source: BMC Complement Med Ther. 2022 Aug 6;22:210. doi: 10.1186/s12906-022-03662-6 (PMC9356435; doi:10.1186/s12906-022-03662-6)
Supplement: Supplementary file 4 — Additional file 4. [file 12906_2022_3662_MOESM4_ESM.docx]

| Gene Symbol | Database |
| --- | --- |
| INS | GeneCards |
| HNF1B | GeneCards |
| KCNJ11 | GeneCards |
| GCK | GeneCards |
| ACE | GeneCards |
| ABCC8 | GeneCards |
| HNF1A | GeneCards |
| HNF4A | GeneCards |
| PPARG | GeneCards |
| PDX1 | GeneCards |
| REN | GeneCards |
| NEUROD1 | GeneCards |
| INSR | GeneCards |
| WFS1 | GeneCards |
| VEGFA | GeneCards |
| PAX4 | GeneCards |
| IL6 | GeneCards |
| UMOD | GeneCards |
| AGTR1 | GeneCards |
| LEP | GeneCards |
| KLF11 | GeneCards |
| AGT | GeneCards |
| ALB | GeneCards |
| IGF1 | GeneCards |
| CCL2 | GeneCards |
| PON1 | GeneCards |
| BLK | GeneCards |
| TCF7L2 | GeneCards |
| NOS3 | GeneCards |
| APOA1 | GeneCards |
| SLC2A2 | GeneCards |
| B2M | GeneCards |
| IRS1 | GeneCards |
| RETN | GeneCards |
| TGFB1 | GeneCards |
| SOD2 | GeneCards |
| SLC30A8 | GeneCards |
| COL4A1 | GeneCards |
| AQP2 | GeneCards |
| HFE | GeneCards |
| APOE | GeneCards |
| GLIS3 | GeneCards |
| MIR21 | GeneCards |
| ADIPOQ | GeneCards |
| FOXP3 | GeneCards |
| IL1RN | GeneCards |
| AVP | GeneCards |
| HLA-DRB1 | GeneCards |
| TNF | GeneCards |
| AKT1 | GeneCards |
| SLC2A4 | GeneCards |
| PTF1A | GeneCards |
| TP53 | GeneCards |
| ENPP1 | GeneCards |
| CTLA4 | GeneCards |
| HLA-DQA1 | GeneCards |
| LEPR | GeneCards |
| NLRP3 | GeneCards |
| ALMS1 | GeneCards |
| EPO | GeneCards |
| IGF2BP2 | GeneCards |
| SLC2A1 | GeneCards |
| AKR1B1 | GeneCards |
| IGF2 | GeneCards |
| CAPN10 | GeneCards |
| BSCL2 | GeneCards |
| IL2RA | GeneCards |
| IRS2 | GeneCards |
| HLA-DQB1 | GeneCards |
| MT-TL1 | GeneCards |
| CRP | GeneCards |
| EIF2AK3 | GeneCards |
| STAT3 | GeneCards |
| TTR | GeneCards |
| AGER | GeneCards |
| KCNQ1 | GeneCards |
| MTNR1B | GeneCards |
| MT-ND1 | GeneCards |
| ZFP57 | GeneCards |
| CEL | GeneCards |
| ADRB3 | GeneCards |
| PTH | GeneCards |
| PTPN22 | GeneCards |
| LPL | GeneCards |
| POMC | GeneCards |
| FN1 | GeneCards |
| AGPAT2 | GeneCards |
| CCR5 | GeneCards |
| IFIH1 | GeneCards |
| PIK3R1 | GeneCards |
| NEUROG3 | GeneCards |
| MC4R | GeneCards |
| SLC5A2 | GeneCards |
| GCG | GeneCards |
| SLC12A3 | GeneCards |
| AIRE | GeneCards |
| STAT1 | GeneCards |
| GHR | GeneCards |
| ADRB2 | GeneCards |
| WT1 | GeneCards |
| IGF1R | GeneCards |
| WRN | GeneCards |
| PCBD1 | GeneCards |
| NPHS1 | GeneCards |
| BMP6 | GeneCards |
| SUMO4 | GeneCards |
| CDKN2A | GeneCards |
| MAFB | GeneCards |
| FOS | GeneCards |
| NPHP1 | GeneCards |
| CD79A | GeneCards |
| HRAS | GeneCards |
| IAPP | GeneCards |
| IL1B | GeneCards |
| AVPR2 | GeneCards |
| F2 | GeneCards |
| SERPINE1 | GeneCards |
| ICAM1 | GeneCards |
| DNAJC3 | GeneCards |
| APPL1 | GeneCards |
| G6PC | GeneCards |
| CTNNB1 | GeneCards |
| PLAGL1 | GeneCards |
| GATA6 | GeneCards |
| CASR | GeneCards |
| GNAS | GeneCards |
| DPP4 | GeneCards |
| BDNF | GeneCards |
| IL10 | GeneCards |
| SAA1 | GeneCards |
| XPNPEP3 | GeneCards |
| FLT1 | GeneCards |
| CCN2 | GeneCards |
| APOA2 | GeneCards |
| MBL2 | GeneCards |
| LCN2 | GeneCards |
| APOB | GeneCards |
| VCAM1 | GeneCards |
| LMNA | GeneCards |
| GAD2 | GeneCards |
| FOXC2 | GeneCards |
| CLCNKB | GeneCards |
| PRKCB | GeneCards |
| HK1 | GeneCards |
| EDN1 | GeneCards |
| ATM | GeneCards |
| CST3 | GeneCards |
| CXCL8 | GeneCards |
| DMPK | GeneCards |
| C3 | GeneCards |
| MUC1 | GeneCards |
| CD40LG | GeneCards |
| HLA-DPB1 | GeneCards |
| RBP4 | GeneCards |
| TLR5 | GeneCards |
| RFX6 | GeneCards |
| HPRT1 | GeneCards |
| MTHFR | GeneCards |
| IL18 | GeneCards |
| CDKAL1 | GeneCards |
| NAGLU | GeneCards |
| MEG3 | GeneCards |
| SOD1 | GeneCards |
| TLR4 | GeneCards |
| MIR192 | GeneCards |
| TSC1 | GeneCards |
| FTO | GeneCards |
| AMBP | GeneCards |
| MT-TE | GeneCards |
| NPPA | GeneCards |
| IGFBP1 | GeneCards |
| AGRP | GeneCards |
| MT-ND4 | GeneCards |
| MIR17 | GeneCards |
| MMP2 | GeneCards |
| VDR | GeneCards |
| SST | GeneCards |
| PTPRN | GeneCards |
| CAT | GeneCards |
| FGA | GeneCards |
| CISD2 | GeneCards |
| MIR140 | GeneCards |
| MYH9 | GeneCards |
| TSC2 | GeneCards |
| IL4 | GeneCards |
| NPHP4 | GeneCards |
| MIR483 | GeneCards |
| MAFA | GeneCards |
| TH | GeneCards |
| HYMAI | GeneCards |
| SLC19A2 | GeneCards |
| CAV1 | GeneCards |
| MMP9 | GeneCards |
| PRODH | GeneCards |
| IGFBP3 | GeneCards |
| COL4A3 | GeneCards |
| GAD1 | GeneCards |
| BGLAP | GeneCards |
| CETP | GeneCards |
| NPHS2 | GeneCards |
| APOC3 | GeneCards |
| RHO | GeneCards |
| SLC12A1 | GeneCards |
| LPA | GeneCards |
| CAVIN1 | GeneCards |
| IDDM7 | GeneCards |
| IDDM8 | GeneCards |
| IDDM4 | GeneCards |
| BBS2 | GeneCards |
| LHX1 | GeneCards |
| IDDM13 | GeneCards |
| IDDM11 | GeneCards |
| NAMPT | GeneCards |
| IDDM3 | GeneCards |
| INS-IGF2 | GeneCards |
| HP | GeneCards |
| PVT1 | GeneCards |
| EGF | GeneCards |
| MIR155 | GeneCards |
| COL4A5 | GeneCards |
| AKT2 | GeneCards |
| PPARA | GeneCards |
| MT-CO2 | GeneCards |
| SELE | GeneCards |
| SGK1 | GeneCards |
| CNDP1 | GeneCards |
| VWF | GeneCards |
| MME | GeneCards |
| CCR6 | GeneCards |
| TLR2 | GeneCards |
| CEP290 | GeneCards |
| CDKN3 | GeneCards |
| AOC3 | GeneCards |
| NPY | GeneCards |
| HBB | GeneCards |
| IFNG | GeneCards |
| H2AC18 | GeneCards |
| HBA1 | GeneCards |
| UCP2 | GeneCards |
| SERPINB7 | GeneCards |
| CFTR | GeneCards |
| UCP1 | GeneCards |
| NOS2 | GeneCards |
| HIF1A | GeneCards |
| MEN1 | GeneCards |
| THBD | GeneCards |
| MTOR | GeneCards |
| COL4A4 | GeneCards |
| GGT1 | GeneCards |
| LTA | GeneCards |
| ST3GAL4 | GeneCards |
| GH1 | GeneCards |
| IGFBP2 | GeneCards |
| CXCL10 | GeneCards |
| CFHR5 | GeneCards |
| MIR377 | GeneCards |
| LIPC | GeneCards |
| FGF2 | GeneCards |
| TNFRSF11B | GeneCards |
| CD4 | GeneCards |
| HMGCR | GeneCards |
| GAPDH | GeneCards |
| MIR20A | GeneCards |
| ITPR3 | GeneCards |
| HMOX1 | GeneCards |
| IDDM15 | GeneCards |
| IDDM6 | GeneCards |
| MT-TK | GeneCards |
| IL2 | GeneCards |
| IDDM17 | GeneCards |
| AR | GeneCards |
| AGTR2 | GeneCards |
| BBS1 | GeneCards |
| IL17A | GeneCards |
| IDDM23 | GeneCards |
| IDDM24 | GeneCards |
| APOA4 | GeneCards |
| MT-CO1 | GeneCards |
| MAPK1 | GeneCards |
| SELL | GeneCards |
| IDDM18 | GeneCards |
| NOD2 | GeneCards |
| PF4 | GeneCards |
| TPO | GeneCards |
| PIK3CG | GeneCards |
| HHEX | GeneCards |
| GCKR | GeneCards |
| NPPB | GeneCards |
| GHRL | GeneCards |
| HLA-A | GeneCards |
| MIR27A | GeneCards |
| MIR30A | GeneCards |
| SELP | GeneCards |
| SIRT1 | GeneCards |
| CP | GeneCards |
| ESR1 | GeneCards |
| ACP1 | GeneCards |
| CFHR1 | GeneCards |
| MAPK8 | GeneCards |
| MPO | GeneCards |
| COG2 | GeneCards |
| PAX6 | GeneCards |
| MAPK14 | GeneCards |
| AHSG | GeneCards |
| RPGRIP1L | GeneCards |
| MEFV | GeneCards |
| TMEM67 | GeneCards |
| GFAP | GeneCards |
| BBS7 | GeneCards |
| XBP1 | GeneCards |
| MT-ND6 | GeneCards |
| VTN | GeneCards |
| SOCS1 | GeneCards |
| SOCS3 | GeneCards |
| LCAT | GeneCards |
| MIR126 | GeneCards |
| CRYAA | GeneCards |
| TNFRSF1A | GeneCards |
| GLP1R | GeneCards |
| APOC2 | GeneCards |
| PTGS2 | GeneCards |
| TLR9 | GeneCards |
| MIR145 | GeneCards |
| TLR3 | GeneCards |
| BBS4 | GeneCards |
| HLA-B | GeneCards |
| SNRPN | GeneCards |
| LMX1B | GeneCards |
| SHH | GeneCards |
| FGF21 | GeneCards |
| PRKCD | GeneCards |
| GSR | GeneCards |
| IFT88 | GeneCards |
| FABP2 | GeneCards |
| CASP3 | GeneCards |
| MT-TS2 | GeneCards |
| NPHP3 | GeneCards |
| UCP3 | GeneCards |
| GLA | GeneCards |
| SERPINA3 | GeneCards |
| LEPQTL1 | GeneCards |
| AHI1 | GeneCards |
| MGAM | GeneCards |
| REG1A | GeneCards |
| ADM | GeneCards |
| ATF6 | GeneCards |
| SPP1 | GeneCards |
| COQ2 | GeneCards |
| FASLG | GeneCards |
| ICOSLG | GeneCards |
| UTS2 | GeneCards |
| MAPK3 | GeneCards |
| TF | GeneCards |
| SCT | GeneCards |
| MT-ATP6 | GeneCards |
| IL1R1 | GeneCards |
| CYP3A4 | GeneCards |
| CD8A | GeneCards |
| SERPINF1 | GeneCards |
| PGF | GeneCards |
| ELN | GeneCards |
| ANGPT2 | GeneCards |
| MKS1 | GeneCards |
| NR3C2 | GeneCards |
| NKX6-1 | GeneCards |
| PRL | GeneCards |
| PTPN1 | GeneCards |
| MT-CO3 | GeneCards |
| TREX1 | GeneCards |
| CCL5 | GeneCards |
| PMM2 | GeneCards |
| SORD | GeneCards |
| HSPD1 | GeneCards |
| CFH | GeneCards |
| CDKN1C | GeneCards |
| BBS5 | GeneCards |
| POLG | GeneCards |
| MT-ND5 | GeneCards |
| KIF7 | GeneCards |
| IGF2R | GeneCards |
| IFT140 | GeneCards |
| IFT172 | GeneCards |
| SEC61A1 | GeneCards |
| NGF | GeneCards |
| CPE | GeneCards |
| CYP27B1 | GeneCards |
| CD28 | GeneCards |
| TTC21B | GeneCards |
| SLC30A10 | GeneCards |
| PCSK1 | GeneCards |
| HGF | GeneCards |
| SRC | GeneCards |
| JUN | GeneCards |
| UBD | GeneCards |
| STAT5B | GeneCards |
| APOA5 | GeneCards |
| CYP21A2 | GeneCards |
| HSPA1A | GeneCards |
| CYP1A2 | GeneCards |
| KDR | GeneCards |
| TAP2 | GeneCards |
| GC | GeneCards |
| CD40 | GeneCards |
| SI | GeneCards |
| ACE2 | GeneCards |
| IL1A | GeneCards |
| LNPEP | GeneCards |
| ABCA1 | GeneCards |
| CDC123 | GeneCards |
| PRTN3 | GeneCards |
| FABP1 | GeneCards |
| HAVCR1 | GeneCards |
| ITGAM | GeneCards |
| PTPRN2 | GeneCards |
| PPY | GeneCards |
| CASP1 | GeneCards |
| DLK1 | GeneCards |
| PLG | GeneCards |
| INF2 | GeneCards |
| ICA1 | GeneCards |
| TLR1 | GeneCards |
| PPP1R15B | GeneCards |
| TGM2 | GeneCards |
| HSPA4 | GeneCards |
| CYP19A1 | GeneCards |
| TMEM216 | GeneCards |
| ADD1 | GeneCards |
| APRT | GeneCards |
| MT-TW | GeneCards |
| NOTCH3 | GeneCards |
| CD80 | GeneCards |
| SLC5A1 | GeneCards |
| SLC5A4 | GeneCards |
| MALAT1 | GeneCards |
| THADA | GeneCards |
| MT-TQ | GeneCards |
| KCNQ1OT1 | GeneCards |
| MIR181A2 | GeneCards |
| HK2 | GeneCards |
| C4A | GeneCards |
| TRH | GeneCards |
| MIR146A | GeneCards |
| ERN1 | GeneCards |
| SDCCAG8 | GeneCards |
| LRP5 | GeneCards |
| SAMHD1 | GeneCards |
| F8 | GeneCards |
| TAP1 | GeneCards |
| PKD2 | GeneCards |
| TG | GeneCards |
| IL6R | GeneCards |
| MT-TF | GeneCards |
| MT-TS1 | GeneCards |
| BRAF | GeneCards |
| NTS | GeneCards |
| OFD1 | GeneCards |
| HSD11B2 | GeneCards |
| PRKAB1 | GeneCards |
| RPGR | GeneCards |
| IKBKB | GeneCards |
| CCND1 | GeneCards |
| TGFB2 | GeneCards |
| FOXA2 | GeneCards |
| TLR10 | GeneCards |
| CFHR2 | GeneCards |
| ESR2 | GeneCards |
| PTEN | GeneCards |
| F3 | GeneCards |
| PECAM1 | GeneCards |
| IGFBP7 | GeneCards |
| CXCL12 | GeneCards |
| SNCA | GeneCards |
| G6PC2 | GeneCards |
| JAK2 | GeneCards |
| PTPN3 | GeneCards |
| MT-TH | GeneCards |
| COL4A2 | GeneCards |
| H19 | GeneCards |
| GATA3 | GeneCards |
| TKT | GeneCards |
| FGF23 | GeneCards |
| PIK3CA | GeneCards |
| IFT80 | GeneCards |
| PARP1 | GeneCards |
| PCK1 | GeneCards |
| TIMP3 | GeneCards |
| TNFSF11 | GeneCards |
| TLR8 | GeneCards |
| CD86 | GeneCards |
| JAZF1 | GeneCards |
| HBA2 | GeneCards |
| TRPV4 | GeneCards |
| IL13 | GeneCards |
| CD2AP | GeneCards |
| AGXT | GeneCards |
| HSPA5 | GeneCards |
| ROBO2 | GeneCards |
| PCK2 | GeneCards |
| EP300 | GeneCards |
| IL18R1 | GeneCards |
| HTR1A | GeneCards |
| ONECUT1 | GeneCards |
| IFNA1 | GeneCards |
| CCL11 | GeneCards |
| TLR6 | GeneCards |
| PSMB8 | GeneCards |
| IL15 | GeneCards |
| KDM4C | GeneCards |
| BLM | GeneCards |
| TNFRSF25 | GeneCards |
| SPINK1 | GeneCards |
| TLR7 | GeneCards |
| OXT | GeneCards |
| FGF8 | GeneCards |
| TWNK | GeneCards |
| SIM1 | GeneCards |
| ADORA1 | GeneCards |
| ISL1 | GeneCards |
| CNTF | GeneCards |
| CFB | GeneCards |
| LRP2 | GeneCards |
| FXYD2 | GeneCards |
| RFC2 | GeneCards |
| XDH | GeneCards |
| HMGB1 | GeneCards |
| WDR19 | GeneCards |
| GAL | GeneCards |
| PAX2 | GeneCards |
| APP | GeneCards |
| GRHPR | GeneCards |
| PRDM10 | GeneCards |
| CYCS | GeneCards |
| BMP7 | GeneCards |
| MIR199A1 | GeneCards |
| CC2D2A | GeneCards |
| WDR4 | GeneCards |
| TIMP1 | GeneCards |
| FABP12 | GeneCards |
| NR3C1 | GeneCards |
| IL5 | GeneCards |
| IFT27 | GeneCards |
| CREB1 | GeneCards |
| HLA-DMA | GeneCards |
| MIR144 | GeneCards |
| PCSK2 | GeneCards |
| PRKAR1A | GeneCards |
| CD151 | GeneCards |
| CRH | GeneCards |
| BSND | GeneCards |
| TBX18 | GeneCards |
| SERPINC1 | GeneCards |
| RAC1 | GeneCards |
| NR1H2 | GeneCards |
| MAPT | GeneCards |
| C12orf43 | GeneCards |
| FAS | GeneCards |
| PPARGC1A | GeneCards |
| PSTPIP1 | GeneCards |
| ANGPT1 | GeneCards |
| SHBG | GeneCards |
| CD38 | GeneCards |
| MIR125A | GeneCards |
| HLA-DMB | GeneCards |
| ARNTL | GeneCards |
| KRAS | GeneCards |
| PKHD1 | GeneCards |
| MIR342 | GeneCards |
| MLXIPL | GeneCards |
| HSPG2 | GeneCards |
| PTPN2 | GeneCards |
| MASP1 | GeneCards |
| SCGB1A1 | GeneCards |
| TMEM231 | GeneCards |
| MIR675 | GeneCards |
| MKRN3 | GeneCards |
| ADAR | GeneCards |
| INVS | GeneCards |
| ACTN4 | GeneCards |
| PBX1 | GeneCards |
| PTPRC | GeneCards |
| RPS6KB1 | GeneCards |
| PLCG1 | GeneCards |
| NPS | GeneCards |
| VIPAS39 | GeneCards |
| CTSB | GeneCards |
| SLPI | GeneCards |
| MAF | GeneCards |
| MT-ND2 | GeneCards |
| FBN1 | GeneCards |
| PDGFB | GeneCards |
| F5 | GeneCards |
| PTX3 | GeneCards |
| PDCD1 | GeneCards |
| MFN2 | GeneCards |
| SLC22A11 | GeneCards |
| F9 | GeneCards |
| SURF1 | GeneCards |
| TRPC6 | GeneCards |
| SYNPO | GeneCards |
| GJB2 | GeneCards |
| TFAM | GeneCards |
| ACTB | GeneCards |
| THBS1 | GeneCards |
| MIR142 | GeneCards |
| MMP1 | GeneCards |
| MTTP | GeneCards |
| SERPINF2 | GeneCards |
| LRBA | GeneCards |
| MIR146B | GeneCards |
| PTGDS | GeneCards |
| APOL1 | GeneCards |
| MEST | GeneCards |
| TNFRSF10A | GeneCards |
| HLA-DRB3 | GeneCards |
| CASP8 | GeneCards |
| PLAT | GeneCards |
| RNASEH2A | GeneCards |
| ZNF423 | GeneCards |
| TFRC | GeneCards |
| FOXM1 | GeneCards |
| IL7R | GeneCards |
| WDTC1 | GeneCards |
| CLU | GeneCards |
| GZMB | GeneCards |
| CLTRN | GeneCards |
| G6PD | GeneCards |
| CCL3 | GeneCards |
| TIMP2 | GeneCards |
| PSMB9 | GeneCards |
| CS | GeneCards |
| HLA-E | GeneCards |
| ACSL4 | GeneCards |
| MIR217 | GeneCards |
| RNASEH2C | GeneCards |
| RNASEH2B | GeneCards |
| SPRY2 | GeneCards |
| PDE11A | GeneCards |
| KCNJ1 | GeneCards |
| PLEK | GeneCards |
| PODXL | GeneCards |
| HSP90AA1 | GeneCards |
| MIR29A | GeneCards |
| MET | GeneCards |
| PIK3C2A | GeneCards |
| DDC | GeneCards |
| KCNJ10 | GeneCards |
| NLRP1 | GeneCards |
| NFE2L2 | GeneCards |
| RET | GeneCards |
| ARMC5 | GeneCards |
| ADIPOR1 | GeneCards |
| NFKBIA | GeneCards |
| VPS33B | GeneCards |
| MIR196A1 | GeneCards |
| FNDC5 | GeneCards |
| CDIPT | GeneCards |
| CD274 | GeneCards |
| PON2 | GeneCards |
| PKLR | GeneCards |
| GPT | GeneCards |
| SLC2A9 | GeneCards |
| GAST | GeneCards |
| FABP4 | GeneCards |
| MIR216A | GeneCards |
| PAPPA | GeneCards |
| ADCYAP1 | GeneCards |
| ARL3 | GeneCards |
| CYBA | GeneCards |
| GAS5 | GeneCards |
| CTNS | GeneCards |
| FOXO1 | GeneCards |
| CDK4 | GeneCards |
| IGF2-AS | GeneCards |
| ESRRA | GeneCards |
| ITGAX | GeneCards |
| MIR93 | GeneCards |
| MIR29C | GeneCards |
| GHRH | GeneCards |
| ETS1 | GeneCards |
| NRAS | GeneCards |
| GUSB | GeneCards |
| CDH4 | GeneCards |
| IGHV4-38-2 | GeneCards |
| NSD1 | GeneCards |
| HLA-DQA2 | GeneCards |
| CPT2 | GeneCards |
| CD69 | GeneCards |
| MIR210 | GeneCards |
| INSM2 | GeneCards |
| MEG8 | GeneCards |
| MIR28 | GeneCards |
| PSMD9 | GeneCards |
| SMAD3 | GeneCards |
| GFPT1 | GeneCards |
| EGFR | GeneCards |
| PTPN11 | GeneCards |
| SMAD2 | GeneCards |
| MAP2K7 | GeneCards |
| TNFRSF1B | GeneCards |
| CLDN16 | GeneCards |
| BMP2 | GeneCards |
| CREBBP | GeneCards |
| PLA2G1B | GeneCards |
| NTF3 | GeneCards |
| ITGA8 | GeneCards |
| UFM1 | GeneCards |
| CDK5RAP3 | GeneCards |
| DDRGK1 | GeneCards |
| UFL1 | GeneCards |
| L1CAM | GeneCards |
| COMT | GeneCards |
| CELA3B | GeneCards |
| SOX17 | GeneCards |
| MIR499A | GeneCards |
| STYX | GeneCards |
| GNRH1 | GeneCards |
| TMEM138 | GeneCards |
| INPP5E | GeneCards |
| RECQL4 | GeneCards |
| MIR200B | GeneCards |
| SP1 | GeneCards |
| LOC108783645 | GeneCards |
| MIR181A1 | GeneCards |
| MYOD1 | GeneCards |
| OAS1 | GeneCards |
| PRKAA2 | GeneCards |
| NR5A1 | GeneCards |
| SLC22A6 | GeneCards |
| CIITA | GeneCards |
| CSN2 | GeneCards |
| GIP | GeneCards |
| MIR15A | GeneCards |
| CNR1 | GeneCards |
| MT-ND3 | GeneCards |
| IPW | GeneCards |
| RARRES2 | GeneCards |
| MIR25 | GeneCards |
| MAPK10 | GeneCards |
| SPG7 | GeneCards |
| PRPF8 | GeneCards |
| ADIPOR2 | GeneCards |
| NUDT10 | GeneCards |
| HLA-DPA1 | GeneCards |
| TSHR | GeneCards |
| ENO1 | GeneCards |
| APLN | GeneCards |
| GNB3 | GeneCards |
| WDR35 | GeneCards |
| SHROOM3 | GeneCards |
| KIAA0586 | GeneCards |
| UBASH3A | GeneCards |
| HAMP | GeneCards |
| ITLN1 | GeneCards |
| KIR3DL1 | GeneCards |
| EDNRA | GeneCards |
| GSTM1 | GeneCards |
| HSPB1 | GeneCards |
| GREM1 | GeneCards |
| SEL1L | GeneCards |
| SLC30A9 | GeneCards |
| GDNF | GeneCards |
| CDH1 | GeneCards |
| HNF4G | GeneCards |
| NRP1 | GeneCards |
| IQCB1 | GeneCards |
| ANGPTL8 | GeneCards |
| GYS1 | GeneCards |
| DARS2 | GeneCards |
| BAMBI | GeneCards |
| TOR1A | GeneCards |
| ZMPSTE24 | GeneCards |
| ACACB | GeneCards |
| NRL | GeneCards |
| PKD1 | GeneCards |
| DDX41 | GeneCards |
| PRSS16 | GeneCards |
| TNFRSF11A | GeneCards |
| PLA2R1 | GeneCards |
| S100A8 | GeneCards |
| ELMO1 | GeneCards |
| GDF15 | GeneCards |
| TNFSF10 | GeneCards |
| CSF1 | GeneCards |
| MICA | GeneCards |
| NPTX2 | GeneCards |
| CD3D | GeneCards |
| TRAF3IP1 | GeneCards |
| PRPS1 | GeneCards |
| MLN | GeneCards |
| SLC22A12 | GeneCards |
| TXNIP | GeneCards |
| MIR214 | GeneCards |
| DYNC2H1 | GeneCards |
| SNHG6 | GeneCards |
| CDH5 | GeneCards |
| MT-ATP8 | GeneCards |
| ADA | GeneCards |
| CYP11B2 | GeneCards |
| CDH23 | GeneCards |
| PTHLH | GeneCards |
| CYP1A1 | GeneCards |
| PAGR1 | GeneCards |
| NOX4 | GeneCards |
| MIR26B | GeneCards |
| ATF4 | GeneCards |
| PLA2G7 | GeneCards |
| BAX | GeneCards |
| SNHG18 | GeneCards |
| APOM | GeneCards |
| PPOX | GeneCards |
| ADAMTS13 | GeneCards |
| MIR148B | GeneCards |
| BAZ1B | GeneCards |
| CEP104 | GeneCards |
| HES1 | GeneCards |
| SLIT2 | GeneCards |
| KLKB1 | GeneCards |
| SERPINA12 | GeneCards |
| OGA | GeneCards |
| TRPV1 | GeneCards |
| NEU1 | GeneCards |
| LIG4 | GeneCards |
| RYR1 | GeneCards |
| PPIG | GeneCards |
| RPGRIP1 | GeneCards |
| TGIF1 | GeneCards |
| ANGPTL4 | GeneCards |
| MIR223 | GeneCards |
| KNG1 | GeneCards |
| MIR34A | GeneCards |
| MIF | GeneCards |
| SREBF1 | GeneCards |
| STAT5A | GeneCards |
| EDNRB | GeneCards |
| ALOX12 | GeneCards |
| DMD | GeneCards |
| FREM2 | GeneCards |
| ACP5 | GeneCards |
| SLC4A4 | GeneCards |
| AHR | GeneCards |
| NDUFV2 | GeneCards |
| MT-TL2 | GeneCards |
| SOD3 | GeneCards |
| CLDN19 | GeneCards |
| PEA15 | GeneCards |
| KL | GeneCards |
| FAM167A | GeneCards |
| MIR9-1 | GeneCards |
| TBL2 | GeneCards |
| JCHAIN | GeneCards |
| BMP4 | GeneCards |
| PRKCA | GeneCards |
| SYVN1 | GeneCards |
| CARTPT | GeneCards |
| TUG1 | GeneCards |
| ALDH2 | GeneCards |
| ALAD | GeneCards |
| MGP | GeneCards |
| GLO1 | GeneCards |
| CPOX | GeneCards |
| EIF2S1 | GeneCards |
| NDUFAF2 | GeneCards |
| CISD1 | GeneCards |
| NDUFS8 | GeneCards |
| NDUFA1 | GeneCards |
| NDUFB3 | GeneCards |
| TMEM126B | GeneCards |
| MT-TI | GeneCards |
| GTF2I | GeneCards |
| CCK | GeneCards |
| MNX1 | GeneCards |
| MT-CYB | GeneCards |
| POMGNT1 | GeneCards |
| PRKCZ | GeneCards |
| LIMK1 | GeneCards |
| SP3 | GeneCards |
| BCL2 | GeneCards |
| GTF2IRD1 | GeneCards |
| CLIP2 | GeneCards |
| MT-TV | GeneCards |
| UBA5 | GeneCards |
| NOTCH1 | GeneCards |
| RTL1 | GeneCards |
| TSPAN8 | GeneCards |
| DHDDS | GeneCards |
| PSMA5 | GeneCards |
| NFKB1 | GeneCards |
| PPARD | GeneCards |
| FGFR1 | GeneCards |
| HMGA2 | GeneCards |
| SOX2 | GeneCards |
| IARS2 | GeneCards |
| BAD | GeneCards |
| RELA | GeneCards |
| FOXA1 | GeneCards |
| SORBS1 | GeneCards |
| SCNN1B | GeneCards |
| CD44 | GeneCards |
| INPPL1 | GeneCards |
| CAMK1D | GeneCards |
| CFAP47 | GeneCards |
| TEK | GeneCards |
| SCNN1A | GeneCards |
| ERBB2 | GeneCards |
| H6PD | GeneCards |
| GAS6 | GeneCards |
| HDC | GeneCards |
| NUP107 | GeneCards |
| CDKN2B-AS1 | GeneCards |
| TTC8 | GeneCards |
| PDGFA | GeneCards |
| GSN | GeneCards |
| TOMM40 | GeneCards |
| APOC1 | GeneCards |
| DYNC2I2 | GeneCards |
| MIR106B | GeneCards |
| SCARB1 | GeneCards |
| VHL | GeneCards |
| SLC9A1 | GeneCards |
| TFR2 | GeneCards |
| CABIN1 | GeneCards |
| SLC17A5 | GeneCards |
| HCRT | GeneCards |
| NKX2-2 | GeneCards |
| POSTN | GeneCards |
| FGFR3 | GeneCards |
| SMURF1 | GeneCards |
| FCAR | GeneCards |
| SIRT3 | GeneCards |
| SMAD4 | GeneCards |
| RHAG | GeneCards |
| APOH | GeneCards |
| PPP1R3B | GeneCards |
| AXIN1 | GeneCards |
| MMP14 | GeneCards |
| CALCA | GeneCards |
| GPX1 | GeneCards |
| EPHB4 | GeneCards |
| PROC | GeneCards |
| JAK1 | GeneCards |
| GIPR | GeneCards |
| TYMP | GeneCards |
| CDKN2B | GeneCards |
| P3H4 | GeneCards |
| HNRNPUL2-BSCL2 | GeneCards |
| FAM20A | GeneCards |
| EIF2AK4 | GeneCards |
| LEMD3 | GeneCards |
| MIA2 | GeneCards |
| COL1A2 | GeneCards |
| RBP1 | GeneCards |
| SLC22A8 | GeneCards |
| SLC9B1 | GeneCards |
| ANXA5 | GeneCards |
| EFEMP1 | GeneCards |
| BCHE | GeneCards |
| CDKN1B | GeneCards |
| RECK | GeneCards |
| BAIAP2L1 | GeneCards |
| BTNL2 | GeneCards |
| MIR20B | GeneCards |
| FRMD3 | GeneCards |
| AFP | GeneCards |
| CGA | GeneCards |
| PRSS1 | GeneCards |
| OCRL | GeneCards |
| MYO7A | GeneCards |
| PAFAH1B1 | GeneCards |
| KCNK16 | GeneCards |
| BCS1L | GeneCards |
| SNORD116@ | GeneCards |
| COL7A1 | GeneCards |
| PRCD | GeneCards |
| NPPC | GeneCards |
| WAS | GeneCards |
| SLC26A4 | GeneCards |
| MMP3 | GeneCards |
| CUBN | GeneCards |
| COL8A2 | GeneCards |
| IL11 | GeneCards |
| PNLIP | GeneCards |
| CISD3 | GeneCards |
| CYB5R4 | GeneCards |
| ENTPD1 | GeneCards |
| ARAP1 | GeneCards |
| CCKAR | GeneCards |
| CYP2D6 | GeneCards |
| MPZ | GeneCards |
| LBR | GeneCards |
| C2CD4A | GeneCards |
| CACNA1H | GeneCards |
| CASP9 | GeneCards |
| GRB10 | GeneCards |
| SCG5 | GeneCards |
| EXO1 | GeneCards |
| C2CD4B | GeneCards |
| CHIT1 | GeneCards |
| IL12B | GeneCards |
| CLCN5 | GeneCards |
| NR5A2 | GeneCards |
| ABCG1 | GeneCards |
| CPT1A | GeneCards |
| DIANPH | GeneCards |
| CSF2 | GeneCards |
| CTSK | GeneCards |
| OLR1 | GeneCards |
| DRD5 | GeneCards |
| SDHB | GeneCards |
| KLF14 | GeneCards |
| MT-TT | GeneCards |
| SHC1 | GeneCards |
| BLOC1S1 | GeneCards |
| MIAT | GeneCards |
| TRIB3 | GeneCards |
| ABCB1 | GeneCards |
| SERPINA1 | GeneCards |
| HTR2A | GeneCards |
| ERCC6 | GeneCards |
| PDSS2 | GeneCards |
| DSTYK | GeneCards |
| USF1 | GeneCards |
| WDR20 | GeneCards |
| ZNHIT3 | GeneCards |
| CYBB | GeneCards |
| CDKN1A | GeneCards |
| SCNN1G | GeneCards |
| MT-TP | GeneCards |
| ALDOB | GeneCards |
| CSF3 | GeneCards |
| MIR149 | GeneCards |
| RHOD | GeneCards |
| PTGS1 | GeneCards |
| MIR32 | GeneCards |
| MT-TN | GeneCards |
| MT-TA | GeneCards |
| NLRP7 | GeneCards |
| CD36 | GeneCards |
| MED13L | GeneCards |
| DRD2 | GeneCards |
| GLIS2 | GeneCards |
| ZFAND3 | GeneCards |
| IDH2 | GeneCards |
| DNMT1 | GeneCards |
| ALAS2 | GeneCards |
| KMT2D | GeneCards |
| EBF3 | GeneCards |
| SLC26A1 | GeneCards |
| VDAC1 | GeneCards |
| IFNB1 | GeneCards |
| DYNC2LI1 | GeneCards |
| CFI | GeneCards |
| CDC42 | GeneCards |
| TUSC3 | GeneCards |
| FOXO3 | GeneCards |
| ARG1 | GeneCards |
| TCF4 | GeneCards |
| TAC1 | GeneCards |
| FASN | GeneCards |
| FOXA3 | GeneCards |
| GCC1 | GeneCards |
| SLC34A1 | GeneCards |
| CXCR4 | GeneCards |
| SYK | GeneCards |
| NAGA | GeneCards |
| F7 | GeneCards |
| EXOSC4 | GeneCards |
| C1GALT1 | GeneCards |
| GJA1 | GeneCards |
| BBS12 | GeneCards |
| ACHE | GeneCards |
| BBS10 | GeneCards |
| NCS1 | GeneCards |
| TMEM126A | GeneCards |
| EIF2AK1 | GeneCards |
| LAMA1 | GeneCards |
| NR2F2 | GeneCards |
| HRH2 | GeneCards |
| HTRA1 | GeneCards |
| FOXP2 | GeneCards |
| MKKS | GeneCards |
| AXL | GeneCards |
| BHLHA15 | GeneCards |
| NKX6-2 | GeneCards |
| PDP1 | GeneCards |
| MIR19A | GeneCards |
| SIRT6 | GeneCards |
| PPP1R15A | GeneCards |
| FABP3 | GeneCards |
| ATXN3 | GeneCards |
| LAMB2 | GeneCards |
| MYC | GeneCards |
| MTR | GeneCards |
| MIR200A | GeneCards |
| CD34 | GeneCards |
| GANAB | GeneCards |
| MB | GeneCards |
| BMP3 | GeneCards |
| SLC2A3 | GeneCards |
| ATXN10 | GeneCards |
| CD19 | GeneCards |
| KCNJ3 | GeneCards |
| LOC108251801 | GeneCards |
| MIR141 | GeneCards |
| CISH | GeneCards |
| PEG3 | GeneCards |
| USB1 | GeneCards |
| ACADS | GeneCards |
| PLUT | GeneCards |
| MCHR1 | GeneCards |
| XYLT2 | GeneCards |
| PROM1 | GeneCards |
| GSTT1 | GeneCards |
| EYA1 | GeneCards |
| MIR122 | GeneCards |
| HSPA8 | GeneCards |
| FMR1 | GeneCards |
| ADA2 | GeneCards |
| CXCL1 | GeneCards |
| NEAT1 | GeneCards |
| MIR26A1 | GeneCards |
| HLA-C | GeneCards |
| IDH1 | GeneCards |
| NGLY1 | GeneCards |
| CFAP410 | GeneCards |
| UROD | GeneCards |
| EPRS1 | GeneCards |
| RECQL5 | GeneCards |
| RNLS | GeneCards |
| GABRR3 | GeneCards |
| KIF12 | GeneCards |
| TBC1D3G | GeneCards |
| LYZ | GeneCards |
| KIF3A | GeneCards |
| FTH1 | GeneCards |
| DDIT3 | GeneCards |
| POLD1 | GeneCards |
| ITGA4 | GeneCards |
| YWHAE | GeneCards |
| RPS27A | GeneCards |
| GNAS-AS1 | GeneCards |
| ARG2 | GeneCards |
| ANPEP | GeneCards |
| MT-RNR1 | GeneCards |
| GPX3 | GeneCards |
| IL12A | GeneCards |
| FAM50B | GeneCards |
| ZDBF2 | GeneCards |
| NLRP5 | GeneCards |
| GBA | GeneCards |
| ADCY10 | GeneCards |
| DUSP1 | GeneCards |
| NEK9 | GeneCards |
| PRSS2 | GeneCards |
| CDK2 | GeneCards |
| NNAT | GeneCards |
| NTRK2 | GeneCards |
| ACSL1 | GeneCards |
| CD1D | GeneCards |
| DBH | GeneCards |
| TBX21 | GeneCards |
| NRIP1 | GeneCards |
| KCNN4 | GeneCards |
| B9D2 | GeneCards |
| WDR73 | GeneCards |
| DYNC2I1 | GeneCards |
| CBS | GeneCards |
| AQP5 | GeneCards |
| DOCK8 | GeneCards |
| SLC39A8 | GeneCards |
| CD63 | GeneCards |
| STUB1 | GeneCards |
| LGALS4 | GeneCards |
| HLA-DRB4 | GeneCards |
| NEK8 | GeneCards |
| APTX | GeneCards |
| U2AF1 | GeneCards |
| PEG10 | GeneCards |
| SIRT4 | GeneCards |
| MVK | GeneCards |
| MAP2K1 | GeneCards |
| WNK1 | GeneCards |
| CEBPA | GeneCards |
| PLA2G2A | GeneCards |
| DDX58 | GeneCards |
| MERTK | GeneCards |
| LDHA | GeneCards |
| IL10RA | GeneCards |
| DUSP2 | GeneCards |
| APEX1 | GeneCards |
| RUNX2 | GeneCards |
| STAT4 | GeneCards |
| TGFBR2 | GeneCards |
| CD55 | GeneCards |
| PRKCSH | GeneCards |
| ECE1 | GeneCards |
| MRE11 | GeneCards |
| RHOA | GeneCards |
| AGRN | GeneCards |
| BECN1 | GeneCards |
| HTT | GeneCards |
| NLRP2 | GeneCards |
| CGAS | GeneCards |
| SLC6A4 | GeneCards |
| RAD51 | GeneCards |
| DNMT3L | GeneCards |
| RASGRF1 | GeneCards |
| KHDC3L | GeneCards |
| PARD6G-AS1 | GeneCards |
| TSC22D1 | GeneCards |
| SIX1 | GeneCards |
| ARL13B | GeneCards |
| B9D1 | GeneCards |
| HPSE | GeneCards |
| XIAP | GeneCards |
| ITGB2 | GeneCards |
| ITGAE | GeneCards |
| NSA2 | GeneCards |
| LECT2 | GeneCards |
| TNNI3 | GeneCards |
| PRKD1 | GeneCards |
| TCTN2 | GeneCards |
| HELLS | GeneCards |
| THSD7A | GeneCards |
| FGFR2 | GeneCards |
| RETNLB | GeneCards |
| LIFR | GeneCards |
| CDK6 | GeneCards |
| HCRTR2 | GeneCards |
| SOX9 | GeneCards |
| MIR423 | GeneCards |
| PYCARD | GeneCards |
| FOXC1 | GeneCards |
| NOS1 | GeneCards |
| CNTLN | GeneCards |
| IFT122 | GeneCards |
| KITLG | GeneCards |
| CYP2C9 | GeneCards |
| TNFRSF18 | GeneCards |
| SRD5A3 | GeneCards |
| SRGAP1 | GeneCards |
| GCM2 | GeneCards |
| YIPF3 | GeneCards |
| GFND1 | GeneCards |
| BBS9 | GeneCards |
| PDE5A | GeneCards |
| FEN1 | GeneCards |
| CDK1 | GeneCards |
| KLRK1 | GeneCards |
| MDM2 | GeneCards |
| TNFSF13B | GeneCards |
| CLCNKA | GeneCards |
| COQ8B | GeneCards |
| GRB2 | GeneCards |
| SLC18A2 | GeneCards |
| GP1BA | GeneCards |
| DDAH2 | GeneCards |
| SFTPD | GeneCards |
| SERPINA7 | GeneCards |
| ITGA2 | GeneCards |
| BCAR1 | GeneCards |
| COQ9 | GeneCards |
| DKK1 | GeneCards |
| AGL | GeneCards |
| TNXB | GeneCards |
| CSF2RA | GeneCards |
| LAMA2 | GeneCards |
| PRKACA | GeneCards |
| PDPK1 | GeneCards |
| MIR31 | GeneCards |
| BTK | GeneCards |
| MCL1 | GeneCards |
| CFP | GeneCards |
| SQSTM1 | GeneCards |
| DNASE1L3 | GeneCards |
| STING1 | GeneCards |
| FECH | GeneCards |
| GSTP1 | GeneCards |
| DIO3 | GeneCards |
| MX1 | GeneCards |
| PMPCA | GeneCards |
| PDYN | GeneCards |
| PRRC2A | GeneCards |
| DGUOK | GeneCards |
| OPN4 | GeneCards |
| SMARCA4 | GeneCards |
| HRH3 | GeneCards |
| IGHE | GeneCards |
| COX4I1 | GeneCards |
| ATN1 | GeneCards |
| MUS81 | GeneCards |
| MIR135A1 | GeneCards |
| SALL1 | GeneCards |
| PTK2B | GeneCards |
| COQ5 | GeneCards |
| CXCR1 | GeneCards |
| CA4 | GeneCards |
| ENG | GeneCards |
| CHI3L1 | GeneCards |
| BDKRB2 | GeneCards |
| TNFRSF10B | GeneCards |
| HOTAIR | GeneCards |
| NAT2 | GeneCards |
| OTC | GeneCards |
| TGFBR1 | GeneCards |
| HLA-G | GeneCards |
| UPK3A | GeneCards |
| MIR378A | GeneCards |
| ADORA2A | GeneCards |
| PKM | GeneCards |
| TOP1 | GeneCards |
| NOTCH2 | GeneCards |
| TERT | GeneCards |
| MIR23B | GeneCards |
| RECQL | GeneCards |
| CD2 | GeneCards |
| CA2 | GeneCards |
| NQO1 | GeneCards |
| ACVRL1 | GeneCards |
| COX10 | GeneCards |
| FLVCR1 | GeneCards |
| CHGA | GeneCards |
| XIST | GeneCards |
| USP25 | GeneCards |
| SH2B1 | GeneCards |
| MIPEP | GeneCards |
| SLC9A3R1 | GeneCards |
| RAB8A | GeneCards |
| IRF5 | GeneCards |
| SAA4 | GeneCards |
| SCARB2 | GeneCards |
| MAT1A | GeneCards |
| CYP24A1 | GeneCards |
| NLRP12 | GeneCards |
| SRY | GeneCards |
| CHUK | GeneCards |
| CMIP | GeneCards |
| VAT1 | GeneCards |
| TCTN1 | GeneCards |
| IL3 | GeneCards |
| IL7 | GeneCards |
| NEK1 | GeneCards |
| DGKE | GeneCards |
| PCAT1 | GeneCards |
| GFRA1 | GeneCards |
| STT3A | GeneCards |
| IFT43 | GeneCards |
| OAT | GeneCards |
| SOST | GeneCards |
| WNT11 | GeneCards |
| PRF1 | GeneCards |
| MNT | GeneCards |
| THY1 | GeneCards |
| TMEM237 | GeneCards |
| BCL2L1 | GeneCards |
| MIR186 | GeneCards |
| AQP1 | GeneCards |
| MCF2L2 | GeneCards |
| C1GALT1C1 | GeneCards |
| KCNJ16 | GeneCards |
| PDGFD | GeneCards |
| CFLAR | GeneCards |
| MIP | GeneCards |
| SLIT3 | GeneCards |
| NCAM1 | GeneCards |
| MT-TC | GeneCards |
| HAX1 | GeneCards |
| CRB2 | GeneCards |
| CCR1 | GeneCards |
| ARSA | GeneCards |
| ZFYVE26 | GeneCards |
| LPP | GeneCards |
| TERF1 | GeneCards |
| PSAP | GeneCards |
| USP7 | GeneCards |
| PGR | GeneCards |
| SIK2 | GeneCards |
| HDAC1 | GeneCards |
| TNKS | GeneCards |
| SH3BP2 | GeneCards |
| SMARCA1 | GeneCards |
| CEP97 | GeneCards |
| MMP7 | GeneCards |
| PKDREJ | GeneCards |
| S100A12 | GeneCards |
| LAP3 | GeneCards |
| TRMU | GeneCards |
| COL1A1 | GeneCards |
| PKD2L1 | GeneCards |
| ACTL7A | GeneCards |
| COL4A6 | GeneCards |
| DNMT3A | GeneCards |
| PARG | GeneCards |
| AKR1B10 | GeneCards |
| KIF17 | GeneCards |
| LRPAP1 | GeneCards |
| PES1 | GeneCards |
| LGALS3 | GeneCards |
| MIR22 | GeneCards |
| PKD1L2 | GeneCards |
| PRNP | GeneCards |
| MIRLET7B | GeneCards |
| SLC17A6 | GeneCards |
| TCTN3 | GeneCards |
| YARS2 | GeneCards |
| LCOR | GeneCards |
| TMEM199 | GeneCards |
| ALK | GeneCards |
| ITGB3 | GeneCards |
| DCN | GeneCards |
| MT-RNR2 | GeneCards |
| TULP3 | GeneCards |
| SLC32A1 | GeneCards |
| ALG9 | GeneCards |
| RAB3IP | GeneCards |
| PIGN | GeneCards |
| ABL1 | GeneCards |
| BABAM1 | GeneCards |
| FUT8 | GeneCards |
| MT-ND4L | GeneCards |
| MMUT | GeneCards |
| CEP164 | GeneCards |
| TNKS2 | GeneCards |
| PDSS1 | GeneCards |
| UIMC1 | GeneCards |
| IFT20 | GeneCards |
| PIFO | GeneCards |
| S100A9 | GeneCards |
| SLC3A1 | GeneCards |
| EZH2 | GeneCards |
| IFT52 | GeneCards |
| DZIP1L | GeneCards |
| PKD1L1 | GeneCards |
| PKD2L2 | GeneCards |
| PKD1L3 | GeneCards |
| CC2D2B | GeneCards |
| RMI2 | GeneCards |
| PARP12 | GeneCards |
| SCO2 | GeneCards |
| PGM3 | GeneCards |
| RAD52 | GeneCards |
| TRPM6 | GeneCards |
| EGR1 | GeneCards |
| CFHR3 | GeneCards |
| CXCL9 | GeneCards |
| ACAD9 | GeneCards |
| ALOX5 | GeneCards |
| MIR382 | GeneCards |
| SLC7A7 | GeneCards |
| IL2RB | GeneCards |
| KIT | GeneCards |
| AIM2 | GeneCards |
| DNAJB11 | GeneCards |
| TNFRSF13B | GeneCards |
| CCND2 | GeneCards |
| MST1 | GeneCards |
| CARS1 | GeneCards |
| GHSR | GeneCards |
| MCU | GeneCards |
| ASS1 | GeneCards |
| VEGFC | GeneCards |
| HDAC9 | GeneCards |
| S100B | GeneCards |
| ITGB1 | GeneCards |
| BDKRB1 | GeneCards |
| CALR | GeneCards |
| SMPD1 | GeneCards |
| DNM2 | GeneCards |
| NEDD4L | GeneCards |
| AQP3 | GeneCards |
| PRKAG2 | GeneCards |
| CYP11B1 | GeneCards |
| THG1L | GeneCards |
| GARS1 | GeneCards |
| SMARCA2 | GeneCards |
| SOX11 | GeneCards |
| LARS2 | GeneCards |
| SLC11A2 | GeneCards |
| UCA1 | GeneCards |
| DVL1 | GeneCards |
| MTMR2 | GeneCards |
| COQ6 | GeneCards |
| WNT9B | GeneCards |
| HOXB7 | GeneCards |
| SBF2 | GeneCards |
| YRDC | GeneCards |
| ARL13A | GeneCards |
| JAG1 | GeneCards |
| KIAA0753 | GeneCards |
| BCL2L11 | GeneCards |
| NOX1 | GeneCards |
| PEX3 | GeneCards |
| MCPH1 | GeneCards |
| MRRF | GeneCards |
| MIRLET7E | GeneCards |
| ESRRB | GeneCards |
| IGFBP4 | GeneCards |
| SEC63 | GeneCards |
| CYP2C19 | GeneCards |
| PLAU | GeneCards |
| SLC22A2 | GeneCards |
| PDGFRB | GeneCards |
| AMPD1 | GeneCards |
| PLAUR | GeneCards |
| AQP11 | GeneCards |
| PKD3 | GeneCards |
| SPATA5L1 | GeneCards |
| CHAT | GeneCards |
| KLF4 | GeneCards |
| SPARC | GeneCards |
| COPA | GeneCards |
| SLC19A3 | GeneCards |
| WNT4 | GeneCards |
| BNIP3 | GeneCards |
| RMI1 | GeneCards |
| DNM1L | GeneCards |
| AKT3 | GeneCards |
| JMJD6 | GeneCards |
| KIAA0319L | GeneCards |
| PKD1P1 | GeneCards |
| BMPR2 | GeneCards |
| FGB | GeneCards |
| NDUFB6 | GeneCards |
| WNK4 | GeneCards |
| CTCF | GeneCards |
| HDAC6 | GeneCards |
| IFNAR1 | GeneCards |
| VEGFB | GeneCards |
| SLC26A9 | GeneCards |
| LOC109623489 | GeneCards |
| PITX2 | GeneCards |
| CLCN1 | GeneCards |
| WNK3 | GeneCards |
| HNRNPH2 | GeneCards |
| ERBB4 | GeneCards |
| MMP8 | GeneCards |
| DDOST | GeneCards |
| FCGR3B | GeneCards |
| SERPINH1 | GeneCards |
| OSM | GeneCards |
| CX3CR1 | GeneCards |
| UBE3A | GeneCards |
| TOP3A | GeneCards |
| TAX1BP3 | GeneCards |
| CTAG1B | GeneCards |
| CCAT1 | GeneCards |
| P2RX5-TAX1BP3 | GeneCards |
| AUH | GeneCards |
| CNDP2 | GeneCards |
| KCNK5 | GeneCards |
| TNNT2 | GeneCards |
| CD163 | GeneCards |
| FABP5 | GeneCards |
| SIX2 | GeneCards |
| PRX | GeneCards |
| SIX5 | GeneCards |
| TFF1 | GeneCards |
| IL1RAPL2 | GeneCards |
| GDAP1 | GeneCards |
| SPRY1 | GeneCards |
| FRAS1 | GeneCards |
| IFT57 | GeneCards |
| FMN1 | GeneCards |
| SH3TC2 | GeneCards |
| C12orf29 | GeneCards |
| CX3CL1 | GeneCards |
| REL | GeneCards |
| OPLAH | GeneCards |
| VAMP2 | GeneCards |
| TFB1M | GeneCards |
| NCOA3 | GeneCards |
| CMA1 | GeneCards |
| LIMK2 | GeneCards |
| GPC3 | GeneCards |
| SMAD7 | GeneCards |
| PALLD | GeneCards |
| NDUFB8 | GeneCards |
| ELAC2 | GeneCards |
| HBEGF | GeneCards |
| VIM | GeneCards |
| ATP6AP2 | GeneCards |
| LAMB1 | GeneCards |
| MFF-DT | GeneCards |
| COQ8A | GeneCards |
| ALPP | GeneCards |
| EIF4EBP1 | GeneCards |
| ARRDC4 | GeneCards |
| ACTL7B | GeneCards |
| FDFT1 | GeneCards |
| MIR335 | GeneCards |
| TXN | GeneCards |
| EPHX2 | GeneCards |
| RBX1 | GeneCards |
| CXCR3 | GeneCards |
| MGAT1 | GeneCards |
| FGF1 | GeneCards |
| STK39 | GeneCards |
| NID1 | GeneCards |
| NOD1 | GeneCards |
| MIR23A | GeneCards |
| MITF | GeneCards |
| MYOM2 | GeneCards |
| UNC119B | GeneCards |
| FCGR2A | GeneCards |
| PARP11 | GeneCards |
| POU5F1 | GeneCards |
| PRSS8 | GeneCards |
| KLHL3 | GeneCards |
| EMX2 | GeneCards |
| SLC4A1 | GeneCards |
| CD226 | GeneCards |
| THPO | GeneCards |
| NTRK1 | GeneCards |
| SORT1 | GeneCards |
| DCX | GeneCards |
| ALPL | GeneCards |
| IRF7 | GeneCards |
| MTERF1 | GeneCards |
| AXIN2 | GeneCards |
| CHD7 | GeneCards |
| MGAT2 | GeneCards |
| ASIC1 | GeneCards |
| RSAD2 | GeneCards |
| DCAF8 | GeneCards |
| TRAT1 | GeneCards |
| LARS1 | GeneCards |
| WHAMM | GeneCards |
| MIR485 | GeneCards |
| SNORD35A | GeneCards |
| NES | GeneCards |
| NAGS | GeneCards |
| GTPBP3 | GeneCards |
| SHPK | GeneCards |
| TMEM70 | GeneCards |
| CDK5RAP1 | GeneCards |
| DRD4 | GeneCards |
| PRKN | GeneCards |
| SNAI1 | GeneCards |
| TAB2 | GeneCards |
| MECOM | GeneCards |
| MTO1 | GeneCards |
| TRHR | GeneCards |
| FBXL4 | GeneCards |
| COA3 | GeneCards |
| HPX | GeneCards |
| TGFB3 | GeneCards |
| PRICKLE1 | GeneCards |
| ADRB1 | GeneCards |
| PCNA | GeneCards |
| PAX3 | GeneCards |
| OPTN | GeneCards |
| LRP1 | GeneCards |
| DUOX2 | GeneCards |
| TUBA1A | GeneCards |
| UPK1A | GeneCards |
| RP1 | GeneCards |
| VNN1 | GeneCards |
| MYD88 | GeneCards |
| IL21 | GeneCards |
| RAG1 | GeneCards |
| ITGA2B | GeneCards |
| SMAD1 | GeneCards |
| DNMT3B | GeneCards |
| LAMP1 | GeneCards |
| BSG | GeneCards |
| CSNK1D | GeneCards |
| CCR2 | GeneCards |
| PIGR | GeneCards |
| CCL4 | GeneCards |
| LAMA5 | GeneCards |
| FOXD1 | GeneCards |
| ERVW-1 | GeneCards |
| PARP15 | GeneCards |
| TRAF3 | GeneCards |
| PLCE1 | GeneCards |
| CD68 | GeneCards |
| FUCA1 | GeneCards |
| KLHDC7A | GeneCards |
| TALDO1 | GeneCards |
| BIRC3 | GeneCards |
| ASL | GeneCards |
| ADAM10 | GeneCards |
| ALOX5AP | GeneCards |
| TMEM147 | GeneCards |
| RB1 | GeneCards |
| SLC26A3 | GeneCards |
| ANKS6 | GeneCards |
| DNAJC13 | GeneCards |
| RAB27A | GeneCards |
| PSMD14 | GeneCards |
| COX15 | GeneCards |
| COX7C | GeneCards |
| ADPRH | GeneCards |
| CASP4 | GeneCards |
| COX6B1 | GeneCards |
| TRIM21 | GeneCards |
| CKAP5 | GeneCards |
| KLRC1 | GeneCards |
| MAGEA3 | GeneCards |
| ELANE | GeneCards |
| PARP9 | GeneCards |
| COL5A1 | GeneCards |
| CTSA | GeneCards |
| PRDX1 | GeneCards |
| PPP1R12A | GeneCards |
| CD14 | GeneCards |
| TTBK2 | GeneCards |
| SNRPA | GeneCards |
| CCP110 | GeneCards |
| ANKS3 | GeneCards |
| FAM186B | GeneCards |
| PRNT | GeneCards |
| PARP3 | GeneCards |
| GJB6 | GeneCards |
| WARS2 | GeneCards |
| SLC25A24 | GeneCards |
| RARS2 | GeneCards |
| TK2 | GeneCards |
| ADI1 | GeneCards |
| COX5B | GeneCards |
| OTOF | GeneCards |
| COX6C | GeneCards |
| MRPL44 | GeneCards |
| P2RX5 | GeneCards |
| FASTKD2 | GeneCards |
| MRPL18 | GeneCards |
| OSGEPL1 | GeneCards |
| ZFYVE21 | GeneCards |
| GPR22 | GeneCards |
| OR4L1 | GeneCards |
| TRMT61B | GeneCards |
| MRM2 | GeneCards |
| SLC66A1 | GeneCards |
| TMEM233 | GeneCards |
| TRL-AAG2-3 | GeneCards |
| TRAP1 | GeneCards |
| SLC27A5 | GeneCards |
| M6PR | GeneCards |
| MIR200C | GeneCards |
| IL23R | GeneCards |
| ZEB1 | GeneCards |
| GRIA3 | GeneCards |
| P2RX7 | GeneCards |
| CSTB | GeneCards |
| FBL | GeneCards |
| SLC6A6 | GeneCards |
| DIABLO | GeneCards |
| PARP2 | GeneCards |
| SMARCB1 | GeneCards |
| MMP12 | GeneCards |
| LSM2 | GeneCards |
| AGPAT1 | GeneCards |
| LAMP2 | GeneCards |
| SLC1A7 | GeneCards |
| FBN2 | GeneCards |
| ABCG5 | GeneCards |
| TJP1 | GeneCards |
| XYLT1 | GeneCards |
| FLT3 | GeneCards |
| RELN | GeneCards |
| PARP8 | GeneCards |
| NUS1 | GeneCards |
| IL22 | GeneCards |
| NFAT5 | GeneCards |
| RAF1 | GeneCards |
| KLK3 | GeneCards |
| CNTNAP2 | GeneCards |
| ABCG2 | GeneCards |
| FOXP1 | GeneCards |
| CD59 | GeneCards |
| MIR199B | GeneCards |
| SLC7A6 | GeneCards |
| CTTN | GeneCards |
| LIF | GeneCards |
| FSHB | GeneCards |
| PARP10 | GeneCards |
| ROBO1 | GeneCards |
| ENSA | GeneCards |
| DGCR5 | GeneCards |
| CAMP | GeneCards |
| ALAS1 | GeneCards |
| HYOU1 | GeneCards |
| SLC3A2 | GeneCards |
| TPRKB | GeneCards |
| ADH1B | GeneCards |
| PROS1 | GeneCards |
| MIR424 | GeneCards |
| TBXAS1 | GeneCards |
| SDC4 | GeneCards |
| TGFBI | GeneCards |
| SERPINB9 | GeneCards |
| MCTP2 | GeneCards |
| NR1H3 | GeneCards |
| TGM1 | GeneCards |
| PSMB5 | GeneCards |
| EPM2A | GeneCards |
| H3-2 | GeneCards |
| COX5A | GeneCards |
| MIR150 | GeneCards |
| ADAMTS9 | GeneCards |
| NCOA2 | GeneCards |
| CES1 | GeneCards |
| HMBS | GeneCards |
| XPO1 | GeneCards |
| CHRNA3 | GeneCards |
| CDK5RAP2 | GeneCards |
| FREM1 | GeneCards |
| CLDN10 | GeneCards |
| MN1 | GeneCards |
| DDB1 | GeneCards |
| SOX18 | GeneCards |
| USP34 | GeneCards |
| ESPN | GeneCards |
| RNF146 | GeneCards |
| PARP16 | GeneCards |
| TTBK1 | GeneCards |
| HYI | GeneCards |
| OARD1 | GeneCards |
| ZCCHC14 | GeneCards |
| RIOX2 | GeneCards |
| ADPRS | GeneCards |
| BHLHA9 | GeneCards |
| C16orf95 | GeneCards |
| RUSF1 | GeneCards |
| TP73-AS1 | GeneCards |
| MIR331 | GeneCards |
| MIR363 | GeneCards |
| PDIA3P1 | GeneCards |
| SULT1A1 | GeneCards |
| CASC2 | GeneCards |
| PPIA | GeneCards |
| COL8A1 | GeneCards |
| ZNF236 | GeneCards |
| P2RX4 | GeneCards |
| LTF | GeneCards |
| NME2 | GeneCards |
| ALG1 | GeneCards |
| PSMA6 | GeneCards |
| CUL3 | GeneCards |
| MSRB3 | GeneCards |
| AIF1 | GeneCards |
| SEMA3A | GeneCards |
| PHEX | GeneCards |
| SERPINB2 | GeneCards |
| SASH1 | GeneCards |
| UNC13B | GeneCards |
| CES2 | GeneCards |
| HSPA1B | GeneCards |
| HAVCR2 | GeneCards |
| TNFSF12 | GeneCards |
| PAX5 | GeneCards |
| C1QTNF3 | GeneCards |
| GLB1 | GeneCards |
| GNA11 | GeneCards |
| TCF3 | GeneCards |
| TBC1D1 | GeneCards |
| NPY1R | GeneCards |
| TRPC1 | GeneCards |
| SRSF1 | GeneCards |
| EZR | GeneCards |
| EVC | GeneCards |
| ZNRF3 | GeneCards |
| SCNN1D | GeneCards |
| SLC7A8 | GeneCards |
| MX2 | GeneCards |
| CPLANE1 | GeneCards |
| F2RL1 | GeneCards |
| CALD1 | GeneCards |
| ISG20 | GeneCards |
| TUBG1 | GeneCards |
| CHD1L | GeneCards |
| LOC111365141 | GeneCards |
| TRAF6 | GeneCards |
| SELENOS | GeneCards |
| ANKRD55 | GeneCards |
| SLC9A3 | GeneCards |
| PROX1 | GeneCards |
| RGMA | GeneCards |
| TREM1 | GeneCards |
| TRIM28 | GeneCards |
| MBP | GeneCards |
| DEFA3 | GeneCards |
| BID | GeneCards |
| NOTCH4 | GeneCards |
| CPB2 | GeneCards |
| LOX | GeneCards |
| ANXA2 | GeneCards |
| AFF3 | GeneCards |
| NLRC4 | GeneCards |
| CXCR6 | GeneCards |
| KMT2C | GeneCards |
| TNFRSF6B | GeneCards |
| IFNGR1 | GeneCards |
| XRCC1 | GeneCards |
| SSTR2 | GeneCards |
| MIR130B | GeneCards |
| FAM135A | GeneCards |
| CXCL5 | GeneCards |
| IRF3 | GeneCards |
| SFTPB | GeneCards |
| DRD3 | GeneCards |
| RPS12 | GeneCards |
| CYP2R1 | GeneCards |
| ASIC5 | GeneCards |
| AGA | GeneCards |
| STIM1 | GeneCards |
| CDH13 | GeneCards |
| PAX8 | GeneCards |
| STS | GeneCards |
| HSPA1L | GeneCards |
| PAX7 | GeneCards |
| TWIST2 | GeneCards |
| CCL20 | GeneCards |
| UGCG | GeneCards |
| SPHK1 | GeneCards |
| ITGA1 | GeneCards |
| ROCK1 | GeneCards |
| IL9 | GeneCards |
| IL33 | GeneCards |
| MAP3K5 | GeneCards |
| NEDD4 | GeneCards |
| HPSE2 | GeneCards |
| MIR497 | GeneCards |
| HAS2 | GeneCards |
| COQ4 | GeneCards |
| GCH1 | GeneCards |
| MIOX | GeneCards |
| DLC1 | GeneCards |
| CD27 | GeneCards |
| RPS3A | GeneCards |
| CCN3 | GeneCards |
| IL4R | GeneCards |
| S100A4 | GeneCards |
| NPNT | GeneCards |
| GJA4 | GeneCards |
| ENO2 | GeneCards |
| TRPV5 | GeneCards |
| A4GALT | GeneCards |
| IRF4 | GeneCards |
| ATOH7 | GeneCards |
| PRICKLE2 | GeneCards |
| NME1-NME2 | GeneCards |
| MIR204 | GeneCards |
| MSR1 | GeneCards |
| ATP6V1B1 | GeneCards |
| SKIL | GeneCards |
| ICOS | GeneCards |
| ABCA3 | GeneCards |
| MAN1B1 | GeneCards |
| TRPV6 | GeneCards |
| DPAGT1 | GeneCards |
| SLC7A9 | GeneCards |
| USP14 | GeneCards |
| COQ7 | GeneCards |
| WASF2 | GeneCards |
| UROS | GeneCards |
| WASF1 | GeneCards |
| GBA2 | GeneCards |
| DNA2 | GeneCards |
| SLC34A3 | GeneCards |
| SPTBN2 | GeneCards |
| TRNT1 | GeneCards |
| CTRL | GeneCards |
| GOSR1 | GeneCards |
| ELP4 | GeneCards |
| SENP8 | GeneCards |
| ACCS | GeneCards |
| ARFRP1 | GeneCards |
| CENPB | GeneCards |
| TSSK1B | GeneCards |
| MYO15A | GeneCards |
| PIF1 | GeneCards |
| KDM8 | GeneCards |
| PLEKHF1 | GeneCards |
| MAGEC1 | GeneCards |
| TECTB | GeneCards |
| CYS1 | GeneCards |
| KATNIP | GeneCards |
| FAM170B | GeneCards |
| MIR135B | GeneCards |
| MIR99B | GeneCards |
| NRIR | GeneCards |
| IGFBP5 | GeneCards |
| PGM1 | GeneCards |
| ATP1A2 | GeneCards |
| ELMO2 | GeneCards |
| IFNL3 | GeneCards |
| JAK3 | GeneCards |
| ITPR1 | GeneCards |
| MCAM | GeneCards |
| MIR455 | GeneCards |
| FCGR3A | GeneCards |
| PUS3 | GeneCards |
| SLC22A1 | GeneCards |
| INSRR | GeneCards |
| C4B | GeneCards |
| PML | GeneCards |
| F10 | GeneCards |
| GP6 | GeneCards |
| CHN2 | GeneCards |
| CCR3 | GeneCards |
| FCRL3 | GeneCards |
| E2F1 | GeneCards |
| IL18BP | GeneCards |
| ANG | GeneCards |
| CTBP1 | GeneCards |
| DNASE1 | GeneCards |
| ATL1 | GeneCards |
| NR1I2 | GeneCards |
| MIRLET7C | GeneCards |
| AGMO | GeneCards |
| AREG | GeneCards |
| TYRO3 | GeneCards |
| GALNT2 | GeneCards |
| SLC15A2 | GeneCards |
| DEFA1 | GeneCards |
| EDN3 | GeneCards |
| KLF6 | GeneCards |
| PTPN13 | GeneCards |
| PHLPP1 | GeneCards |
| RGS1 | GeneCards |
| SDC2 | GeneCards |
| ABCC2 | GeneCards |
| ST6GALNAC2 | GeneCards |
| MMP10 | GeneCards |
| MIR26A2 | GeneCards |
| FBF1 | GeneCards |
| CXCL16 | GeneCards |
| CPVL | GeneCards |
| TNFSF13 | GeneCards |
| KCNMA1 | GeneCards |
| MTHFD1L | GeneCards |
| MTRR | GeneCards |
| PROCR | GeneCards |
| TTC21B-AS1 | GeneCards |
| GRN | GeneCards |
| HSD3B2 | GeneCards |
| LTBP1 | GeneCards |
| UCHL1 | GeneCards |
| NUP133 | GeneCards |
| CASP7 | GeneCards |
| ZEB2 | GeneCards |
| CELA1 | GeneCards |
| TNFRSF8 | GeneCards |
| CD24 | GeneCards |
| ESM1 | GeneCards |
| LYVE1 | GeneCards |
| GSTA1 | GeneCards |
| HDAC4 | GeneCards |
| KIRREL1 | GeneCards |
| CNKSR3 | GeneCards |
| NFATC1 | GeneCards |
| SRSF6 | GeneCards |
| PTPRO | GeneCards |
| UTS2R | GeneCards |
| XRCC2 | GeneCards |
| NDUFS3 | GeneCards |
| RNF10 | GeneCards |
| TWIST1 | GeneCards |
| DEFB1 | GeneCards |
| DCC | GeneCards |
| SCAF4 | GeneCards |
| GABRR1 | GeneCards |
| MASP2 | GeneCards |
| LGALS1 | GeneCards |
| HIPK2 | GeneCards |
| PXN | GeneCards |
| PLA2G4A | GeneCards |
| IL19 | GeneCards |
| ITGAL | GeneCards |
| COL3A1 | GeneCards |
| LRG1 | GeneCards |
| PTER | GeneCards |
| EEF1A1 | GeneCards |
| LIPA | GeneCards |
| MECP2 | GeneCards |
| ACTC1 | GeneCards |
| APCS | GeneCards |
| CYP11A1 | GeneCards |
| TRA | GeneCards |
| MAP2K2 | GeneCards |
| SDK1 | GeneCards |
| ZMIZ1 | GeneCards |
| CYLD | GeneCards |
| SDC3 | GeneCards |
| SOX2-OT | GeneCards |
| CEP120 | GeneCards |
| MFF | GeneCards |
| ADAMTSL1 | GeneCards |
| ADAM17 | GeneCards |
| GLRA3 | GeneCards |
| SLC6A2 | GeneCards |
| C5 | GeneCards |
| NTN1 | GeneCards |
| MIR183 | GeneCards |
| BLZF1 | GeneCards |
| CRY2 | GeneCards |
| TAGLN | GeneCards |
| STX8 | GeneCards |
| TXNRD1 | GeneCards |
| AHSP | GeneCards |
| APLNR | GeneCards |
| CCNB1 | GeneCards |
| MIR196A2 | GeneCards |
| GPR55 | GeneCards |
| SKP1 | GeneCards |
| ICAM3 | GeneCards |
| SCAF8 | GeneCards |
| MDK | GeneCards |
| LHX3 | GeneCards |
| GRAMD2B | GeneCards |
| MIR646HG | GeneCards |
| LOC110283621 | GeneCards |
| SKP2 | GeneCards |
| LINC01512 | GeneCards |
| MIR34B | GeneCards |
| MIR133B | GeneCards |
| ITGA6 | GeneCards |
| APAF1 | GeneCards |
| ANGPTL3 | GeneCards |
| MIR9-2 | GeneCards |
| PREP | GeneCards |
| H3-3B | GeneCards |
| ABCG8 | GeneCards |
| ALCAM | GeneCards |
| ULK1 | GeneCards |
| PCSK1N | GeneCards |
| HAS3 | GeneCards |
| CTSG | GeneCards |
| HMCN1 | GeneCards |
| SMAD5 | GeneCards |
| CASP12 | GeneCards |
| SLC22A3 | GeneCards |
| CYP2J2 | GeneCards |
| NCR3 | GeneCards |
| GFER | GeneCards |
| FCGRT | GeneCards |
| SCD5 | GeneCards |
| TKTL1 | GeneCards |
| IRAK1 | GeneCards |
| TSPAN2 | GeneCards |
| SNCAIP | GeneCards |
| ANGPTL2 | GeneCards |
| SUV39H1 | GeneCards |
| MOK | GeneCards |
| GSTK1 | GeneCards |
| PSRC1 | GeneCards |
| LAMC1 | GeneCards |
| CYP4A11 | GeneCards |
| RNF5 | GeneCards |
| KLF10 | GeneCards |
| ARAP1-AS2 | GeneCards |
| PTK2 | GeneCards |
| ACVR1 | GeneCards |
| MIR193B | GeneCards |
| ILK | GeneCards |
| IFI27 | GeneCards |
| RNF185 | GeneCards |
| ITGB6 | GeneCards |
| NCR2 | GeneCards |
| FN3K | GeneCards |
| CXCR2 | GeneCards |
| CCN1 | GeneCards |
| TYMS | GeneCards |
| MIRLET7A3 | GeneCards |
| BHLHE40 | GeneCards |
| ENHO | GeneCards |
| RASGRP1 | GeneCards |
| ERRFI1 | GeneCards |
| MIRLET7A2 | GeneCards |
| TNC | GeneCards |
| CCDC80 | GeneCards |
| PRPSAP1 | GeneCards |
| MCM10 | GeneCards |
| MIR193A | GeneCards |
| ITIH4 | GeneCards |
| ZNF365 | GeneCards |
| MIR152 | GeneCards |
| EFNB2 | GeneCards |
| ETFB | GeneCards |
| CALB1 | GeneCards |
| MIR9-3 | GeneCards |
| CASQ1 | GeneCards |
| SPINT2 | GeneCards |
| KLK1 | GeneCards |
| OGG1 | GeneCards |
| HSP90B1 | GeneCards |
| MIR215 | GeneCards |
| MIR100 | GeneCards |
| GSK3A | GeneCards |
| ARAP1-AS1 | GeneCards |
| HRH1 | GeneCards |
| ATP1A1 | GeneCards |
| RARB | GeneCards |
| ANXA1 | GeneCards |
| CCL26 | GeneCards |
| ZFP36 | GeneCards |
| FOXP4 | GeneCards |
| RENBP | GeneCards |
| TACR1 | GeneCards |
| LRRC7 | GeneCards |
| GFPT2 | GeneCards |
| ADD2 | GeneCards |
| XRCC3 | GeneCards |
| DES | GeneCards |
| IRX3 | GeneCards |
| TET2 | GeneCards |
| DRD1 | GeneCards |
| NDST1 | GeneCards |
| EHMT2 | GeneCards |
| CLDN1 | GeneCards |
| TMPO | GeneCards |
| SYN2 | GeneCards |
| RARA | GeneCards |
| RFT1 | GeneCards |
| AK3 | GeneCards |
| AK4 | GeneCards |
| EPAS1 | GeneCards |
| ZFP36L1 | GeneCards |
| PNKP | GeneCards |
| SLC2A5 | GeneCards |
| KHK | GeneCards |
| SMAD6 | GeneCards |
| ITGB4 | GeneCards |
| ITGA3 | GeneCards |
| TNFRSF10D | GeneCards |
| PARK7 | GeneCards |
| RPSAP52 | GeneCards |
| LOC110673971 | GeneCards |
| UCN3 | GeneCards |
| CADM1 | GeneCards |
| RBMS1 | GeneCards |
| LINC01191 | GeneCards |
| RBL1 | GeneCards |
| GABRR2 | GeneCards |
| PRNCR1 | GeneCards |
| H2AX | GeneCards |
| TTC39C | GeneCards |
| IL5RA | GeneCards |
| CHD9 | GeneCards |
| ERCC1 | GeneCards |
| MIR137 | GeneCards |
| TCF19 | GeneCards |
| ZFYVE9 | GeneCards |
| FCER2 | GeneCards |
| NPR3 | GeneCards |
| CYP3A5 | GeneCards |
| SAFB | GeneCards |
| NRG3 | GeneCards |
| H19-ICR | GeneCards |
| RICTOR | GeneCards |
| DAG1 | GeneCards |
| MKI67 | GeneCards |
| CDK5 | GeneCards |
| ACTA1 | GeneCards |
| TIMM44 | GeneCards |
| HSPB2 | GeneCards |
| CYP2A6 | GeneCards |
| BTD | GeneCards |
| MIR29B1 | GeneCards |
| GPHA2 | GeneCards |
| PSD3 | GeneCards |
| SEZ6L | GeneCards |
| TSBP1 | GeneCards |
| ODC1 | GeneCards |
| TBK1 | GeneCards |
| VCL | GeneCards |
| CCR7 | GeneCards |
| H3C14 | GeneCards |
| CRHR1 | GeneCards |
| FMN2 | GeneCards |
| UBC | GeneCards |
| ST6GAL1 | GeneCards |
| F12 | GeneCards |
| MAGI2 | GeneCards |
| RXRG | GeneCards |
| BRD4 | GeneCards |
| FAH | GeneCards |
| SRGAP2 | GeneCards |
| CNSN | GeneCards |
| UBE2S | GeneCards |
| LGALSL | GeneCards |
| SLC2A12 | GeneCards |
| IL1RAP | GeneCards |
| PAK1 | GeneCards |
| HCRTR1 | GeneCards |
| S100A1 | GeneCards |
| PEPD | GeneCards |
| ARNT | GeneCards |
| CDK7 | GeneCards |
| CEP41 | GeneCards |
| EVC2 | GeneCards |
| TMX1 | GeneCards |
| TMEM255B | GeneCards |
| PLEKHH2 | GeneCards |
| SERPINA4 | GeneCards |
| ZAP70 | GeneCards |
| MT2A | GeneCards |
| OGN | GeneCards |
| KCNH7 | GeneCards |
| SLC9A3R2 | GeneCards |
| STAC | GeneCards |
| ITGAV | GeneCards |
| EXOC7 | GeneCards |
| SRSF2 | GeneCards |
| CYGB | GeneCards |
| TAF8 | GeneCards |
| CTU1 | GeneCards |
| IRX6 | GeneCards |
| ROCK2 | GeneCards |
| SLC15A1 | GeneCards |
| NCOA1 | GeneCards |
| ELAVL1 | GeneCards |
| LCN1 | GeneCards |
| PLVAP | GeneCards |
| CD209 | GeneCards |
| LOC105378979 | GeneCards |
| IRAK4 | GeneCards |
| F2R | GeneCards |
| HAS1 | GeneCards |
| MAPKBP1 | GeneCards |
| GPR1 | GeneCards |
| LY86-AS1 | GeneCards |
| NDFIP2 | GeneCards |
| DDAH1 | GeneCards |
| ZRANB3 | GeneCards |
| MIR107 | GeneCards |
| CR1 | GeneCards |
| PCSK6 | GeneCards |
| NAV3 | GeneCards |
| SOAT1 | GeneCards |
| GPR158 | GeneCards |
| ANKS1B | GeneCards |
| VASH1 | GeneCards |
| PARL | GeneCards |
| CD46 | GeneCards |
| GIPC1 | GeneCards |
| FMOD | GeneCards |
| SLC2A10 | GeneCards |
| CSNK2B | GeneCards |
| KLF15 | GeneCards |
| NCALD | GeneCards |
| RAP1B | GeneCards |
| SLC37A4 | GeneCards |
| KDM6A | GeneCards |
| CYP2B6 | GeneCards |
| MGAT5B | GeneCards |
| SLC13A3 | GeneCards |
| MIR1207 | GeneCards |
| PGC | GeneCards |
| MR1 | GeneCards |
| GADD45G | GeneCards |
| FCN3 | GeneCards |
| LRP2BP | GeneCards |
| SERPINB13 | GeneCards |
| SETD7 | GeneCards |
| MYL2 | GeneCards |
| VLDLR | GeneCards |
| SOX6 | GeneCards |
| ZNF407 | GeneCards |
| UHRF1 | GeneCards |
| SFTPC | GeneCards |
| MYT1L | GeneCards |
| ACTA2 | GeneCards |
| AGTRAP | GeneCards |
| MIR194-1 | GeneCards |
| ACAD11 | GeneCards |
| MEP1B | GeneCards |
| ADNP | GeneCards |
| GCLM | GeneCards |
| TRPA1 | GeneCards |
| SPON2 | GeneCards |
| RGN | GeneCards |
| NFATC3 | GeneCards |
| TBC1D5 | GeneCards |
| RAD51B | GeneCards |
| DCAF5 | GeneCards |
| RPL26P31 | GeneCards |
| ENSG00000227489 | GeneCards |
| piR-39858-250 | GeneCards |
| lnc-RPGRIP1L-2 | GeneCards |
| PTAFR | GeneCards |
| MIA3 | GeneCards |
| AASS | GeneCards |
| MAEA | GeneCards |
| TBXT | GeneCards |
| MIR103A1 | GeneCards |
| KIRREL2 | GeneCards |
| TRIM25 | GeneCards |
| SORCS3 | GeneCards |
| ITGA5 | GeneCards |
| TNFRSF10C | GeneCards |
| FIS1 | GeneCards |
| SNORA33 | GeneCards |
| WNT1 | GeneCards |
| RNASE3 | GeneCards |
| HLTF | GeneCards |
| IL1RL1 | GeneCards |
| ACKR1 | GeneCards |
| CD247 | GeneCards |
| GPANK1 | GeneCards |
| IMMP2L | GeneCards |
| A2M | GeneCards |
| GPRC5B | GeneCards |
| AZGP1 | GeneCards |
| CPT1C | GeneCards |
| LY75 | GeneCards |
| ORAI1 | GeneCards |
| NEU2 | GeneCards |
| CCNL1 | GeneCards |
| ERVFRD-1 | GeneCards |
| TENM2 | GeneCards |
| STAB1 | GeneCards |
| MTMR3 | GeneCards |
| WDR62 | GeneCards |
| CYP1B1 | GeneCards |
| BGN | GeneCards |
| ELK1 | GeneCards |
| GRIA1 | GeneCards |
| GPX4 | GeneCards |
| NPR1 | GeneCards |
| PTPN6 | GeneCards |
| NEFL | GeneCards |
| SLC26A8 | GeneCards |
| RYR3 | GeneCards |
| ALPI | GeneCards |
| ADORA2B | GeneCards |
| SAA2 | GeneCards |
| MIR135A2 | GeneCards |
| TFF3 | GeneCards |
| NDST2 | GeneCards |
| MIR4490 | GeneCards |
| ESRRG | GeneCards |
| LRP8 | GeneCards |
| IRX5 | GeneCards |
| RAPGEF5 | GeneCards |
| BTBD11 | GeneCards |
| STEAP1B | GeneCards |
| BMPR1A | GeneCards |
| CEP89 | GeneCards |
| PDLIM5 | GeneCards |
| CALCR | GeneCards |
| F2RL3 | GeneCards |
| MMADHC | GeneCards |
| FLNB | GeneCards |
| PDGFC | GeneCards |
| CALB2 | GeneCards |
| MUC7 | GeneCards |
| ACKR2 | GeneCards |
| CCL25 | GeneCards |
| DMRT1 | GeneCards |
| SOSTDC1 | GeneCards |
| MIR103A2 | GeneCards |
| RTN1 | GeneCards |
| KCNK1 | GeneCards |
| ASCL2 | GeneCards |
| SUCNR1 | GeneCards |
| NUP93 | GeneCards |
| TIPIN | GeneCards |
| ASTN1 | GeneCards |
| IQGAP1 | GeneCards |
| LAMA4 | GeneCards |
| TNFAIP8 | GeneCards |
| STK19 | GeneCards |
| DXO | GeneCards |
| TRB | GeneCards |
| BBOX1 | GeneCards |
| HNRNPF | GeneCards |
| RLN2 | GeneCards |
| RELB | GeneCards |
| TRPM5 | GeneCards |
| LEKR1 | GeneCards |
| UBE3C | GeneCards |
| NOX5 | GeneCards |
| COLEC11 | GeneCards |
| DIAPH3 | GeneCards |
| MORC2 | GeneCards |
| PFDN4 | GeneCards |
| BCAS1 | GeneCards |
| DCLK3 | GeneCards |
| PIK3IP1 | GeneCards |
| FSTL5 | GeneCards |
| ALLC | GeneCards |
| SFI1 | GeneCards |
| SPPL3 | GeneCards |
| EXD2 | GeneCards |
| AMTN | GeneCards |
| KRTAP3-2 | GeneCards |
| KRTAP3-3 | GeneCards |
| SUSD6 | GeneCards |
| HAND2-AS1 | GeneCards |
| CASC19 | GeneCards |
| LINC01266 | GeneCards |
| MGC32805 | GeneCards |
| INSYN1-AS1 | GeneCards |
| RNU6ATAC2P | GeneCards |
| LINC02511 | GeneCards |
| RNU6-921P | GeneCards |
| ENSG00000258623 | GeneCards |
| ENSG00000266602 | GeneCards |
| GSTM5P1 | GeneCards |
| HMGN1P17 | GeneCards |
| RNU7-88P | GeneCards |
| ENSG00000224239 | GeneCards |
| TOMM22P3 | GeneCards |
| ENSG00000257407 | GeneCards |
| ENSG00000249697 | GeneCards |
| ENSG00000260624 | GeneCards |
| ENSG00000235158 | GeneCards |
| ENSG00000259038 | GeneCards |
| RN7SL865P | GeneCards |
| GLRX3P1 | GeneCards |
| ENSG00000285731 | GeneCards |
| lnc-CYP24A1-3 | GeneCards |
| RF00066-055 | GeneCards |
| lnc-ZFP36L1-7 | GeneCards |
| HSALNG0130972 | GeneCards |
| piR-57461-063 | GeneCards |
| HSALNG0041938 | GeneCards |
| HSALNG0025132 | GeneCards |
| HSALNG0025135 | GeneCards |
| LOC107984625 | GeneCards |
| LOC105378977 | GeneCards |
| ENSG00000237434 | GeneCards |
| YAP1 | GeneCards |
| IRF8 | GeneCards |
| MST1R | GeneCards |
| GCLC | GeneCards |
| SPI1 | GeneCards |
| TNFSF4 | GeneCards |
| FBLN1 | GeneCards |
| VPS4B | GeneCards |
| MBD2 | GeneCards |
| ZADH2 | GeneCards |
| CDH20 | GeneCards |
| ALPK2 | GeneCards |
| AOX1 | GeneCards |
| NCOR1 | GeneCards |
| STAT6 | GeneCards |
| RAMP2 | GeneCards |
| ALS2 | GeneCards |
| IPPK | GeneCards |
| SUV39H2 | GeneCards |
| IGES | GeneCards |
| LINC01619 | GeneCards |
| THBS4 | GeneCards |
| TRAF1 | GeneCards |
| UBE2V1 | GeneCards |
| RPTOR | GeneCards |
| DDR1 | GeneCards |
| GP2 | GeneCards |
| CD74 | GeneCards |
| UBE4A | GeneCards |
| TRIM56 | GeneCards |
| STIM2 | GeneCards |
| CCL21 | GeneCards |
| PLSCR3 | GeneCards |
| PPP3CA | GeneCards |
| EGFL8 | GeneCards |
| IKZF1 | GeneCards |
| STMN1 | GeneCards |
| ATF3 | GeneCards |
| CR2 | GeneCards |
| MTHFS | GeneCards |
| GPRC5A | GeneCards |
| DHFR | GeneCards |
| NCF2 | GeneCards |
| SDC1 | GeneCards |
| MED25 | GeneCards |
| RARS1 | GeneCards |
| WTAP | GeneCards |
| TNFRSF9 | GeneCards |
| RREB1 | GeneCards |
| CRY1 | GeneCards |
| WT1-AS | GeneCards |
| CCL28 | GeneCards |
| RBFOX1 | GeneCards |
| TSHZ2 | GeneCards |
| MTSS1 | GeneCards |
| CERT1 | GeneCards |
| CLEC4A | GeneCards |
| IGSF21 | GeneCards |
| CD81 | GeneCards |
| CD47 | GeneCards |
| FAT1 | GeneCards |
| NT5E | GeneCards |
| SMN1 | GeneCards |
| ATP6V0A2 | GeneCards |
| CTCFL | GeneCards |
| LCP1 | GeneCards |
| AK1 | GeneCards |
| ACTR2 | GeneCards |
| DPEP1 | GeneCards |
| PFKFB2 | GeneCards |
| SLCO1A2 | GeneCards |
| CTSC | GeneCards |
| DACH1 | GeneCards |
| LALBA | GeneCards |
| DCDC2 | GeneCards |
| MT-TR | GeneCards |
| PTPRU | GeneCards |
| CAPN6 | GeneCards |
| RCAN1 | GeneCards |
| FDX1 | GeneCards |
| PODN | GeneCards |
| HECW1 | GeneCards |
| VAV3 | GeneCards |
| CCT3 | GeneCards |
| NUP62 | GeneCards |
| TRIAP1 | GeneCards |
| CCND3 | GeneCards |
| MATN2 | GeneCards |
| CARD9 | GeneCards |
| NOX3 | GeneCards |
| MIR320A | GeneCards |
| BRD2 | GeneCards |
| KCTD7 | GeneCards |
| NUMB | GeneCards |
| MAD2L2 | GeneCards |
| PICK1 | GeneCards |
| DERL2 | GeneCards |
| CD22 | GeneCards |
| GORAB | GeneCards |
| SYT1 | GeneCards |
| TNFRSF19 | GeneCards |
| RPL37A | GeneCards |
| ZNF175 | GeneCards |
| SHANK1 | GeneCards |
| SAP30BP | GeneCards |
| GUCY1A1 | GeneCards |
| TRIM65 | GeneCards |
| SEPTIN9 | GeneCards |
| TRIM47 | GeneCards |
| MYZAP | GeneCards |
| GCOM1 | GeneCards |
| C19orf81 | GeneCards |
| ADM5 | GeneCards |
| KRT18P34 | GeneCards |
| MIR133A1 | GeneCards |
| HSD17B10 | GeneCards |
| CASP5 | GeneCards |
| DYNC1H1 | GeneCards |
| MBTPS2 | GeneCards |
| NDUFB4 | GeneCards |
| CDCA7 | GeneCards |
| PTH1R | GeneCards |
| KLRA1P | GeneCards |
| SETD2 | GeneCards |
| POLR2I | GeneCards |
| SLC41A1 | GeneCards |
| NOSIP | GeneCards |
| CTIF | GeneCards |
| MPND | GeneCards |
| ANXA11 | GeneCards |
| UNC13D | GeneCards |
| WWC1 | GeneCards |
| FUT9 | GeneCards |
| KERA | GeneCards |
| REV1 | GeneCards |
| SUCLG2 | GeneCards |
| CDK3 | GeneCards |
| SETMAR | GeneCards |
| REPS1 | GeneCards |
| LSAMP | GeneCards |
| GATC | GeneCards |
| MAGOH | GeneCards |
| CRCP | GeneCards |
| CGNL1 | GeneCards |
| SRP68 | GeneCards |
| SYT3 | GeneCards |
| KDM4D | GeneCards |
| ZHX2 | GeneCards |
| ZNF473 | GeneCards |
| MED20 | GeneCards |
| EPYC | GeneCards |
| PTOV1 | GeneCards |
| TAPT1 | GeneCards |
| UST | GeneCards |
| CABYR | GeneCards |
| METTL23 | GeneCards |
| MFSD11 | GeneCards |
| DYDC1 | GeneCards |
| TPRG1 | GeneCards |
| TSPAN14 | GeneCards |
| TSGA10 | GeneCards |
| UNK | GeneCards |
| POP5 | GeneCards |
| CABP1 | GeneCards |
| SLC35A5 | GeneCards |
| ANKRD29 | GeneCards |
| ABRACL | GeneCards |
| C19orf48 | GeneCards |
| DYDC2 | GeneCards |
| SPINK4 | GeneCards |
| MITD1 | GeneCards |
| CFAP44 | GeneCards |
| TBC1D31 | GeneCards |
| LYG1 | GeneCards |
| IGSF22 | GeneCards |
| PLAC9 | GeneCards |
| CD200R1L | GeneCards |
| NEPRO | GeneCards |
| CZIB | GeneCards |
| PRXL2A | GeneCards |
| SCGB2B2 | GeneCards |
| SMIM13 | GeneCards |
| FBLL1 | GeneCards |
| PLEKHD1 | GeneCards |
| TRABD2B | GeneCards |
| EFCAB8 | GeneCards |
| SNHG16 | GeneCards |
| LINC00857 | GeneCards |
| FOXP4-AS1 | GeneCards |
| CASC9 | GeneCards |
| NUTM2B-AS1 | GeneCards |
| B4GALT1-AS1 | GeneCards |
| LINC00484 | GeneCards |
| MBL1P | GeneCards |
| SCN1A-AS1 | GeneCards |
| PTOV1-AS2 | GeneCards |
| TPRG1-AS1 | GeneCards |
| TBC1D27P | GeneCards |
| LINC01003 | GeneCards |
| LINC01249 | GeneCards |
| LINC01276 | GeneCards |
| LINC01151 | GeneCards |
| LINC02607 | GeneCards |
| LOC729296 | GeneCards |
| LINC01771 | GeneCards |
| LINC02237 | GeneCards |
| RNU6-844P | GeneCards |
| RPL31P52 | GeneCards |
| EIF5AP4 | GeneCards |
| ENSG00000111780 | GeneCards |
| ENSG00000233290 | GeneCards |
| ENSG00000235192 | GeneCards |
| ENSG00000256915 | GeneCards |
| ENSG00000249319 | GeneCards |
| LOC100996664 | GeneCards |
| RNU6-41P | GeneCards |
| ENSG00000235858 | GeneCards |
| ENSG00000266980 | GeneCards |
| ENSG00000267543 | GeneCards |
| ENSG00000267546 | GeneCards |
| ENSG00000267808 | GeneCards |
| ENSG00000268047 | GeneCards |
| LINC02720 | GeneCards |
| RNA5SP250 | GeneCards |
| LOC100130698 | GeneCards |
| RNU6-1128P | GeneCards |
| ENSG00000231113 | GeneCards |
| ENSG00000232762 | GeneCards |
| ENSG00000232234 | GeneCards |
| ENSG00000212590 | GeneCards |
| ENSG00000256569 | GeneCards |
| ENSG00000250602 | GeneCards |
| ENSG00000248973 | GeneCards |
| ENSG00000253369 | GeneCards |
| ENSG00000285216 | GeneCards |
| RNA5SP86 | GeneCards |
| RN7SKP82 | GeneCards |
| RNU6-34P | GeneCards |
| RPL31P29 | GeneCards |
| ENSG00000124593 | GeneCards |
| SLC7A15P | GeneCards |
| SMC3P1 | GeneCards |
| ENSG00000234578 | GeneCards |
| ENSG00000234185 | GeneCards |
| ENSG00000230537 | GeneCards |
| ENSG00000258631 | GeneCards |
| ENSG00000258703 | GeneCards |
| ENSG00000251517 | GeneCards |
| ENSG00000254966 | GeneCards |
| ENSG00000257781 | GeneCards |
| HMGB1P13 | GeneCards |
| ENSG00000253880 | GeneCards |
| LINC01738 | GeneCards |
| ENSG00000264924 | GeneCards |
| ENSG00000265204 | GeneCards |
| ENSG00000267078 | GeneCards |
| ENSG00000267342 | GeneCards |
| ENSG00000259026 | GeneCards |
| ENSG00000259283 | GeneCards |
| ENSG00000275329 | GeneCards |
| ARL6IP1P3 | GeneCards |
| ENSG00000288034 | GeneCards |
| ENSG00000287358 | GeneCards |
| ENSG00000286280 | GeneCards |
| RN7SL72P | GeneCards |
| RNU6-1200P | GeneCards |
| RNU6-1059P | GeneCards |
| HMGB1P50 | GeneCards |
| ENSG00000231863 | GeneCards |
| ENSG00000258225 | GeneCards |
| ENSG00000272386 | GeneCards |
| ENSG00000273306 | GeneCards |
| ENSG00000259133 | GeneCards |
| lnc-DERL1-5 | GeneCards |
| RF00066-125 | GeneCards |
| RF00017-6767 | GeneCards |
| RF00017-5268 | GeneCards |
| RF00017-5079 | GeneCards |
| FJ601684-299 | GeneCards |
| RF00017-3806 | GeneCards |
| RF00017-3811 | GeneCards |
| lnc-FOXP4-2 | GeneCards |
| lnc-SHANK1-2 | GeneCards |
| RPA2P2 | GeneCards |
| RNU4-37P | GeneCards |
| NPM1P48 | GeneCards |
| ENSG00000234382 | GeneCards |
| lnc-LPP-2 | GeneCards |
| lnc-PRCD-5 | GeneCards |
| tRX-Lys-NNN-6-1 | GeneCards |
| ENSG00000242308 | GeneCards |
| MN297907-011 | GeneCards |
| NONHSAG023555.2 | GeneCards |
| ENSG00000273381 | GeneCards |
| ENSG00000265853 | GeneCards |
| lnc-ABRACL-1 | GeneCards |
| lnc-TCF12-2 | GeneCards |
| RF00017-5082 | GeneCards |
| FJ601684-412 | GeneCards |
| ENSG00000285775 | GeneCards |
| piR-39701-037 | GeneCards |
| RF00017-3278 | GeneCards |
| RF00026-362 | GeneCards |
| MN298114-045 | GeneCards |
| RF00994-964 | GeneCards |
| piR-57461-057 | GeneCards |
| MIR4456-001 | GeneCards |
| MN309609 | GeneCards |
| MN298066 | GeneCards |
| HSALNG0111626 | GeneCards |
| ENSG00000225416 | GeneCards |
| HSALNG0092595 | GeneCards |
| hsa-miR-5095-097 | GeneCards |
| piR-38959-011 | GeneCards |
| piR-31199-129 | GeneCards |
| lnc-CLEC2L-4 | GeneCards |
| MG828736 | GeneCards |
| MG828737 | GeneCards |
| RF00017-6688 | GeneCards |
| ENSG00000283555 | GeneCards |
| piR-39476-002 | GeneCards |
| lnc-GAP43-16 | GeneCards |
| lnc-SHANK1-3 | GeneCards |
| RF00951-072 | GeneCards |
| RF01045-144 | GeneCards |
| ENSG00000205625 | GeneCards |
| piR-30175-020 | GeneCards |
| LOC107986400 | GeneCards |
| ENSG00000279359 | GeneCards |
| ENSG00000274133 | GeneCards |
| piR-32532-002 | GeneCards |
| piR-51705-002 | GeneCards |
| piR-50948-002 | GeneCards |
| piR-34665-002 | GeneCards |
| piR-42730 | GeneCards |
| piR-34000-002 | GeneCards |
| piR-41669 | GeneCards |
| ENSG00000231139 | GeneCards |
| piR-53819-002 | GeneCards |
| piR-61185-002 | GeneCards |
| piR-30060-002 | GeneCards |
| LOC105378728 | GeneCards |
| ENSG00000241385 | GeneCards |
| piR-47386-002 | GeneCards |
| LILRB3 | GeneCards |
| MUC16 | GeneCards |
| ACSM3 | GeneCards |
| ARRB2 | GeneCards |
| NAP1L4 | GeneCards |
| MRPL23 | GeneCards |
| POFUT2 | GeneCards |
| LDB1 | GeneCards |
| HIF3A | GeneCards |
| S100A6 | GeneCards |
| PRMT1 | GeneCards |
| GOLIM4 | GeneCards |
| AP2B1 | GeneCards |
| ACAA1 | GeneCards |
| SCP2 | GeneCards |
| MSN | GeneCards |
| GCM1 | GeneCards |
| TRIM68 | GeneCards |
| ZNF7 | GeneCards |
| COMMD5 | GeneCards |
| OR52I1 | GeneCards |
| RAMP3 | GeneCards |
| CHRNA1 | GeneCards |
| VEPH1 | GeneCards |
| CLEC12A | GeneCards |
| SLC7A5 | GeneCards |
| SLC12A5 | GeneCards |
| FCN1 | GeneCards |
| SLC22A18 | GeneCards |
| MRPL19 | GeneCards |
| SPNS1 | GeneCards |
| DNAJC14 | GeneCards |
| TSSC4 | GeneCards |
| KCNQ1-AS1 | GeneCards |
| XCL1 | GeneCards |
| NAP1L5 | GeneCards |
| NRP2 | GeneCards |
| CRBN | GeneCards |
| HNRNPC | GeneCards |
| CARD8 | GeneCards |
| ARF6 | GeneCards |
| LAMP3 | GeneCards |
| RRAGB | GeneCards |
| CCL23 | GeneCards |
| CYP20A1 | GeneCards |
| FOSL1 | GeneCards |
| MIR490 | GeneCards |
| FCGR1A | GeneCards |
| PRCP | GeneCards |
| GNG2 | GeneCards |
| MPRIP | GeneCards |
| MAP1LC3A | GeneCards |
| DGKQ | GeneCards |
| BIRC2 | GeneCards |
| MTM1 | GeneCards |
| PREX1 | GeneCards |
| FYN | GeneCards |
| GNE | GeneCards |
| SLAMF1 | GeneCards |
| EIF2A | GeneCards |
| HRG | GeneCards |
| SLC5A6 | GeneCards |
| BCL6 | GeneCards |
| CBL | GeneCards |
| ADH7 | GeneCards |
| RAB38 | GeneCards |
| NFKB2 | GeneCards |
| TIMM8A | GeneCards |
| SLC7A2 | GeneCards |
| GPER1 | GeneCards |
| GREM2 | GeneCards |
| TCP1 | GeneCards |
| STX11 | GeneCards |
| CREB5 | GeneCards |
| CCDC68 | GeneCards |
| MAT2A | GeneCards |
| VAV1 | GeneCards |
| PLOD2 | GeneCards |
| ATP6AP1 | GeneCards |
| CD5 | GeneCards |
| DUSP5 | GeneCards |
| BCL2L12 | GeneCards |
| B4GALT1 | GeneCards |
| MIR374A | GeneCards |
| PTGIR | GeneCards |
| C1QA | GeneCards |
| DDN | GeneCards |
| TNFSF12-TNFSF13 | GeneCards |
| FES | GeneCards |
| CD79B | GeneCards |
| WEE1 | GeneCards |
| TUBA8 | GeneCards |
| TXK | GeneCards |
| TEC | GeneCards |
| CA9 | GeneCards |
| BCL2L2 | GeneCards |
| PSMB10 | GeneCards |
| COL5A2 | GeneCards |
| SMG6 | GeneCards |
| KLHL2 | GeneCards |
| POU2AF1 | GeneCards |
| SLC35C2 | GeneCards |
| IMMP1L | GeneCards |
| IGHM | GeneCards |
| NAIP | GeneCards |
| LTC4S | GeneCards |
| KAT5 | GeneCards |
| INPP5D | GeneCards |
| S1PR1 | GeneCards |
| MEOX2 | GeneCards |
| LIN7C | GeneCards |
| CA3 | GeneCards |
| DPM1 | GeneCards |
| SLC26A5 | GeneCards |
| IKZF3 | GeneCards |
| OSBPL5 | GeneCards |
| RAI1 | GeneCards |
| ALG12 | GeneCards |
| COG7 | GeneCards |
| PELP1 | GeneCards |
| DPH2 | GeneCards |
| IL36G | GeneCards |
| PHLDA2 | GeneCards |
| GINS2 | GeneCards |
| SSR3 | GeneCards |
| TSPAN32 | GeneCards |
| ASB7 | GeneCards |
| COMMD9 | GeneCards |
| C5orf15 | GeneCards |
| TYW5 | GeneCards |
| TM2D3 | GeneCards |
| DCDC1 | GeneCards |
| SLC22A18AS | GeneCards |
| C11orf21 | GeneCards |
| EIPR1 | GeneCards |
| WASH3P | GeneCards |
| CCL8 | GeneCards |
| HORMAD2-AS1 | GeneCards |
| EXT1 | GeneCards |
| TXN2 | GeneCards |
| TNR | GeneCards |
| CAP1 | GeneCards |
| FOXN2 | GeneCards |
| CALCRL | GeneCards |
| MAP3K14 | GeneCards |
| TNFRSF12A | GeneCards |
| TNS1 | GeneCards |
| ANXA7 | GeneCards |
| GAL3ST1 | GeneCards |
| TSPYL2 | GeneCards |
| VSTM2B | GeneCards |
| SEMA3G | GeneCards |
| MRPS15 | GeneCards |
| OSCP1 | GeneCards |
| MAP3K21 | GeneCards |
| LINC00917 | GeneCards |
| LINC02774 | GeneCards |
| LOC100506023 | GeneCards |
| LOC729200 | GeneCards |
| LOC101928236 | GeneCards |
| LOC100131080 | GeneCards |
| KCNE1 | GeneCards |
| H2BS1 | GeneCards |
| DUP2Q31.1 | GeneCards |
| PLD1 | GeneCards |
| RNPC3 | GeneCards |
| NSD2 | GeneCards |
| MAX | GeneCards |
| PDLIM1 | GeneCards |
| ATP5F1B | GeneCards |
| ALG14 | GeneCards |
| CDH22 | GeneCards |
| PMAIP1 | GeneCards |
| TIA1 | GeneCards |
| MIR744 | GeneCards |
| ARHGAP45 | GeneCards |
| PPT2-EGFL8 | GeneCards |
| MYCT1 | GeneCards |
| KIF6 | GeneCards |
| PGA3 | GeneCards |
| PGA5 | GeneCards |
| PGA4 | GeneCards |
| SLC9C1 | GeneCards |
| EGLN1 | GeneCards |
| COG6 | GeneCards |
| EIF5B | GeneCards |
| SH2D4B | GeneCards |
| CRYM | GeneCards |
| ARX | GeneCards |
| KPNA1 | GeneCards |
| APOL3 | GeneCards |
| RNR1 | GeneCards |
| VTCN1 | GeneCards |
| HIF1AN | GeneCards |
| HOXD13 | GeneCards |
| KDM4A | GeneCards |
| COG4 | GeneCards |
| ADGRG1 | GeneCards |
| VSTM4 | GeneCards |
| MIR379 | GeneCards |
| ST2 | GeneCards |
| LTBR | GeneCards |
| ACTG2 | GeneCards |
| CHPT1 | GeneCards |
| CRABP2 | GeneCards |
| FBXW7 | GeneCards |
| EIF5A | GeneCards |
| SLC6A19 | GeneCards |
| SIAH1 | GeneCards |
| SPTA1 | GeneCards |
| TAT | GeneCards |
| SESN2 | GeneCards |
| UBA52 | GeneCards |
| POMGNT2 | GeneCards |
| C5AR1 | GeneCards |
| CXCR5 | GeneCards |
| TFPT | GeneCards |
| MMP16 | GeneCards |
| SLC5A5 | GeneCards |
| NUDC | GeneCards |
| CRLS1 | GeneCards |
| RIPK1 | GeneCards |
| FIBP | GeneCards |
| MFAP3 | GeneCards |
| RIPK2 | GeneCards |
| EGLN2 | GeneCards |
| MYO1C | GeneCards |
| GOLGB1 | GeneCards |
| TRARG1 | GeneCards |
| ORAI3 | GeneCards |
| CCNE1 | GeneCards |
| TNFSF14 | GeneCards |
| MMP24 | GeneCards |
| EGLN3 | GeneCards |
| CACNA1I | GeneCards |
| ATP6V0A4 | GeneCards |
| TNFRSF17 | GeneCards |
| CD33 | GeneCards |
| IGLL1 | GeneCards |
| SPTBN1 | GeneCards |
| SGCE | GeneCards |
| UCHL5 | GeneCards |
| WWP1 | GeneCards |
| FAM20C | GeneCards |
| ALG2 | GeneCards |
| CKS1B | GeneCards |
| NEDD8 | GeneCards |
| PAFAH1B3 | GeneCards |
| MOGS | GeneCards |
| DEGS1 | GeneCards |
| SLC35C1 | GeneCards |
| TRIP10 | GeneCards |
| KDELR1 | GeneCards |
| APBA3 | GeneCards |
| ADAMTSL5 | GeneCards |
| SPCS3 | GeneCards |
| SNRPD3 | GeneCards |
| ENOSF1 | GeneCards |
| PRB1 | GeneCards |
| PRH1 | GeneCards |
| ELOB | GeneCards |
| PRH2 | GeneCards |
| WASF5P | GeneCards |
| LOC107988023 | GeneCards |
| GCA | GeneCards |
| POLI | GeneCards |
| SMPD2 | GeneCards |
| CLCN2 | GeneCards |
| TPSAB1 | GeneCards |
| SCN7A | GeneCards |
| SH2D4A | GeneCards |
| STARD5 | GeneCards |
| MIR544A | GeneCards |
| TAS2R43 | GeneCards |
| MFAP4 | GeneCards |
| HNRNPA2B1 | GeneCards |
| PCM1 | GeneCards |
| KRIT1 | GeneCards |
| DERL1 | GeneCards |
| LPAR1 | GeneCards |
| ZYX | GeneCards |
| SMPDL3B | GeneCards |
| GRAP | GeneCards |
| SMPD3 | GeneCards |
| MIR302A | GeneCards |
| RND3 | GeneCards |
| ADK | GeneCards |
| LTA4H | GeneCards |
| ASGR2 | GeneCards |
| FHL2 | GeneCards |
| SLC44A3 | GeneCards |
| BARX2 | GeneCards |
| GALNT18 | GeneCards |
| TMEM45B | GeneCards |
| RBM43 | GeneCards |
| APELA | GeneCards |
| MBL3P | GeneCards |
| HLA-DPB2 | GeneCards |
| PDPN | GeneCards |
| GSS | GeneCards |
| GBE1 | GeneCards |
| MS4A1 | GeneCards |
| ATF2 | GeneCards |
| AZU1 | GeneCards |
| RNY5 | GeneCards |
| DYSF | GeneCards |
| KPNA4 | GeneCards |
| TSLP | GeneCards |
| RB1CC1 | GeneCards |
| WIPI2 | GeneCards |
| PSORS1C1 | GeneCards |
| WNT9A | GeneCards |
| TNFAIP1 | GeneCards |
| HEXD | GeneCards |
| COL20A1 | GeneCards |
| PITRM1 | GeneCards |
| CXCL11 | GeneCards |
| ABL2 | GeneCards |
| LINC00472 | GeneCards |
| CLCN4 | GeneCards |
| ASGR1 | GeneCards |
| CD248 | GeneCards |
| PTGER2 | GeneCards |
| IGHMBP2 | GeneCards |
| C1D | GeneCards |
| USF2 | GeneCards |
| CCT2 | GeneCards |
| SKI | GeneCards |
| CHIA | GeneCards |
| FUT4 | GeneCards |
| LGALS9 | GeneCards |
| NLRC5 | GeneCards |
| MUCL3 | GeneCards |
| AP2A2 | GeneCards |
| LYN | GeneCards |
| CUL5 | GeneCards |
| ANXA3 | GeneCards |
| PLXNA1 | GeneCards |
| CCT5 | GeneCards |
| HORMAD2 | GeneCards |
| CD84 | GeneCards |
| GOLGA2 | GeneCards |
| DKK3 | GeneCards |
| ZNF543 | GeneCards |
| H1-5 | GeneCards |
| DEFA1A3 | GeneCards |
| SV2B | GeneCards |
| ORAI2 | GeneCards |
| SLC25A10 | GeneCards |
| GZMA | GeneCards |
| CDH11 | GeneCards |
| ARF3 | GeneCards |
| MPP7 | GeneCards |
| FCGR2B | GeneCards |
| PHLDB1 | GeneCards |
| DCAF1 | GeneCards |
| SLC25A11 | GeneCards |
| TRDN | GeneCards |
| GAMT | GeneCards |
| AICDA | GeneCards |
| C3AR1 | GeneCards |
| BACH1 | GeneCards |
| ADAM19 | GeneCards |
| IGKV3-20 | GeneCards |
| APOL4 | GeneCards |
| CHRM2 | GeneCards |
| ZBTB16 | GeneCards |
| SLC25A21 | GeneCards |
| MIR98 | GeneCards |
| MIR510 | GeneCards |
| GALNT10 | GeneCards |
| GATM | GeneCards |
| TBX3 | GeneCards |
| FAM151A | GeneCards |
| SSPOP | GeneCards |
| GRIN1 | GeneCards |
| WWTR1 | GeneCards |
| VCAN | GeneCards |
| HLA-S | GeneCards |
| ZBED5 | GeneCards |
| RPS27P20 | GeneCards |
| FAR2 | GeneCards |
| PAK2 | GeneCards |
| SEPTIN7 | GeneCards |
| CXCL13 | GeneCards |
| FCAMR | GeneCards |
| CUX1 | GeneCards |
| CORO2B | GeneCards |
| EEF1G | GeneCards |
| GTPBP4 | GeneCards |
| SPAG5 | GeneCards |
| TMEM212 | GeneCards |
| SPAG5-AS1 | GeneCards |
| ACOXL | GeneCards |
| ACTN1 | GeneCards |
| MIR4767 | GeneCards |
| TNS2 | GeneCards |
| DNM3 | GeneCards |
| SCAI | GeneCards |
| ARRB1 | GeneCards |
| PAQR3 | GeneCards |
| EEF1D | GeneCards |
| MYO1E | GeneCards |
| RIPK3 | GeneCards |
| CFL1 | GeneCards |
| SDCBP | GeneCards |
| C4BPB | GeneCards |
| SNTA1 | GeneCards |
| SNTB2 | GeneCards |
| PRDX6 | GeneCards |
| UTRN | GeneCards |
| KDM3B | GeneCards |
| AZIN2 | GeneCards |
| NAT8 | GeneCards |
| YBX1 | GeneCards |
| CARNS1 | GeneCards |
| ID1 | GeneCards |
| ADCK2 | GeneCards |
| GPR146 | GeneCards |
| BMPR1B | GeneCards |
| SLC6A8 | GeneCards |
| CCL13 | GeneCards |
| SMURF2 | GeneCards |
| PIP5K1C | GeneCards |
| MEP1A | GeneCards |
| GZF1 | GeneCards |
| ANKRD1 | GeneCards |
| DUSP13 | GeneCards |
| SERPINB4 | GeneCards |
| DEFT1P2 | GeneCards |
| CCT4 | GeneCards |
| VAMP1 | GeneCards |
| VAV2 | GeneCards |
| HIPK3 | GeneCards |
| HIPK1 | GeneCards |
| SGK2 | GeneCards |
| PPM1A | GeneCards |
| BMI1 | GeneCards |
| RAB34 | GeneCards |
| CCT8 | GeneCards |
| AMPD3 | GeneCards |
| DAO | GeneCards |
| MARK2 | GeneCards |
| PARVA | GeneCards |
| SGK3 | GeneCards |
| ANLN | GeneCards |
| NLN | GeneCards |
| SMOX | GeneCards |
| FERMT2 | GeneCards |
| GLIPR2 | GeneCards |
| GPR182 | GeneCards |
| IGKC | GeneCards |
| ABCC8 | OMIM |
| ACE | OMIM |
| AKT2 | OMIM |
| APPL1 | OMIM |
| AQP2 | OMIM |
| AVP | OMIM |
| AVPR2 | OMIM |
| BLK | OMIM |
| CAPN10 | OMIM |
| CCR5 | OMIM |
| CD151 | OMIM |
| CEL | OMIM |
| CFHR5 | OMIM |
| COL4A1 | OMIM |
| CTLA4 | OMIM |
| DNAJC3 | OMIM |
| ENPP1 | OMIM |
| EPO | OMIM |
| FOXC2 | OMIM |
| GCK | OMIM |
| GLIS3 | OMIM |
| GPD2 | OMIM |
| HFE | OMIM |
| HMGA1 | OMIM |
| HNF1A | OMIM |
| HNF1B | OMIM |
| HNF4A | OMIM |
| IAPP | OMIM |
| IER3IP1 | OMIM |
| IGF2BP2 | OMIM |
| IL1RN | OMIM |
| IL2RA | OMIM |
| IL6 | OMIM |
| INS | OMIM |
| INSR | OMIM |
| IRS1 | OMIM |
| IRS2 | OMIM |
| ITPR3 | OMIM |
| KCNJ11 | OMIM |
| KLF11 | OMIM |
| LIPC | OMIM |
| MAFA | OMIM |
| MAPK8IP1 | OMIM |
| MTNR1B | OMIM |
| NEUROD1 | OMIM |
| PAX4 | OMIM |
| PDX1 | OMIM |
| PON1 | OMIM |
| PPARG | OMIM |
| PTPN22 | OMIM |
| REN | OMIM |
| RETN | OMIM |
| RRAD | OMIM |
| SEC61A1 | OMIM |
| SLC2A2 | OMIM |
| SLC30A8 | OMIM |
| SOD2 | OMIM |
| SPINK1 | OMIM |
| SPRY2 | OMIM |
| SUMO4 | OMIM |
| TBC1D4 | OMIM |
| TCF7L2 | OMIM |
| UCP3 | OMIM |
| UMOD | OMIM |
| VEGFA | OMIM |
| WFS1 | OMIM |
| XPNPEP3 | OMIM |
| ZFP57 | OMIM |
| DIANPH | PharmGkb |
| AVPR2 | PharmGkb |
| IDDM11 | PharmGkb |
| IDDM13 | PharmGkb |
| IDDM15 | PharmGkb |
| IDDM17 | PharmGkb |
| IDDM4 | PharmGkb |
| IDDM6 | PharmGkb |
| IDDM8 | PharmGkb |
| IDDM3 | PharmGkb |
| IDDM7 | PharmGkb |
| IDDM9 | PharmGkb |
| IDDM16 | PharmGkb |
| IDDM18 | PharmGkb |
| IDDM14 | PharmGkb |
| NIDDM1 | PharmGkb |
| NIDDM2 | PharmGkb |
| TP53INP2 | PharmGkb |
| ANKRD23 | PharmGkb |
| SOX13 | PharmGkb |
| ATP5MD | PharmGkb |
| RRAD | PharmGkb |
| PEA15 | PharmGkb |
| PRG4 | PharmGkb |
| NPHP4 | PharmGkb |
| NPHP3 | PharmGkb |
| NPHP1 | PharmGkb |
| AVP | PharmGkb |
| NPHS2 | PharmGkb |
| OSR1 | PharmGkb |
| CEP290 | PharmGkb |
| FGF8 | PharmGkb |
| BMP7 | PharmGkb |
| WT1 | PharmGkb |
| LHON | PharmGkb |
| IBGC1 | PharmGkb |
| EGI | PharmGkb |
| CMTX3 | PharmGkb |
| CMTX2 | PharmGkb |
| NPHS1 | PharmGkb |
| INVS | PharmGkb |
| IRX1 | PharmGkb |
| SDCCAG8 | PharmGkb |
| IQCB1 | PharmGkb |
| REN | PharmGkb |
| CCN3 | PharmGkb |
| CLDN16 | PharmGkb |
| NPNT | PharmGkb |
| HOXC11 | PharmGkb |
| GLIS2 | PharmGkb |
| GDNF | PharmGkb |
| SIX2 | PharmGkb |
| GJB1 | PharmGkb |
| PNPLA6 | PharmGkb |
| TSHZ3 | PharmGkb |
| HOXD11 | PharmGkb |
| GDF11 | PharmGkb |
| SLC5A1 | PharmGkb |
| SPRY1 | PharmGkb |
| ITGA8 | PharmGkb |
| PAX8 | PharmGkb |
| HOXA11 | PharmGkb |
| FGF10 | PharmGkb |
| ID3 | PharmGkb |
| ROBO2 | PharmGkb |
| RDH10 | PharmGkb |
| BMP4 | PharmGkb |
| PAX2 | PharmGkb |
| PLCE1 | PharmGkb |
| EYA1 | PharmGkb |
| FOXC2 | PharmGkb |
| NIPBL | PharmGkb |
| ZBTB16 | PharmGkb |
| CITED1 | PharmGkb |
| NF1 | PharmGkb |
| SLIT2 | PharmGkb |
| GLI3 | PharmGkb |
| BCL2 | PharmGkb |
| SHH | PharmGkb |
| ACE | DrugBank |
| MTHFR | DrugBank |
| SLC15A1 | DrugBank |
| SLC15A2 | DrugBank |
| UGT1A9 | DrugBank |
| UGT2B4 | DrugBank |
| CYP3A4 | DrugBank |
| SLC5A2 | DrugBank |
| ABCB1 | DrugBank |
| ABCC2 | DrugBank |
| ORM1 | DrugBank |
| ABCG2 | DrugBank |
| AGTR1 | DrugBank |
| CYP2C8 | DrugBank |
| CYP2C9 | DrugBank |
| UGT1A3 | DrugBank |
| PTGS1 | DrugBank |
| ALB | DrugBank |
| MMP2 | DrugBank |
| MMP9 | DrugBank |
| SLC22A6 | DrugBank |
| LTA4H | DrugBank |
| BDKRB1 | DrugBank |
| SLC22A8 | DrugBank |
| SLC22A7 | DrugBank |
| SLCO1A2 | DrugBank |
| JUN | DrugBank |
| ORM2 | DrugBank |
| REN | DrugBank |
| UGT1A1 | DrugBank |
| UGT1A10 | DrugBank |
| UGT2B7 | DrugBank |
| UGT2B17 | DrugBank |
| CYP2C19 | DrugBank |
| SLC22A12 | DrugBank |
| SLC2A9 | DrugBank |
| ABCB11 | DrugBank |
| SLCO1B3 | DrugBank |
| SLCO1B1 | DrugBank |
| BCHE | DrugBank |
| SFRP4 | DrugBank |
| CES1 | DrugBank |
| PPARG | DrugBank |
