# Supplementary material for: Network pharmacology and molecular docking technology-based predictive study of the active ingredients and potential targets of rhubarb for the treatment of diabetic nephropathy
Source: BMC Complement Med Ther. 2022 Aug 6;22:210. doi: 10.1186/s12906-022-03662-6 (PMC9356435; doi:10.1186/s12906-022-03662-6)
Supplement: Supplementary file 5 — Additional file 5. [file 12906_2022_3662_MOESM5_ESM.docx]

| ONTOLOGY | ID | Description | GeneRatio | pvalue | p.adjust | qvalue | geneID | Count |
| --- | --- | --- | --- | --- | --- | --- | --- | --- |
| BP | GO:0006352 | DNA-templated transcription, initiation | 12/37 | 3.35E-14 | 7.10E-11 | 3.11E-11 | AR/ESR2/PPARD/JUN/ESR1/PGR/BAX/CDKN1A/TP53/CDK1/CCNB1/PPARG | 12 |
| BP | GO:0006367 | transcription initiation from RNA polymerase II promoter | 11/37 | 5.47E-14 | 7.10E-11 | 3.11E-11 | AR/ESR2/PPARD/ESR1/PGR/BAX/CDKN1A/TP53/CDK1/CCNB1/PPARG | 11 |
| BP | GO:0035690 | cellular response to drug | 13/37 | 1.32E-13 | 1.14E-10 | 5.00E-11 | NOS2/PTGS2/KDR/DRD1/CHRM2/CASP9/TP53/CDK1/PCNA/MYC/IL1B/PRKCD/DPEP1 | 13 |
| BP | GO:0048545 | response to steroid hormone | 13/37 | 2.27E-13 | 1.31E-10 | 5.73E-11 | AR/PTGS2/ESR2/NCOA2/PPARD/ESR1/PGR/BCL2/CASP9/CASP3/CDKN1A/PCNA/PPARG | 13 |
| BP | GO:0048511 | rhythmic process | 12/37 | 2.52E-13 | 1.31E-10 | 5.73E-11 | NOS2/F7/NCOA2/JUN/ESR1/PGR/SLC6A4/CASP3/TP53/CDK1/PCNA/PPARG | 12 |
| BP | GO:0070482 | response to oxygen levels | 13/37 | 3.05E-13 | 1.32E-10 | 5.77E-11 | NOS2/PTGS2/F7/DPP4/PPARD/SLC6A4/BCL2/CASP3/CDKN1A/TP53/MYC/CCNB1/PPARG | 13 |
| BP | GO:0048145 | regulation of fibroblast proliferation | 8/37 | 3.75E-12 | 1.34E-09 | 5.88E-10 | JUN/ESR1/BAX/CDKN1A/TP53/MYC/CCNB1/PPARG | 8 |
| BP | GO:0048144 | fibroblast proliferation | 8/37 | 4.14E-12 | 1.34E-09 | 5.88E-10 | JUN/ESR1/BAX/CDKN1A/TP53/MYC/CCNB1/PPARG | 8 |
| BP | GO:0031667 | response to nutrient levels | 13/37 | 6.03E-12 | 1.66E-09 | 7.29E-10 | PTGS2/F7/PPARD/JUN/ADRB2/SLC6A4/BCL2/PON1/CDKN1A/TP53/PCNA/IL1B/PPARG | 13 |
| BP | GO:0009411 | response to UV | 9/37 | 6.42E-12 | 1.66E-09 | 7.29E-10 | PTGS2/BCL2/BAX/CASP9/CASP3/CDKN1A/TP53/PCNA/MYC | 9 |
| BP | GO:0071214 | cellular response to abiotic stimulus | 11/37 | 2.61E-11 | 5.65E-09 | 2.47E-09 | PTGS2/AKR1B1/BAX/CASP9/CASP3/CASP8/CDKN1A/TP53/PCNA/MYC/IL1B | 11 |
| BP | GO:0104004 | cellular response to environmental stimulus | 11/37 | 2.61E-11 | 5.65E-09 | 2.47E-09 | PTGS2/AKR1B1/BAX/CASP9/CASP3/CASP8/CDKN1A/TP53/PCNA/MYC/IL1B | 11 |
| BP | GO:0001666 | response to hypoxia | 11/37 | 6.24E-11 | 1.24E-08 | 5.45E-09 | NOS2/PTGS2/F7/DPP4/PPARD/SLC6A4/BCL2/CASP3/TP53/MYC/CCNB1 | 11 |
| BP | GO:0034349 | glial cell apoptotic process | 5/37 | 6.83E-11 | 1.27E-08 | 5.54E-09 | CASP9/CASP3/PRKCA/TP53/PRKCD | 5 |
| BP | GO:0036293 | response to decreased oxygen levels | 11/37 | 8.61E-11 | 1.49E-08 | 6.52E-09 | NOS2/PTGS2/F7/DPP4/PPARD/SLC6A4/BCL2/CASP3/TP53/MYC/CCNB1 | 11 |
| BP | GO:0016572 | histone phosphorylation | 6/37 | 1.04E-10 | 1.69E-08 | 7.41E-09 | DRD1/PRKCA/CDK1/IL1B/PRKCD/CCNB1 | 6 |
| BP | GO:0031099 | regeneration | 9/37 | 1.36E-10 | 2.08E-08 | 9.10E-09 | F7/PPARD/JUN/BCL2/CDKN1A/CDK1/PCNA/CCNB1/PPARG | 9 |
| BP | GO:0032355 | response to estradiol | 8/37 | 1.85E-10 | 2.66E-08 | 1.17E-08 | PTGS2/F7/ESR1/SLC6A4/CASP9/CASP3/CASP8/PCNA | 8 |
| BP | GO:0034644 | cellular response to UV | 7/37 | 2.25E-10 | 3.07E-08 | 1.34E-08 | PTGS2/BAX/CASP9/CDKN1A/TP53/PCNA/MYC | 7 |
| BP | GO:0009416 | response to light stimulus | 10/37 | 3.66E-10 | 4.65E-08 | 2.04E-08 | PTGS2/DRD1/BCL2/BAX/CASP9/CASP3/CDKN1A/TP53/PCNA/MYC | 10 |
| BP | GO:0044706 | multi-multicellular organism process | 9/37 | 3.76E-10 | 4.65E-08 | 2.04E-08 | AR/PTGS2/PPARD/AKR1B1/ESR1/PGR/SLC6A4/BCL2/IL1B | 9 |
| BP | GO:0009314 | response to radiation | 11/37 | 6.55E-10 | 7.72E-08 | 3.38E-08 | PTGS2/JUN/DRD1/BCL2/BAX/CASP9/CASP3/CDKN1A/TP53/PCNA/MYC | 11 |
| BP | GO:0001101 | response to acid chemical | 10/37 | 8.62E-10 | 9.72E-08 | 4.26E-08 | PTGS2/F7/KDR/AKR1B1/SLC6A4/CASP3/PON1/PCNA/CCNB1/PPARG | 10 |
| BP | GO:0006977 | DNA damage response, signal transduction by p53 class mediator resulting in cell cycle arrest | 6/37 | 1.20E-09 | 1.29E-07 | 5.66E-08 | BAX/CDKN1A/TP53/CDK1/PCNA/CCNB1 | 6 |
| BP | GO:0072431 | signal transduction involved in mitotic G1 DNA damage checkpoint | 6/37 | 1.33E-09 | 1.33E-07 | 5.83E-08 | BAX/CDKN1A/TP53/CDK1/PCNA/CCNB1 | 6 |
| BP | GO:1902400 | intracellular signal transduction involved in G1 DNA damage checkpoint | 6/37 | 1.33E-09 | 1.33E-07 | 5.83E-08 | BAX/CDKN1A/TP53/CDK1/PCNA/CCNB1 | 6 |
| BP | GO:0010038 | response to metal ion | 10/37 | 1.53E-09 | 1.43E-07 | 6.26E-08 | PTGS2/JUN/BCL2/CASP9/CASP3/CASP8/CDK1/PCNA/CCNB1/DPEP1 | 10 |
| BP | GO:0072413 | signal transduction involved in mitotic cell cycle checkpoint | 6/37 | 1.65E-09 | 1.43E-07 | 6.26E-08 | BAX/CDKN1A/TP53/CDK1/PCNA/CCNB1 | 6 |
| BP | GO:1902402 | signal transduction involved in mitotic DNA damage checkpoint | 6/37 | 1.65E-09 | 1.43E-07 | 6.26E-08 | BAX/CDKN1A/TP53/CDK1/PCNA/CCNB1 | 6 |
| BP | GO:1902403 | signal transduction involved in mitotic DNA integrity checkpoint | 6/37 | 1.65E-09 | 1.43E-07 | 6.26E-08 | BAX/CDKN1A/TP53/CDK1/PCNA/CCNB1 | 6 |
| BP | GO:0031571 | mitotic G1 DNA damage checkpoint | 6/37 | 2.48E-09 | 2.01E-07 | 8.79E-08 | BAX/CDKN1A/TP53/CDK1/PCNA/CCNB1 | 6 |
| BP | GO:0044819 | mitotic G1/S transition checkpoint | 6/37 | 2.48E-09 | 2.01E-07 | 8.79E-08 | BAX/CDKN1A/TP53/CDK1/PCNA/CCNB1 | 6 |
| BP | GO:0044783 | G1 DNA damage checkpoint | 6/37 | 2.73E-09 | 2.08E-07 | 9.12E-08 | BAX/CDKN1A/TP53/CDK1/PCNA/CCNB1 | 6 |
| BP | GO:0060135 | maternal process involved in female pregnancy | 6/37 | 2.73E-09 | 2.08E-07 | 9.12E-08 | AR/PTGS2/PPARD/AKR1B1/ESR1/PGR | 6 |
| BP | GO:0007565 | female pregnancy | 8/37 | 3.23E-09 | 2.40E-07 | 1.05E-07 | AR/PTGS2/PPARD/AKR1B1/ESR1/PGR/BCL2/IL1B | 8 |
| BP | GO:1901654 | response to ketone | 8/37 | 3.37E-09 | 2.43E-07 | 1.06E-07 | AR/F7/NCOA2/AKR1B1/CASP9/CDKN1A/PCNA/PPARG | 8 |
| BP | GO:2000134 | negative regulation of G1/S transition of mitotic cell cycle | 7/37 | 4.44E-09 | 3.11E-07 | 1.36E-07 | BCL2/BAX/CDKN1A/TP53/CDK1/PCNA/CCNB1 | 7 |
| BP | GO:0071482 | cellular response to light stimulus | 7/37 | 5.53E-09 | 3.77E-07 | 1.65E-07 | PTGS2/BAX/CASP9/CDKN1A/TP53/PCNA/MYC | 7 |
| BP | GO:0007623 | circadian rhythm | 8/37 | 6.07E-09 | 3.80E-07 | 1.66E-07 | NOS2/F7/NCOA2/JUN/SLC6A4/TP53/CDK1/PPARG | 8 |
| BP | GO:0072401 | signal transduction involved in DNA integrity checkpoint | 6/37 | 6.12E-09 | 3.80E-07 | 1.66E-07 | BAX/CDKN1A/TP53/CDK1/PCNA/CCNB1 | 6 |
| BP | GO:0072422 | signal transduction involved in DNA damage checkpoint | 6/37 | 6.12E-09 | 3.80E-07 | 1.66E-07 | BAX/CDKN1A/TP53/CDK1/PCNA/CCNB1 | 6 |
| BP | GO:1902807 | negative regulation of cell cycle G1/S phase transition | 7/37 | 6.16E-09 | 3.80E-07 | 1.66E-07 | BCL2/BAX/CDKN1A/TP53/CDK1/PCNA/CCNB1 | 7 |
| BP | GO:0072395 | signal transduction involved in cell cycle checkpoint | 6/37 | 6.65E-09 | 4.01E-07 | 1.76E-07 | BAX/CDKN1A/TP53/CDK1/PCNA/CCNB1 | 6 |
| BP | GO:0042770 | signal transduction in response to DNA damage | 7/37 | 6.84E-09 | 4.03E-07 | 1.77E-07 | BAX/CASP9/CDKN1A/TP53/CDK1/PCNA/CCNB1 | 7 |
| BP | GO:0048608 | reproductive structure development | 10/37 | 7.73E-09 | 4.45E-07 | 1.95E-07 | AR/PTGS2/PPARD/ESR1/PGR/BCL2/BAX/CASP3/CASP8/PPARG | 10 |
| BP | GO:0050673 | epithelial cell proliferation | 10/37 | 8.26E-09 | 4.56E-07 | 2.00E-07 | AR/KDR/PPARD/JUN/ESR1/PGR/BAX/PRKCA/MYC/PPARG | 10 |
| BP | GO:0061458 | reproductive system development | 10/37 | 8.26E-09 | 4.56E-07 | 2.00E-07 | AR/PTGS2/PPARD/ESR1/PGR/BCL2/BAX/CASP3/CASP8/PPARG | 10 |
| BP | GO:0046677 | response to antibiotic | 9/37 | 1.12E-08 | 6.07E-07 | 2.66E-07 | JUN/BCL2/CASP9/CASP3/CASP8/TP53/CDK1/PCNA/PRKCD | 9 |
| BP | GO:0006979 | response to oxidative stress | 10/37 | 1.19E-08 | 6.30E-07 | 2.76E-07 | PTGS2/PTGS1/JUN/BCL2/CASP3/TP53/CDK1/PCNA/PRKCD/DPEP1 | 10 |
| BP | GO:0032496 | response to lipopolysaccharide | 9/37 | 1.22E-08 | 6.30E-07 | 2.76E-07 | NOS2/PTGS2/PPARD/JUN/CASP9/CASP3/CASP8/PRKCA/IL1B | 9 |
| BP | GO:0071158 | positive regulation of cell cycle arrest | 6/37 | 1.24E-08 | 6.32E-07 | 2.77E-07 | BAX/CDKN1A/TP53/CDK1/PCNA/CCNB1 | 6 |
| BP | GO:0002237 | response to molecule of bacterial origin | 9/37 | 1.70E-08 | 8.47E-07 | 3.71E-07 | NOS2/PTGS2/PPARD/JUN/CASP9/CASP3/CASP8/PRKCA/IL1B | 9 |
| BP | GO:0033002 | muscle cell proliferation | 8/37 | 1.80E-08 | 8.80E-07 | 3.85E-07 | PTGS2/PPARD/AKR1B1/JUN/CDKN1A/CDK1/CCNB1/PPARG | 8 |
| BP | GO:0045926 | negative regulation of growth | 8/37 | 2.47E-08 | 1.19E-06 | 5.20E-07 | ESR2/PPARD/ADRB2/SLC6A4/BCL2/CDKN1A/TP53/PPARG | 8 |
| BP | GO:1901216 | positive regulation of neuron death | 6/37 | 2.84E-08 | 1.34E-06 | 5.87E-07 | JUN/BAX/CASP9/CASP3/CASP8/TP53 | 6 |
| BP | GO:0044773 | mitotic DNA damage checkpoint | 6/37 | 3.43E-08 | 1.59E-06 | 6.96E-07 | BAX/CDKN1A/TP53/CDK1/PCNA/CCNB1 | 6 |
| BP | GO:0050678 | regulation of epithelial cell proliferation | 9/37 | 3.92E-08 | 1.78E-06 | 7.81E-07 | AR/KDR/PPARD/JUN/PGR/BAX/PRKCA/MYC/PPARG | 9 |
| BP | GO:0045787 | positive regulation of cell cycle | 9/37 | 5.01E-08 | 2.23E-06 | 9.77E-07 | SLC6A4/BAX/PRKCA/CDKN1A/TP53/CDK1/PCNA/IL1B/CCNB1 | 9 |
| BP | GO:0048146 | positive regulation of fibroblast proliferation | 5/37 | 5.07E-08 | 2.23E-06 | 9.77E-07 | JUN/ESR1/CDKN1A/MYC/CCNB1 | 5 |
| BP | GO:0044774 | mitotic DNA integrity checkpoint | 6/37 | 5.85E-08 | 2.53E-06 | 1.11E-06 | BAX/CDKN1A/TP53/CDK1/PCNA/CCNB1 | 6 |
| BP | GO:0000082 | G1/S transition of mitotic cell cycle | 8/37 | 5.97E-08 | 2.54E-06 | 1.11E-06 | BCL2/BAX/CDKN1A/TP53/CDK1/PCNA/MYC/CCNB1 | 8 |
| BP | GO:0030330 | DNA damage response, signal transduction by p53 class mediator | 6/37 | 6.19E-08 | 2.59E-06 | 1.13E-06 | BAX/CDKN1A/TP53/CDK1/PCNA/CCNB1 | 6 |
| BP | GO:2000045 | regulation of G1/S transition of mitotic cell cycle | 7/37 | 6.46E-08 | 2.65E-06 | 1.16E-06 | BCL2/BAX/CDKN1A/TP53/CDK1/PCNA/CCNB1 | 7 |
| BP | GO:0071156 | regulation of cell cycle arrest | 6/37 | 6.54E-08 | 2.65E-06 | 1.16E-06 | BAX/CDKN1A/TP53/CDK1/PCNA/CCNB1 | 6 |
| BP | GO:0097193 | intrinsic apoptotic signaling pathway | 8/37 | 7.83E-08 | 3.12E-06 | 1.37E-06 | PTGS2/BCL2/BAX/CASP9/CASP3/CDKN1A/TP53/PRKCD | 8 |
| BP | GO:0010332 | response to gamma radiation | 5/37 | 8.19E-08 | 3.22E-06 | 1.41E-06 | BCL2/BAX/CDKN1A/TP53/MYC | 5 |
| BP | GO:0071478 | cellular response to radiation | 7/37 | 8.34E-08 | 3.23E-06 | 1.41E-06 | PTGS2/BAX/CASP9/CDKN1A/TP53/PCNA/MYC | 7 |
| BP | GO:0043525 | positive regulation of neuron apoptotic process | 5/37 | 8.97E-08 | 3.42E-06 | 1.50E-06 | JUN/BAX/CASP9/CASP3/TP53 | 5 |
| BP | GO:0044843 | cell cycle G1/S phase transition | 8/37 | 9.91E-08 | 3.72E-06 | 1.63E-06 | BCL2/BAX/CDKN1A/TP53/CDK1/PCNA/MYC/CCNB1 | 8 |
| BP | GO:0018105 | peptidyl-serine phosphorylation | 8/37 | 1.02E-07 | 3.77E-06 | 1.65E-06 | PTGS2/DRD1/BCL2/BAX/PRKCA/CDK1/PRKCD/CCNB1 | 8 |
| BP | GO:0051052 | regulation of DNA metabolic process | 9/37 | 1.15E-07 | 4.22E-06 | 1.85E-06 | JUN/BAX/CDKN1A/TP53/CDK1/PCNA/MYC/PRKCD/PPARG | 9 |
| BP | GO:1902806 | regulation of cell cycle G1/S phase transition | 7/37 | 1.22E-07 | 4.40E-06 | 1.93E-06 | BCL2/BAX/CDKN1A/TP53/CDK1/PCNA/CCNB1 | 7 |
| BP | GO:0007568 | aging | 8/37 | 1.75E-07 | 6.22E-06 | 2.72E-06 | PTGS2/JUN/BCL2/CASP9/CDKN1A/TP53/CDK1/PRKCD | 8 |
| BP | GO:0018209 | peptidyl-serine modification | 8/37 | 1.79E-07 | 6.28E-06 | 2.75E-06 | PTGS2/DRD1/BCL2/BAX/PRKCA/CDK1/PRKCD/CCNB1 | 8 |
| BP | GO:0043281 | regulation of cysteine-type endopeptidase activity involved in apoptotic process | 7/37 | 1.87E-07 | 6.47E-06 | 2.83E-06 | PTGS2/BAX/CASP9/CASP8/MYC/DPEP1/PPARG | 7 |
| BP | GO:0062012 | regulation of small molecule metabolic process | 9/37 | 2.05E-07 | 6.99E-06 | 3.06E-06 | NOS2/PTGS2/NCOA2/TP53/FASN/CDK1/IL1B/CCNB1/PPARG | 9 |
| BP | GO:0007584 | response to nutrient | 7/37 | 2.12E-07 | 7.14E-06 | 3.13E-06 | PTGS2/F7/PPARD/SLC6A4/PCNA/IL1B/PPARG | 7 |
| BP | GO:0097191 | extrinsic apoptotic signaling pathway | 7/37 | 2.47E-07 | 8.21E-06 | 3.60E-06 | AR/BCL2/BAX/CASP9/CASP3/CASP8/IL1B | 7 |
| BP | GO:0071496 | cellular response to external stimulus | 8/37 | 2.65E-07 | 8.71E-06 | 3.82E-06 | PTGS2/JUN/BCL2/CASP8/CDKN1A/TP53/IL1B/PPARG | 8 |
| BP | GO:0038034 | signal transduction in absence of ligand | 5/37 | 2.93E-07 | 9.39E-06 | 4.11E-06 | BCL2/BAX/CASP9/CASP3/IL1B | 5 |
| BP | GO:0097192 | extrinsic apoptotic signaling pathway in absence of ligand | 5/37 | 2.93E-07 | 9.39E-06 | 4.11E-06 | BCL2/BAX/CASP9/CASP3/IL1B | 5 |
| BP | GO:0000302 | response to reactive oxygen species | 7/37 | 3.13E-07 | 9.78E-06 | 4.29E-06 | JUN/BCL2/CASP3/CDK1/PCNA/PRKCD/DPEP1 | 7 |
| BP | GO:0031100 | animal organ regeneration | 5/37 | 3.14E-07 | 9.78E-06 | 4.29E-06 | F7/CDKN1A/CDK1/PCNA/PPARG | 5 |
| BP | GO:0048638 | regulation of developmental growth | 8/37 | 3.17E-07 | 9.78E-06 | 4.29E-06 | AR/PPARD/ADRB2/SLC6A4/BCL2/CDKN1A/CDK1/CCNB1 | 8 |
| BP | GO:0071453 | cellular response to oxygen levels | 7/37 | 3.32E-07 | 1.01E-05 | 4.44E-06 | PTGS2/PPARD/BCL2/TP53/MYC/CCNB1/PPARG | 7 |
| BP | GO:0001844 | protein insertion into mitochondrial membrane involved in apoptotic signaling pathway | 4/37 | 3.45E-07 | 1.03E-05 | 4.50E-06 | BCL2/BAX/CASP8/TP53 | 4 |
| BP | GO:0048147 | negative regulation of fibroblast proliferation | 4/37 | 3.45E-07 | 1.03E-05 | 4.50E-06 | BAX/TP53/MYC/PPARG | 4 |
| BP | GO:0007050 | cell cycle arrest | 7/37 | 3.62E-07 | 1.07E-05 | 4.67E-06 | BAX/CDKN1A/TP53/CDK1/PCNA/MYC/CCNB1 | 7 |
| BP | GO:0000077 | DNA damage checkpoint | 6/37 | 3.77E-07 | 1.10E-05 | 4.81E-06 | BAX/CDKN1A/TP53/CDK1/PCNA/CCNB1 | 6 |
| BP | GO:2000116 | regulation of cysteine-type endopeptidase activity | 7/37 | 3.83E-07 | 1.10E-05 | 4.83E-06 | PTGS2/BAX/CASP9/CASP8/MYC/DPEP1/PPARG | 7 |
| BP | GO:0042542 | response to hydrogen peroxide | 6/37 | 3.93E-07 | 1.11E-05 | 4.85E-06 | JUN/BCL2/CASP3/CDK1/PCNA/PRKCD | 6 |
| BP | GO:0051384 | response to glucocorticoid | 6/37 | 3.93E-07 | 1.11E-05 | 4.85E-06 | PTGS2/BCL2/CASP9/CASP3/CDKN1A/PCNA | 6 |
| BP | GO:0010212 | response to ionizing radiation | 6/37 | 4.09E-07 | 1.14E-05 | 4.99E-06 | BCL2/BAX/CASP3/CDKN1A/TP53/MYC | 6 |
| BP | GO:1901991 | negative regulation of mitotic cell cycle phase transition | 7/37 | 4.91E-07 | 1.36E-05 | 5.94E-06 | BCL2/BAX/CDKN1A/TP53/CDK1/PCNA/CCNB1 | 7 |
| BP | GO:0051204 | protein insertion into mitochondrial membrane | 4/37 | 5.13E-07 | 1.39E-05 | 6.07E-06 | BCL2/BAX/CASP8/TP53 | 4 |
| BP | GO:0062013 | positive regulation of small molecule metabolic process | 6/37 | 5.17E-07 | 1.39E-05 | 6.07E-06 | NOS2/PTGS2/CDK1/IL1B/CCNB1/PPARG | 6 |
| BP | GO:0071383 | cellular response to steroid hormone stimulus | 7/37 | 5.19E-07 | 1.39E-05 | 6.07E-06 | AR/ESR2/PPARD/ESR1/PGR/CASP9/PPARG | 7 |
| BP | GO:0006970 | response to osmotic stress | 5/37 | 5.99E-07 | 1.58E-05 | 6.91E-06 | PTGS2/AKR1B1/BAX/CASP3/TP53 | 5 |
| BP | GO:0031570 | DNA integrity checkpoint | 6/37 | 6.02E-07 | 1.58E-05 | 6.91E-06 | BAX/CDKN1A/TP53/CDK1/PCNA/CCNB1 | 6 |
| BP | GO:1901030 | positive regulation of mitochondrial outer membrane permeabilization involved in apoptotic signaling pathway | 4/37 | 6.54E-07 | 1.70E-05 | 7.43E-06 | BCL2/BAX/CASP8/TP53 | 4 |
| BP | GO:0031960 | response to corticosteroid | 6/37 | 7.24E-07 | 1.85E-05 | 8.10E-06 | PTGS2/BCL2/CASP9/CASP3/CDKN1A/PCNA | 6 |
| BP | GO:0030224 | monocyte differentiation | 4/37 | 7.35E-07 | 1.85E-05 | 8.10E-06 | JUN/FASN/MYC/PPARG | 4 |
| BP | GO:1903131 | mononuclear cell differentiation | 4/37 | 7.35E-07 | 1.85E-05 | 8.10E-06 | JUN/FASN/MYC/PPARG | 4 |
| BP | GO:0007093 | mitotic cell cycle checkpoint | 6/37 | 8.06E-07 | 1.97E-05 | 8.65E-06 | BAX/CDKN1A/TP53/CDK1/PCNA/CCNB1 | 6 |
| BP | GO:0072331 | signal transduction by p53 class mediator | 7/37 | 8.07E-07 | 1.97E-05 | 8.65E-06 | BCL2/BAX/CDKN1A/TP53/CDK1/PCNA/CCNB1 | 7 |
| BP | GO:1901988 | negative regulation of cell cycle phase transition | 7/37 | 8.07E-07 | 1.97E-05 | 8.65E-06 | BCL2/BAX/CDKN1A/TP53/CDK1/PCNA/CCNB1 | 7 |
| BP | GO:0032025 | response to cobalt ion | 3/37 | 8.52E-07 | 2.06E-05 | 9.04E-06 | CASP9/CASP3/CASP8 | 3 |
| BP | GO:1901568 | fatty acid derivative metabolic process | 6/37 | 8.65E-07 | 2.08E-05 | 9.10E-06 | PTGS2/PTGS1/PON1/FASN/IL1B/DPEP1 | 6 |
| BP | GO:0046651 | lymphocyte proliferation | 7/37 | 9.14E-07 | 2.17E-05 | 9.52E-06 | BCL2/BAX/CASP3/CDKN1A/TP53/IL1B/PRKCD | 7 |
| BP | GO:0048660 | regulation of smooth muscle cell proliferation | 6/37 | 9.27E-07 | 2.19E-05 | 9.57E-06 | PTGS2/PPARD/AKR1B1/JUN/CDKN1A/PPARG | 6 |
| BP | GO:0032943 | mononuclear cell proliferation | 7/37 | 9.60E-07 | 2.24E-05 | 9.82E-06 | BCL2/BAX/CASP3/CDKN1A/TP53/IL1B/PRKCD | 7 |
| BP | GO:0048659 | smooth muscle cell proliferation | 6/37 | 9.93E-07 | 2.30E-05 | 1.01E-05 | PTGS2/PPARD/AKR1B1/JUN/CDKN1A/PPARG | 6 |
| BP | GO:2001233 | regulation of apoptotic signaling pathway | 8/37 | 1.04E-06 | 2.38E-05 | 1.04E-05 | AR/PTGS2/BCL2/BAX/CASP8/TP53/IL1B/PRKCD | 8 |
| BP | GO:0030522 | intracellular receptor signaling pathway | 7/37 | 1.11E-06 | 2.52E-05 | 1.10E-05 | AR/ESR2/PPARD/ESR1/PGR/CASP8/PPARG | 7 |
| BP | GO:0042100 | B cell proliferation | 5/37 | 1.18E-06 | 2.65E-05 | 1.16E-05 | BCL2/BAX/CASP3/CDKN1A/PRKCD | 5 |
| BP | GO:0072593 | reactive oxygen species metabolic process | 7/37 | 1.22E-06 | 2.73E-05 | 1.19E-05 | NOS2/PTGS2/BCL2/CDKN1A/TP53/IL1B/PRKCD | 7 |
| BP | GO:0008585 | female gonad development | 5/37 | 1.24E-06 | 2.74E-05 | 1.20E-05 | ESR1/PGR/BCL2/BAX/CASP3 | 5 |
| BP | GO:0030099 | myeloid cell differentiation | 8/37 | 1.24E-06 | 2.74E-05 | 1.20E-05 | JUN/CASP9/CASP3/CASP8/PRKCA/FASN/MYC/PPARG | 8 |
| BP | GO:0008217 | regulation of blood pressure | 6/37 | 1.43E-06 | 3.11E-05 | 1.36E-05 | NOS2/AR/PTGS2/PTGS1/ADRB2/PPARG | 6 |
| BP | GO:0009410 | response to xenobiotic stimulus | 7/37 | 1.47E-06 | 3.17E-05 | 1.39E-05 | F7/PTGS1/DRD1/CASP9/PCNA/DPEP1/PPARG | 7 |
| BP | GO:0043401 | steroid hormone mediated signaling pathway | 6/37 | 1.52E-06 | 3.26E-05 | 1.43E-05 | AR/ESR2/PPARD/ESR1/PGR/PPARG | 6 |
| BP | GO:0007077 | mitotic nuclear envelope disassembly | 3/37 | 1.56E-06 | 3.28E-05 | 1.44E-05 | PRKCA/CDK1/CCNB1 | 3 |
| BP | GO:0010623 | programmed cell death involved in cell development | 3/37 | 1.56E-06 | 3.28E-05 | 1.44E-05 | BCL2/BAX/IL1B | 3 |
| BP | GO:0001933 | negative regulation of protein phosphorylation | 8/37 | 1.57E-06 | 3.28E-05 | 1.44E-05 | JUN/BAX/CASP3/CDKN1A/MYC/IL1B/PRKCD/CCNB1 | 8 |
| BP | GO:0046545 | development of primary female sexual characteristics | 5/37 | 1.59E-06 | 3.30E-05 | 1.45E-05 | ESR1/PGR/BCL2/BAX/CASP3 | 5 |
| BP | GO:0030308 | negative regulation of cell growth | 6/37 | 1.62E-06 | 3.34E-05 | 1.46E-05 | ESR2/PPARD/BCL2/CDKN1A/TP53/PPARG | 6 |
| BP | GO:2000379 | positive regulation of reactive oxygen species metabolic process | 5/37 | 1.67E-06 | 3.35E-05 | 1.47E-05 | PTGS2/CDKN1A/TP53/IL1B/PRKCD | 5 |
| BP | GO:1901028 | regulation of mitochondrial outer membrane permeabilization involved in apoptotic signaling pathway | 4/37 | 1.67E-06 | 3.35E-05 | 1.47E-05 | BCL2/BAX/CASP8/TP53 | 4 |
| BP | GO:0070661 | leukocyte proliferation | 7/37 | 1.68E-06 | 3.35E-05 | 1.47E-05 | BCL2/BAX/CASP3/CDKN1A/TP53/IL1B/PRKCD | 7 |
| BP | GO:0090068 | positive regulation of cell cycle process | 7/37 | 1.68E-06 | 3.35E-05 | 1.47E-05 | BAX/CDKN1A/TP53/CDK1/PCNA/IL1B/CCNB1 | 7 |
| BP | GO:0048732 | gland development | 8/37 | 1.71E-06 | 3.38E-05 | 1.48E-05 | AR/JUN/ESR1/PGR/BCL2/BAX/FASN/PCNA | 8 |
| BP | GO:0034599 | cellular response to oxidative stress | 7/37 | 1.84E-06 | 3.59E-05 | 1.57E-05 | JUN/BCL2/TP53/CDK1/PCNA/PRKCD/DPEP1 | 7 |
| BP | GO:0008630 | intrinsic apoptotic signaling pathway in response to DNA damage | 5/37 | 1.84E-06 | 3.59E-05 | 1.57E-05 | BCL2/BAX/CASP9/CDKN1A/TP53 | 5 |
| BP | GO:0060443 | mammary gland morphogenesis | 4/37 | 2.01E-06 | 3.88E-05 | 1.70E-05 | AR/ESR1/PGR/BAX | 4 |
| BP | GO:2000377 | regulation of reactive oxygen species metabolic process | 6/37 | 2.13E-06 | 4.10E-05 | 1.80E-05 | PTGS2/BCL2/CDKN1A/TP53/IL1B/PRKCD | 6 |
| BP | GO:0042113 | B cell activation | 7/37 | 2.18E-06 | 4.15E-05 | 1.82E-05 | BCL2/BAX/CASP3/CASP8/CDKN1A/TP53/PRKCD | 7 |
| BP | GO:0061614 | pri-miRNA transcription by RNA polymerase II | 4/37 | 2.19E-06 | 4.15E-05 | 1.82E-05 | PPARD/JUN/TP53/PPARG | 4 |
| BP | GO:1901214 | regulation of neuron death | 7/37 | 2.33E-06 | 4.37E-05 | 1.92E-05 | JUN/BCL2/BAX/CASP9/CASP3/CASP8/TP53 | 7 |
| BP | GO:0051205 | protein insertion into membrane | 4/37 | 2.59E-06 | 4.84E-05 | 2.12E-05 | BCL2/BAX/CASP8/TP53 | 4 |
| BP | GO:0002573 | myeloid leukocyte differentiation | 6/37 | 2.77E-06 | 5.10E-05 | 2.23E-05 | JUN/CASP8/PRKCA/FASN/MYC/PPARG | 6 |
| BP | GO:0035265 | organ growth | 6/37 | 2.77E-06 | 5.10E-05 | 2.23E-05 | AR/ESR1/SLC6A4/BCL2/CDK1/CCNB1 | 6 |
| BP | GO:0006690 | icosanoid metabolic process | 5/37 | 2.90E-06 | 5.29E-05 | 2.32E-05 | PTGS2/PTGS1/PON1/IL1B/DPEP1 | 5 |
| BP | GO:1903829 | positive regulation of cellular protein localization | 7/37 | 2.92E-06 | 5.30E-05 | 2.32E-05 | PTGS2/BCL2/CASP8/TP53/CDK1/IL1B/PRKCD | 7 |
| BP | GO:0042326 | negative regulation of phosphorylation | 8/37 | 2.99E-06 | 5.34E-05 | 2.34E-05 | JUN/BAX/CASP3/CDKN1A/MYC/IL1B/PRKCD/CCNB1 | 8 |
| BP | GO:0002064 | epithelial cell development | 6/37 | 3.02E-06 | 5.34E-05 | 2.34E-05 | AR/ESR1/PGR/CDKN1A/FASN/IL1B | 6 |
| BP | GO:0071456 | cellular response to hypoxia | 6/37 | 3.02E-06 | 5.34E-05 | 2.34E-05 | PTGS2/PPARD/BCL2/TP53/MYC/CCNB1 | 6 |
| BP | GO:0046660 | female sex differentiation | 5/37 | 3.03E-06 | 5.34E-05 | 2.34E-05 | ESR1/PGR/BCL2/BAX/CASP3 | 5 |
| BP | GO:0007569 | cell aging | 5/37 | 3.16E-06 | 5.53E-05 | 2.42E-05 | BCL2/CDKN1A/TP53/CDK1/PRKCD | 5 |
| BP | GO:0009612 | response to mechanical stimulus | 6/37 | 3.28E-06 | 5.63E-05 | 2.47E-05 | PTGS2/JUN/CASP8/IL1B/CCNB1/PPARG | 6 |
| BP | GO:0043523 | regulation of neuron apoptotic process | 6/37 | 3.28E-06 | 5.63E-05 | 2.47E-05 | JUN/BCL2/BAX/CASP9/CASP3/TP53 | 6 |
| BP | GO:0010822 | positive regulation of mitochondrion organization | 5/37 | 3.29E-06 | 5.63E-05 | 2.47E-05 | KDR/BCL2/BAX/CASP8/TP53 | 5 |
| BP | GO:0001655 | urogenital system development | 7/37 | 3.30E-06 | 5.63E-05 | 2.47E-05 | AR/AKR1B1/ESR1/BCL2/BAX/CASP9/MYC | 7 |
| BP | GO:2001020 | regulation of response to DNA damage stimulus | 6/37 | 3.65E-06 | 6.19E-05 | 2.71E-05 | BCL2/CASP9/TP53/PCNA/MYC/PRKCD | 6 |
| BP | GO:0022612 | gland morphogenesis | 5/37 | 3.73E-06 | 6.26E-05 | 2.74E-05 | AR/ESR1/PGR/BCL2/BAX | 5 |
| BP | GO:0097345 | mitochondrial outer membrane permeabilization | 4/37 | 3.85E-06 | 6.26E-05 | 2.74E-05 | BCL2/BAX/CASP8/TP53 | 4 |
| BP | GO:0000075 | cell cycle checkpoint | 6/37 | 3.85E-06 | 6.26E-05 | 2.74E-05 | BAX/CDKN1A/TP53/CDK1/PCNA/CCNB1 | 6 |
| BP | GO:0045930 | negative regulation of mitotic cell cycle | 7/37 | 3.87E-06 | 6.26E-05 | 2.74E-05 | BCL2/BAX/CDKN1A/TP53/CDK1/PCNA/CCNB1 | 7 |
| BP | GO:0048871 | multicellular organismal homeostasis | 8/37 | 3.89E-06 | 6.26E-05 | 2.74E-05 | PTGS2/AKR1B1/DRD1/ADRB2/BCL2/BAX/PRKCA/IL1B | 8 |
| BP | GO:0030397 | membrane disassembly | 3/37 | 3.94E-06 | 6.26E-05 | 2.74E-05 | PRKCA/CDK1/CCNB1 | 3 |
| BP | GO:0032225 | regulation of synaptic transmission, dopaminergic | 3/37 | 3.94E-06 | 6.26E-05 | 2.74E-05 | PTGS2/DRD1/SLC6A4 | 3 |
| BP | GO:0051081 | nuclear envelope disassembly | 3/37 | 3.94E-06 | 6.26E-05 | 2.74E-05 | PRKCA/CDK1/CCNB1 | 3 |
| BP | GO:0060965 | negative regulation of gene silencing by miRNA | 3/37 | 3.94E-06 | 6.26E-05 | 2.74E-05 | ESR1/TP53/PPARG | 3 |
| BP | GO:0008406 | gonad development | 6/37 | 3.96E-06 | 6.26E-05 | 2.74E-05 | AR/ESR1/PGR/BCL2/BAX/CASP3 | 6 |
| BP | GO:0036294 | cellular response to decreased oxygen levels | 6/37 | 3.96E-06 | 6.26E-05 | 2.74E-05 | PTGS2/PPARD/BCL2/TP53/MYC/CCNB1 | 6 |
| BP | GO:0008637 | apoptotic mitochondrial changes | 5/37 | 4.38E-06 | 6.89E-05 | 3.02E-05 | JUN/BCL2/BAX/CASP8/TP53 | 5 |
| BP | GO:0045137 | development of primary sexual characteristics | 6/37 | 4.63E-06 | 7.23E-05 | 3.17E-05 | AR/ESR1/PGR/BCL2/BAX/CASP3 | 6 |
| BP | GO:0070997 | neuron death | 7/37 | 4.68E-06 | 7.27E-05 | 3.18E-05 | JUN/BCL2/BAX/CASP9/CASP3/CASP8/TP53 | 7 |
| BP | GO:0031649 | heat generation | 3/37 | 4.78E-06 | 7.38E-05 | 3.23E-05 | PTGS2/ADRB2/IL1B | 3 |
| BP | GO:0010631 | epithelial cell migration | 7/37 | 4.95E-06 | 7.60E-05 | 3.33E-05 | PTGS2/DPP4/KDR/PPARD/JUN/PRKCA/PPARG | 7 |
| BP | GO:0090132 | epithelium migration | 7/37 | 5.24E-06 | 7.98E-05 | 3.49E-05 | PTGS2/DPP4/KDR/PPARD/JUN/PRKCA/PPARG | 7 |
| BP | GO:0051054 | positive regulation of DNA metabolic process | 6/37 | 5.26E-06 | 7.98E-05 | 3.49E-05 | JUN/BAX/CDK1/PCNA/MYC/PRKCD | 6 |
| BP | GO:0001836 | release of cytochrome c from mitochondria | 4/37 | 5.49E-06 | 8.28E-05 | 3.63E-05 | JUN/BCL2/BAX/TP53 | 4 |
| BP | GO:2001234 | negative regulation of apoptotic signaling pathway | 6/37 | 5.53E-06 | 8.29E-05 | 3.63E-05 | AR/PTGS2/BCL2/BAX/CASP8/IL1B | 6 |
| BP | GO:0090130 | tissue migration | 7/37 | 5.85E-06 | 8.71E-05 | 3.82E-05 | PTGS2/DPP4/KDR/PPARD/JUN/PRKCA/PPARG | 7 |
| BP | GO:1902110 | positive regulation of mitochondrial membrane permeability involved in apoptotic process | 4/37 | 5.88E-06 | 8.71E-05 | 3.82E-05 | BCL2/BAX/CASP8/TP53 | 4 |
| BP | GO:0043280 | positive regulation of cysteine-type endopeptidase activity involved in apoptotic process | 5/37 | 5.95E-06 | 8.72E-05 | 3.82E-05 | BAX/CASP9/CASP8/MYC/PPARG | 5 |
| BP | GO:0010948 | negative regulation of cell cycle process | 7/37 | 5.96E-06 | 8.72E-05 | 3.82E-05 | BCL2/BAX/CDKN1A/TP53/CDK1/PCNA/CCNB1 | 7 |
| BP | GO:0009755 | hormone-mediated signaling pathway | 6/37 | 6.26E-06 | 9.12E-05 | 3.99E-05 | AR/ESR2/PPARD/ESR1/PGR/PPARG | 6 |
| BP | GO:0006869 | lipid transport | 7/37 | 6.40E-06 | 9.27E-05 | 4.06E-05 | NOS2/NCOA2/PPARD/PON1/IL1B/PRKCD/PPARG | 7 |
| BP | GO:0031669 | cellular response to nutrient levels | 6/37 | 6.57E-06 | 9.46E-05 | 4.14E-05 | PTGS2/JUN/BCL2/CDKN1A/TP53/PPARG | 6 |
| BP | GO:0046824 | positive regulation of nucleocytoplasmic transport | 4/37 | 6.71E-06 | 9.55E-05 | 4.19E-05 | PTGS2/TP53/IL1B/PRKCD | 4 |
| BP | GO:1902686 | mitochondrial outer membrane permeabilization involved in programmed cell death | 4/37 | 6.71E-06 | 9.55E-05 | 4.19E-05 | BCL2/BAX/CASP8/TP53 | 4 |
| BP | GO:0060149 | negative regulation of posttranscriptional gene silencing | 3/37 | 6.79E-06 | 9.57E-05 | 4.19E-05 | ESR1/TP53/PPARG | 3 |
| BP | GO:0060967 | negative regulation of gene silencing by RNA | 3/37 | 6.79E-06 | 9.57E-05 | 4.19E-05 | ESR1/TP53/PPARG | 3 |
| BP | GO:0051402 | neuron apoptotic process | 6/37 | 6.89E-06 | 9.66E-05 | 4.23E-05 | JUN/BCL2/BAX/CASP9/CASP3/TP53 | 6 |
| BP | GO:0051098 | regulation of binding | 7/37 | 7.38E-06 | 0.000103 | 4.50E-05 | JUN/ADRB2/BCL2/BAX/PON1/PRKCD/PPARG | 7 |
| BP | GO:0035794 | positive regulation of mitochondrial membrane permeability | 4/37 | 7.62E-06 | 0.000106 | 4.63E-05 | BCL2/BAX/CASP8/TP53 | 4 |
| BP | GO:0097237 | cellular response to toxic substance | 6/37 | 8.32E-06 | 0.000115 | 5.03E-05 | PTGS2/KDR/PTGS1/CDK1/PCNA/PRKCD | 6 |
| BP | GO:1902108 | regulation of mitochondrial membrane permeability involved in apoptotic process | 4/37 | 8.62E-06 | 0.000116 | 5.10E-05 | BCL2/BAX/CASP8/TP53 | 4 |
| BP | GO:1905710 | positive regulation of membrane permeability | 4/37 | 8.62E-06 | 0.000116 | 5.10E-05 | BCL2/BAX/CASP8/TP53 | 4 |
| BP | GO:0006631 | fatty acid metabolic process | 7/37 | 8.76E-06 | 0.000116 | 5.10E-05 | PTGS2/PPARD/PTGS1/PON1/FASN/IL1B/PPARG | 7 |
| BP | GO:0006606 | protein import into nucleus | 5/37 | 8.79E-06 | 0.000116 | 5.10E-05 | PTGS2/DRD1/CDKN1A/TP53/PRKCD | 5 |
| BP | GO:0030879 | mammary gland development | 5/37 | 8.79E-06 | 0.000116 | 5.10E-05 | AR/ESR1/PGR/BAX/FASN | 5 |
| BP | GO:0035296 | regulation of tube diameter | 5/37 | 8.79E-06 | 0.000116 | 5.10E-05 | PTGS2/PPARD/DRD1/ADRB2/SLC6A4 | 5 |
| BP | GO:0050880 | regulation of blood vessel size | 5/37 | 8.79E-06 | 0.000116 | 5.10E-05 | PTGS2/PPARD/DRD1/ADRB2/SLC6A4 | 5 |
| BP | GO:0097746 | regulation of blood vessel diameter | 5/37 | 8.79E-06 | 0.000116 | 5.10E-05 | PTGS2/PPARD/DRD1/ADRB2/SLC6A4 | 5 |
| BP | GO:0035150 | regulation of tube size | 5/37 | 9.10E-06 | 0.00012 | 5.24E-05 | PTGS2/PPARD/DRD1/ADRB2/SLC6A4 | 5 |
| BP | GO:0042698 | ovulation cycle | 4/37 | 1.03E-05 | 0.000135 | 5.90E-05 | ESR1/PGR/CASP3/PCNA | 4 |
| BP | GO:0061041 | regulation of wound healing | 5/37 | 1.04E-05 | 0.000135 | 5.93E-05 | F7/PPARD/PRKCA/CDKN1A/PRKCD | 5 |
| BP | GO:0014074 | response to purine-containing compound | 5/37 | 1.07E-05 | 0.000139 | 6.07E-05 | PTGS2/JUN/SLC6A4/IL1B/PPARG | 5 |
| BP | GO:2001056 | positive regulation of cysteine-type endopeptidase activity | 5/37 | 1.07E-05 | 0.000139 | 6.07E-05 | BAX/CASP9/CASP8/MYC/PPARG | 5 |
| BP | GO:0048754 | branching morphogenesis of an epithelial tube | 5/37 | 1.11E-05 | 0.000142 | 6.24E-05 | AR/ESR1/PGR/BCL2/MYC | 5 |
| BP | GO:0010876 | lipid localization | 7/37 | 1.16E-05 | 0.000148 | 6.48E-05 | NOS2/NCOA2/PPARD/PON1/IL1B/PRKCD/PPARG | 7 |
| BP | GO:0034504 | protein localization to nucleus | 6/37 | 1.16E-05 | 0.000148 | 6.48E-05 | PTGS2/DRD1/CDKN1A/TP53/CDK1/PRKCD | 6 |
| BP | GO:0050805 | negative regulation of synaptic transmission | 4/37 | 1.22E-05 | 0.000153 | 6.72E-05 | PTGS2/DRD1/SLC6A4/IL1B | 4 |
| BP | GO:0061180 | mammary gland epithelium development | 4/37 | 1.22E-05 | 0.000153 | 6.72E-05 | AR/ESR1/PGR/BAX | 4 |
| BP | GO:0031668 | cellular response to extracellular stimulus | 6/37 | 1.32E-05 | 0.000166 | 7.26E-05 | PTGS2/JUN/BCL2/CDKN1A/TP53/PPARG | 6 |
| BP | GO:0019216 | regulation of lipid metabolic process | 7/37 | 1.36E-05 | 0.00017 | 7.45E-05 | PTGS2/NCOA2/PPARD/FASN/IL1B/PRKCD/PPARG | 7 |
| BP | GO:0007548 | sex differentiation | 6/37 | 1.38E-05 | 0.000171 | 7.48E-05 | AR/ESR1/PGR/BCL2/BAX/CASP3 | 6 |
| BP | GO:1905952 | regulation of lipid localization | 5/37 | 1.38E-05 | 0.000171 | 7.48E-05 | PPARD/PON1/IL1B/PRKCD/PPARG | 5 |
| BP | GO:0046902 | regulation of mitochondrial membrane permeability | 4/37 | 1.51E-05 | 0.000186 | 8.14E-05 | BCL2/BAX/CASP8/TP53 | 4 |
| BP | GO:0043270 | positive regulation of ion transport | 6/37 | 1.53E-05 | 0.000187 | 8.21E-05 | DRD1/ADRB2/SLC6A4/BAX/IL1B/PRKCD | 6 |
| BP | GO:0048678 | response to axon injury | 4/37 | 1.59E-05 | 0.000194 | 8.49E-05 | JUN/BCL2/BAX/CDK1 | 4 |
| BP | GO:0060444 | branching involved in mammary gland duct morphogenesis | 3/37 | 1.60E-05 | 0.000194 | 8.49E-05 | AR/ESR1/PGR | 3 |
| BP | GO:0015718 | monocarboxylic acid transport | 5/37 | 1.61E-05 | 0.000194 | 8.51E-05 | NOS2/NCOA2/PPARD/IL1B/PPARG | 5 |
| BP | GO:0051170 | import into nucleus | 5/37 | 1.66E-05 | 0.000199 | 8.72E-05 | PTGS2/DRD1/CDKN1A/TP53/PRKCD | 5 |
| BP | GO:0072332 | intrinsic apoptotic signaling pathway by p53 class mediator | 4/37 | 1.68E-05 | 0.0002 | 8.77E-05 | BCL2/BAX/CDKN1A/TP53 | 4 |
| BP | GO:0052548 | regulation of endopeptidase activity | 7/37 | 1.72E-05 | 0.000205 | 8.96E-05 | PTGS2/BAX/CASP9/CASP8/MYC/DPEP1/PPARG | 7 |
| BP | GO:0016579 | protein deubiquitination | 6/37 | 1.80E-05 | 0.000212 | 9.28E-05 | AR/ESR1/ADRB2/TP53/CDK1/MYC | 6 |
| BP | GO:1900739 | regulation of protein insertion into mitochondrial membrane involved in apoptotic signaling pathway | 3/37 | 1.81E-05 | 0.000212 | 9.28E-05 | BCL2/CASP8/TP53 | 3 |
| BP | GO:1900740 | positive regulation of protein insertion into mitochondrial membrane involved in apoptotic signaling pathway | 3/37 | 1.81E-05 | 0.000212 | 9.28E-05 | BCL2/CASP8/TP53 | 3 |
| BP | GO:0051348 | negative regulation of transferase activity | 6/37 | 1.87E-05 | 0.000219 | 9.59E-05 | CASP3/CDKN1A/TP53/IL1B/PRKCD/PPARG | 6 |
| BP | GO:0034614 | cellular response to reactive oxygen species | 5/37 | 1.92E-05 | 0.000223 | 9.77E-05 | JUN/CDK1/PCNA/PRKCD/DPEP1 | 5 |
| BP | GO:0009651 | response to salt stress | 3/37 | 2.03E-05 | 0.000234 | 0.000102 | AKR1B1/BAX/TP53 | 3 |
| BP | GO:2000144 | positive regulation of DNA-templated transcription, initiation | 3/37 | 2.03E-05 | 0.000234 | 0.000102 | JUN/ESR1/TP53 | 3 |
| BP | GO:0060249 | anatomical structure homeostasis | 7/37 | 2.06E-05 | 0.000236 | 0.000103 | AKR1B1/ADRB2/BCL2/BAX/PRKCA/PCNA/MYC | 7 |
| BP | GO:0003018 | vascular process in circulatory system | 5/37 | 2.21E-05 | 0.000252 | 0.000111 | PTGS2/PPARD/DRD1/ADRB2/SLC6A4 | 5 |
| BP | GO:0001516 | prostaglandin biosynthetic process | 3/37 | 2.27E-05 | 0.000257 | 0.000113 | PTGS2/PTGS1/IL1B | 3 |
| BP | GO:0046457 | prostanoid biosynthetic process | 3/37 | 2.27E-05 | 0.000257 | 0.000113 | PTGS2/PTGS1/IL1B | 3 |
| BP | GO:1901990 | regulation of mitotic cell cycle phase transition | 7/37 | 2.28E-05 | 0.000257 | 0.000113 | BCL2/BAX/CDKN1A/TP53/CDK1/PCNA/CCNB1 | 7 |
| BP | GO:0050796 | regulation of insulin secretion | 5/37 | 2.40E-05 | 0.000269 | 0.000118 | NOS2/DPP4/PPARD/PRKCA/IL1B | 5 |
| BP | GO:0070646 | protein modification by small protein removal | 6/37 | 2.46E-05 | 0.000271 | 0.000119 | AR/ESR1/ADRB2/TP53/CDK1/MYC | 6 |
| BP | GO:0048469 | cell maturation | 5/37 | 2.47E-05 | 0.000271 | 0.000119 | PGR/BCL2/CDKN1A/CCNB1/PPARG | 5 |
| BP | GO:0006919 | activation of cysteine-type endopeptidase activity involved in apoptotic process | 4/37 | 2.47E-05 | 0.000271 | 0.000119 | BAX/CASP9/CASP8/PPARG | 4 |
| BP | GO:0070542 | response to fatty acid | 4/37 | 2.47E-05 | 0.000271 | 0.000119 | PTGS2/PON1/CCNB1/PPARG | 4 |
| BP | GO:0090559 | regulation of membrane permeability | 4/37 | 2.47E-05 | 0.000271 | 0.000119 | BCL2/BAX/CASP8/TP53 | 4 |
| BP | GO:0010950 | positive regulation of endopeptidase activity | 5/37 | 2.53E-05 | 0.000277 | 0.000121 | BAX/CASP9/CASP8/MYC/PPARG | 5 |
| BP | GO:0052547 | regulation of peptidase activity | 7/37 | 2.55E-05 | 0.000278 | 0.000122 | PTGS2/BAX/CASP9/CASP8/MYC/DPEP1/PPARG | 7 |
| BP | GO:1903034 | regulation of response to wounding | 5/37 | 2.60E-05 | 0.000281 | 0.000123 | F7/PPARD/PRKCA/CDKN1A/PRKCD | 5 |
| BP | GO:2001235 | positive regulation of apoptotic signaling pathway | 5/37 | 2.60E-05 | 0.000281 | 0.000123 | BCL2/BAX/CASP8/TP53/PRKCD | 5 |
| BP | GO:0016570 | histone modification | 7/37 | 2.63E-05 | 0.000283 | 0.000124 | DRD1/PRKCA/TP53/CDK1/IL1B/PRKCD/CCNB1 | 7 |
| BP | GO:0097194 | execution phase of apoptosis | 4/37 | 2.70E-05 | 0.000288 | 0.000126 | BAX/CASP3/CASP8/TP53 | 4 |
| BP | GO:1904951 | positive regulation of establishment of protein localization | 7/37 | 2.70E-05 | 0.000288 | 0.000126 | PTGS2/PPARD/BCL2/CASP8/TP53/IL1B/PRKCD | 7 |
| BP | GO:0001782 | B cell homeostasis | 3/37 | 2.80E-05 | 0.000297 | 0.00013 | BCL2/BAX/CASP3 | 3 |
| BP | GO:0010821 | regulation of mitochondrion organization | 5/37 | 2.82E-05 | 0.000297 | 0.00013 | KDR/BCL2/BAX/CASP8/TP53 | 5 |
| BP | GO:0061138 | morphogenesis of a branching epithelium | 5/37 | 2.82E-05 | 0.000297 | 0.00013 | AR/ESR1/PGR/BCL2/MYC | 5 |
| BP | GO:0001667 | ameboidal-type cell migration | 7/37 | 2.90E-05 | 0.000304 | 0.000133 | PTGS2/DPP4/KDR/PPARD/JUN/PRKCA/PPARG | 7 |
| BP | GO:0023061 | signal release | 7/37 | 2.94E-05 | 0.000307 | 0.000135 | NOS2/DPP4/PPARD/DRD1/CHRM2/PRKCA/IL1B | 7 |
| BP | GO:0042110 | T cell activation | 7/37 | 3.02E-05 | 0.000315 | 0.000138 | DPP4/BCL2/BAX/CASP3/CASP8/TP53/IL1B | 7 |
| BP | GO:0001963 | synaptic transmission, dopaminergic | 3/37 | 3.10E-05 | 0.000319 | 0.00014 | PTGS2/DRD1/SLC6A4 | 3 |
| BP | GO:0010165 | response to X-ray | 3/37 | 3.10E-05 | 0.000319 | 0.00014 | CASP3/CDKN1A/TP53 | 3 |
| BP | GO:0043457 | regulation of cellular respiration | 3/37 | 3.10E-05 | 0.000319 | 0.00014 | NOS2/CDK1/CCNB1 | 3 |
| BP | GO:0033273 | response to vitamin | 4/37 | 3.36E-05 | 0.000343 | 0.00015 | PTGS2/F7/PPARD/PPARG | 4 |
| BP | GO:1901655 | cellular response to ketone | 4/37 | 3.36E-05 | 0.000343 | 0.00015 | AR/AKR1B1/CASP9/PPARG | 4 |
| BP | GO:0016569 | covalent chromatin modification | 7/37 | 3.46E-05 | 0.000352 | 0.000154 | DRD1/PRKCA/TP53/CDK1/IL1B/PRKCD/CCNB1 | 7 |
| BP | GO:0042594 | response to starvation | 5/37 | 3.55E-05 | 0.00036 | 0.000158 | JUN/BCL2/CDKN1A/TP53/PPARG | 5 |
| BP | GO:0060562 | epithelial tube morphogenesis | 6/37 | 3.72E-05 | 0.000375 | 0.000164 | AR/ESR1/PGR/BCL2/CASP3/MYC | 6 |
| BP | GO:1901987 | regulation of cell cycle phase transition | 7/37 | 3.75E-05 | 0.000376 | 0.000165 | BCL2/BAX/CDKN1A/TP53/CDK1/PCNA/CCNB1 | 7 |
| BP | GO:0060603 | mammary gland duct morphogenesis | 3/37 | 3.75E-05 | 0.000376 | 0.000165 | AR/ESR1/PGR | 3 |
| BP | GO:0015908 | fatty acid transport | 4/37 | 3.96E-05 | 0.000395 | 0.000173 | NOS2/PPARD/IL1B/PPARG | 4 |
| BP | GO:0050727 | regulation of inflammatory response | 7/37 | 4.00E-05 | 0.000396 | 0.000174 | NOS2/PTGS2/PPARD/ESR1/IL1B/PRKCD/PPARG | 7 |
| BP | GO:0001763 | morphogenesis of a branching structure | 5/37 | 4.02E-05 | 0.000396 | 0.000174 | AR/ESR1/PGR/BCL2/MYC | 5 |
| BP | GO:0071897 | DNA biosynthetic process | 5/37 | 4.02E-05 | 0.000396 | 0.000174 | CDKN1A/TP53/PCNA/MYC/PPARG | 5 |
| BP | GO:0010952 | positive regulation of peptidase activity | 5/37 | 4.12E-05 | 0.000405 | 0.000177 | BAX/CASP9/CASP8/MYC/PPARG | 5 |
| BP | GO:1901570 | fatty acid derivative biosynthetic process | 4/37 | 4.29E-05 | 0.00042 | 0.000184 | PTGS2/PTGS1/FASN/IL1B | 4 |
| BP | GO:0017038 | protein import | 5/37 | 4.32E-05 | 0.000421 | 0.000185 | PTGS2/DRD1/CDKN1A/TP53/PRKCD | 5 |
| BP | GO:0008202 | steroid metabolic process | 6/37 | 4.34E-05 | 0.000421 | 0.000185 | PPARD/AKR1B1/ESR1/PON1/FASN/IL1B | 6 |
| BP | GO:0045740 | positive regulation of DNA replication | 3/37 | 4.89E-05 | 0.000473 | 0.000207 | JUN/CDK1/PCNA | 3 |
| BP | GO:0050679 | positive regulation of epithelial cell proliferation | 5/37 | 5.09E-05 | 0.000491 | 0.000215 | AR/KDR/JUN/PRKCA/MYC | 5 |
| BP | GO:0046822 | regulation of nucleocytoplasmic transport | 4/37 | 5.21E-05 | 0.000495 | 0.000217 | PTGS2/TP53/IL1B/PRKCD | 4 |
| BP | GO:0071887 | leukocyte apoptotic process | 4/37 | 5.21E-05 | 0.000495 | 0.000217 | BAX/CASP9/CASP3/TP53 | 4 |
| BP | GO:2001237 | negative regulation of extrinsic apoptotic signaling pathway | 4/37 | 5.21E-05 | 0.000495 | 0.000217 | AR/BCL2/CASP8/IL1B | 4 |
| BP | GO:0030073 | insulin secretion | 5/37 | 5.21E-05 | 0.000495 | 0.000217 | NOS2/DPP4/PPARD/PRKCA/IL1B | 5 |
| BP | GO:0006913 | nucleocytoplasmic transport | 6/37 | 5.29E-05 | 0.000496 | 0.000217 | PTGS2/DRD1/CDKN1A/TP53/IL1B/PRKCD | 6 |
| BP | GO:0001662 | behavioral fear response | 3/37 | 5.31E-05 | 0.000496 | 0.000217 | DPP4/DRD1/BCL2 | 3 |
| BP | GO:0043029 | T cell homeostasis | 3/37 | 5.31E-05 | 0.000496 | 0.000217 | BCL2/BAX/CASP3 | 3 |
| BP | GO:0045923 | positive regulation of fatty acid metabolic process | 3/37 | 5.31E-05 | 0.000496 | 0.000217 | PTGS2/IL1B/PPARG | 3 |
| BP | GO:2000142 | regulation of DNA-templated transcription, initiation | 3/37 | 5.31E-05 | 0.000496 | 0.000217 | JUN/ESR1/TP53 | 3 |
| BP | GO:0090276 | regulation of peptide hormone secretion | 5/37 | 5.33E-05 | 0.000496 | 0.000217 | NOS2/DPP4/PPARD/PRKCA/IL1B | 5 |
| BP | GO:0051169 | nuclear transport | 6/37 | 5.55E-05 | 0.000514 | 0.000225 | PTGS2/DRD1/CDKN1A/TP53/IL1B/PRKCD | 6 |
| BP | GO:0042136 | neurotransmitter biosynthetic process | 4/37 | 5.61E-05 | 0.000518 | 0.000227 | NOS2/PTGS2/SLC6A4/IL1B | 4 |
| BP | GO:0002209 | behavioral defense response | 3/37 | 5.76E-05 | 0.00053 | 0.000232 | DPP4/DRD1/BCL2 | 3 |
| BP | GO:0006275 | regulation of DNA replication | 4/37 | 6.04E-05 | 0.000551 | 0.000241 | JUN/TP53/CDK1/PCNA | 4 |
| BP | GO:2000278 | regulation of DNA biosynthetic process | 4/37 | 6.04E-05 | 0.000551 | 0.000241 | CDKN1A/TP53/MYC/PPARG | 4 |
| BP | GO:0060969 | negative regulation of gene silencing | 3/37 | 6.23E-05 | 0.000567 | 0.000248 | ESR1/TP53/PPARG | 3 |
| BP | GO:0001505 | regulation of neurotransmitter levels | 6/37 | 6.30E-05 | 0.000571 | 0.00025 | NOS2/PTGS2/DRD1/CHRM2/SLC6A4/IL1B | 6 |
| BP | GO:0006939 | smooth muscle contraction | 4/37 | 6.49E-05 | 0.000586 | 0.000257 | PTGS2/DRD1/CHRM2/ADRB2 | 4 |
| BP | GO:0042596 | fear response | 3/37 | 6.73E-05 | 0.000604 | 0.000264 | DPP4/DRD1/BCL2 | 3 |
| BP | GO:0071470 | cellular response to osmotic stress | 3/37 | 6.73E-05 | 0.000604 | 0.000264 | PTGS2/AKR1B1/CASP3 | 3 |
| BP | GO:0045862 | positive regulation of proteolysis | 6/37 | 7.24E-05 | 0.000646 | 0.000283 | BAX/CASP9/CASP8/MYC/IL1B/PPARG | 6 |
| BP | GO:1902893 | regulation of pri-miRNA transcription by RNA polymerase II | 3/37 | 7.25E-05 | 0.000646 | 0.000283 | PPARD/JUN/TP53 | 3 |
| BP | GO:0001894 | tissue homeostasis | 5/37 | 8.07E-05 | 0.000717 | 0.000314 | AKR1B1/ADRB2/BCL2/BAX/PRKCA | 5 |
| BP | GO:0002761 | regulation of myeloid leukocyte differentiation | 4/37 | 8.25E-05 | 0.00073 | 0.00032 | JUN/CASP8/PRKCA/MYC | 4 |
| BP | GO:0006692 | prostanoid metabolic process | 3/37 | 8.37E-05 | 0.000736 | 0.000322 | PTGS2/PTGS1/IL1B | 3 |
| BP | GO:0006693 | prostaglandin metabolic process | 3/37 | 8.37E-05 | 0.000736 | 0.000322 | PTGS2/PTGS1/IL1B | 3 |
| BP | GO:0097305 | response to alcohol | 5/37 | 9.13E-05 | 0.000799 | 0.00035 | F7/CASP8/CDKN1A/CDK1/PPARG | 5 |
| BP | GO:0042771 | intrinsic apoptotic signaling pathway in response to DNA damage by p53 class mediator | 3/37 | 9.60E-05 | 0.000838 | 0.000367 | BCL2/CDKN1A/TP53 | 3 |
| BP | GO:0071216 | cellular response to biotic stimulus | 5/37 | 9.69E-05 | 0.000843 | 0.000369 | NOS2/PPARD/PRKCA/TP53/IL1B | 5 |
| BP | GO:1901653 | cellular response to peptide | 6/37 | 0.0001 | 0.000868 | 0.00038 | AKR1B1/ADRB2/TP53/IL1B/PRKCD/PPARG | 6 |
| BP | GO:0022602 | ovulation cycle process | 3/37 | 0.000103 | 0.000886 | 0.000388 | ESR1/PGR/CASP3 | 3 |
| BP | GO:0048806 | genitalia development | 3/37 | 0.000109 | 0.000942 | 0.000413 | AR/ESR1/BAX | 3 |
| BP | GO:0018107 | peptidyl-threonine phosphorylation | 4/37 | 0.00011 | 0.000944 | 0.000413 | BCL2/PRKCA/CDK1/PRKCD | 4 |
| BP | GO:0009266 | response to temperature stimulus | 5/37 | 0.000111 | 0.000952 | 0.000417 | PTGS2/ADRB2/CASP8/CDKN1A/PPARG | 5 |
| BP | GO:0032368 | regulation of lipid transport | 4/37 | 0.000113 | 0.000967 | 0.000423 | PON1/IL1B/PRKCD/PPARG | 4 |
| BP | GO:0051972 | regulation of telomerase activity | 3/37 | 0.000117 | 0.000991 | 0.000434 | TP53/MYC/PPARG | 3 |
| BP | GO:0097300 | programmed necrotic cell death | 3/37 | 0.000124 | 0.00105 | 0.00046 | BAX/CASP8/TP53 | 3 |
| BP | GO:0030072 | peptide hormone secretion | 5/37 | 0.000127 | 0.001073 | 0.00047 | NOS2/DPP4/PPARD/PRKCA/IL1B | 5 |
| BP | GO:0030518 | intracellular steroid hormone receptor signaling pathway | 4/37 | 0.000132 | 0.001102 | 0.000483 | AR/ESR2/ESR1/PGR | 4 |
| BP | GO:0002931 | response to ischemia | 3/37 | 0.000132 | 0.001102 | 0.000483 | BCL2/CASP9/TP53 | 3 |
| BP | GO:0009409 | response to cold | 3/37 | 0.000132 | 0.001102 | 0.000483 | ADRB2/CASP8/PPARG | 3 |
| BP | GO:2000027 | regulation of animal organ morphogenesis | 5/37 | 0.000134 | 0.00112 | 0.000491 | AR/ESR1/BCL2/BAX/MYC | 5 |
| BP | GO:0007006 | mitochondrial membrane organization | 4/37 | 0.000139 | 0.00115 | 0.000504 | BCL2/BAX/CASP8/TP53 | 4 |
| BP | GO:0018210 | peptidyl-threonine modification | 4/37 | 0.000139 | 0.00115 | 0.000504 | BCL2/PRKCA/CDK1/PRKCD | 4 |
| BP | GO:0046683 | response to organophosphorus | 4/37 | 0.000139 | 0.00115 | 0.000504 | PTGS2/JUN/SLC6A4/IL1B | 4 |
| BP | GO:0007566 | embryo implantation | 3/37 | 0.00014 | 0.00115 | 0.000504 | PTGS2/PPARD/IL1B | 3 |
| BP | GO:0007611 | learning or memory | 5/37 | 0.000142 | 0.001165 | 0.00051 | PTGS2/JUN/DRD1/SLC6A4/CASP3 | 5 |
| BP | GO:0007292 | female gamete generation | 4/37 | 0.000148 | 0.001208 | 0.000529 | PTGS2/PGR/BCL2/CCNB1 | 4 |
| BP | GO:0006998 | nuclear envelope organization | 3/37 | 0.000148 | 0.001208 | 0.000529 | PRKCA/CDK1/CCNB1 | 3 |
| BP | GO:0072655 | establishment of protein localization to mitochondrion | 4/37 | 0.000152 | 0.001234 | 0.000541 | BCL2/BAX/CASP8/TP53 | 4 |
| BP | GO:0001558 | regulation of cell growth | 6/37 | 0.000153 | 0.001238 | 0.000542 | ESR2/PPARD/BCL2/CDKN1A/TP53/PPARG | 6 |
| BP | GO:0051091 | positive regulation of DNA-binding transcription factor activity | 5/37 | 0.000155 | 0.001255 | 0.00055 | AR/ESR2/ESR1/IL1B/PPARG | 5 |
| BP | GO:0008584 | male gonad development | 4/37 | 0.000156 | 0.001258 | 0.000551 | AR/ESR1/BCL2/BAX | 4 |
| BP | GO:0015850 | organic hydroxy compound transport | 5/37 | 0.000158 | 0.00127 | 0.000556 | NCOA2/DRD1/SLC6A4/PON1/PPARG | 5 |
| BP | GO:0046546 | development of primary male sexual characteristics | 4/37 | 0.000161 | 0.001285 | 0.000563 | AR/ESR1/BCL2/BAX | 4 |
| BP | GO:0002763 | positive regulation of myeloid leukocyte differentiation | 3/37 | 0.000166 | 0.001318 | 0.000577 | JUN/CASP8/PRKCA | 3 |
| BP | GO:0006636 | unsaturated fatty acid biosynthetic process | 3/37 | 0.000166 | 0.001318 | 0.000577 | PTGS2/PTGS1/IL1B | 3 |
| BP | GO:0032386 | regulation of intracellular transport | 6/37 | 0.000167 | 0.001318 | 0.000577 | PTGS2/DRD1/CHRM2/TP53/IL1B/PRKCD | 6 |
| BP | GO:0070585 | protein localization to mitochondrion | 4/37 | 0.00017 | 0.001318 | 0.000577 | BCL2/BAX/CASP8/TP53 | 4 |
| BP | GO:0046883 | regulation of hormone secretion | 5/37 | 0.00017 | 0.001318 | 0.000577 | NOS2/DPP4/PPARD/PRKCA/IL1B | 5 |
| BP | GO:0001660 | fever generation | 2/37 | 0.00017 | 0.001318 | 0.000577 | PTGS2/IL1B | 2 |
| BP | GO:0032070 | regulation of deoxyribonuclease activity | 2/37 | 0.00017 | 0.001318 | 0.000577 | PCNA/PRKCD | 2 |
| BP | GO:0034350 | regulation of glial cell apoptotic process | 2/37 | 0.00017 | 0.001318 | 0.000577 | PRKCA/PRKCD | 2 |
| BP | GO:0060068 | vagina development | 2/37 | 0.00017 | 0.001318 | 0.000577 | ESR1/BAX | 2 |
| BP | GO:1903799 | negative regulation of production of miRNAs involved in gene silencing by miRNA | 2/37 | 0.00017 | 0.001318 | 0.000577 | ESR1/TP53 | 2 |
| BP | GO:1903862 | positive regulation of oxidative phosphorylation | 2/37 | 0.00017 | 0.001318 | 0.000577 | CDK1/CCNB1 | 2 |
| BP | GO:1900542 | regulation of purine nucleotide metabolic process | 4/37 | 0.000174 | 0.001343 | 0.000588 | NOS2/TP53/CDK1/CCNB1 | 4 |
| BP | GO:0001541 | ovarian follicle development | 3/37 | 0.000175 | 0.001343 | 0.000588 | ESR1/BCL2/BAX | 3 |
| BP | GO:0046456 | icosanoid biosynthetic process | 3/37 | 0.000175 | 0.001343 | 0.000588 | PTGS2/PTGS1/IL1B | 3 |
| BP | GO:0045165 | cell fate commitment | 5/37 | 0.000182 | 0.001392 | 0.00061 | AR/BCL2/CASP3/TP53/PPARG | 5 |
| BP | GO:0051090 | regulation of DNA-binding transcription factor activity | 6/37 | 0.000188 | 0.001431 | 0.000627 | AR/ESR2/JUN/ESR1/IL1B/PPARG | 6 |
| BP | GO:0071901 | negative regulation of protein serine/threonine kinase activity | 4/37 | 0.000189 | 0.001436 | 0.000629 | CASP3/CDKN1A/IL1B/PRKCD | 4 |
| BP | GO:0043542 | endothelial cell migration | 5/37 | 0.000192 | 0.001451 | 0.000636 | PTGS2/DPP4/KDR/PRKCA/PPARG | 5 |
| BP | GO:0042391 | regulation of membrane potential | 6/37 | 0.000192 | 0.001451 | 0.000636 | KDR/JUN/DRD1/ADRB2/BCL2/BAX | 6 |
| BP | GO:0006140 | regulation of nucleotide metabolic process | 4/37 | 0.000194 | 0.001451 | 0.000636 | NOS2/TP53/CDK1/CCNB1 | 4 |
| BP | GO:0035264 | multicellular organism growth | 4/37 | 0.000194 | 0.001451 | 0.000636 | AR/ADRB2/BCL2/TP53 | 4 |
| BP | GO:0045834 | positive regulation of lipid metabolic process | 4/37 | 0.000194 | 0.001451 | 0.000636 | PTGS2/IL1B/PRKCD/PPARG | 4 |
| BP | GO:0045981 | positive regulation of nucleotide metabolic process | 3/37 | 0.000195 | 0.001451 | 0.000636 | NOS2/CDK1/CCNB1 | 3 |
| BP | GO:1900544 | positive regulation of purine nucleotide metabolic process | 3/37 | 0.000195 | 0.001451 | 0.000636 | NOS2/CDK1/CCNB1 | 3 |
| BP | GO:0050804 | modulation of chemical synaptic transmission | 6/37 | 0.000197 | 0.001465 | 0.000642 | PTGS2/DRD1/CHRM2/ADRB2/SLC6A4/IL1B | 6 |
| BP | GO:0071236 | cellular response to antibiotic | 4/37 | 0.000199 | 0.001475 | 0.000646 | TP53/CDK1/PCNA/PRKCD | 4 |
| BP | GO:0099177 | regulation of trans-synaptic signaling | 6/37 | 0.0002 | 0.001475 | 0.000646 | PTGS2/DRD1/CHRM2/ADRB2/SLC6A4/IL1B | 6 |
| BP | GO:0030520 | intracellular estrogen receptor signaling pathway | 3/37 | 0.000205 | 0.001493 | 0.000654 | AR/ESR2/ESR1 | 3 |
| BP | GO:1903749 | positive regulation of establishment of protein localization to mitochondrion | 3/37 | 0.000205 | 0.001493 | 0.000654 | BCL2/CASP8/TP53 | 3 |
| BP | GO:0019371 | cyclooxygenase pathway | 2/37 | 0.000208 | 0.001493 | 0.000654 | PTGS2/PTGS1 | 2 |
| BP | GO:0031652 | positive regulation of heat generation | 2/37 | 0.000208 | 0.001493 | 0.000654 | PTGS2/IL1B | 2 |
| BP | GO:0035404 | histone-serine phosphorylation | 2/37 | 0.000208 | 0.001493 | 0.000654 | DRD1/CCNB1 | 2 |
| BP | GO:0045899 | positive regulation of RNA polymerase II transcriptional preinitiation complex assembly | 2/37 | 0.000208 | 0.001493 | 0.000654 | ESR1/TP53 | 2 |
| BP | GO:0051974 | negative regulation of telomerase activity | 2/37 | 0.000208 | 0.001493 | 0.000654 | TP53/PPARG | 2 |
| BP | GO:0055015 | ventricular cardiac muscle cell development | 2/37 | 0.000208 | 0.001493 | 0.000654 | CDK1/CCNB1 | 2 |
| BP | GO:0060736 | prostate gland growth | 2/37 | 0.000208 | 0.001493 | 0.000654 | AR/ESR1 | 2 |
| BP | GO:2001269 | positive regulation of cysteine-type endopeptidase activity involved in apoptotic signaling pathway | 2/37 | 0.000208 | 0.001493 | 0.000654 | BAX/CASP8 | 2 |
| BP | GO:0001822 | kidney development | 5/37 | 0.000208 | 0.001493 | 0.000654 | AKR1B1/BCL2/BAX/CASP9/MYC | 5 |
| BP | GO:0009267 | cellular response to starvation | 4/37 | 0.00021 | 0.001498 | 0.000656 | JUN/BCL2/CDKN1A/TP53 | 4 |
| BP | GO:0097755 | positive regulation of blood vessel diameter | 3/37 | 0.000216 | 0.001537 | 0.000673 | PPARD/DRD1/ADRB2 | 3 |
| BP | GO:0001890 | placenta development | 4/37 | 0.000226 | 0.001603 | 0.000702 | PTGS2/PPARD/CASP8/PPARG | 4 |
| BP | GO:0032409 | regulation of transporter activity | 5/37 | 0.000226 | 0.001603 | 0.000702 | ADRB2/BCL2/PON1/PRKCD/PPARG | 5 |
| BP | GO:0021700 | developmental maturation | 5/37 | 0.00023 | 0.001621 | 0.00071 | PGR/BCL2/CDKN1A/CCNB1/PPARG | 5 |
| BP | GO:0031647 | regulation of protein stability | 5/37 | 0.00023 | 0.001621 | 0.00071 | BCL2/CASP3/CDKN1A/TP53/PRKCD | 5 |
| BP | GO:0042133 | neurotransmitter metabolic process | 4/37 | 0.000232 | 0.001626 | 0.000712 | NOS2/PTGS2/SLC6A4/IL1B | 4 |
| BP | GO:0090316 | positive regulation of intracellular protein transport | 4/37 | 0.000232 | 0.001626 | 0.000712 | PTGS2/TP53/IL1B/PRKCD | 4 |
| BP | GO:0015980 | energy derivation by oxidation of organic compounds | 5/37 | 0.000234 | 0.001634 | 0.000716 | NOS2/TP53/CDK1/MYC/CCNB1 | 5 |
| BP | GO:0030888 | regulation of B cell proliferation | 3/37 | 0.000238 | 0.001652 | 0.000723 | BCL2/CASP3/CDKN1A | 3 |
| BP | GO:0032370 | positive regulation of lipid transport | 3/37 | 0.000238 | 0.001652 | 0.000723 | PON1/IL1B/PRKCD | 3 |
| BP | GO:2001244 | positive regulation of intrinsic apoptotic signaling pathway | 3/37 | 0.000238 | 0.001652 | 0.000723 | BCL2/BAX/TP53 | 3 |
| BP | GO:2001236 | regulation of extrinsic apoptotic signaling pathway | 4/37 | 0.000244 | 0.001686 | 0.000738 | AR/BCL2/CASP8/IL1B | 4 |
| BP | GO:0031392 | regulation of prostaglandin biosynthetic process | 2/37 | 0.000249 | 0.001706 | 0.000747 | PTGS2/IL1B | 2 |
| BP | GO:0043535 | regulation of blood vessel endothelial cell migration | 4/37 | 0.00025 | 0.001706 | 0.000747 | PTGS2/KDR/PRKCA/PPARG | 4 |
| BP | GO:0002260 | lymphocyte homeostasis | 3/37 | 0.00025 | 0.001706 | 0.000747 | BCL2/BAX/CASP3 | 3 |
| BP | GO:0070059 | intrinsic apoptotic signaling pathway in response to endoplasmic reticulum stress | 3/37 | 0.00025 | 0.001706 | 0.000747 | BCL2/BAX/TP53 | 3 |
| BP | GO:0070265 | necrotic cell death | 3/37 | 0.00025 | 0.001706 | 0.000747 | BAX/CASP8/TP53 | 3 |
| BP | GO:0010632 | regulation of epithelial cell migration | 5/37 | 0.000257 | 0.001752 | 0.000767 | PTGS2/KDR/JUN/PRKCA/PPARG | 5 |
| BP | GO:0046686 | response to cadmium ion | 3/37 | 0.000262 | 0.001776 | 0.000778 | JUN/CDK1/PCNA | 3 |
| BP | GO:0043467 | regulation of generation of precursor metabolites and energy | 4/37 | 0.000262 | 0.001776 | 0.000778 | NOS2/TP53/CDK1/CCNB1 | 4 |
| BP | GO:0072001 | renal system development | 5/37 | 0.000266 | 0.001794 | 0.000786 | AKR1B1/BCL2/BAX/CASP9/MYC | 5 |
| BP | GO:0045600 | positive regulation of fat cell differentiation | 3/37 | 0.000275 | 0.001849 | 0.00081 | PTGS2/PPARD/PPARG | 3 |
| BP | GO:0046661 | male sex differentiation | 4/37 | 0.000275 | 0.001849 | 0.00081 | AR/ESR1/BCL2/BAX | 4 |
| BP | GO:0050890 | cognition | 5/37 | 0.000279 | 0.001864 | 0.000817 | PTGS2/JUN/DRD1/SLC6A4/CASP3 | 5 |
| BP | GO:0003012 | muscle system process | 6/37 | 0.000279 | 0.001864 | 0.000817 | PTGS2/DRD1/CHRM2/ADRB2/PRKCA/IL1B | 6 |
| BP | GO:0006940 | regulation of smooth muscle contraction | 3/37 | 0.000288 | 0.001917 | 0.00084 | PTGS2/CHRM2/ADRB2 | 3 |
| BP | GO:0031650 | regulation of heat generation | 2/37 | 0.000294 | 0.001945 | 0.000852 | PTGS2/IL1B | 2 |
| BP | GO:0033127 | regulation of histone phosphorylation | 2/37 | 0.000294 | 0.001945 | 0.000852 | IL1B/CCNB1 | 2 |
| BP | GO:0090399 | replicative senescence | 2/37 | 0.000294 | 0.001945 | 0.000852 | CDKN1A/TP53 | 2 |
| BP | GO:0045931 | positive regulation of mitotic cell cycle | 4/37 | 0.000295 | 0.001949 | 0.000854 | PRKCA/CDK1/IL1B/CCNB1 | 4 |
| BP | GO:0006633 | fatty acid biosynthetic process | 4/37 | 0.000302 | 0.00199 | 0.000872 | PTGS2/PTGS1/FASN/IL1B | 4 |
| BP | GO:0071695 | anatomical structure maturation | 4/37 | 0.000309 | 0.002026 | 0.000888 | PGR/BCL2/CDKN1A/CCNB1 | 4 |
| BP | GO:2001242 | regulation of intrinsic apoptotic signaling pathway | 4/37 | 0.000309 | 0.002026 | 0.000888 | PTGS2/BCL2/BAX/TP53 | 4 |
| BP | GO:0048662 | negative regulation of smooth muscle cell proliferation | 3/37 | 0.000315 | 0.002054 | 0.0009 | PPARD/CDKN1A/PPARG | 3 |
| BP | GO:0019915 | lipid storage | 3/37 | 0.000329 | 0.002135 | 0.000935 | PPARD/IL1B/PPARG | 3 |
| BP | GO:0071242 | cellular response to ammonium ion | 3/37 | 0.000329 | 0.002135 | 0.000935 | DRD1/CHRM2/CASP3 | 3 |
| BP | GO:0015711 | organic anion transport | 6/37 | 0.000338 | 0.002191 | 0.00096 | NOS2/NCOA2/PPARD/IL1B/PRKCD/PPARG | 6 |
| BP | GO:0051100 | negative regulation of binding | 4/37 | 0.000339 | 0.002192 | 0.00096 | JUN/ADRB2/BAX/PRKCD | 4 |
| BP | GO:0032310 | prostaglandin secretion | 2/37 | 0.000343 | 0.002199 | 0.000963 | NOS2/IL1B | 2 |
| BP | GO:0045651 | positive regulation of macrophage differentiation | 2/37 | 0.000343 | 0.002199 | 0.000963 | CASP8/PRKCA | 2 |
| BP | GO:2001279 | regulation of unsaturated fatty acid biosynthetic process | 2/37 | 0.000343 | 0.002199 | 0.000963 | PTGS2/IL1B | 2 |
| BP | GO:0016049 | cell growth | 6/37 | 0.000346 | 0.002213 | 0.000969 | ESR2/PPARD/BCL2/CDKN1A/TP53/PPARG | 6 |
| BP | GO:0010634 | positive regulation of epithelial cell migration | 4/37 | 0.000354 | 0.002255 | 0.000988 | PTGS2/KDR/JUN/PRKCA | 4 |
| BP | GO:0016241 | regulation of macroautophagy | 4/37 | 0.000354 | 0.002255 | 0.000988 | KDR/ADRB2/CASP3/TP53 | 4 |
| BP | GO:0046879 | hormone secretion | 5/37 | 0.000355 | 0.002255 | 0.000988 | NOS2/DPP4/PPARD/PRKCA/IL1B | 5 |
| BP | GO:0001659 | temperature homeostasis | 4/37 | 0.00037 | 0.002348 | 0.001029 | PTGS2/DRD1/ADRB2/IL1B | 4 |
| BP | GO:0070371 | ERK1 and ERK2 cascade | 5/37 | 0.000382 | 0.002413 | 0.001057 | KDR/JUN/PRKCA/MYC/IL1B | 5 |
| BP | GO:0051881 | regulation of mitochondrial membrane potential | 3/37 | 0.000389 | 0.002448 | 0.001072 | KDR/BCL2/BAX | 3 |
| BP | GO:1903747 | regulation of establishment of protein localization to mitochondrion | 3/37 | 0.000389 | 0.002448 | 0.001072 | BCL2/CASP8/TP53 | 3 |
| BP | GO:0030730 | sequestering of triglyceride | 2/37 | 0.000395 | 0.002452 | 0.001074 | IL1B/PPARG | 2 |
| BP | GO:0045898 | regulation of RNA polymerase II transcriptional preinitiation complex assembly | 2/37 | 0.000395 | 0.002452 | 0.001074 | ESR1/TP53 | 2 |
| BP | GO:0048070 | regulation of developmental pigmentation | 2/37 | 0.000395 | 0.002452 | 0.001074 | BCL2/BAX | 2 |
| BP | GO:1900119 | positive regulation of execution phase of apoptosis | 2/37 | 0.000395 | 0.002452 | 0.001074 | BAX/TP53 | 2 |
| BP | GO:1903729 | regulation of plasma membrane organization | 2/37 | 0.000395 | 0.002452 | 0.001074 | AR/PRKCD | 2 |
| BP | GO:0001936 | regulation of endothelial cell proliferation | 4/37 | 0.000395 | 0.002452 | 0.001074 | KDR/JUN/PRKCA/PPARG | 4 |
| BP | GO:0033555 | multicellular organismal response to stress | 3/37 | 0.000405 | 0.002501 | 0.001095 | DPP4/DRD1/BCL2 | 3 |
| BP | GO:0043627 | response to estrogen | 3/37 | 0.000405 | 0.002501 | 0.001095 | F7/ESR1/PPARG | 3 |
| BP | GO:0009914 | hormone transport | 5/37 | 0.00041 | 0.002525 | 0.001106 | NOS2/DPP4/PPARD/PRKCA/IL1B | 5 |
| BP | GO:0032103 | positive regulation of response to external stimulus | 5/37 | 0.000416 | 0.002555 | 0.001119 | PTGS2/F7/KDR/PRKCA/IL1B | 5 |
| BP | GO:0048771 | tissue remodeling | 4/37 | 0.000421 | 0.002578 | 0.001129 | ADRB2/BAX/PRKCA/TP53 | 4 |
| BP | GO:1900182 | positive regulation of protein localization to nucleus | 3/37 | 0.000422 | 0.002578 | 0.001129 | PTGS2/CDK1/PRKCD | 3 |
| BP | GO:0043534 | blood vessel endothelial cell migration | 4/37 | 0.00043 | 0.002614 | 0.001145 | PTGS2/KDR/PRKCA/PPARG | 4 |
| BP | GO:0071346 | cellular response to interferon-gamma | 4/37 | 0.00043 | 0.002614 | 0.001145 | NOS2/TP53/PRKCD/PPARG | 4 |
| BP | GO:0071466 | cellular response to xenobiotic stimulus | 4/37 | 0.00043 | 0.002614 | 0.001145 | PTGS1/CASP9/PCNA/DPEP1 | 4 |
| BP | GO:0150076 | neuroinflammatory response | 3/37 | 0.000439 | 0.002657 | 0.001164 | PTGS2/JUN/IL1B | 3 |
| BP | GO:0010506 | regulation of autophagy | 5/37 | 0.00044 | 0.002658 | 0.001164 | KDR/ADRB2/BCL2/CASP3/TP53 | 5 |
| BP | GO:0002070 | epithelial cell maturation | 2/37 | 0.000451 | 0.002692 | 0.001179 | PGR/CDKN1A | 2 |
| BP | GO:0010885 | regulation of cholesterol storage | 2/37 | 0.000451 | 0.002692 | 0.001179 | PPARD/PPARG | 2 |
| BP | GO:0019372 | lipoxygenase pathway | 2/37 | 0.000451 | 0.002692 | 0.001179 | PTGS2/PON1 | 2 |
| BP | GO:0045986 | negative regulation of smooth muscle contraction | 2/37 | 0.000451 | 0.002692 | 0.001179 | PTGS2/ADRB2 | 2 |
| BP | GO:0055012 | ventricular cardiac muscle cell differentiation | 2/37 | 0.000451 | 0.002692 | 0.001179 | CDK1/CCNB1 | 2 |
| BP | GO:0051235 | maintenance of location | 5/37 | 0.000459 | 0.002733 | 0.001197 | PPARD/DRD1/BAX/IL1B/PPARG | 5 |
| BP | GO:0042098 | T cell proliferation | 4/37 | 0.000468 | 0.002775 | 0.001216 | BAX/CASP3/TP53/IL1B | 4 |
| BP | GO:0048639 | positive regulation of developmental growth | 4/37 | 0.000468 | 0.002775 | 0.001216 | PPARD/BCL2/CDK1/CCNB1 | 4 |
| BP | GO:0006809 | nitric oxide biosynthetic process | 3/37 | 0.000474 | 0.002805 | 0.001229 | NOS2/PTGS2/IL1B | 3 |
| BP | GO:0015849 | organic acid transport | 5/37 | 0.000478 | 0.002816 | 0.001233 | NOS2/NCOA2/PPARD/IL1B/PPARG | 5 |
| BP | GO:0046942 | carboxylic acid transport | 5/37 | 0.000478 | 0.002816 | 0.001233 | NOS2/NCOA2/PPARD/IL1B/PPARG | 5 |
| BP | GO:0042246 | tissue regeneration | 3/37 | 0.000492 | 0.002887 | 0.001264 | PPARD/CDKN1A/CCNB1 | 3 |
| BP | GO:0090398 | cellular senescence | 3/37 | 0.000492 | 0.002887 | 0.001264 | CDKN1A/TP53/PRKCD | 3 |
| BP | GO:1905475 | regulation of protein localization to membrane | 4/37 | 0.000497 | 0.00291 | 0.001274 | AR/BCL2/CASP8/TP53 | 4 |
| BP | GO:0006978 | DNA damage response, signal transduction by p53 class mediator resulting in transcription of p21 class mediator | 2/37 | 0.00051 | 0.002917 | 0.001278 | CDKN1A/TP53 | 2 |
| BP | GO:0010224 | response to UV-B | 2/37 | 0.00051 | 0.002917 | 0.001278 | BCL2/CDKN1A | 2 |
| BP | GO:0015732 | prostaglandin transport | 2/37 | 0.00051 | 0.002917 | 0.001278 | NOS2/IL1B | 2 |
| BP | GO:0030540 | female genitalia development | 2/37 | 0.00051 | 0.002917 | 0.001278 | ESR1/BAX | 2 |
| BP | GO:0032966 | negative regulation of collagen biosynthetic process | 2/37 | 0.00051 | 0.002917 | 0.001278 | PPARD/PPARG | 2 |
| BP | GO:0070242 | thymocyte apoptotic process | 2/37 | 0.00051 | 0.002917 | 0.001278 | BAX/TP53 | 2 |
| BP | GO:0071850 | mitotic cell cycle arrest | 2/37 | 0.00051 | 0.002917 | 0.001278 | CDKN1A/TP53 | 2 |
| BP | GO:2001267 | regulation of cysteine-type endopeptidase activity involved in apoptotic signaling pathway | 2/37 | 0.00051 | 0.002917 | 0.001278 | BAX/CASP8 | 2 |
| BP | GO:0030193 | regulation of blood coagulation | 3/37 | 0.000511 | 0.002917 | 0.001278 | F7/PRKCA/PRKCD | 3 |
| BP | GO:0043536 | positive regulation of blood vessel endothelial cell migration | 3/37 | 0.000511 | 0.002917 | 0.001278 | PTGS2/KDR/PRKCA | 3 |
| BP | GO:0071260 | cellular response to mechanical stimulus | 3/37 | 0.000511 | 0.002917 | 0.001278 | PTGS2/CASP8/IL1B | 3 |
| BP | GO:0071248 | cellular response to metal ion | 4/37 | 0.000528 | 0.003008 | 0.001317 | PTGS2/JUN/CCNB1/DPEP1 | 4 |
| BP | GO:1900046 | regulation of hemostasis | 3/37 | 0.00053 | 0.003014 | 0.00132 | F7/PRKCA/PRKCD | 3 |
| BP | GO:0001935 | endothelial cell proliferation | 4/37 | 0.000538 | 0.003054 | 0.001338 | KDR/JUN/PRKCA/PPARG | 4 |
| BP | GO:0072330 | monocarboxylic acid biosynthetic process | 5/37 | 0.000547 | 0.003095 | 0.001356 | PTGS2/PTGS1/TP53/FASN/IL1B | 5 |
| BP | GO:0014855 | striated muscle cell proliferation | 3/37 | 0.00055 | 0.003098 | 0.001357 | PPARD/CDK1/CCNB1 | 3 |
| BP | GO:0110110 | positive regulation of animal organ morphogenesis | 3/37 | 0.00055 | 0.003098 | 0.001357 | AR/BAX/MYC | 3 |
| BP | GO:0046209 | nitric oxide metabolic process | 3/37 | 0.00057 | 0.003169 | 0.001388 | NOS2/PTGS2/IL1B | 3 |
| BP | GO:1905954 | positive regulation of lipid localization | 3/37 | 0.00057 | 0.003169 | 0.001388 | PON1/IL1B/PRKCD | 3 |
| BP | GO:0010713 | negative regulation of collagen metabolic process | 2/37 | 0.000573 | 0.003169 | 0.001388 | PPARD/PPARG | 2 |
| BP | GO:0010878 | cholesterol storage | 2/37 | 0.000573 | 0.003169 | 0.001388 | PPARD/PPARG | 2 |
| BP | GO:0042772 | DNA damage response, signal transduction resulting in transcription | 2/37 | 0.000573 | 0.003169 | 0.001388 | CDKN1A/TP53 | 2 |
| BP | GO:0060749 | mammary gland alveolus development | 2/37 | 0.000573 | 0.003169 | 0.001388 | AR/ESR1 | 2 |
| BP | GO:0061377 | mammary gland lobule development | 2/37 | 0.000573 | 0.003169 | 0.001388 | AR/ESR1 | 2 |
| BP | GO:0070230 | positive regulation of lymphocyte apoptotic process | 2/37 | 0.000573 | 0.003169 | 0.001388 | BAX/TP53 | 2 |
| BP | GO:0090330 | regulation of platelet aggregation | 2/37 | 0.000573 | 0.003169 | 0.001388 | PRKCA/PRKCD | 2 |
| BP | GO:0046889 | positive regulation of lipid biosynthetic process | 3/37 | 0.000611 | 0.003365 | 0.001474 | PTGS2/IL1B/PRKCD | 3 |
| BP | GO:0050818 | regulation of coagulation | 3/37 | 0.000611 | 0.003365 | 0.001474 | F7/PRKCA/PRKCD | 3 |
| BP | GO:0046890 | regulation of lipid biosynthetic process | 4/37 | 0.000616 | 0.003386 | 0.001483 | PTGS2/FASN/IL1B/PRKCD | 4 |
| BP | GO:0034341 | response to interferon-gamma | 4/37 | 0.000628 | 0.003443 | 0.001508 | NOS2/TP53/PRKCD/PPARG | 4 |
| BP | GO:1904705 | regulation of vascular smooth muscle cell proliferation | 3/37 | 0.000633 | 0.003447 | 0.00151 | JUN/CDKN1A/PPARG | 3 |
| BP | GO:1990874 | vascular smooth muscle cell proliferation | 3/37 | 0.000633 | 0.003447 | 0.00151 | JUN/CDKN1A/PPARG | 3 |
| BP | GO:2001057 | reactive nitrogen species metabolic process | 3/37 | 0.000633 | 0.003447 | 0.00151 | NOS2/PTGS2/IL1B | 3 |
| BP | GO:0010888 | negative regulation of lipid storage | 2/37 | 0.00064 | 0.003463 | 0.001517 | PPARD/PPARG | 2 |
| BP | GO:0033189 | response to vitamin A | 2/37 | 0.00064 | 0.003463 | 0.001517 | PPARD/PPARG | 2 |
| BP | GO:1903798 | regulation of production of miRNAs involved in gene silencing by miRNA | 2/37 | 0.00064 | 0.003463 | 0.001517 | ESR1/TP53 | 2 |
| BP | GO:0001776 | leukocyte homeostasis | 3/37 | 0.000655 | 0.00353 | 0.001546 | BCL2/BAX/CASP3 | 3 |
| BP | GO:0008625 | extrinsic apoptotic signaling pathway via death domain receptors | 3/37 | 0.000655 | 0.00353 | 0.001546 | BCL2/BAX/CASP8 | 3 |
| BP | GO:0045844 | positive regulation of striated muscle tissue development | 3/37 | 0.000677 | 0.003636 | 0.001592 | BCL2/CDK1/CCNB1 | 3 |
| BP | GO:0048636 | positive regulation of muscle organ development | 3/37 | 0.000677 | 0.003636 | 0.001592 | BCL2/CDK1/CCNB1 | 3 |
| BP | GO:0031331 | positive regulation of cellular catabolic process | 5/37 | 0.000689 | 0.003685 | 0.001614 | KDR/ADRB2/BAX/IL1B/PRKCD | 5 |
| BP | GO:0045766 | positive regulation of angiogenesis | 4/37 | 0.000689 | 0.003685 | 0.001614 | PTGS2/KDR/PRKCA/IL1B | 4 |
| BP | GO:0034103 | regulation of tissue remodeling | 3/37 | 0.0007 | 0.003722 | 0.00163 | BAX/PRKCA/TP53 | 3 |
| BP | GO:1901863 | positive regulation of muscle tissue development | 3/37 | 0.0007 | 0.003722 | 0.00163 | BCL2/CDK1/CCNB1 | 3 |
| BP | GO:0071222 | cellular response to lipopolysaccharide | 4/37 | 0.000702 | 0.003722 | 0.00163 | NOS2/PPARD/PRKCA/IL1B | 4 |
| BP | GO:0097164 | ammonium ion metabolic process | 4/37 | 0.000702 | 0.003722 | 0.00163 | AKR1B1/DRD1/PON1/PRKCD | 4 |
| BP | GO:0030220 | platelet formation | 2/37 | 0.00071 | 0.003742 | 0.001639 | CASP9/CASP3 | 2 |
| BP | GO:0045655 | regulation of monocyte differentiation | 2/37 | 0.00071 | 0.003742 | 0.001639 | JUN/MYC | 2 |
| BP | GO:0060261 | positive regulation of transcription initiation from RNA polymerase II promoter | 2/37 | 0.00071 | 0.003742 | 0.001639 | ESR1/TP53 | 2 |
| BP | GO:0006066 | alcohol metabolic process | 5/37 | 0.000715 | 0.003761 | 0.001648 | PPARD/AKR1B1/PON1/FASN/IL1B | 5 |
| BP | GO:0032102 | negative regulation of response to external stimulus | 5/37 | 0.000724 | 0.003801 | 0.001665 | PPARD/DRD1/CDKN1A/PRKCD/PPARG | 5 |
| BP | GO:0050670 | regulation of lymphocyte proliferation | 4/37 | 0.000741 | 0.003882 | 0.0017 | BCL2/CASP3/CDKN1A/IL1B | 4 |
| BP | GO:0001656 | metanephros development | 3/37 | 0.000748 | 0.0039 | 0.001708 | AKR1B1/BCL2/MYC | 3 |
| BP | GO:0031058 | positive regulation of histone modification | 3/37 | 0.000748 | 0.0039 | 0.001708 | TP53/IL1B/CCNB1 | 3 |
| BP | GO:0032944 | regulation of mononuclear cell proliferation | 4/37 | 0.000754 | 0.003921 | 0.001717 | BCL2/CASP3/CDKN1A/IL1B | 4 |
| BP | GO:0071229 | cellular response to acid chemical | 4/37 | 0.000754 | 0.003921 | 0.001717 | KDR/SLC6A4/CCNB1/PPARG | 4 |
| BP | GO:0045639 | positive regulation of myeloid cell differentiation | 3/37 | 0.000772 | 0.003987 | 0.001747 | JUN/CASP8/PRKCA | 3 |
| BP | GO:0051899 | membrane depolarization | 3/37 | 0.000772 | 0.003987 | 0.001747 | KDR/JUN/BCL2 | 3 |
| BP | GO:0060333 | interferon-gamma-mediated signaling pathway | 3/37 | 0.000772 | 0.003987 | 0.001747 | TP53/PRKCD/PPARG | 3 |
| BP | GO:0030728 | ovulation | 2/37 | 0.000784 | 0.004 | 0.001752 | PTGS2/PGR | 2 |
| BP | GO:0036344 | platelet morphogenesis | 2/37 | 0.000784 | 0.004 | 0.001752 | CASP9/CASP3 | 2 |
| BP | GO:0046827 | positive regulation of protein export from nucleus | 2/37 | 0.000784 | 0.004 | 0.001752 | TP53/IL1B | 2 |
| BP | GO:0051900 | regulation of mitochondrial depolarization | 2/37 | 0.000784 | 0.004 | 0.001752 | KDR/BCL2 | 2 |
| BP | GO:0055093 | response to hyperoxia | 2/37 | 0.000784 | 0.004 | 0.001752 | CDKN1A/PPARG | 2 |
| BP | GO:0070920 | regulation of production of small RNA involved in gene silencing by RNA | 2/37 | 0.000784 | 0.004 | 0.001752 | ESR1/TP53 | 2 |
| BP | GO:0071219 | cellular response to molecule of bacterial origin | 4/37 | 0.000796 | 0.004054 | 0.001776 | NOS2/PPARD/PRKCA/IL1B | 4 |
| BP | GO:0032388 | positive regulation of intracellular transport | 4/37 | 0.000838 | 0.004263 | 0.001867 | PTGS2/TP53/IL1B/PRKCD | 4 |
| BP | GO:0032069 | regulation of nuclease activity | 2/37 | 0.000861 | 0.004335 | 0.001899 | PCNA/PRKCD | 2 |
| BP | GO:0032469 | endoplasmic reticulum calcium ion homeostasis | 2/37 | 0.000861 | 0.004335 | 0.001899 | BCL2/BAX | 2 |
| BP | GO:0043496 | regulation of protein homodimerization activity | 2/37 | 0.000861 | 0.004335 | 0.001899 | BCL2/BAX | 2 |
| BP | GO:0045649 | regulation of macrophage differentiation | 2/37 | 0.000861 | 0.004335 | 0.001899 | CASP8/PRKCA | 2 |
| BP | GO:0045723 | positive regulation of fatty acid biosynthetic process | 2/37 | 0.000861 | 0.004335 | 0.001899 | PTGS2/IL1B | 2 |
| BP | GO:0043393 | regulation of protein binding | 4/37 | 0.000868 | 0.004353 | 0.001907 | ADRB2/BCL2/BAX/PRKCD | 4 |
| BP | GO:0071241 | cellular response to inorganic substance | 4/37 | 0.000868 | 0.004353 | 0.001907 | PTGS2/JUN/CCNB1/DPEP1 | 4 |
| BP | GO:0010001 | glial cell differentiation | 4/37 | 0.000883 | 0.004419 | 0.001936 | DRD1/CDK1/IL1B/PPARG | 4 |
| BP | GO:0045765 | regulation of angiogenesis | 5/37 | 0.000899 | 0.004488 | 0.001966 | PTGS2/KDR/PRKCA/IL1B/PPARG | 5 |
| BP | GO:0019217 | regulation of fatty acid metabolic process | 3/37 | 0.000902 | 0.004488 | 0.001966 | PTGS2/IL1B/PPARG | 3 |
| BP | GO:0097327 | response to antineoplastic agent | 3/37 | 0.000902 | 0.004488 | 0.001966 | F7/CASP9/PCNA | 3 |
| BP | GO:0007188 | adenylate cyclase-modulating G protein-coupled receptor signaling pathway | 4/37 | 0.000929 | 0.004607 | 0.002018 | DRD1/CHRM2/ADRB2/PRKCA | 4 |
| BP | GO:2001022 | positive regulation of response to DNA damage stimulus | 3/37 | 0.000929 | 0.004607 | 0.002018 | PCNA/MYC/PRKCD | 3 |
| BP | GO:0051882 | mitochondrial depolarization | 2/37 | 0.000942 | 0.004661 | 0.002041 | KDR/BCL2 | 2 |
| BP | GO:0070663 | regulation of leukocyte proliferation | 4/37 | 0.000945 | 0.004665 | 0.002044 | BCL2/CASP3/CDKN1A/IL1B | 4 |
| BP | GO:0045444 | fat cell differentiation | 4/37 | 0.000961 | 0.004735 | 0.002074 | PTGS2/PPARD/ADRB2/PPARG | 4 |
| BP | GO:0070301 | cellular response to hydrogen peroxide | 3/37 | 0.000986 | 0.004851 | 0.002125 | CDK1/PCNA/PRKCD | 3 |
| BP | GO:0033157 | regulation of intracellular protein transport | 4/37 | 0.000993 | 0.004876 | 0.002136 | PTGS2/TP53/IL1B/PRKCD | 4 |
| BP | GO:0007200 | phospholipase C-activating G protein-coupled receptor signaling pathway | 3/37 | 0.001015 | 0.004975 | 0.002179 | ESR1/DRD1/CHRM2 | 3 |
| BP | GO:0010959 | regulation of metal ion transport | 5/37 | 0.00102 | 0.004983 | 0.002183 | PTGS2/DRD1/ADRB2/BCL2/BAX | 5 |
| BP | GO:0044346 | fibroblast apoptotic process | 2/37 | 0.001026 | 0.004983 | 0.002183 | TP53/MYC | 2 |
| BP | GO:0046697 | decidualization | 2/37 | 0.001026 | 0.004983 | 0.002183 | PTGS2/PPARD | 2 |
| BP | GO:0050996 | positive regulation of lipid catabolic process | 2/37 | 0.001026 | 0.004983 | 0.002183 | IL1B/PRKCD | 2 |
| BP | GO:2000178 | negative regulation of neural precursor cell proliferation | 2/37 | 0.001026 | 0.004983 | 0.002183 | SLC6A4/TP53 | 2 |
| BP | GO:0048661 | positive regulation of smooth muscle cell proliferation | 3/37 | 0.001045 | 0.005063 | 0.002218 | PTGS2/AKR1B1/JUN | 3 |
| BP | GO:0010594 | regulation of endothelial cell migration | 4/37 | 0.00106 | 0.005127 | 0.002246 | PTGS2/KDR/PRKCA/PPARG | 4 |
| BP | GO:0000079 | regulation of cyclin-dependent protein serine/threonine kinase activity | 3/37 | 0.001075 | 0.005172 | 0.002266 | CASP3/CDKN1A/CCNB1 | 3 |
| BP | GO:0010522 | regulation of calcium ion transport into cytosol | 3/37 | 0.001075 | 0.005172 | 0.002266 | DRD1/BCL2/BAX | 3 |
| BP | GO:0009743 | response to carbohydrate | 4/37 | 0.001077 | 0.005172 | 0.002266 | PTGS2/PPARD/CASP3/IL1B | 4 |
| BP | GO:1904018 | positive regulation of vasculature development | 4/37 | 0.001077 | 0.005172 | 0.002266 | PTGS2/KDR/PRKCA/IL1B | 4 |
| BP | GO:0018958 | phenol-containing compound metabolic process | 3/37 | 0.001106 | 0.00528 | 0.002313 | AKR1B1/DRD1/BCL2 | 3 |
| BP | GO:0032091 | negative regulation of protein binding | 3/37 | 0.001106 | 0.00528 | 0.002313 | ADRB2/BAX/PRKCD | 3 |
| BP | GO:1905269 | positive regulation of chromatin organization | 3/37 | 0.001106 | 0.00528 | 0.002313 | TP53/IL1B/CCNB1 | 3 |
| BP | GO:0009299 | mRNA transcription | 2/37 | 0.001114 | 0.005281 | 0.002313 | PPARD/TP53 | 2 |
| BP | GO:0032461 | positive regulation of protein oligomerization | 2/37 | 0.001114 | 0.005281 | 0.002313 | BAX/TP53 | 2 |
| BP | GO:0034110 | regulation of homotypic cell-cell adhesion | 2/37 | 0.001114 | 0.005281 | 0.002313 | PRKCA/PRKCD | 2 |
| BP | GO:0050927 | positive regulation of positive chemotaxis | 2/37 | 0.001114 | 0.005281 | 0.002313 | F7/KDR | 2 |
| BP | GO:0022407 | regulation of cell-cell adhesion | 5/37 | 0.001128 | 0.005338 | 0.002338 | DPP4/CASP3/PRKCA/IL1B/PRKCD | 5 |
| BP | GO:0043900 | regulation of multi-organism process | 5/37 | 0.001153 | 0.005447 | 0.002386 | NOS2/JUN/BCL2/PRKCA/IL1B | 5 |
| BP | GO:0006469 | negative regulation of protein kinase activity | 4/37 | 0.001166 | 0.005499 | 0.002408 | CASP3/CDKN1A/IL1B/PRKCD | 4 |
| BP | GO:1904029 | regulation of cyclin-dependent protein kinase activity | 3/37 | 0.001201 | 0.005621 | 0.002462 | CASP3/CDKN1A/CCNB1 | 3 |
| BP | GO:0002360 | T cell lineage commitment | 2/37 | 0.001205 | 0.005621 | 0.002462 | BCL2/TP53 | 2 |
| BP | GO:0006309 | apoptotic DNA fragmentation | 2/37 | 0.001205 | 0.005621 | 0.002462 | BAX/CASP3 | 2 |
| BP | GO:0050926 | regulation of positive chemotaxis | 2/37 | 0.001205 | 0.005621 | 0.002462 | F7/KDR | 2 |
| BP | GO:0060740 | prostate gland epithelium morphogenesis | 2/37 | 0.001205 | 0.005621 | 0.002462 | AR/ESR1 | 2 |
| BP | GO:0097066 | response to thyroid hormone | 2/37 | 0.001205 | 0.005621 | 0.002462 | F7/AKR1B1 | 2 |
| BP | GO:1904659 | glucose transmembrane transport | 3/37 | 0.001268 | 0.005901 | 0.002585 | PPARD/DRD1/IL1B | 3 |
| BP | GO:0010971 | positive regulation of G2/M transition of mitotic cell cycle | 2/37 | 0.0013 | 0.006017 | 0.002635 | CDK1/CCNB1 | 2 |
| BP | GO:0045932 | negative regulation of muscle contraction | 2/37 | 0.0013 | 0.006017 | 0.002635 | PTGS2/ADRB2 | 2 |
| BP | GO:0098810 | neurotransmitter reuptake | 2/37 | 0.0013 | 0.006017 | 0.002635 | DRD1/SLC6A4 | 2 |
| BP | GO:0021761 | limbic system development | 3/37 | 0.001302 | 0.006017 | 0.002635 | DRD1/BAX/CASP3 | 3 |
| BP | GO:0051222 | positive regulation of protein transport | 5/37 | 0.001327 | 0.006121 | 0.002681 | PTGS2/PPARD/TP53/IL1B/PRKCD | 5 |
| BP | GO:0033559 | unsaturated fatty acid metabolic process | 3/37 | 0.001336 | 0.006155 | 0.002696 | PTGS2/PTGS1/IL1B | 3 |
| BP | GO:1901342 | regulation of vasculature development | 5/37 | 0.001384 | 0.006336 | 0.002775 | PTGS2/KDR/PRKCA/IL1B/PPARG | 5 |
| BP | GO:0009896 | positive regulation of catabolic process | 5/37 | 0.001398 | 0.006336 | 0.002775 | KDR/ADRB2/BAX/IL1B/PRKCD | 5 |
| BP | GO:0033598 | mammary gland epithelial cell proliferation | 2/37 | 0.001398 | 0.006336 | 0.002775 | ESR1/BAX | 2 |
| BP | GO:0036296 | response to increased oxygen levels | 2/37 | 0.001398 | 0.006336 | 0.002775 | CDKN1A/PPARG | 2 |
| BP | GO:0051123 | RNA polymerase II preinitiation complex assembly | 2/37 | 0.001398 | 0.006336 | 0.002775 | ESR1/TP53 | 2 |
| BP | GO:0060260 | regulation of transcription initiation from RNA polymerase II promoter | 2/37 | 0.001398 | 0.006336 | 0.002775 | ESR1/TP53 | 2 |
| BP | GO:0060512 | prostate gland morphogenesis | 2/37 | 0.001398 | 0.006336 | 0.002775 | AR/ESR1 | 2 |
| BP | GO:1990776 | response to angiotensin | 2/37 | 0.001398 | 0.006336 | 0.002775 | PTGS2/PRKCD | 2 |
| BP | GO:2000108 | positive regulation of leukocyte apoptotic process | 2/37 | 0.001398 | 0.006336 | 0.002775 | BAX/TP53 | 2 |
| BP | GO:0001938 | positive regulation of endothelial cell proliferation | 3/37 | 0.001408 | 0.006336 | 0.002775 | KDR/JUN/PRKCA | 3 |
| BP | GO:0008645 | hexose transmembrane transport | 3/37 | 0.001408 | 0.006336 | 0.002775 | PPARD/DRD1/IL1B | 3 |
| BP | GO:0032411 | positive regulation of transporter activity | 3/37 | 0.001408 | 0.006336 | 0.002775 | ADRB2/PON1/PRKCD | 3 |
| BP | GO:0043279 | response to alkaloid | 3/37 | 0.001408 | 0.006336 | 0.002775 | DRD1/CASP3/PPARG | 3 |
| BP | GO:0042180 | cellular ketone metabolic process | 4/37 | 0.001422 | 0.006391 | 0.0028 | PTGS2/AKR1B1/IL1B/PPARG | 4 |
| BP | GO:0019318 | hexose metabolic process | 4/37 | 0.001443 | 0.006433 | 0.002818 | NCOA2/PPARD/AKR1B1/TP53 | 4 |
| BP | GO:0043200 | response to amino acid | 3/37 | 0.001444 | 0.006433 | 0.002818 | F7/CASP3/PCNA | 3 |
| BP | GO:0046620 | regulation of organ growth | 3/37 | 0.001444 | 0.006433 | 0.002818 | SLC6A4/CDK1/CCNB1 | 3 |
| BP | GO:0048640 | negative regulation of developmental growth | 3/37 | 0.001444 | 0.006433 | 0.002818 | ADRB2/SLC6A4/CDKN1A | 3 |
| BP | GO:0060964 | regulation of gene silencing by miRNA | 3/37 | 0.001444 | 0.006433 | 0.002818 | ESR1/TP53/PPARG | 3 |
| BP | GO:0015749 | monosaccharide transmembrane transport | 3/37 | 0.001481 | 0.006564 | 0.002875 | PPARD/DRD1/IL1B | 3 |
| BP | GO:0042752 | regulation of circadian rhythm | 3/37 | 0.001481 | 0.006564 | 0.002875 | TP53/CDK1/PPARG | 3 |
| BP | GO:0045446 | endothelial cell differentiation | 3/37 | 0.001481 | 0.006564 | 0.002875 | KDR/FASN/IL1B | 3 |
| BP | GO:0045637 | regulation of myeloid cell differentiation | 4/37 | 0.001486 | 0.006577 | 0.002881 | JUN/CASP8/PRKCA/MYC | 4 |
| BP | GO:0010575 | positive regulation of vascular endothelial growth factor production | 2/37 | 0.0015 | 0.006627 | 0.002903 | PTGS2/IL1B | 2 |
| BP | GO:0051924 | regulation of calcium ion transport | 4/37 | 0.001553 | 0.006842 | 0.002997 | PTGS2/DRD1/BCL2/BAX | 4 |
| BP | GO:0034219 | carbohydrate transmembrane transport | 3/37 | 0.001557 | 0.006842 | 0.002997 | PPARD/DRD1/IL1B | 3 |
| BP | GO:1900180 | regulation of protein localization to nucleus | 3/37 | 0.001557 | 0.006842 | 0.002997 | PTGS2/CDK1/PRKCD | 3 |
| BP | GO:0007613 | memory | 3/37 | 0.001596 | 0.006937 | 0.003039 | PTGS2/DRD1/SLC6A4 | 3 |
| BP | GO:0060147 | regulation of posttranscriptional gene silencing | 3/37 | 0.001596 | 0.006937 | 0.003039 | ESR1/TP53/PPARG | 3 |
| BP | GO:0060966 | regulation of gene silencing by RNA | 3/37 | 0.001596 | 0.006937 | 0.003039 | ESR1/TP53/PPARG | 3 |
| BP | GO:0006839 | mitochondrial transport | 4/37 | 0.001598 | 0.006937 | 0.003039 | BCL2/BAX/CASP8/TP53 | 4 |
| BP | GO:0007187 | G protein-coupled receptor signaling pathway, coupled to cyclic nucleotide second messenger | 4/37 | 0.001598 | 0.006937 | 0.003039 | DRD1/CHRM2/ADRB2/PRKCA | 4 |
| BP | GO:0043434 | response to peptide hormone | 5/37 | 0.001598 | 0.006937 | 0.003039 | PTGS2/F7/IL1B/PRKCD/PPARG | 5 |
| BP | GO:0048873 | homeostasis of number of cells within a tissue | 2/37 | 0.001605 | 0.006937 | 0.003039 | BCL2/BAX | 2 |
| BP | GO:0071480 | cellular response to gamma radiation | 2/37 | 0.001605 | 0.006937 | 0.003039 | CDKN1A/TP53 | 2 |
| BP | GO:0090200 | positive regulation of release of cytochrome c from mitochondria | 2/37 | 0.001605 | 0.006937 | 0.003039 | BAX/TP53 | 2 |
| BP | GO:1902751 | positive regulation of cell cycle G2/M phase transition | 2/37 | 0.001605 | 0.006937 | 0.003039 | CDK1/CCNB1 | 2 |
| BP | GO:0033673 | negative regulation of kinase activity | 4/37 | 0.001621 | 0.006993 | 0.003063 | CASP3/CDKN1A/IL1B/PRKCD | 4 |
| BP | GO:0010256 | endomembrane system organization | 5/37 | 0.00163 | 0.007023 | 0.003076 | AR/PRKCA/CDK1/PRKCD/CCNB1 | 5 |
| BP | GO:0019932 | second-messenger-mediated signaling | 5/37 | 0.001647 | 0.007082 | 0.003102 | NOS2/KDR/DRD1/ADRB2/PRKCA | 5 |
| BP | GO:0090257 | regulation of muscle system process | 4/37 | 0.001668 | 0.007159 | 0.003136 | PTGS2/CHRM2/ADRB2/PRKCA | 4 |
| BP | GO:0002082 | regulation of oxidative phosphorylation | 2/37 | 0.001714 | 0.007285 | 0.003191 | CDK1/CCNB1 | 2 |
| BP | GO:0002675 | positive regulation of acute inflammatory response | 2/37 | 0.001714 | 0.007285 | 0.003191 | PTGS2/IL1B | 2 |
| BP | GO:0010543 | regulation of platelet activation | 2/37 | 0.001714 | 0.007285 | 0.003191 | PRKCA/PRKCD | 2 |
| BP | GO:0033198 | response to ATP | 2/37 | 0.001714 | 0.007285 | 0.003191 | PTGS2/IL1B | 2 |
| BP | GO:1902253 | regulation of intrinsic apoptotic signaling pathway by p53 class mediator | 2/37 | 0.001714 | 0.007285 | 0.003191 | BCL2/TP53 | 2 |
| BP | GO:1902895 | positive regulation of pri-miRNA transcription by RNA polymerase II | 2/37 | 0.001714 | 0.007285 | 0.003191 | JUN/TP53 | 2 |
| BP | GO:1903409 | reactive oxygen species biosynthetic process | 3/37 | 0.001799 | 0.007608 | 0.003333 | NOS2/PTGS2/IL1B | 3 |
| BP | GO:1903578 | regulation of ATP metabolic process | 3/37 | 0.001799 | 0.007608 | 0.003333 | TP53/CDK1/CCNB1 | 3 |
| BP | GO:1905477 | positive regulation of protein localization to membrane | 3/37 | 0.001799 | 0.007608 | 0.003333 | BCL2/CASP8/TP53 | 3 |
| BP | GO:0042311 | vasodilation | 2/37 | 0.001826 | 0.007698 | 0.003372 | DRD1/ADRB2 | 2 |
| BP | GO:0051968 | positive regulation of synaptic transmission, glutamatergic | 2/37 | 0.001826 | 0.007698 | 0.003372 | PTGS2/DRD1 | 2 |
| BP | GO:0044839 | cell cycle G2/M phase transition | 4/37 | 0.001838 | 0.007738 | 0.003389 | CDKN1A/TP53/CDK1/CCNB1 | 4 |
| BP | GO:0014013 | regulation of gliogenesis | 3/37 | 0.001884 | 0.007919 | 0.003468 | CDK1/IL1B/PPARG | 3 |
| BP | GO:0031334 | positive regulation of protein complex assembly | 4/37 | 0.001889 | 0.007926 | 0.003472 | JUN/ESR1/BAX/TP53 | 4 |
| BP | GO:0043491 | protein kinase B signaling | 4/37 | 0.001915 | 0.008022 | 0.003514 | F7/KDR/ESR1/IL1B | 4 |
| BP | GO:0045927 | positive regulation of growth | 4/37 | 0.001941 | 0.008092 | 0.003545 | PPARD/BCL2/CDK1/CCNB1 | 4 |
| BP | GO:0010039 | response to iron ion | 2/37 | 0.001941 | 0.008092 | 0.003545 | BCL2/CCNB1 | 2 |
| BP | GO:0045736 | negative regulation of cyclin-dependent protein serine/threonine kinase activity | 2/37 | 0.001941 | 0.008092 | 0.003545 | CASP3/CDKN1A | 2 |
| BP | GO:1901617 | organic hydroxy compound biosynthetic process | 4/37 | 0.001967 | 0.008188 | 0.003587 | AKR1B1/TP53/FASN/IL1B | 4 |
| BP | GO:1902105 | regulation of leukocyte differentiation | 4/37 | 0.001994 | 0.008285 | 0.003629 | JUN/CASP8/PRKCA/MYC | 4 |
| BP | GO:0006260 | DNA replication | 4/37 | 0.002048 | 0.00849 | 0.003719 | JUN/TP53/CDK1/PCNA | 4 |
| BP | GO:0046394 | carboxylic acid biosynthetic process | 5/37 | 0.00206 | 0.00849 | 0.003719 | PTGS2/PTGS1/TP53/FASN/IL1B | 5 |
| BP | GO:0000737 | DNA catabolic process, endonucleolytic | 2/37 | 0.00206 | 0.00849 | 0.003719 | BAX/CASP3 | 2 |
| BP | GO:0010574 | regulation of vascular endothelial growth factor production | 2/37 | 0.00206 | 0.00849 | 0.003719 | PTGS2/IL1B | 2 |
| BP | GO:1904030 | negative regulation of cyclin-dependent protein kinase activity | 2/37 | 0.00206 | 0.00849 | 0.003719 | CASP3/CDKN1A | 2 |
| BP | GO:0010595 | positive regulation of endothelial cell migration | 3/37 | 0.002063 | 0.00849 | 0.003719 | PTGS2/KDR/PRKCA | 3 |
| BP | GO:0016053 | organic acid biosynthetic process | 5/37 | 0.002079 | 0.008543 | 0.003742 | PTGS2/PTGS1/TP53/FASN/IL1B | 5 |
| BP | GO:0006997 | nucleus organization | 3/37 | 0.002156 | 0.008828 | 0.003867 | PRKCA/CDK1/CCNB1 | 3 |
| BP | GO:0050671 | positive regulation of lymphocyte proliferation | 3/37 | 0.002156 | 0.008828 | 0.003867 | BCL2/CDKN1A/IL1B | 3 |
| BP | GO:0043254 | regulation of protein complex assembly | 5/37 | 0.002158 | 0.008828 | 0.003867 | JUN/ESR1/BAX/TP53/PRKCD | 5 |
| BP | GO:0001893 | maternal placenta development | 2/37 | 0.002182 | 0.008882 | 0.003891 | PTGS2/PPARD | 2 |
| BP | GO:0030262 | apoptotic nuclear changes | 2/37 | 0.002182 | 0.008882 | 0.003891 | BAX/CASP3 | 2 |
| BP | GO:1904031 | positive regulation of cyclin-dependent protein kinase activity | 2/37 | 0.002182 | 0.008882 | 0.003891 | CDKN1A/CCNB1 | 2 |
| BP | GO:0030183 | B cell differentiation | 3/37 | 0.002204 | 0.008942 | 0.003917 | BCL2/BAX/TP53 | 3 |
| BP | GO:0032946 | positive regulation of mononuclear cell proliferation | 3/37 | 0.002204 | 0.008942 | 0.003917 | BCL2/CDKN1A/IL1B | 3 |
| BP | GO:0003158 | endothelium development | 3/37 | 0.002252 | 0.009109 | 0.00399 | KDR/FASN/IL1B | 3 |
| BP | GO:0045598 | regulation of fat cell differentiation | 3/37 | 0.002252 | 0.009109 | 0.00399 | PTGS2/PPARD/PPARG | 3 |
| BP | GO:0050708 | regulation of protein secretion | 5/37 | 0.002261 | 0.009131 | 0.004 | NOS2/DPP4/PPARD/PRKCA/IL1B | 5 |
| BP | GO:0010573 | vascular endothelial growth factor production | 2/37 | 0.002308 | 0.009262 | 0.004057 | PTGS2/IL1B | 2 |
| BP | GO:0046627 | negative regulation of insulin receptor signaling pathway | 2/37 | 0.002308 | 0.009262 | 0.004057 | IL1B/PRKCD | 2 |
| BP | GO:1901099 | negative regulation of signal transduction in absence of ligand | 2/37 | 0.002308 | 0.009262 | 0.004057 | BCL2/IL1B | 2 |
| BP | GO:2001240 | negative regulation of extrinsic apoptotic signaling pathway in absence of ligand | 2/37 | 0.002308 | 0.009262 | 0.004057 | BCL2/IL1B | 2 |
| BP | GO:0006766 | vitamin metabolic process | 3/37 | 0.00235 | 0.009404 | 0.004119 | PRSS1/PPARD/IL1B | 3 |
| BP | GO:0048565 | digestive tract development | 3/37 | 0.00235 | 0.009404 | 0.004119 | BCL2/CDKN1A/CCNB1 | 3 |
| BP | GO:0034976 | response to endoplasmic reticulum stress | 4/37 | 0.002362 | 0.009437 | 0.004134 | JUN/BCL2/BAX/TP53 | 4 |
| BP | GO:0060359 | response to ammonium ion | 3/37 | 0.0024 | 0.009575 | 0.004194 | DRD1/CHRM2/CASP3 | 3 |
| BP | GO:0006921 | cellular component disassembly involved in execution phase of apoptosis | 2/37 | 0.002436 | 0.009675 | 0.004238 | BAX/CASP3 | 2 |
| BP | GO:0042307 | positive regulation of protein import into nucleus | 2/37 | 0.002436 | 0.009675 | 0.004238 | PTGS2/PRKCD | 2 |
| BP | GO:0090050 | positive regulation of cell migration involved in sprouting angiogenesis | 2/37 | 0.002436 | 0.009675 | 0.004238 | PTGS2/KDR | 2 |
| BP | GO:0042063 | gliogenesis | 4/37 | 0.002515 | 0.009973 | 0.004368 | DRD1/CDK1/IL1B/PPARG | 4 |
| BP | GO:0051249 | regulation of lymphocyte activation | 5/37 | 0.002543 | 0.010069 | 0.00441 | DPP4/BCL2/CASP3/CDKN1A/IL1B | 5 |
| BP | GO:0007190 | activation of adenylate cyclase activity | 2/37 | 0.002569 | 0.010076 | 0.004413 | DRD1/ADRB2 | 2 |
| BP | GO:0046825 | regulation of protein export from nucleus | 2/37 | 0.002569 | 0.010076 | 0.004413 | TP53/IL1B | 2 |
| BP | GO:0060045 | positive regulation of cardiac muscle cell proliferation | 2/37 | 0.002569 | 0.010076 | 0.004413 | CDK1/CCNB1 | 2 |
| BP | GO:1900077 | negative regulation of cellular response to insulin stimulus | 2/37 | 0.002569 | 0.010076 | 0.004413 | IL1B/PRKCD | 2 |
| BP | GO:1904706 | negative regulation of vascular smooth muscle cell proliferation | 2/37 | 0.002569 | 0.010076 | 0.004413 | CDKN1A/PPARG | 2 |
| BP | GO:2000279 | negative regulation of DNA biosynthetic process | 2/37 | 0.002569 | 0.010076 | 0.004413 | TP53/PPARG | 2 |
| BP | GO:0005996 | monosaccharide metabolic process | 4/37 | 0.002579 | 0.0101 | 0.004424 | NCOA2/PPARD/AKR1B1/TP53 | 4 |
| BP | GO:0007189 | adenylate cyclase-activating G protein-coupled receptor signaling pathway | 3/37 | 0.002607 | 0.010149 | 0.004445 | DRD1/ADRB2/PRKCA | 3 |
| BP | GO:0033135 | regulation of peptidyl-serine phosphorylation | 3/37 | 0.002607 | 0.010149 | 0.004445 | PTGS2/BCL2/BAX | 3 |
| BP | GO:0051588 | regulation of neurotransmitter transport | 3/37 | 0.002607 | 0.010149 | 0.004445 | DRD1/CHRM2/SLC6A4 | 3 |
| BP | GO:0070665 | positive regulation of leukocyte proliferation | 3/37 | 0.002607 | 0.010149 | 0.004445 | BCL2/CDKN1A/IL1B | 3 |
| BP | GO:0051146 | striated muscle cell differentiation | 4/37 | 0.002611 | 0.010149 | 0.004445 | BCL2/CASP3/CDK1/CCNB1 | 4 |
| BP | GO:0016236 | macroautophagy | 4/37 | 0.002675 | 0.010385 | 0.004549 | KDR/ADRB2/CASP3/TP53 | 4 |
| BP | GO:0010661 | positive regulation of muscle cell apoptotic process | 2/37 | 0.002704 | 0.010449 | 0.004577 | TP53/PPARG | 2 |
| BP | GO:0033146 | regulation of intracellular estrogen receptor signaling pathway | 2/37 | 0.002704 | 0.010449 | 0.004577 | AR/ESR1 | 2 |
| BP | GO:1904591 | positive regulation of protein import | 2/37 | 0.002704 | 0.010449 | 0.004577 | PTGS2/PRKCD | 2 |
| BP | GO:0006914 | autophagy | 5/37 | 0.002802 | 0.010797 | 0.004729 | KDR/ADRB2/BCL2/CASP3/TP53 | 5 |
| BP | GO:0061919 | process utilizing autophagic mechanism | 5/37 | 0.002802 | 0.010797 | 0.004729 | KDR/ADRB2/BCL2/CASP3/TP53 | 5 |
| BP | GO:0031056 | regulation of histone modification | 3/37 | 0.002825 | 0.01087 | 0.004761 | TP53/IL1B/CCNB1 | 3 |
| BP | GO:0070372 | regulation of ERK1 and ERK2 cascade | 4/37 | 0.002842 | 0.010872 | 0.004762 | KDR/JUN/PRKCA/IL1B | 4 |
| BP | GO:0007618 | mating | 2/37 | 0.002843 | 0.010872 | 0.004762 | DRD1/SLC6A4 | 2 |
| BP | GO:0051281 | positive regulation of release of sequestered calcium ion into cytosol | 2/37 | 0.002843 | 0.010872 | 0.004762 | DRD1/BAX | 2 |
| BP | GO:0071548 | response to dexamethasone | 2/37 | 0.002843 | 0.010872 | 0.004762 | CASP9/PCNA | 2 |
| BP | GO:1902107 | positive regulation of leukocyte differentiation | 3/37 | 0.002882 | 0.010988 | 0.004813 | JUN/CASP8/PRKCA | 3 |
| BP | GO:1904064 | positive regulation of cation transmembrane transport | 3/37 | 0.002882 | 0.010988 | 0.004813 | DRD1/ADRB2/BAX | 3 |
| BP | GO:0002791 | regulation of peptide secretion | 5/37 | 0.002901 | 0.011045 | 0.004838 | NOS2/DPP4/PPARD/PRKCA/IL1B | 5 |
| BP | GO:0007612 | learning | 3/37 | 0.002938 | 0.011172 | 0.004894 | PTGS2/JUN/DRD1 | 3 |
| BP | GO:0060326 | cell chemotaxis | 4/37 | 0.002981 | 0.011233 | 0.00492 | F7/KDR/IL1B/PRKCD | 4 |
| BP | GO:0006308 | DNA catabolic process | 2/37 | 0.002985 | 0.011233 | 0.00492 | BAX/CASP3 | 2 |
| BP | GO:0030890 | positive regulation of B cell proliferation | 2/37 | 0.002985 | 0.011233 | 0.00492 | BCL2/CDKN1A | 2 |
| BP | GO:0050873 | brown fat cell differentiation | 2/37 | 0.002985 | 0.011233 | 0.00492 | PTGS2/ADRB2 | 2 |
| BP | GO:0061028 | establishment of endothelial barrier | 2/37 | 0.002985 | 0.011233 | 0.00492 | FASN/IL1B | 2 |
| BP | GO:0150077 | regulation of neuroinflammatory response | 2/37 | 0.002985 | 0.011233 | 0.00492 | PTGS2/IL1B | 2 |
| BP | GO:1900117 | regulation of execution phase of apoptosis | 2/37 | 0.002985 | 0.011233 | 0.00492 | BAX/TP53 | 2 |
| BP | GO:0055123 | digestive system development | 3/37 | 0.002996 | 0.011259 | 0.004932 | BCL2/CDKN1A/CCNB1 | 3 |
| BP | GO:0043524 | negative regulation of neuron apoptotic process | 3/37 | 0.003054 | 0.011461 | 0.00502 | JUN/BCL2/BAX | 3 |
| BP | GO:0008643 | carbohydrate transport | 3/37 | 0.003113 | 0.011666 | 0.00511 | PPARD/DRD1/IL1B | 3 |
| BP | GO:0009895 | negative regulation of catabolic process | 4/37 | 0.003124 | 0.011678 | 0.005115 | NOS2/BCL2/TP53/IL1B | 4 |
| BP | GO:0006284 | base-excision repair | 2/37 | 0.00313 | 0.011678 | 0.005115 | TP53/PCNA | 2 |
| BP | GO:0042149 | cellular response to glucose starvation | 2/37 | 0.00313 | 0.011678 | 0.005115 | BCL2/TP53 | 2 |
| BP | GO:0008203 | cholesterol metabolic process | 3/37 | 0.003233 | 0.012046 | 0.005276 | PPARD/PON1/FASN | 3 |
| BP | GO:0006775 | fat-soluble vitamin metabolic process | 2/37 | 0.003279 | 0.012145 | 0.00532 | PPARD/IL1B | 2 |
| BP | GO:0008631 | intrinsic apoptotic signaling pathway in response to oxidative stress | 2/37 | 0.003279 | 0.012145 | 0.00532 | BCL2/PRKCD | 2 |
| BP | GO:0032459 | regulation of protein oligomerization | 2/37 | 0.003279 | 0.012145 | 0.00532 | BAX/TP53 | 2 |
| BP | GO:0045429 | positive regulation of nitric oxide biosynthetic process | 2/37 | 0.003279 | 0.012145 | 0.00532 | PTGS2/IL1B | 2 |
| BP | GO:0016202 | regulation of striated muscle tissue development | 3/37 | 0.003356 | 0.012397 | 0.00543 | BCL2/CDK1/CCNB1 | 3 |
| BP | GO:0030902 | hindbrain development | 3/37 | 0.003356 | 0.012397 | 0.00543 | SLC6A4/BCL2/TP53 | 3 |
| BP | GO:0014002 | astrocyte development | 2/37 | 0.003431 | 0.012547 | 0.005496 | DRD1/IL1B | 2 |
| BP | GO:0030225 | macrophage differentiation | 2/37 | 0.003431 | 0.012547 | 0.005496 | CASP8/PRKCA | 2 |
| BP | GO:0032965 | regulation of collagen biosynthetic process | 2/37 | 0.003431 | 0.012547 | 0.005496 | PPARD/PPARG | 2 |
| BP | GO:0034105 | positive regulation of tissue remodeling | 2/37 | 0.003431 | 0.012547 | 0.005496 | BAX/PRKCA | 2 |
| BP | GO:0070266 | necroptotic process | 2/37 | 0.003431 | 0.012547 | 0.005496 | CASP8/TP53 | 2 |
| BP | GO:0070897 | transcription preinitiation complex assembly | 2/37 | 0.003431 | 0.012547 | 0.005496 | ESR1/TP53 | 2 |
| BP | GO:1904407 | positive regulation of nitric oxide metabolic process | 2/37 | 0.003431 | 0.012547 | 0.005496 | PTGS2/IL1B | 2 |
| BP | GO:0007204 | positive regulation of cytosolic calcium ion concentration | 4/37 | 0.003542 | 0.012895 | 0.005648 | ESR1/DRD1/BCL2/BAX | 4 |
| BP | GO:0060968 | regulation of gene silencing | 3/37 | 0.003546 | 0.012895 | 0.005648 | ESR1/TP53/PPARG | 3 |
| BP | GO:1901861 | regulation of muscle tissue development | 3/37 | 0.003546 | 0.012895 | 0.005648 | BCL2/CDK1/CCNB1 | 3 |
| BP | GO:1902652 | secondary alcohol metabolic process | 3/37 | 0.003546 | 0.012895 | 0.005648 | PPARD/PON1/FASN | 3 |
| BP | GO:0032309 | icosanoid secretion | 2/37 | 0.003586 | 0.013022 | 0.005704 | NOS2/IL1B | 2 |
| BP | GO:0034767 | positive regulation of ion transmembrane transport | 3/37 | 0.00361 | 0.013075 | 0.005727 | DRD1/ADRB2/BAX | 3 |
| BP | GO:0048634 | regulation of muscle organ development | 3/37 | 0.00361 | 0.013075 | 0.005727 | BCL2/CDK1/CCNB1 | 3 |
| BP | GO:0060402 | calcium ion transport into cytosol | 3/37 | 0.003742 | 0.013409 | 0.005874 | DRD1/BCL2/BAX | 3 |
| BP | GO:0001504 | neurotransmitter uptake | 2/37 | 0.003744 | 0.013409 | 0.005874 | DRD1/SLC6A4 | 2 |
| BP | GO:0010883 | regulation of lipid storage | 2/37 | 0.003744 | 0.013409 | 0.005874 | PPARD/PPARG | 2 |
| BP | GO:0014075 | response to amine | 2/37 | 0.003744 | 0.013409 | 0.005874 | DRD1/CDK1 | 2 |
| BP | GO:0035196 | production of miRNAs involved in gene silencing by miRNA | 2/37 | 0.003744 | 0.013409 | 0.005874 | ESR1/TP53 | 2 |
| BP | GO:0045776 | negative regulation of blood pressure | 2/37 | 0.003744 | 0.013409 | 0.005874 | NOS2/ADRB2 | 2 |
| BP | GO:0048512 | circadian behavior | 2/37 | 0.003744 | 0.013409 | 0.005874 | NCOA2/TP53 | 2 |
| BP | GO:0048599 | oocyte development | 2/37 | 0.003744 | 0.013409 | 0.005874 | BCL2/CCNB1 | 2 |
| BP | GO:0002673 | regulation of acute inflammatory response | 3/37 | 0.003809 | 0.013622 | 0.005967 | PTGS2/IL1B/PPARG | 3 |
| BP | GO:0006953 | acute-phase response | 2/37 | 0.003906 | 0.013835 | 0.00606 | PTGS2/IL1B | 2 |
| BP | GO:0007622 | rhythmic behavior | 2/37 | 0.003906 | 0.013835 | 0.00606 | NCOA2/TP53 | 2 |
| BP | GO:0030261 | chromosome condensation | 2/37 | 0.003906 | 0.013835 | 0.00606 | CDK1/CCNB1 | 2 |
| BP | GO:0048066 | developmental pigmentation | 2/37 | 0.003906 | 0.013835 | 0.00606 | BCL2/BAX | 2 |
| BP | GO:0070231 | T cell apoptotic process | 2/37 | 0.003906 | 0.013835 | 0.00606 | BAX/TP53 | 2 |
| BP | GO:1903580 | positive regulation of ATP metabolic process | 2/37 | 0.003906 | 0.013835 | 0.00606 | CDK1/CCNB1 | 2 |
| BP | GO:2001239 | regulation of extrinsic apoptotic signaling pathway in absence of ligand | 2/37 | 0.003906 | 0.013835 | 0.00606 | BCL2/IL1B | 2 |
| BP | GO:0016485 | protein processing | 4/37 | 0.003911 | 0.013837 | 0.006061 | F7/CASP3/CASP8/IL1B | 4 |
| BP | GO:0030850 | prostate gland development | 2/37 | 0.00407 | 0.014321 | 0.006273 | AR/ESR1 | 2 |
| BP | GO:0055023 | positive regulation of cardiac muscle tissue growth | 2/37 | 0.00407 | 0.014321 | 0.006273 | CDK1/CCNB1 | 2 |
| BP | GO:0072604 | interleukin-6 secretion | 2/37 | 0.00407 | 0.014321 | 0.006273 | NOS2/IL1B | 2 |
| BP | GO:0090199 | regulation of release of cytochrome c from mitochondria | 2/37 | 0.00407 | 0.014321 | 0.006273 | BAX/TP53 | 2 |
| BP | GO:0090150 | establishment of protein localization to membrane | 4/37 | 0.004084 | 0.014348 | 0.006285 | BCL2/BAX/CASP8/TP53 | 4 |
| BP | GO:0032147 | activation of protein kinase activity | 4/37 | 0.004127 | 0.014482 | 0.006344 | ADRB2/CDK1/IL1B/PRKCD | 4 |
| BP | GO:0035821 | modification of morphology or physiology of other organism | 3/37 | 0.004153 | 0.014554 | 0.006375 | NOS2/JUN/CASP8 | 3 |
| BP | GO:0030336 | negative regulation of cell migration | 4/37 | 0.004172 | 0.014558 | 0.006377 | PPARD/BCL2/DPEP1/PPARG | 4 |
| BP | GO:0051251 | positive regulation of lymphocyte activation | 4/37 | 0.004172 | 0.014558 | 0.006377 | DPP4/BCL2/CDKN1A/IL1B | 4 |
| BP | GO:0071902 | positive regulation of protein serine/threonine kinase activity | 4/37 | 0.004172 | 0.014558 | 0.006377 | ADRB2/CDK1/IL1B/CCNB1 | 4 |
| BP | GO:0050680 | negative regulation of epithelial cell proliferation | 3/37 | 0.004225 | 0.014634 | 0.00641 | AR/PPARD/PPARG | 3 |
| BP | GO:0003254 | regulation of membrane depolarization | 2/37 | 0.004238 | 0.014634 | 0.00641 | KDR/BCL2 | 2 |
| BP | GO:0010712 | regulation of collagen metabolic process | 2/37 | 0.004238 | 0.014634 | 0.00641 | PPARD/PPARG | 2 |
| BP | GO:0035094 | response to nicotine | 2/37 | 0.004238 | 0.014634 | 0.00641 | BCL2/CASP3 | 2 |
| BP | GO:0043124 | negative regulation of I-kappaB kinase/NF-kappaB signaling | 2/37 | 0.004238 | 0.014634 | 0.00641 | ESR1/CASP8 | 2 |
| BP | GO:0061912 | selective autophagy | 2/37 | 0.004238 | 0.014634 | 0.00641 | ADRB2/TP53 | 2 |
| BP | GO:0071715 | icosanoid transport | 2/37 | 0.004238 | 0.014634 | 0.00641 | NOS2/IL1B | 2 |
| BP | GO:1901571 | fatty acid derivative transport | 2/37 | 0.004238 | 0.014634 | 0.00641 | NOS2/IL1B | 2 |
| BP | GO:0016125 | sterol metabolic process | 3/37 | 0.004297 | 0.014815 | 0.006489 | PPARD/PON1/FASN | 3 |
| BP | GO:1903793 | positive regulation of anion transport | 2/37 | 0.004409 | 0.015184 | 0.006651 | IL1B/PRKCD | 2 |
| BP | GO:0050806 | positive regulation of synaptic transmission | 3/37 | 0.004443 | 0.015278 | 0.006692 | PTGS2/DRD1/ADRB2 | 3 |
| BP | GO:0050728 | negative regulation of inflammatory response | 3/37 | 0.004517 | 0.015513 | 0.006795 | PPARD/PRKCD/PPARG | 3 |
| BP | GO:0033044 | regulation of chromosome organization | 4/37 | 0.004536 | 0.01556 | 0.006815 | TP53/MYC/IL1B/CCNB1 | 4 |
| BP | GO:0009994 | oocyte differentiation | 2/37 | 0.004584 | 0.015598 | 0.006832 | BCL2/CCNB1 | 2 |
| BP | GO:0019369 | arachidonic acid metabolic process | 2/37 | 0.004584 | 0.015598 | 0.006832 | PTGS2/PTGS1 | 2 |
| BP | GO:0031050 | dsRNA processing | 2/37 | 0.004584 | 0.015598 | 0.006832 | ESR1/TP53 | 2 |
| BP | GO:0031103 | axon regeneration | 2/37 | 0.004584 | 0.015598 | 0.006832 | JUN/BCL2 | 2 |
| BP | GO:0070918 | production of small RNA involved in gene silencing by RNA | 2/37 | 0.004584 | 0.015598 | 0.006832 | ESR1/TP53 | 2 |
| BP | GO:0097366 | response to bronchodilator | 2/37 | 0.004584 | 0.015598 | 0.006832 | DRD1/DPEP1 | 2 |
| BP | GO:0010721 | negative regulation of cell development | 4/37 | 0.004631 | 0.015738 | 0.006894 | SLC6A4/BCL2/TP53/IL1B | 4 |
| BP | GO:0006937 | regulation of muscle contraction | 3/37 | 0.004667 | 0.01582 | 0.00693 | PTGS2/CHRM2/ADRB2 | 3 |
| BP | GO:0060401 | cytosolic calcium ion transport | 3/37 | 0.004667 | 0.01582 | 0.00693 | DRD1/BCL2/BAX | 3 |
| BP | GO:0021543 | pallium development | 3/37 | 0.004744 | 0.016058 | 0.007034 | DRD1/BAX/CASP3 | 3 |
| BP | GO:0060421 | positive regulation of heart growth | 2/37 | 0.004761 | 0.016096 | 0.00705 | CDK1/CCNB1 | 2 |
| BP | GO:0010469 | regulation of signaling receptor activity | 3/37 | 0.004821 | 0.016256 | 0.00712 | ESR2/ADRB2/PRKCD | 3 |
| BP | GO:0055002 | striated muscle cell development | 3/37 | 0.004821 | 0.016256 | 0.00712 | BCL2/CDK1/CCNB1 | 3 |
| BP | GO:2000146 | negative regulation of cell motility | 4/37 | 0.004873 | 0.01641 | 0.007188 | PPARD/BCL2/DPEP1/PPARG | 4 |
| BP | GO:2001252 | positive regulation of chromosome organization | 3/37 | 0.004899 | 0.016476 | 0.007217 | TP53/IL1B/CCNB1 | 3 |
| BP | GO:0032964 | collagen biosynthetic process | 2/37 | 0.004942 | 0.016576 | 0.007261 | PPARD/PPARG | 2 |
| BP | GO:0070228 | regulation of lymphocyte apoptotic process | 2/37 | 0.004942 | 0.016576 | 0.007261 | BAX/TP53 | 2 |
| BP | GO:0046165 | alcohol biosynthetic process | 3/37 | 0.004978 | 0.016676 | 0.007304 | AKR1B1/FASN/IL1B | 3 |
| BP | GO:0006584 | catecholamine metabolic process | 2/37 | 0.005125 | 0.017082 | 0.007482 | AKR1B1/DRD1 | 2 |
| BP | GO:0009712 | catechol-containing compound metabolic process | 2/37 | 0.005125 | 0.017082 | 0.007482 | AKR1B1/DRD1 | 2 |
| BP | GO:0010524 | positive regulation of calcium ion transport into cytosol | 2/37 | 0.005125 | 0.017082 | 0.007482 | DRD1/BAX | 2 |
| BP | GO:0050994 | regulation of lipid catabolic process | 2/37 | 0.005125 | 0.017082 | 0.007482 | IL1B/PRKCD | 2 |
| BP | GO:0050821 | protein stabilization | 3/37 | 0.005218 | 0.01737 | 0.007608 | CDKN1A/TP53/PRKCD | 3 |
| BP | GO:0051480 | regulation of cytosolic calcium ion concentration | 4/37 | 0.005278 | 0.017545 | 0.007685 | ESR1/DRD1/BCL2/BAX | 4 |
| BP | GO:0042304 | regulation of fatty acid biosynthetic process | 2/37 | 0.005312 | 0.017614 | 0.007715 | PTGS2/IL1B | 2 |
| BP | GO:0042306 | regulation of protein import into nucleus | 2/37 | 0.005312 | 0.017614 | 0.007715 | PTGS2/PRKCD | 2 |
| BP | GO:0006936 | muscle contraction | 4/37 | 0.005435 | 0.017999 | 0.007884 | PTGS2/DRD1/CHRM2/ADRB2 | 4 |
| BP | GO:0010565 | regulation of cellular ketone metabolic process | 3/37 | 0.005466 | 0.018078 | 0.007919 | PTGS2/IL1B/PPARG | 3 |
| BP | GO:0060688 | regulation of morphogenesis of a branching structure | 2/37 | 0.005502 | 0.018174 | 0.007961 | AR/ESR1 | 2 |
| BP | GO:0009566 | fertilization | 3/37 | 0.00555 | 0.018309 | 0.00802 | AR/BAX/CDK1 | 3 |
| BP | GO:0060043 | regulation of cardiac muscle cell proliferation | 2/37 | 0.005695 | 0.018716 | 0.008198 | CDK1/CCNB1 | 2 |
| BP | GO:0071398 | cellular response to fatty acid | 2/37 | 0.005695 | 0.018716 | 0.008198 | CCNB1/PPARG | 2 |
| BP | GO:1903428 | positive regulation of reactive oxygen species biosynthetic process | 2/37 | 0.005695 | 0.018716 | 0.008198 | PTGS2/IL1B | 2 |
| BP | GO:0043409 | negative regulation of MAPK cascade | 3/37 | 0.005721 | 0.018753 | 0.008214 | MYC/IL1B/PRKCD | 3 |
| BP | GO:0050864 | regulation of B cell activation | 3/37 | 0.005721 | 0.018753 | 0.008214 | BCL2/CASP3/CDKN1A | 3 |
| BP | GO:1902275 | regulation of chromatin organization | 3/37 | 0.005807 | 0.018988 | 0.008317 | TP53/IL1B/CCNB1 | 3 |
| BP | GO:1903708 | positive regulation of hemopoiesis | 3/37 | 0.005807 | 0.018988 | 0.008317 | JUN/CASP8/PRKCA | 3 |
| BP | GO:0031663 | lipopolysaccharide-mediated signaling pathway | 2/37 | 0.005891 | 0.019152 | 0.008389 | PRKCA/IL1B | 2 |
| BP | GO:0061900 | glial cell activation | 2/37 | 0.005891 | 0.019152 | 0.008389 | JUN/IL1B | 2 |
| BP | GO:1904589 | regulation of protein import | 2/37 | 0.005891 | 0.019152 | 0.008389 | PTGS2/PRKCD | 2 |
| BP | GO:0022900 | electron transport chain | 3/37 | 0.005894 | 0.019152 | 0.008389 | AKR1B1/CDK1/CCNB1 | 3 |
| BP | GO:0055001 | muscle cell development | 3/37 | 0.005894 | 0.019152 | 0.008389 | BCL2/CDK1/CCNB1 | 3 |
| BP | GO:0019933 | cAMP-mediated signaling | 3/37 | 0.005982 | 0.019414 | 0.008504 | DRD1/ADRB2/PRKCA | 3 |
| BP | GO:0043902 | positive regulation of multi-organism process | 3/37 | 0.006071 | 0.019642 | 0.008603 | NOS2/JUN/PRKCA | 3 |
| BP | GO:0001658 | branching involved in ureteric bud morphogenesis | 2/37 | 0.00609 | 0.019642 | 0.008603 | BCL2/MYC | 2 |
| BP | GO:0001885 | endothelial cell development | 2/37 | 0.00609 | 0.019642 | 0.008603 | FASN/IL1B | 2 |
| BP | GO:0031102 | neuron projection regeneration | 2/37 | 0.00609 | 0.019642 | 0.008603 | JUN/BCL2 | 2 |
| BP | GO:0070527 | platelet aggregation | 2/37 | 0.00609 | 0.019642 | 0.008603 | PRKCA/PRKCD | 2 |
| BP | GO:0001701 | in utero embryonic development | 4/37 | 0.006154 | 0.019823 | 0.008683 | AR/CASP8/TP53/CCNB1 | 4 |
| BP | GO:0090303 | positive regulation of wound healing | 2/37 | 0.006498 | 0.020878 | 0.009145 | F7/PPARD | 2 |
| BP | GO:1905953 | negative regulation of lipid localization | 2/37 | 0.006498 | 0.020878 | 0.009145 | PPARD/PPARG | 2 |
| BP | GO:0045333 | cellular respiration | 3/37 | 0.006527 | 0.020945 | 0.009175 | NOS2/CDK1/CCNB1 | 3 |
| BP | GO:0002696 | positive regulation of leukocyte activation | 4/37 | 0.006566 | 0.021046 | 0.009219 | DPP4/BCL2/CDKN1A/IL1B | 4 |
| BP | GO:0045453 | bone resorption | 2/37 | 0.006706 | 0.021441 | 0.009391 | ADRB2/PRKCA | 2 |
| BP | GO:0055025 | positive regulation of cardiac muscle tissue development | 2/37 | 0.006706 | 0.021441 | 0.009391 | CDK1/CCNB1 | 2 |
| BP | GO:0006694 | steroid biosynthetic process | 3/37 | 0.00681 | 0.02172 | 0.009514 | AKR1B1/FASN/IL1B | 3 |
| BP | GO:0051271 | negative regulation of cellular component movement | 4/37 | 0.00681 | 0.02172 | 0.009514 | PPARD/BCL2/DPEP1/PPARG | 4 |
| BP | GO:0042692 | muscle cell differentiation | 4/37 | 0.006872 | 0.02189 | 0.009588 | BCL2/CASP3/CDK1/CCNB1 | 4 |
| BP | GO:0032371 | regulation of sterol transport | 2/37 | 0.006917 | 0.021954 | 0.009616 | PON1/PPARG | 2 |
| BP | GO:0032374 | regulation of cholesterol transport | 2/37 | 0.006917 | 0.021954 | 0.009616 | PON1/PPARG | 2 |
| BP | GO:0046622 | positive regulation of organ growth | 2/37 | 0.006917 | 0.021954 | 0.009616 | CDK1/CCNB1 | 2 |
| BP | GO:0009165 | nucleotide biosynthetic process | 4/37 | 0.006934 | 0.02198 | 0.009628 | NOS2/PTGS2/TP53/FASN | 4 |
| BP | GO:0007626 | locomotory behavior | 3/37 | 0.007003 | 0.02217 | 0.009711 | DPP4/NCOA2/DRD1 | 3 |
| BP | GO:1903672 | positive regulation of sprouting angiogenesis | 2/37 | 0.007131 | 0.022523 | 0.009866 | PTGS2/KDR | 2 |
| BP | GO:2000378 | negative regulation of reactive oxygen species metabolic process | 2/37 | 0.007131 | 0.022523 | 0.009866 | BCL2/TP53 | 2 |
| BP | GO:1901293 | nucleoside phosphate biosynthetic process | 4/37 | 0.007187 | 0.02267 | 0.00993 | NOS2/PTGS2/TP53/FASN | 4 |
| BP | GO:0014823 | response to activity | 2/37 | 0.007349 | 0.023125 | 0.010129 | PPARD/CDK1 | 2 |
| BP | GO:0060675 | ureteric bud morphogenesis | 2/37 | 0.007349 | 0.023125 | 0.010129 | BCL2/MYC | 2 |
| BP | GO:0009746 | response to hexose | 3/37 | 0.007398 | 0.023251 | 0.010184 | PTGS2/PPARD/CASP3 | 3 |
| BP | GO:0050867 | positive regulation of cell activation | 4/37 | 0.007445 | 0.023372 | 0.010237 | DPP4/BCL2/CDKN1A/IL1B | 4 |
| BP | GO:0006687 | glycosphingolipid metabolic process | 2/37 | 0.007569 | 0.023585 | 0.010331 | BAX/PRKCD | 2 |
| BP | GO:0040014 | regulation of multicellular organism growth | 2/37 | 0.007569 | 0.023585 | 0.010331 | ADRB2/BCL2 | 2 |
| BP | GO:0045428 | regulation of nitric oxide biosynthetic process | 2/37 | 0.007569 | 0.023585 | 0.010331 | PTGS2/IL1B | 2 |
| BP | GO:0046626 | regulation of insulin receptor signaling pathway | 2/37 | 0.007569 | 0.023585 | 0.010331 | IL1B/PRKCD | 2 |
| BP | GO:0051926 | negative regulation of calcium ion transport | 2/37 | 0.007569 | 0.023585 | 0.010331 | PTGS2/BCL2 | 2 |
| BP | GO:0072171 | mesonephric tubule morphogenesis | 2/37 | 0.007569 | 0.023585 | 0.010331 | BCL2/MYC | 2 |
| BP | GO:0040013 | negative regulation of locomotion | 4/37 | 0.007577 | 0.023585 | 0.010331 | PPARD/BCL2/DPEP1/PPARG | 4 |
| BP | GO:0034764 | positive regulation of transmembrane transport | 3/37 | 0.0076 | 0.02363 | 0.01035 | DRD1/ADRB2/BAX | 3 |
| BP | GO:0051604 | protein maturation | 4/37 | 0.007643 | 0.023735 | 0.010396 | F7/CASP3/CASP8/IL1B | 4 |
| BP | GO:0001503 | ossification | 4/37 | 0.00771 | 0.023914 | 0.010475 | PTGS2/ADRB2/BCL2/FASN | 4 |
| BP | GO:0019748 | secondary metabolic process | 2/37 | 0.007792 | 0.024111 | 0.010561 | AKR1B1/BCL2 | 2 |
| BP | GO:0060038 | cardiac muscle cell proliferation | 2/37 | 0.007792 | 0.024111 | 0.010561 | CDK1/CCNB1 | 2 |
| BP | GO:0034284 | response to monosaccharide | 3/37 | 0.00791 | 0.024446 | 0.010708 | PTGS2/PPARD/CASP3 | 3 |
| BP | GO:1901215 | negative regulation of neuron death | 3/37 | 0.008015 | 0.024693 | 0.010816 | JUN/BCL2/BAX | 3 |
| BP | GO:0016239 | positive regulation of macroautophagy | 2/37 | 0.008018 | 0.024693 | 0.010816 | KDR/ADRB2 | 2 |
| BP | GO:0050918 | positive chemotaxis | 2/37 | 0.008018 | 0.024693 | 0.010816 | F7/KDR | 2 |
| BP | GO:0045785 | positive regulation of cell adhesion | 4/37 | 0.00805 | 0.02476 | 0.010845 | DPP4/KDR/PRKCA/IL1B | 4 |
| BP | GO:0006006 | glucose metabolic process | 3/37 | 0.008121 | 0.024919 | 0.010915 | NCOA2/PPARD/TP53 | 3 |
| BP | GO:0051701 | interaction with host | 3/37 | 0.008121 | 0.024919 | 0.010915 | DPP4/CASP8/CDK1 | 3 |
| BP | GO:0006323 | DNA packaging | 3/37 | 0.008227 | 0.025216 | 0.011045 | TP53/CDK1/CCNB1 | 3 |
| BP | GO:0070227 | lymphocyte apoptotic process | 2/37 | 0.008247 | 0.025219 | 0.011046 | BAX/TP53 | 2 |
| BP | GO:0071300 | cellular response to retinoic acid | 2/37 | 0.008247 | 0.025219 | 0.011046 | SLC6A4/PPARG | 2 |
| BP | GO:0033077 | T cell differentiation in thymus | 2/37 | 0.008479 | 0.025837 | 0.011317 | BCL2/TP53 | 2 |
| BP | GO:0050766 | positive regulation of phagocytosis | 2/37 | 0.008479 | 0.025837 | 0.011317 | IL1B/PPARG | 2 |
| BP | GO:0051966 | regulation of synaptic transmission, glutamatergic | 2/37 | 0.008479 | 0.025837 | 0.011317 | PTGS2/DRD1 | 2 |
| BP | GO:1902749 | regulation of cell cycle G2/M phase transition | 3/37 | 0.008552 | 0.026027 | 0.0114 | TP53/CDK1/CCNB1 | 3 |
| BP | GO:0071479 | cellular response to ionizing radiation | 2/37 | 0.008714 | 0.026459 | 0.01159 | CDKN1A/TP53 | 2 |
| BP | GO:1904427 | positive regulation of calcium ion transmembrane transport | 2/37 | 0.008714 | 0.026459 | 0.01159 | DRD1/BAX | 2 |
| BP | GO:0019935 | cyclic-nucleotide-mediated signaling | 3/37 | 0.008772 | 0.026573 | 0.01164 | DRD1/ADRB2/PRKCA | 3 |
| BP | GO:0070374 | positive regulation of ERK1 and ERK2 cascade | 3/37 | 0.008772 | 0.026573 | 0.01164 | KDR/JUN/PRKCA | 3 |
| BP | GO:0032869 | cellular response to insulin stimulus | 3/37 | 0.008884 | 0.02688 | 0.011774 | IL1B/PRKCD/PPARG | 3 |
| BP | GO:0006801 | superoxide metabolic process | 2/37 | 0.009193 | 0.02775 | 0.012155 | NOS2/PRKCD | 2 |
| BP | GO:0045685 | regulation of glial cell differentiation | 2/37 | 0.009193 | 0.02775 | 0.012155 | CDK1/PPARG | 2 |
| BP | GO:0002526 | acute inflammatory response | 3/37 | 0.009338 | 0.028156 | 0.012333 | PTGS2/IL1B/PPARG | 3 |
| BP | GO:0014015 | positive regulation of gliogenesis | 2/37 | 0.009437 | 0.028288 | 0.012391 | IL1B/PPARG | 2 |
| BP | GO:0031670 | cellular response to nutrient | 2/37 | 0.009437 | 0.028288 | 0.012391 | PTGS2/PPARG | 2 |
| BP | GO:0072078 | nephron tubule morphogenesis | 2/37 | 0.009437 | 0.028288 | 0.012391 | BCL2/MYC | 2 |
| BP | GO:1900076 | regulation of cellular response to insulin stimulus | 2/37 | 0.009437 | 0.028288 | 0.012391 | IL1B/PRKCD | 2 |
| BP | GO:1903036 | positive regulation of response to wounding | 2/37 | 0.009437 | 0.028288 | 0.012391 | F7/PPARD | 2 |
| BP | GO:0019674 | NAD metabolic process | 2/37 | 0.009933 | 0.029638 | 0.012982 | PTGS2/TP53 | 2 |
| BP | GO:0042310 | vasoconstriction | 2/37 | 0.009933 | 0.029638 | 0.012982 | PTGS2/SLC6A4 | 2 |
| BP | GO:0061045 | negative regulation of wound healing | 2/37 | 0.009933 | 0.029638 | 0.012982 | CDKN1A/PRKCD | 2 |
| BP | GO:0072088 | nephron epithelium morphogenesis | 2/37 | 0.009933 | 0.029638 | 0.012982 | BCL2/MYC | 2 |
| BP | GO:0006816 | calcium ion transport | 4/37 | 0.010377 | 0.030867 | 0.013521 | PTGS2/DRD1/BCL2/BAX | 4 |
| BP | GO:0033143 | regulation of intracellular steroid hormone receptor signaling pathway | 2/37 | 0.01044 | 0.030867 | 0.013521 | AR/ESR1 | 2 |
| BP | GO:0043407 | negative regulation of MAP kinase activity | 2/37 | 0.01044 | 0.030867 | 0.013521 | IL1B/PRKCD | 2 |
| BP | GO:0055021 | regulation of cardiac muscle tissue growth | 2/37 | 0.01044 | 0.030867 | 0.013521 | CDK1/CCNB1 | 2 |
| BP | GO:0061333 | renal tubule morphogenesis | 2/37 | 0.01044 | 0.030867 | 0.013521 | BCL2/MYC | 2 |
| BP | GO:0072028 | nephron morphogenesis | 2/37 | 0.01044 | 0.030867 | 0.013521 | BCL2/MYC | 2 |
| BP | GO:0072347 | response to anesthetic | 2/37 | 0.01044 | 0.030867 | 0.013521 | F7/DRD1 | 2 |
| BP | GO:2000300 | regulation of synaptic vesicle exocytosis | 2/37 | 0.01044 | 0.030867 | 0.013521 | DRD1/CHRM2 | 2 |
| BP | GO:0042445 | hormone metabolic process | 3/37 | 0.010783 | 0.031845 | 0.013949 | AKR1B1/ESR1/IL1B | 3 |
| BP | GO:0022617 | extracellular matrix disassembly | 2/37 | 0.010959 | 0.032327 | 0.01416 | DPP4/PRSS1 | 2 |
| BP | GO:0007045 | cell-substrate adherens junction assembly | 2/37 | 0.011222 | 0.032843 | 0.014386 | KDR/BCL2 | 2 |
| BP | GO:0021766 | hippocampus development | 2/37 | 0.011222 | 0.032843 | 0.014386 | DRD1/CASP3 | 2 |
| BP | GO:0031145 | anaphase-promoting complex-dependent catabolic process | 2/37 | 0.011222 | 0.032843 | 0.014386 | CDK1/CCNB1 | 2 |
| BP | GO:0034109 | homotypic cell-cell adhesion | 2/37 | 0.011222 | 0.032843 | 0.014386 | PRKCA/PRKCD | 2 |
| BP | GO:0048041 | focal adhesion assembly | 2/37 | 0.011222 | 0.032843 | 0.014386 | KDR/BCL2 | 2 |
| BP | GO:0048708 | astrocyte differentiation | 2/37 | 0.011222 | 0.032843 | 0.014386 | DRD1/IL1B | 2 |
| BP | GO:1902930 | regulation of alcohol biosynthetic process | 2/37 | 0.011222 | 0.032843 | 0.014386 | FASN/IL1B | 2 |
| BP | GO:0044282 | small molecule catabolic process | 4/37 | 0.011298 | 0.033029 | 0.014467 | NOS2/PPARD/PON1/TP53 | 4 |
| BP | GO:0043122 | regulation of I-kappaB kinase/NF-kappaB signaling | 3/37 | 0.011421 | 0.03335 | 0.014608 | ESR1/CASP8/IL1B | 3 |
| BP | GO:0032637 | interleukin-8 production | 2/37 | 0.011488 | 0.033396 | 0.014628 | NOS2/IL1B | 2 |
| BP | GO:0034502 | protein localization to chromosome | 2/37 | 0.011488 | 0.033396 | 0.014628 | ESR1/CDK1 | 2 |
| BP | GO:0051279 | regulation of release of sequestered calcium ion into cytosol | 2/37 | 0.011488 | 0.033396 | 0.014628 | DRD1/BAX | 2 |
| BP | GO:0090049 | regulation of cell migration involved in sprouting angiogenesis | 2/37 | 0.011488 | 0.033396 | 0.014628 | PTGS2/KDR | 2 |
| BP | GO:0031348 | negative regulation of defense response | 3/37 | 0.011682 | 0.033922 | 0.014858 | PPARD/PRKCD/PPARG | 3 |
| BP | GO:0071277 | cellular response to calcium ion | 2/37 | 0.011757 | 0.034064 | 0.014921 | JUN/DPEP1 | 2 |
| BP | GO:2000106 | regulation of leukocyte apoptotic process | 2/37 | 0.011757 | 0.034064 | 0.014921 | BAX/TP53 | 2 |
| BP | GO:0045088 | regulation of innate immune response | 4/37 | 0.011911 | 0.03447 | 0.015099 | ESR1/CASP8/PRKCD/PPARG | 4 |
| BP | GO:0034329 | cell junction assembly | 3/37 | 0.011947 | 0.034536 | 0.015127 | KDR/BCL2/PRKCA | 3 |
| BP | GO:0010507 | negative regulation of autophagy | 2/37 | 0.012029 | 0.034543 | 0.01513 | BCL2/TP53 | 2 |
| BP | GO:0014910 | regulation of smooth muscle cell migration | 2/37 | 0.012029 | 0.034543 | 0.01513 | PPARD/BCL2 | 2 |
| BP | GO:0043154 | negative regulation of cysteine-type endopeptidase activity involved in apoptotic process | 2/37 | 0.012029 | 0.034543 | 0.01513 | PTGS2/DPEP1 | 2 |
| BP | GO:0060420 | regulation of heart growth | 2/37 | 0.012029 | 0.034543 | 0.01513 | CDK1/CCNB1 | 2 |
| BP | GO:0097756 | negative regulation of blood vessel diameter | 2/37 | 0.012029 | 0.034543 | 0.01513 | PTGS2/SLC6A4 | 2 |
| BP | GO:1902803 | regulation of synaptic vesicle transport | 2/37 | 0.012029 | 0.034543 | 0.01513 | DRD1/CHRM2 | 2 |
| BP | GO:0006874 | cellular calcium ion homeostasis | 4/37 | 0.012453 | 0.03572 | 0.015646 | ESR1/DRD1/BCL2/BAX | 4 |
| BP | GO:0042446 | hormone biosynthetic process | 2/37 | 0.012581 | 0.036048 | 0.01579 | AKR1B1/IL1B | 2 |
| BP | GO:0048872 | homeostasis of number of cells | 3/37 | 0.012624 | 0.03613 | 0.015826 | BCL2/BAX/CASP3 | 3 |
| BP | GO:0000086 | G2/M transition of mitotic cell cycle | 3/37 | 0.012762 | 0.036485 | 0.015981 | CDKN1A/CDK1/CCNB1 | 3 |
| BP | GO:0014068 | positive regulation of phosphatidylinositol 3-kinase signaling | 2/37 | 0.012861 | 0.036688 | 0.01607 | KDR/PPARD | 2 |
| BP | GO:0048477 | oogenesis | 2/37 | 0.012861 | 0.036688 | 0.01607 | BCL2/CCNB1 | 2 |
| BP | GO:0046434 | organophosphate catabolic process | 3/37 | 0.012901 | 0.03676 | 0.016102 | PON1/TP53/PRKCD | 3 |
| BP | GO:0009791 | post-embryonic development | 2/37 | 0.013144 | 0.037372 | 0.01637 | BCL2/BAX | 2 |
| BP | GO:2000177 | regulation of neural precursor cell proliferation | 2/37 | 0.013144 | 0.037372 | 0.01637 | SLC6A4/TP53 | 2 |
| BP | GO:0021537 | telencephalon development | 3/37 | 0.013323 | 0.037837 | 0.016573 | DRD1/BAX/CASP3 | 3 |
| BP | GO:0055013 | cardiac muscle cell development | 2/37 | 0.01343 | 0.0381 | 0.016689 | CDK1/CCNB1 | 2 |
| BP | GO:0055074 | calcium ion homeostasis | 4/37 | 0.013682 | 0.038706 | 0.016954 | ESR1/DRD1/BCL2/BAX | 4 |
| BP | GO:0015844 | monoamine transport | 2/37 | 0.013718 | 0.038706 | 0.016954 | DRD1/SLC6A4 | 2 |
| BP | GO:0034333 | adherens junction assembly | 2/37 | 0.013718 | 0.038706 | 0.016954 | KDR/BCL2 | 2 |
| BP | GO:0046849 | bone remodeling | 2/37 | 0.013718 | 0.038706 | 0.016954 | ADRB2/PRKCA | 2 |
| BP | GO:1903035 | negative regulation of response to wounding | 2/37 | 0.013718 | 0.038706 | 0.016954 | CDKN1A/PRKCD | 2 |
| BP | GO:0014909 | smooth muscle cell migration | 2/37 | 0.014009 | 0.039441 | 0.017276 | PPARD/BCL2 | 2 |
| BP | GO:1901992 | positive regulation of mitotic cell cycle phase transition | 2/37 | 0.014009 | 0.039441 | 0.017276 | CDK1/CCNB1 | 2 |
| BP | GO:1903706 | regulation of hemopoiesis | 4/37 | 0.014075 | 0.039584 | 0.017339 | JUN/CASP8/PRKCA/MYC | 4 |
| BP | GO:2000117 | negative regulation of cysteine-type endopeptidase activity | 2/37 | 0.014302 | 0.04018 | 0.0176 | PTGS2/DPEP1 | 2 |
| BP | GO:0003073 | regulation of systemic arterial blood pressure | 2/37 | 0.014599 | 0.040835 | 0.017887 | AR/ADRB2 | 2 |
| BP | GO:0035249 | synaptic transmission, glutamatergic | 2/37 | 0.014599 | 0.040835 | 0.017887 | PTGS2/DRD1 | 2 |
| BP | GO:0036473 | cell death in response to oxidative stress | 2/37 | 0.014599 | 0.040835 | 0.017887 | BCL2/PRKCD | 2 |
| BP | GO:0072080 | nephron tubule development | 2/37 | 0.014599 | 0.040835 | 0.017887 | BCL2/MYC | 2 |
| BP | GO:0009108 | coenzyme biosynthetic process | 3/37 | 0.014785 | 0.041313 | 0.018096 | PTGS2/TP53/FASN | 3 |
| BP | GO:0070838 | divalent metal ion transport | 4/37 | 0.014883 | 0.041537 | 0.018194 | PTGS2/DRD1/BCL2/BAX | 4 |
| BP | GO:0060993 | kidney morphogenesis | 2/37 | 0.014898 | 0.041537 | 0.018194 | BCL2/MYC | 2 |
| BP | GO:0007281 | germ cell development | 3/37 | 0.014936 | 0.041601 | 0.018222 | BCL2/BAX/CCNB1 | 3 |
| BP | GO:0044070 | regulation of anion transport | 2/37 | 0.015199 | 0.042151 | 0.018463 | IL1B/PRKCD | 2 |
| BP | GO:0050810 | regulation of steroid biosynthetic process | 2/37 | 0.015199 | 0.042151 | 0.018463 | FASN/IL1B | 2 |
| BP | GO:0055006 | cardiac cell development | 2/37 | 0.015199 | 0.042151 | 0.018463 | CDK1/CCNB1 | 2 |
| BP | GO:0061326 | renal tubule development | 2/37 | 0.015199 | 0.042151 | 0.018463 | BCL2/MYC | 2 |
| BP | GO:0010660 | regulation of muscle cell apoptotic process | 2/37 | 0.015503 | 0.04287 | 0.018778 | TP53/PPARG | 2 |
| BP | GO:0048010 | vascular endothelial growth factor receptor signaling pathway | 2/37 | 0.015503 | 0.04287 | 0.018778 | KDR/IL1B | 2 |
| BP | GO:0072511 | divalent inorganic cation transport | 4/37 | 0.015508 | 0.04287 | 0.018778 | PTGS2/DRD1/BCL2/BAX | 4 |
| BP | GO:0001657 | ureteric bud development | 2/37 | 0.01581 | 0.043566 | 0.019083 | BCL2/MYC | 2 |
| BP | GO:0007044 | cell-substrate junction assembly | 2/37 | 0.01581 | 0.043566 | 0.019083 | KDR/BCL2 | 2 |
| BP | GO:0042775 | mitochondrial ATP synthesis coupled electron transport | 2/37 | 0.01581 | 0.043566 | 0.019083 | CDK1/CCNB1 | 2 |
| BP | GO:0072503 | cellular divalent inorganic cation homeostasis | 4/37 | 0.015934 | 0.04386 | 0.019212 | ESR1/DRD1/BCL2/BAX | 4 |
| BP | GO:0006836 | neurotransmitter transport | 3/37 | 0.016019 | 0.043952 | 0.019252 | DRD1/CHRM2/SLC6A4 | 3 |
| BP | GO:0007249 | I-kappaB kinase/NF-kappaB signaling | 3/37 | 0.016019 | 0.043952 | 0.019252 | ESR1/CASP8/IL1B | 3 |
| BP | GO:0002042 | cell migration involved in sprouting angiogenesis | 2/37 | 0.01612 | 0.043952 | 0.019252 | PTGS2/KDR | 2 |
| BP | GO:0042773 | ATP synthesis coupled electron transport | 2/37 | 0.01612 | 0.043952 | 0.019252 | CDK1/CCNB1 | 2 |
| BP | GO:0043473 | pigmentation | 2/37 | 0.01612 | 0.043952 | 0.019252 | BCL2/BAX | 2 |
| BP | GO:0050764 | regulation of phagocytosis | 2/37 | 0.01612 | 0.043952 | 0.019252 | IL1B/PPARG | 2 |
| BP | GO:0072163 | mesonephric epithelium development | 2/37 | 0.01612 | 0.043952 | 0.019252 | BCL2/MYC | 2 |
| BP | GO:0072164 | mesonephric tubule development | 2/37 | 0.01612 | 0.043952 | 0.019252 | BCL2/MYC | 2 |
| BP | GO:2001243 | negative regulation of intrinsic apoptotic signaling pathway | 2/37 | 0.01612 | 0.043952 | 0.019252 | PTGS2/BCL2 | 2 |
| BP | GO:0019395 | fatty acid oxidation | 2/37 | 0.016432 | 0.044756 | 0.019604 | PPARD/PPARG | 2 |
| BP | GO:0032868 | response to insulin | 3/37 | 0.016496 | 0.044884 | 0.01966 | IL1B/PRKCD/PPARG | 3 |
| BP | GO:0010657 | muscle cell apoptotic process | 2/37 | 0.016746 | 0.045422 | 0.019896 | TP53/PPARG | 2 |
| BP | GO:0030301 | cholesterol transport | 2/37 | 0.016746 | 0.045422 | 0.019896 | PON1/PPARG | 2 |
| BP | GO:0055024 | regulation of cardiac muscle tissue development | 2/37 | 0.016746 | 0.045422 | 0.019896 | CDK1/CCNB1 | 2 |
| BP | GO:0016054 | organic acid catabolic process | 3/37 | 0.016981 | 0.045963 | 0.020133 | NOS2/PPARD/PON1 | 3 |
| BP | GO:0046395 | carboxylic acid catabolic process | 3/37 | 0.016981 | 0.045963 | 0.020133 | NOS2/PPARD/PON1 | 3 |
| BP | GO:0032611 | interleukin-1 beta production | 2/37 | 0.017064 | 0.045993 | 0.020146 | CASP8/IL1B | 2 |
| BP | GO:0034440 | lipid oxidation | 2/37 | 0.017064 | 0.045993 | 0.020146 | PPARD/PPARG | 2 |
| BP | GO:1903008 | organelle disassembly | 2/37 | 0.017064 | 0.045993 | 0.020146 | TP53/CDK1 | 2 |
| BP | GO:1903426 | regulation of reactive oxygen species biosynthetic process | 2/37 | 0.017064 | 0.045993 | 0.020146 | PTGS2/IL1B | 2 |
| BP | GO:0001823 | mesonephros development | 2/37 | 0.017383 | 0.046758 | 0.020481 | BCL2/MYC | 2 |
| BP | GO:0098869 | cellular oxidant detoxification | 2/37 | 0.017383 | 0.046758 | 0.020481 | PTGS2/PTGS1 | 2 |
| BP | GO:0060485 | mesenchyme development | 3/37 | 0.017474 | 0.046955 | 0.020567 | BCL2/MYC/IL1B | 3 |
| BP | GO:0001523 | retinoid metabolic process | 2/37 | 0.018031 | 0.048299 | 0.021156 | PPARD/AKR1B1 | 2 |
| BP | GO:0014812 | muscle cell migration | 2/37 | 0.018031 | 0.048299 | 0.021156 | PPARD/BCL2 | 2 |
| BP | GO:0046928 | regulation of neurotransmitter secretion | 2/37 | 0.018031 | 0.048299 | 0.021156 | DRD1/CHRM2 | 2 |
| BP | GO:0007009 | plasma membrane organization | 2/37 | 0.018358 | 0.049024 | 0.021473 | AR/PRKCD | 2 |
| BP | GO:0033138 | positive regulation of peptidyl-serine phosphorylation | 2/37 | 0.018358 | 0.049024 | 0.021473 | PTGS2/BCL2 | 2 |
| BP | GO:0055017 | cardiac muscle tissue growth | 2/37 | 0.018358 | 0.049024 | 0.021473 | CDK1/CCNB1 | 2 |
| BP | GO:1901989 | positive regulation of cell cycle phase transition | 2/37 | 0.018688 | 0.049854 | 0.021837 | CDK1/CCNB1 | 2 |
| BP | GO:0044262 | cellular carbohydrate metabolic process | 3/37 | 0.018829 | 0.050178 | 0.021979 | NCOA2/AKR1B1/TP53 | 3 |
| BP | GO:0071824 | protein-DNA complex subunit organization | 3/37 | 0.019176 | 0.050408 | 0.02208 | ESR1/TP53/MYC | 3 |
| BP | GO:0015696 | ammonium transport | 2/37 | 0.019355 | 0.050408 | 0.02208 | DRD1/SLC6A4 | 2 |
| BP | GO:0032526 | response to retinoic acid | 2/37 | 0.019355 | 0.050408 | 0.02208 | SLC6A4/PPARG | 2 |
| BP | GO:0034330 | cell junction organization | 3/37 | 0.019527 | 0.050408 | 0.02208 | KDR/BCL2/PRKCA | 3 |
| BP | GO:0001820 | serotonin secretion | 1/37 | 0.019647 | 0.050408 | 0.02208 | SLC6A4 | 1 |
| BP | GO:0002934 | desmosome organization | 1/37 | 0.019647 | 0.050408 | 0.02208 | PRKCA | 1 |
| BP | GO:0007191 | adenylate cyclase-activating dopamine receptor signaling pathway | 1/37 | 0.019647 | 0.050408 | 0.02208 | DRD1 | 1 |
| BP | GO:0010739 | positive regulation of protein kinase A signaling | 1/37 | 0.019647 | 0.050408 | 0.02208 | ADRB2 | 1 |
| BP | GO:0014041 | regulation of neuron maturation | 1/37 | 0.019647 | 0.050408 | 0.02208 | BCL2 | 1 |
| BP | GO:0021548 | pons development | 1/37 | 0.019647 | 0.050408 | 0.02208 | BCL2 | 1 |
| BP | GO:0021924 | cell proliferation in external granule layer | 1/37 | 0.019647 | 0.050408 | 0.02208 | SLC6A4 | 1 |
| BP | GO:0021930 | cerebellar granule cell precursor proliferation | 1/37 | 0.019647 | 0.050408 | 0.02208 | SLC6A4 | 1 |
| BP | GO:0021936 | regulation of cerebellar granule cell precursor proliferation | 1/37 | 0.019647 | 0.050408 | 0.02208 | SLC6A4 | 1 |
| BP | GO:0030432 | peristalsis | 1/37 | 0.019647 | 0.050408 | 0.02208 | DRD1 | 1 |
| BP | GO:0030647 | aminoglycoside antibiotic metabolic process | 1/37 | 0.019647 | 0.050408 | 0.02208 | AKR1B1 | 1 |
| BP | GO:0031284 | positive regulation of guanylate cyclase activity | 1/37 | 0.019647 | 0.050408 | 0.02208 | NOS2 | 1 |
| BP | GO:0032308 | positive regulation of prostaglandin secretion | 1/37 | 0.019647 | 0.050408 | 0.02208 | IL1B | 1 |
| BP | GO:0043653 | mitochondrial fragmentation involved in apoptotic process | 1/37 | 0.019647 | 0.050408 | 0.02208 | BAX | 1 |
| BP | GO:0044359 | modulation of molecular function in other organism | 1/37 | 0.019647 | 0.050408 | 0.02208 | CASP8 | 1 |
| BP | GO:0045657 | positive regulation of monocyte differentiation | 1/37 | 0.019647 | 0.050408 | 0.02208 | JUN | 1 |
| BP | GO:0045713 | low-density lipoprotein particle receptor biosynthetic process | 1/37 | 0.019647 | 0.050408 | 0.02208 | PPARG | 1 |
| BP | GO:0045945 | positive regulation of transcription by RNA polymerase III | 1/37 | 0.019647 | 0.050408 | 0.02208 | AR | 1 |
| BP | GO:0046485 | ether lipid metabolic process | 1/37 | 0.019647 | 0.050408 | 0.02208 | FASN | 1 |
| BP | GO:0051901 | positive regulation of mitochondrial depolarization | 1/37 | 0.019647 | 0.050408 | 0.02208 | KDR | 1 |
| BP | GO:0052205 | modulation of molecular function in other organism involved in symbiotic interaction | 1/37 | 0.019647 | 0.050408 | 0.02208 | CASP8 | 1 |
| BP | GO:0060513 | prostatic bud formation | 1/37 | 0.019647 | 0.050408 | 0.02208 | AR | 1 |
| BP | GO:0060768 | regulation of epithelial cell proliferation involved in prostate gland development | 1/37 | 0.019647 | 0.050408 | 0.02208 | AR | 1 |
| BP | GO:0071281 | cellular response to iron ion | 1/37 | 0.019647 | 0.050408 | 0.02208 | CCNB1 | 1 |
| BP | GO:0072203 | cell proliferation involved in metanephros development | 1/37 | 0.019647 | 0.050408 | 0.02208 | MYC | 1 |
| BP | GO:0090154 | positive regulation of sphingolipid biosynthetic process | 1/37 | 0.019647 | 0.050408 | 0.02208 | PRKCD | 1 |
| BP | GO:0090336 | positive regulation of brown fat cell differentiation | 1/37 | 0.019647 | 0.050408 | 0.02208 | PTGS2 | 1 |
| BP | GO:0090557 | establishment of endothelial intestinal barrier | 1/37 | 0.019647 | 0.050408 | 0.02208 | FASN | 1 |
| BP | GO:0106049 | regulation of cellular response to osmotic stress | 1/37 | 0.019647 | 0.050408 | 0.02208 | PTGS2 | 1 |
| BP | GO:0110096 | cellular response to aldehyde | 1/37 | 0.019647 | 0.050408 | 0.02208 | AKR1B1 | 1 |
| BP | GO:1903800 | positive regulation of production of miRNAs involved in gene silencing by miRNA | 1/37 | 0.019647 | 0.050408 | 0.02208 | TP53 | 1 |
| BP | GO:2000304 | positive regulation of ceramide biosynthetic process | 1/37 | 0.019647 | 0.050408 | 0.02208 | PRKCD | 1 |
| BP | GO:2001214 | positive regulation of vasculogenesis | 1/37 | 0.019647 | 0.050408 | 0.02208 | KDR | 1 |
| BP | GO:0001676 | long-chain fatty acid metabolic process | 2/37 | 0.019693 | 0.050408 | 0.02208 | PTGS2/PTGS1 | 2 |
| BP | GO:0006289 | nucleotide-excision repair | 2/37 | 0.019693 | 0.050408 | 0.02208 | TP53/PCNA | 2 |
| BP | GO:0072009 | nephron epithelium development | 2/37 | 0.019693 | 0.050408 | 0.02208 | BCL2/MYC | 2 |
| BP | GO:0006664 | glycolipid metabolic process | 2/37 | 0.020033 | 0.051177 | 0.022417 | BAX/PRKCD | 2 |
| BP | GO:0016101 | diterpenoid metabolic process | 2/37 | 0.020033 | 0.051177 | 0.022417 | PPARD/AKR1B1 | 2 |
| BP | GO:0022037 | metencephalon development | 2/37 | 0.020375 | 0.051949 | 0.022755 | BCL2/TP53 | 2 |
| BP | GO:1903509 | liposaccharide metabolic process | 2/37 | 0.020375 | 0.051949 | 0.022755 | BAX/PRKCD | 2 |
| BP | GO:0050768 | negative regulation of neurogenesis | 3/37 | 0.020421 | 0.052015 | 0.022784 | SLC6A4/TP53/IL1B | 3 |
| BP | GO:0015918 | sterol transport | 2/37 | 0.02072 | 0.052365 | 0.022937 | PON1/PPARG | 2 |
| BP | GO:0017158 | regulation of calcium ion-dependent exocytosis | 2/37 | 0.02072 | 0.052365 | 0.022937 | DRD1/CHRM2 | 2 |
| BP | GO:0042303 | molting cycle | 2/37 | 0.02072 | 0.052365 | 0.022937 | PTGS2/BCL2 | 2 |
| BP | GO:0042633 | hair cycle | 2/37 | 0.02072 | 0.052365 | 0.022937 | PTGS2/BCL2 | 2 |
| BP | GO:0051817 | modification of morphology or physiology of other organism involved in symbiotic interaction | 2/37 | 0.02072 | 0.052365 | 0.022937 | JUN/CASP8 | 2 |
| BP | GO:0060419 | heart growth | 2/37 | 0.02072 | 0.052365 | 0.022937 | CDK1/CCNB1 | 2 |
| BP | GO:0098693 | regulation of synaptic vesicle cycle | 2/37 | 0.02072 | 0.052365 | 0.022937 | DRD1/CHRM2 | 2 |
| BP | GO:1990748 | cellular detoxification | 2/37 | 0.02072 | 0.052365 | 0.022937 | PTGS2/PTGS1 | 2 |
| BP | GO:0002758 | innate immune response-activating signal transduction | 3/37 | 0.020967 | 0.052939 | 0.023189 | ESR1/CASP8/PRKCD | 3 |
| BP | GO:0006164 | purine nucleotide biosynthetic process | 3/37 | 0.021336 | 0.05337 | 0.023377 | NOS2/TP53/FASN | 3 |
| BP | GO:0030282 | bone mineralization | 2/37 | 0.021417 | 0.05337 | 0.023377 | PTGS2/ADRB2 | 2 |
| BP | GO:0021534 | cell proliferation in hindbrain | 1/37 | 0.021591 | 0.05337 | 0.023377 | SLC6A4 | 1 |
| BP | GO:0021892 | cerebral cortex GABAergic interneuron differentiation | 1/37 | 0.021591 | 0.05337 | 0.023377 | DRD1 | 1 |
| BP | GO:0031442 | positive regulation of mRNA 3'-end processing | 1/37 | 0.021591 | 0.05337 | 0.023377 | CCNB1 | 1 |
| BP | GO:0031915 | positive regulation of synaptic plasticity | 1/37 | 0.021591 | 0.05337 | 0.023377 | PTGS2 | 1 |
| BP | GO:0032306 | regulation of prostaglandin secretion | 1/37 | 0.021591 | 0.05337 | 0.023377 | IL1B | 1 |
| BP | GO:0033148 | positive regulation of intracellular estrogen receptor signaling pathway | 1/37 | 0.021591 | 0.05337 | 0.023377 | AR | 1 |
| BP | GO:0033327 | Leydig cell differentiation | 1/37 | 0.021591 | 0.05337 | 0.023377 | AR | 1 |
| BP | GO:0033632 | regulation of cell-cell adhesion mediated by integrin | 1/37 | 0.021591 | 0.05337 | 0.023377 | DPP4 | 1 |
| BP | GO:0036462 | TRAIL-activated apoptotic signaling pathway | 1/37 | 0.021591 | 0.05337 | 0.023377 | CASP8 | 1 |
| BP | GO:0040015 | negative regulation of multicellular organism growth | 1/37 | 0.021591 | 0.05337 | 0.023377 | ADRB2 | 1 |
| BP | GO:0046007 | negative regulation of activated T cell proliferation | 1/37 | 0.021591 | 0.05337 | 0.023377 | CASP3 | 1 |
| BP | GO:0046886 | positive regulation of hormone biosynthetic process | 1/37 | 0.021591 | 0.05337 | 0.023377 | IL1B | 1 |
| BP | GO:0048742 | regulation of skeletal muscle fiber development | 1/37 | 0.021591 | 0.05337 | 0.023377 | BCL2 | 1 |
| BP | GO:0051583 | dopamine uptake involved in synaptic transmission | 1/37 | 0.021591 | 0.05337 | 0.023377 | DRD1 | 1 |
| BP | GO:0051934 | catecholamine uptake involved in synaptic transmission | 1/37 | 0.021591 | 0.05337 | 0.023377 | DRD1 | 1 |
| BP | GO:0060525 | prostate glandular acinus development | 1/37 | 0.021591 | 0.05337 | 0.023377 | ESR1 | 1 |
| BP | GO:0060767 | epithelial cell proliferation involved in prostate gland development | 1/37 | 0.021591 | 0.05337 | 0.023377 | AR | 1 |
| BP | GO:1901096 | regulation of autophagosome maturation | 1/37 | 0.021591 | 0.05337 | 0.023377 | ADRB2 | 1 |
| BP | GO:1902065 | response to L-glutamate | 1/37 | 0.021591 | 0.05337 | 0.023377 | PCNA | 1 |
| BP | GO:1990440 | positive regulation of transcription from RNA polymerase II promoter in response to endoplasmic reticulum stress | 1/37 | 0.021591 | 0.05337 | 0.023377 | TP53 | 1 |
| BP | GO:0090305 | nucleic acid phosphodiester bond hydrolysis | 3/37 | 0.021709 | 0.053611 | 0.023483 | BAX/CASP3/PCNA | 3 |
| BP | GO:0032612 | interleukin-1 production | 2/37 | 0.021769 | 0.053657 | 0.023503 | CASP8/IL1B | 2 |
| BP | GO:0032963 | collagen metabolic process | 2/37 | 0.021769 | 0.053657 | 0.023503 | PPARD/PPARG | 2 |
| BP | GO:1903037 | regulation of leukocyte cell-cell adhesion | 3/37 | 0.022085 | 0.054385 | 0.023822 | DPP4/CASP3/IL1B | 3 |
| BP | GO:0021782 | glial cell development | 2/37 | 0.022124 | 0.054428 | 0.023841 | DRD1/IL1B | 2 |
| BP | GO:0046034 | ATP metabolic process | 3/37 | 0.022275 | 0.054747 | 0.02398 | TP53/CDK1/CCNB1 | 3 |
| BP | GO:0010508 | positive regulation of autophagy | 2/37 | 0.022481 | 0.055045 | 0.024111 | KDR/ADRB2 | 2 |
| BP | GO:0010906 | regulation of glucose metabolic process | 2/37 | 0.022481 | 0.055045 | 0.024111 | NCOA2/TP53 | 2 |
| BP | GO:0022904 | respiratory electron transport chain | 2/37 | 0.022481 | 0.055045 | 0.024111 | CDK1/CCNB1 | 2 |
| BP | GO:1900371 | regulation of purine nucleotide biosynthetic process | 2/37 | 0.022481 | 0.055045 | 0.024111 | NOS2/TP53 | 2 |
| BP | GO:0030808 | regulation of nucleotide biosynthetic process | 2/37 | 0.02284 | 0.055806 | 0.024444 | NOS2/TP53 | 2 |
| BP | GO:0001952 | regulation of cell-matrix adhesion | 2/37 | 0.023202 | 0.055806 | 0.024444 | KDR/BCL2 | 2 |
| BP | GO:0016079 | synaptic vesicle exocytosis | 2/37 | 0.023202 | 0.055806 | 0.024444 | DRD1/CHRM2 | 2 |
| BP | GO:0002024 | diet induced thermogenesis | 1/37 | 0.023531 | 0.055806 | 0.024444 | ADRB2 | 1 |
| BP | GO:0002674 | negative regulation of acute inflammatory response | 1/37 | 0.023531 | 0.055806 | 0.024444 | PPARG | 1 |
| BP | GO:0006527 | arginine catabolic process | 1/37 | 0.023531 | 0.055806 | 0.024444 | NOS2 | 1 |
| BP | GO:0006662 | glycerol ether metabolic process | 1/37 | 0.023531 | 0.055806 | 0.024444 | FASN | 1 |
| BP | GO:0006983 | ER overload response | 1/37 | 0.023531 | 0.055806 | 0.024444 | TP53 | 1 |
| BP | GO:0007320 | insemination | 1/37 | 0.023531 | 0.055806 | 0.024444 | SLC6A4 | 1 |
| BP | GO:0009886 | post-embryonic animal morphogenesis | 1/37 | 0.023531 | 0.055806 | 0.024444 | BAX | 1 |
| BP | GO:0010889 | regulation of sequestering of triglyceride | 1/37 | 0.023531 | 0.055806 | 0.024444 | PPARG | 1 |
| BP | GO:0031282 | regulation of guanylate cyclase activity | 1/37 | 0.023531 | 0.055806 | 0.024444 | NOS2 | 1 |
| BP | GO:0033197 | response to vitamin E | 1/37 | 0.023531 | 0.055806 | 0.024444 | PPARG | 1 |
| BP | GO:0042368 | vitamin D biosynthetic process | 1/37 | 0.023531 | 0.055806 | 0.024444 | IL1B | 1 |
| BP | GO:0042659 | regulation of cell fate specification | 1/37 | 0.023531 | 0.055806 | 0.024444 | AR | 1 |
| BP | GO:0043922 | negative regulation by host of viral transcription | 1/37 | 0.023531 | 0.055806 | 0.024444 | JUN | 1 |
| BP | GO:0045080 | positive regulation of chemokine biosynthetic process | 1/37 | 0.023531 | 0.055806 | 0.024444 | IL1B | 1 |
| BP | GO:0051095 | regulation of helicase activity | 1/37 | 0.023531 | 0.055806 | 0.024444 | TP53 | 1 |
| BP | GO:0051988 | regulation of attachment of spindle microtubules to kinetochore | 1/37 | 0.023531 | 0.055806 | 0.024444 | CCNB1 | 1 |
| BP | GO:0060442 | branching involved in prostate gland morphogenesis | 1/37 | 0.023531 | 0.055806 | 0.024444 | ESR1 | 1 |
| BP | GO:0060742 | epithelial cell differentiation involved in prostate gland development | 1/37 | 0.023531 | 0.055806 | 0.024444 | AR | 1 |
| BP | GO:0070243 | regulation of thymocyte apoptotic process | 1/37 | 0.023531 | 0.055806 | 0.024444 | TP53 | 1 |
| BP | GO:0070486 | leukocyte aggregation | 1/37 | 0.023531 | 0.055806 | 0.024444 | IL1B | 1 |
| BP | GO:0071472 | cellular response to salt stress | 1/37 | 0.023531 | 0.055806 | 0.024444 | AKR1B1 | 1 |
| BP | GO:0071639 | positive regulation of monocyte chemotactic protein-1 production | 1/37 | 0.023531 | 0.055806 | 0.024444 | IL1B | 1 |
| BP | GO:0072520 | seminiferous tubule development | 1/37 | 0.023531 | 0.055806 | 0.024444 | AR | 1 |
| BP | GO:0090331 | negative regulation of platelet aggregation | 1/37 | 0.023531 | 0.055806 | 0.024444 | PRKCD | 1 |
| BP | GO:0097201 | negative regulation of transcription from RNA polymerase II promoter in response to stress | 1/37 | 0.023531 | 0.055806 | 0.024444 | JUN | 1 |
| BP | GO:1900402 | regulation of carbohydrate metabolic process by regulation of transcription from RNA polymerase II promoter | 1/37 | 0.023531 | 0.055806 | 0.024444 | TP53 | 1 |
| BP | GO:1902337 | regulation of apoptotic process involved in morphogenesis | 1/37 | 0.023531 | 0.055806 | 0.024444 | BAX | 1 |
| BP | GO:1902510 | regulation of apoptotic DNA fragmentation | 1/37 | 0.023531 | 0.055806 | 0.024444 | BAX | 1 |
| BP | GO:1903894 | regulation of IRE1-mediated unfolded protein response | 1/37 | 0.023531 | 0.055806 | 0.024444 | BAX | 1 |
| BP | GO:1904181 | positive regulation of membrane depolarization | 1/37 | 0.023531 | 0.055806 | 0.024444 | KDR | 1 |
| BP | GO:1905461 | positive regulation of vascular associated smooth muscle cell apoptotic process | 1/37 | 0.023531 | 0.055806 | 0.024444 | PPARG | 1 |
| BP | GO:0003014 | renal system process | 2/37 | 0.023566 | 0.055806 | 0.024444 | AKR1B1/BCL2 | 2 |
| BP | GO:0006721 | terpenoid metabolic process | 2/37 | 0.023566 | 0.055806 | 0.024444 | PPARD/AKR1B1 | 2 |
| BP | GO:0034612 | response to tumor necrosis factor | 3/37 | 0.023626 | 0.055896 | 0.024483 | PTGS2/CASP3/CASP8 | 3 |
| BP | GO:0072522 | purine-containing compound biosynthetic process | 3/37 | 0.023822 | 0.056309 | 0.024665 | NOS2/TP53/FASN | 3 |
| BP | GO:0043618 | regulation of transcription from RNA polymerase II promoter in response to stress | 2/37 | 0.023933 | 0.056467 | 0.024734 | JUN/TP53 | 2 |
| BP | GO:0046718 | viral entry into host cell | 2/37 | 0.023933 | 0.056467 | 0.024734 | DPP4/CDK1 | 2 |
| BP | GO:0050863 | regulation of T cell activation | 3/37 | 0.02402 | 0.056621 | 0.024801 | DPP4/CASP3/IL1B | 3 |
| BP | GO:0031098 | stress-activated protein kinase signaling cascade | 3/37 | 0.024218 | 0.056985 | 0.024961 | AKR1B1/MYC/IL1B | 3 |
| BP | GO:0051961 | negative regulation of nervous system development | 3/37 | 0.024218 | 0.056985 | 0.024961 | SLC6A4/TP53/IL1B | 3 |
| BP | GO:0043500 | muscle adaptation | 2/37 | 0.024302 | 0.05713 | 0.025024 | PRKCA/IL1B | 2 |
| BP | GO:0051928 | positive regulation of calcium ion transport | 2/37 | 0.024673 | 0.05795 | 0.025383 | DRD1/BAX | 2 |
| BP | GO:0002218 | activation of innate immune response | 3/37 | 0.025021 | 0.057978 | 0.025395 | ESR1/CASP8/PRKCD | 3 |
| BP | GO:0014066 | regulation of phosphatidylinositol 3-kinase signaling | 2/37 | 0.025046 | 0.057978 | 0.025395 | KDR/PPARD | 2 |
| BP | GO:0019218 | regulation of steroid metabolic process | 2/37 | 0.025046 | 0.057978 | 0.025395 | FASN/IL1B | 2 |
| BP | GO:0046717 | acid secretion | 2/37 | 0.025046 | 0.057978 | 0.025395 | NOS2/IL1B | 2 |
| BP | GO:0048284 | organelle fusion | 2/37 | 0.025046 | 0.057978 | 0.025395 | BAX/CDK1 | 2 |
| BP | GO:0051101 | regulation of DNA binding | 2/37 | 0.025046 | 0.057978 | 0.025395 | JUN/PPARG | 2 |
| BP | GO:0051209 | release of sequestered calcium ion into cytosol | 2/37 | 0.025046 | 0.057978 | 0.025395 | DRD1/BAX | 2 |
| BP | GO:0006805 | xenobiotic metabolic process | 2/37 | 0.025422 | 0.057978 | 0.025395 | PTGS1/DPEP1 | 2 |
| BP | GO:0045471 | response to ethanol | 2/37 | 0.025422 | 0.057978 | 0.025395 | CASP8/CDK1 | 2 |
| BP | GO:0071375 | cellular response to peptide hormone stimulus | 3/37 | 0.025428 | 0.057978 | 0.025395 | IL1B/PRKCD/PPARG | 3 |
| BP | GO:0007171 | activation of transmembrane receptor protein tyrosine kinase activity | 1/37 | 0.025467 | 0.057978 | 0.025395 | ADRB2 | 1 |
| BP | GO:0010745 | negative regulation of macrophage derived foam cell differentiation | 1/37 | 0.025467 | 0.057978 | 0.025395 | PPARG | 1 |
| BP | GO:0014842 | regulation of skeletal muscle satellite cell proliferation | 1/37 | 0.025467 | 0.057978 | 0.025395 | PPARD | 1 |
| BP | GO:0030656 | regulation of vitamin metabolic process | 1/37 | 0.025467 | 0.057978 | 0.025395 | IL1B | 1 |
| BP | GO:0031953 | negative regulation of protein autophosphorylation | 1/37 | 0.025467 | 0.057978 | 0.025395 | JUN | 1 |
| BP | GO:0032725 | positive regulation of granulocyte macrophage colony-stimulating factor production | 1/37 | 0.025467 | 0.057978 | 0.025395 | IL1B | 1 |
| BP | GO:0035641 | locomotory exploration behavior | 1/37 | 0.025467 | 0.057978 | 0.025395 | DPP4 | 1 |
| BP | GO:0042415 | norepinephrine metabolic process | 1/37 | 0.025467 | 0.057978 | 0.025395 | AKR1B1 | 1 |
| BP | GO:0042538 | hyperosmotic salinity response | 1/37 | 0.025467 | 0.057978 | 0.025395 | AKR1B1 | 1 |
| BP | GO:0042711 | maternal behavior | 1/37 | 0.025467 | 0.057978 | 0.025395 | DRD1 | 1 |
| BP | GO:0043568 | positive regulation of insulin-like growth factor receptor signaling pathway | 1/37 | 0.025467 | 0.057978 | 0.025395 | AR | 1 |
| BP | GO:0045086 | positive regulation of interleukin-2 biosynthetic process | 1/37 | 0.025467 | 0.057978 | 0.025395 | IL1B | 1 |
| BP | GO:0047484 | regulation of response to osmotic stress | 1/37 | 0.025467 | 0.057978 | 0.025395 | PTGS2 | 1 |
| BP | GO:0050872 | white fat cell differentiation | 1/37 | 0.025467 | 0.057978 | 0.025395 | PPARG | 1 |
| BP | GO:0060100 | positive regulation of phagocytosis, engulfment | 1/37 | 0.025467 | 0.057978 | 0.025395 | PPARG | 1 |
| BP | GO:0060576 | intestinal epithelial cell development | 1/37 | 0.025467 | 0.057978 | 0.025395 | CDKN1A | 1 |
| BP | GO:0060601 | lateral sprouting from an epithelium | 1/37 | 0.025467 | 0.057978 | 0.025395 | AR | 1 |
| BP | GO:0060746 | parental behavior | 1/37 | 0.025467 | 0.057978 | 0.025395 | DRD1 | 1 |
| BP | GO:0061029 | eyelid development in camera-type eye | 1/37 | 0.025467 | 0.057978 | 0.025395 | JUN | 1 |
| BP | GO:0072044 | collecting duct development | 1/37 | 0.025467 | 0.057978 | 0.025395 | AKR1B1 | 1 |
| BP | GO:1900103 | positive regulation of endoplasmic reticulum unfolded protein response | 1/37 | 0.025467 | 0.057978 | 0.025395 | BAX | 1 |
| BP | GO:1901550 | regulation of endothelial cell development | 1/37 | 0.025467 | 0.057978 | 0.025395 | IL1B | 1 |
| BP | GO:1902894 | negative regulation of pri-miRNA transcription by RNA polymerase II | 1/37 | 0.025467 | 0.057978 | 0.025395 | PPARD | 1 |
| BP | GO:1903140 | regulation of establishment of endothelial barrier | 1/37 | 0.025467 | 0.057978 | 0.025395 | IL1B | 1 |
| BP | GO:1905155 | positive regulation of membrane invagination | 1/37 | 0.025467 | 0.057978 | 0.025395 | PPARG | 1 |
| BP | GO:0051283 | negative regulation of sequestering of calcium ion | 2/37 | 0.025801 | 0.058685 | 0.025705 | DRD1/BAX | 2 |
| BP | GO:1903311 | regulation of mRNA metabolic process | 3/37 | 0.026044 | 0.059187 | 0.025925 | PRKCA/PRKCD/CCNB1 | 3 |
| BP | GO:0043620 | regulation of DNA-templated transcription in response to stress | 2/37 | 0.026181 | 0.059446 | 0.026039 | JUN/TP53 | 2 |
| BP | GO:0009615 | response to virus | 3/37 | 0.02646 | 0.059974 | 0.02627 | CHRM2/BCL2/IL1B | 3 |
| BP | GO:0051188 | cofactor biosynthetic process | 3/37 | 0.02646 | 0.059974 | 0.02627 | PTGS2/TP53/FASN | 3 |
| BP | GO:0051282 | regulation of sequestering of calcium ion | 2/37 | 0.026564 | 0.060157 | 0.02635 | DRD1/BAX | 2 |
| BP | GO:0071103 | DNA conformation change | 3/37 | 0.026669 | 0.060261 | 0.026396 | TP53/CDK1/CCNB1 | 3 |
| BP | GO:0019058 | viral life cycle | 3/37 | 0.026879 | 0.060261 | 0.026396 | DPP4/BCL2/CDK1 | 3 |
| BP | GO:0034754 | cellular hormone metabolic process | 2/37 | 0.026949 | 0.060261 | 0.026396 | AKR1B1/ESR1 | 2 |
| BP | GO:0050853 | B cell receptor signaling pathway | 2/37 | 0.026949 | 0.060261 | 0.026396 | BCL2/BAX | 2 |
| BP | GO:0006684 | sphingomyelin metabolic process | 1/37 | 0.0274 | 0.060261 | 0.026396 | PRKCD | 1 |
| BP | GO:0010248 | establishment or maintenance of transmembrane electrochemical gradient | 1/37 | 0.0274 | 0.060261 | 0.026396 | BAX | 1 |
| BP | GO:0010917 | negative regulation of mitochondrial membrane potential | 1/37 | 0.0274 | 0.060261 | 0.026396 | BAX | 1 |
| BP | GO:0014841 | skeletal muscle satellite cell proliferation | 1/37 | 0.0274 | 0.060261 | 0.026396 | PPARD | 1 |
| BP | GO:0014857 | regulation of skeletal muscle cell proliferation | 1/37 | 0.0274 | 0.060261 | 0.026396 | PPARD | 1 |
| BP | GO:0030213 | hyaluronan biosynthetic process | 1/37 | 0.0274 | 0.060261 | 0.026396 | IL1B | 1 |
| BP | GO:0034356 | NAD biosynthesis via nicotinamide riboside salvage pathway | 1/37 | 0.0274 | 0.060261 | 0.026396 | PTGS2 | 1 |
| BP | GO:0035112 | genitalia morphogenesis | 1/37 | 0.0274 | 0.060261 | 0.026396 | AR | 1 |
| BP | GO:0036295 | cellular response to increased oxygen levels | 1/37 | 0.0274 | 0.060261 | 0.026396 | PPARG | 1 |
| BP | GO:0043374 | CD8-positive, alpha-beta T cell differentiation | 1/37 | 0.0274 | 0.060261 | 0.026396 | BCL2 | 1 |
| BP | GO:0046479 | glycosphingolipid catabolic process | 1/37 | 0.0274 | 0.060261 | 0.026396 | PRKCD | 1 |
| BP | GO:0048308 | organelle inheritance | 1/37 | 0.0274 | 0.060261 | 0.026396 | CDK1 | 1 |
| BP | GO:0048313 | Golgi inheritance | 1/37 | 0.0274 | 0.060261 | 0.026396 | CDK1 | 1 |
| BP | GO:0050667 | homocysteine metabolic process | 1/37 | 0.0274 | 0.060261 | 0.026396 | DPEP1 | 1 |
| BP | GO:0051712 | positive regulation of killing of cells of other organism | 1/37 | 0.0274 | 0.060261 | 0.026396 | NOS2 | 1 |
| BP | GO:0070234 | positive regulation of T cell apoptotic process | 1/37 | 0.0274 | 0.060261 | 0.026396 | TP53 | 1 |
| BP | GO:0071391 | cellular response to estrogen stimulus | 1/37 | 0.0274 | 0.060261 | 0.026396 | ESR1 | 1 |
| BP | GO:0072216 | positive regulation of metanephros development | 1/37 | 0.0274 | 0.060261 | 0.026396 | MYC | 1 |
| BP | GO:0090231 | regulation of spindle checkpoint | 1/37 | 0.0274 | 0.060261 | 0.026396 | CCNB1 | 1 |
| BP | GO:0090266 | regulation of mitotic cell cycle spindle assembly checkpoint | 1/37 | 0.0274 | 0.060261 | 0.026396 | CCNB1 | 1 |
| BP | GO:0097154 | GABAergic neuron differentiation | 1/37 | 0.0274 | 0.060261 | 0.026396 | DRD1 | 1 |
| BP | GO:0097202 | activation of cysteine-type endopeptidase activity | 1/37 | 0.0274 | 0.060261 | 0.026396 | CASP8 | 1 |
| BP | GO:0106070 | regulation of adenylate cyclase-activating G protein-coupled receptor signaling pathway | 1/37 | 0.0274 | 0.060261 | 0.026396 | PRKCA | 1 |
| BP | GO:1901722 | regulation of cell proliferation involved in kidney development | 1/37 | 0.0274 | 0.060261 | 0.026396 | MYC | 1 |
| BP | GO:1903054 | negative regulation of extracellular matrix organization | 1/37 | 0.0274 | 0.060261 | 0.026396 | DPP4 | 1 |
| BP | GO:1903351 | cellular response to dopamine | 1/37 | 0.0274 | 0.060261 | 0.026396 | DRD1 | 1 |
| BP | GO:1903504 | regulation of mitotic spindle checkpoint | 1/37 | 0.0274 | 0.060261 | 0.026396 | CCNB1 | 1 |
| BP | GO:1903624 | regulation of DNA catabolic process | 1/37 | 0.0274 | 0.060261 | 0.026396 | BAX | 1 |
| BP | GO:1904748 | regulation of apoptotic process involved in development | 1/37 | 0.0274 | 0.060261 | 0.026396 | BAX | 1 |
| BP | GO:2001212 | regulation of vasculogenesis | 1/37 | 0.0274 | 0.060261 | 0.026396 | KDR | 1 |
| BP | GO:0051208 | sequestering of calcium ion | 2/37 | 0.027726 | 0.060875 | 0.026665 | DRD1/BAX | 2 |
| BP | GO:0098754 | detoxification | 2/37 | 0.027726 | 0.060875 | 0.026665 | PTGS2/PTGS1 | 2 |
| BP | GO:0016042 | lipid catabolic process | 3/37 | 0.027943 | 0.0613 | 0.026851 | PPARD/IL1B/PRKCD | 3 |
| BP | GO:0045995 | regulation of embryonic development | 2/37 | 0.028118 | 0.061579 | 0.026973 | AR/CDK1 | 2 |
| BP | GO:0055007 | cardiac muscle cell differentiation | 2/37 | 0.028118 | 0.061579 | 0.026973 | CDK1/CCNB1 | 2 |
| BP | GO:0009205 | purine ribonucleoside triphosphate metabolic process | 3/37 | 0.028375 | 0.062089 | 0.027196 | TP53/CDK1/CCNB1 | 3 |
| BP | GO:0007596 | blood coagulation | 3/37 | 0.028592 | 0.062132 | 0.027215 | F7/PRKCA/PRKCD | 3 |
| BP | GO:0007159 | leukocyte cell-cell adhesion | 3/37 | 0.02881 | 0.062132 | 0.027215 | DPP4/CASP3/IL1B | 3 |
| BP | GO:0043405 | regulation of MAP kinase activity | 3/37 | 0.02881 | 0.062132 | 0.027215 | CDK1/IL1B/PRKCD | 3 |
| BP | GO:0030260 | entry into host cell | 2/37 | 0.028909 | 0.062132 | 0.027215 | DPP4/CDK1 | 2 |
| BP | GO:0044409 | entry into host | 2/37 | 0.028909 | 0.062132 | 0.027215 | DPP4/CDK1 | 2 |
| BP | GO:0051806 | entry into cell of other organism involved in symbiotic interaction | 2/37 | 0.028909 | 0.062132 | 0.027215 | DPP4/CDK1 | 2 |
| BP | GO:0051828 | entry into other organism involved in symbiotic interaction | 2/37 | 0.028909 | 0.062132 | 0.027215 | DPP4/CDK1 | 2 |
| BP | GO:0001889 | liver development | 2/37 | 0.029307 | 0.062132 | 0.027215 | JUN/PCNA | 2 |
| BP | GO:0050921 | positive regulation of chemotaxis | 2/37 | 0.029307 | 0.062132 | 0.027215 | F7/KDR | 2 |
| BP | GO:0006000 | fructose metabolic process | 1/37 | 0.029329 | 0.062132 | 0.027215 | AKR1B1 | 1 |
| BP | GO:0009415 | response to water | 1/37 | 0.029329 | 0.062132 | 0.027215 | AKR1B1 | 1 |
| BP | GO:0014856 | skeletal muscle cell proliferation | 1/37 | 0.029329 | 0.062132 | 0.027215 | PPARD | 1 |
| BP | GO:0015671 | oxygen transport | 1/37 | 0.029329 | 0.062132 | 0.027215 | MYC | 1 |
| BP | GO:0017014 | protein nitrosylation | 1/37 | 0.029329 | 0.062132 | 0.027215 | NOS2 | 1 |
| BP | GO:0018119 | peptidyl-cysteine S-nitrosylation | 1/37 | 0.029329 | 0.062132 | 0.027215 | NOS2 | 1 |
| BP | GO:0030238 | male sex determination | 1/37 | 0.029329 | 0.062132 | 0.027215 | AR | 1 |
| BP | GO:0030812 | negative regulation of nucleotide catabolic process | 1/37 | 0.029329 | 0.062132 | 0.027215 | TP53 | 1 |
| BP | GO:0032352 | positive regulation of hormone metabolic process | 1/37 | 0.029329 | 0.062132 | 0.027215 | IL1B | 1 |
| BP | GO:0032645 | regulation of granulocyte macrophage colony-stimulating factor production | 1/37 | 0.029329 | 0.062132 | 0.027215 | IL1B | 1 |
| BP | GO:0033689 | negative regulation of osteoblast proliferation | 1/37 | 0.029329 | 0.062132 | 0.027215 | BCL2 | 1 |
| BP | GO:0034111 | negative regulation of homotypic cell-cell adhesion | 1/37 | 0.029329 | 0.062132 | 0.027215 | PRKCD | 1 |
| BP | GO:0042362 | fat-soluble vitamin biosynthetic process | 1/37 | 0.029329 | 0.062132 | 0.027215 | IL1B | 1 |
| BP | GO:0045073 | regulation of chemokine biosynthetic process | 1/37 | 0.029329 | 0.062132 | 0.027215 | IL1B | 1 |
| BP | GO:0045410 | positive regulation of interleukin-6 biosynthetic process | 1/37 | 0.029329 | 0.062132 | 0.027215 | IL1B | 1 |
| BP | GO:0045475 | locomotor rhythm | 1/37 | 0.029329 | 0.062132 | 0.027215 | NCOA2 | 1 |
| BP | GO:0045837 | negative regulation of membrane potential | 1/37 | 0.029329 | 0.062132 | 0.027215 | BAX | 1 |
| BP | GO:0046321 | positive regulation of fatty acid oxidation | 1/37 | 0.029329 | 0.062132 | 0.027215 | PPARG | 1 |
| BP | GO:0048569 | post-embryonic animal organ development | 1/37 | 0.029329 | 0.062132 | 0.027215 | BAX | 1 |
| BP | GO:0051044 | positive regulation of membrane protein ectodomain proteolysis | 1/37 | 0.029329 | 0.062132 | 0.027215 | IL1B | 1 |
| BP | GO:0051198 | negative regulation of coenzyme metabolic process | 1/37 | 0.029329 | 0.062132 | 0.027215 | TP53 | 1 |
| BP | GO:0051770 | positive regulation of nitric-oxide synthase biosynthetic process | 1/37 | 0.029329 | 0.062132 | 0.027215 | KDR | 1 |
| BP | GO:0060099 | regulation of phagocytosis, engulfment | 1/37 | 0.029329 | 0.062132 | 0.027215 | PPARG | 1 |
| BP | GO:0060134 | prepulse inhibition | 1/37 | 0.029329 | 0.062132 | 0.027215 | DRD1 | 1 |
| BP | GO:0071474 | cellular hyperosmotic response | 1/37 | 0.029329 | 0.062132 | 0.027215 | AKR1B1 | 1 |
| BP | GO:0072075 | metanephric mesenchyme development | 1/37 | 0.029329 | 0.062132 | 0.027215 | MYC | 1 |
| BP | GO:0090141 | positive regulation of mitochondrial fission | 1/37 | 0.029329 | 0.062132 | 0.027215 | KDR | 1 |
| BP | GO:0090494 | dopamine uptake | 1/37 | 0.029329 | 0.062132 | 0.027215 | DRD1 | 1 |
| BP | GO:1903350 | response to dopamine | 1/37 | 0.029329 | 0.062132 | 0.027215 | DRD1 | 1 |
| BP | GO:2001028 | positive regulation of endothelial cell chemotaxis | 1/37 | 0.029329 | 0.062132 | 0.027215 | KDR | 1 |
| BP | GO:0009167 | purine ribonucleoside monophosphate metabolic process | 3/37 | 0.02947 | 0.06238 | 0.027324 | TP53/CDK1/CCNB1 | 3 |
| BP | GO:0007599 | hemostasis | 3/37 | 0.029692 | 0.062696 | 0.027462 | F7/PRKCA/PRKCD | 3 |
| BP | GO:0009126 | purine nucleoside monophosphate metabolic process | 3/37 | 0.029692 | 0.062696 | 0.027462 | TP53/CDK1/CCNB1 | 3 |
| BP | GO:0009199 | ribonucleoside triphosphate metabolic process | 3/37 | 0.029692 | 0.062696 | 0.027462 | TP53/CDK1/CCNB1 | 3 |
| BP | GO:0009144 | purine nucleoside triphosphate metabolic process | 3/37 | 0.029914 | 0.063012 | 0.027601 | TP53/CDK1/CCNB1 | 3 |
| BP | GO:0050817 | coagulation | 3/37 | 0.029914 | 0.063012 | 0.027601 | F7/PRKCA/PRKCD | 3 |
| BP | GO:1904062 | regulation of cation transmembrane transport | 3/37 | 0.029914 | 0.063012 | 0.027601 | DRD1/ADRB2/BAX | 3 |
| BP | GO:0034605 | cellular response to heat | 2/37 | 0.030111 | 0.063324 | 0.027737 | PTGS2/CDKN1A | 2 |
| BP | GO:1903670 | regulation of sprouting angiogenesis | 2/37 | 0.030111 | 0.063324 | 0.027737 | PTGS2/KDR | 2 |
| BP | GO:0061008 | hepaticobiliary system development | 2/37 | 0.030516 | 0.064124 | 0.028088 | JUN/PCNA | 2 |
| BP | GO:0002831 | regulation of response to biotic stimulus | 2/37 | 0.030924 | 0.064217 | 0.028128 | PRKCA/IL1B | 2 |
| BP | GO:0006720 | isoprenoid metabolic process | 2/37 | 0.030924 | 0.064217 | 0.028128 | PPARD/AKR1B1 | 2 |
| BP | GO:0006837 | serotonin transport | 1/37 | 0.031254 | 0.064217 | 0.028128 | SLC6A4 | 1 |
| BP | GO:0010225 | response to UV-C | 1/37 | 0.031254 | 0.064217 | 0.028128 | TP53 | 1 |
| BP | GO:0010875 | positive regulation of cholesterol efflux | 1/37 | 0.031254 | 0.064217 | 0.028128 | PON1 | 1 |
| BP | GO:0019377 | glycolipid catabolic process | 1/37 | 0.031254 | 0.064217 | 0.028128 | PRKCD | 1 |
| BP | GO:0021542 | dentate gyrus development | 1/37 | 0.031254 | 0.064217 | 0.028128 | DRD1 | 1 |
| BP | GO:0030889 | negative regulation of B cell proliferation | 1/37 | 0.031254 | 0.064217 | 0.028128 | CASP3 | 1 |
| BP | GO:0032305 | positive regulation of icosanoid secretion | 1/37 | 0.031254 | 0.064217 | 0.028128 | IL1B | 1 |
| BP | GO:0032604 | granulocyte macrophage colony-stimulating factor production | 1/37 | 0.031254 | 0.064217 | 0.028128 | IL1B | 1 |
| BP | GO:0033033 | negative regulation of myeloid cell apoptotic process | 1/37 | 0.031254 | 0.064217 | 0.028128 | BCL2 | 1 |
| BP | GO:0033631 | cell-cell adhesion mediated by integrin | 1/37 | 0.031254 | 0.064217 | 0.028128 | DPP4 | 1 |
| BP | GO:0034116 | positive regulation of heterotypic cell-cell adhesion | 1/37 | 0.031254 | 0.064217 | 0.028128 | IL1B | 1 |
| BP | GO:0042033 | chemokine biosynthetic process | 1/37 | 0.031254 | 0.064217 | 0.028128 | IL1B | 1 |
| BP | GO:0044849 | estrous cycle | 1/37 | 0.031254 | 0.064217 | 0.028128 | PCNA | 1 |
| BP | GO:0050755 | chemokine metabolic process | 1/37 | 0.031254 | 0.064217 | 0.028128 | IL1B | 1 |
| BP | GO:0060572 | morphogenesis of an epithelial bud | 1/37 | 0.031254 | 0.064217 | 0.028128 | AR | 1 |
| BP | GO:0071380 | cellular response to prostaglandin E stimulus | 1/37 | 0.031254 | 0.064217 | 0.028128 | PPARG | 1 |
| BP | GO:0071732 | cellular response to nitric oxide | 1/37 | 0.031254 | 0.064217 | 0.028128 | DPEP1 | 1 |
| BP | GO:0090153 | regulation of sphingolipid biosynthetic process | 1/37 | 0.031254 | 0.064217 | 0.028128 | PRKCD | 1 |
| BP | GO:0090335 | regulation of brown fat cell differentiation | 1/37 | 0.031254 | 0.064217 | 0.028128 | PTGS2 | 1 |
| BP | GO:0090493 | catecholamine uptake | 1/37 | 0.031254 | 0.064217 | 0.028128 | DRD1 | 1 |
| BP | GO:1900451 | positive regulation of glutamate receptor signaling pathway | 1/37 | 0.031254 | 0.064217 | 0.028128 | ADRB2 | 1 |
| BP | GO:1902004 | positive regulation of amyloid-beta formation | 1/37 | 0.031254 | 0.064217 | 0.028128 | CASP3 | 1 |
| BP | GO:1902166 | negative regulation of intrinsic apoptotic signaling pathway in response to DNA damage by p53 class mediator | 1/37 | 0.031254 | 0.064217 | 0.028128 | BCL2 | 1 |
| BP | GO:1905038 | regulation of membrane lipid metabolic process | 1/37 | 0.031254 | 0.064217 | 0.028128 | PRKCD | 1 |
| BP | GO:1905153 | regulation of membrane invagination | 1/37 | 0.031254 | 0.064217 | 0.028128 | PPARG | 1 |
| BP | GO:2000303 | regulation of ceramide biosynthetic process | 1/37 | 0.031254 | 0.064217 | 0.028128 | PRKCD | 1 |
| BP | GO:0072073 | kidney epithelium development | 2/37 | 0.031333 | 0.064278 | 0.028155 | BCL2/MYC | 2 |
| BP | GO:0097553 | calcium ion transmembrane import into cytosol | 2/37 | 0.031333 | 0.064278 | 0.028155 | DRD1/BAX | 2 |
| BP | GO:0008286 | insulin receptor signaling pathway | 2/37 | 0.031745 | 0.065071 | 0.028503 | IL1B/PRKCD | 2 |
| BP | GO:0051260 | protein homooligomerization | 3/37 | 0.031958 | 0.065456 | 0.028671 | SLC6A4/BAX/TP53 | 3 |
| BP | GO:0010675 | regulation of cellular carbohydrate metabolic process | 2/37 | 0.032159 | 0.06566 | 0.028761 | NCOA2/TP53 | 2 |
| BP | GO:0034332 | adherens junction organization | 2/37 | 0.032159 | 0.06566 | 0.028761 | KDR/BCL2 | 2 |
| BP | GO:0050871 | positive regulation of B cell activation | 2/37 | 0.032159 | 0.06566 | 0.028761 | BCL2/CDKN1A | 2 |
| BP | GO:0072006 | nephron development | 2/37 | 0.032159 | 0.06566 | 0.028761 | BCL2/MYC | 2 |
| BP | GO:0030098 | lymphocyte differentiation | 3/37 | 0.032422 | 0.066145 | 0.028973 | BCL2/BAX/TP53 | 3 |
| BP | GO:0009161 | ribonucleoside monophosphate metabolic process | 3/37 | 0.032655 | 0.066516 | 0.029136 | TP53/CDK1/CCNB1 | 3 |
| BP | GO:0031589 | cell-substrate adhesion | 3/37 | 0.032655 | 0.066516 | 0.029136 | KDR/PPARD/BCL2 | 3 |
| BP | GO:0006089 | lactate metabolic process | 1/37 | 0.033176 | 0.066685 | 0.02921 | TP53 | 1 |
| BP | GO:0008340 | determination of adult lifespan | 1/37 | 0.033176 | 0.066685 | 0.02921 | TP53 | 1 |
| BP | GO:0010715 | regulation of extracellular matrix disassembly | 1/37 | 0.033176 | 0.066685 | 0.02921 | DPP4 | 1 |
| BP | GO:0017085 | response to insecticide | 1/37 | 0.033176 | 0.066685 | 0.02921 | CCNB1 | 1 |
| BP | GO:0018904 | ether metabolic process | 1/37 | 0.033176 | 0.066685 | 0.02921 | FASN | 1 |
| BP | GO:0031000 | response to caffeine | 1/37 | 0.033176 | 0.066685 | 0.02921 | PPARG | 1 |
| BP | GO:0033145 | positive regulation of intracellular steroid hormone receptor signaling pathway | 1/37 | 0.033176 | 0.066685 | 0.02921 | AR | 1 |
| BP | GO:0033599 | regulation of mammary gland epithelial cell proliferation | 1/37 | 0.033176 | 0.066685 | 0.02921 | BAX | 1 |
| BP | GO:0034501 | protein localization to kinetochore | 1/37 | 0.033176 | 0.066685 | 0.02921 | CDK1 | 1 |
| BP | GO:0036270 | response to diuretic | 1/37 | 0.033176 | 0.066685 | 0.02921 | PPARG | 1 |
| BP | GO:0042953 | lipoprotein transport | 1/37 | 0.033176 | 0.066685 | 0.02921 | PPARG | 1 |
| BP | GO:0043923 | positive regulation by host of viral transcription | 1/37 | 0.033176 | 0.066685 | 0.02921 | JUN | 1 |
| BP | GO:0044872 | lipoprotein localization | 1/37 | 0.033176 | 0.066685 | 0.02921 | PPARG | 1 |
| BP | GO:0051709 | regulation of killing of cells of other organism | 1/37 | 0.033176 | 0.066685 | 0.02921 | NOS2 | 1 |
| BP | GO:0060850 | regulation of transcription involved in cell fate commitment | 1/37 | 0.033176 | 0.066685 | 0.02921 | PPARG | 1 |
| BP | GO:1901524 | regulation of mitophagy | 1/37 | 0.033176 | 0.066685 | 0.02921 | TP53 | 1 |
| BP | GO:2000811 | negative regulation of anoikis | 1/37 | 0.033176 | 0.066685 | 0.02921 | BCL2 | 1 |
| BP | GO:0022412 | cellular process involved in reproduction in multicellular organism | 3/37 | 0.033361 | 0.067005 | 0.02935 | BCL2/BAX/CCNB1 | 3 |
| BP | GO:0006119 | oxidative phosphorylation | 2/37 | 0.033414 | 0.06706 | 0.029374 | CDK1/CCNB1 | 2 |
| BP | GO:0002224 | toll-like receptor signaling pathway | 2/37 | 0.033836 | 0.067803 | 0.029699 | ESR1/CASP8 | 2 |
| BP | GO:2000241 | regulation of reproductive process | 2/37 | 0.033836 | 0.067803 | 0.029699 | AR/ESR1 | 2 |
| BP | GO:0002262 | myeloid cell homeostasis | 2/37 | 0.034261 | 0.068442 | 0.029979 | BAX/CASP3 | 2 |
| BP | GO:0019359 | nicotinamide nucleotide biosynthetic process | 2/37 | 0.034261 | 0.068442 | 0.029979 | PTGS2/TP53 | 2 |
| BP | GO:0019363 | pyridine nucleotide biosynthetic process | 2/37 | 0.034261 | 0.068442 | 0.029979 | PTGS2/TP53 | 2 |
| BP | GO:0061351 | neural precursor cell proliferation | 2/37 | 0.034261 | 0.068442 | 0.029979 | SLC6A4/TP53 | 2 |
| BP | GO:0001654 | eye development | 3/37 | 0.034554 | 0.068878 | 0.03017 | JUN/BCL2/BAX | 3 |
| BP | GO:0009141 | nucleoside triphosphate metabolic process | 3/37 | 0.034554 | 0.068878 | 0.03017 | TP53/CDK1/CCNB1 | 3 |
| BP | GO:0014065 | phosphatidylinositol 3-kinase signaling | 2/37 | 0.034687 | 0.068878 | 0.03017 | KDR/PPARD | 2 |
| BP | GO:0051592 | response to calcium ion | 2/37 | 0.034687 | 0.068878 | 0.03017 | JUN/DPEP1 | 2 |
| BP | GO:0018108 | peptidyl-tyrosine phosphorylation | 3/37 | 0.034795 | 0.068878 | 0.03017 | KDR/TP53/PRKCD | 3 |
| BP | GO:0045861 | negative regulation of proteolysis | 3/37 | 0.034795 | 0.068878 | 0.03017 | PTGS2/TP53/DPEP1 | 3 |
| BP | GO:0002902 | regulation of B cell apoptotic process | 1/37 | 0.035093 | 0.068878 | 0.03017 | BAX | 1 |
| BP | GO:0021756 | striatum development | 1/37 | 0.035093 | 0.068878 | 0.03017 | DRD1 | 1 |
| BP | GO:0031065 | positive regulation of histone deacetylation | 1/37 | 0.035093 | 0.068878 | 0.03017 | TP53 | 1 |
| BP | GO:0035994 | response to muscle stretch | 1/37 | 0.035093 | 0.068878 | 0.03017 | JUN | 1 |
| BP | GO:0045780 | positive regulation of bone resorption | 1/37 | 0.035093 | 0.068878 | 0.03017 | PRKCA | 1 |
| BP | GO:0046852 | positive regulation of bone remodeling | 1/37 | 0.035093 | 0.068878 | 0.03017 | PRKCA | 1 |
| BP | GO:0051782 | negative regulation of cell division | 1/37 | 0.035093 | 0.068878 | 0.03017 | MYC | 1 |
| BP | GO:0071318 | cellular response to ATP | 1/37 | 0.035093 | 0.068878 | 0.03017 | PTGS2 | 1 |
| BP | GO:0071605 | monocyte chemotactic protein-1 production | 1/37 | 0.035093 | 0.068878 | 0.03017 | IL1B | 1 |
| BP | GO:0071637 | regulation of monocyte chemotactic protein-1 production | 1/37 | 0.035093 | 0.068878 | 0.03017 | IL1B | 1 |
| BP | GO:0150078 | positive regulation of neuroinflammatory response | 1/37 | 0.035093 | 0.068878 | 0.03017 | IL1B | 1 |
| BP | GO:1902165 | regulation of intrinsic apoptotic signaling pathway in response to DNA damage by p53 class mediator | 1/37 | 0.035093 | 0.068878 | 0.03017 | BCL2 | 1 |
| BP | GO:2000193 | positive regulation of fatty acid transport | 1/37 | 0.035093 | 0.068878 | 0.03017 | IL1B | 1 |
| BP | GO:2000479 | regulation of cAMP-dependent protein kinase activity | 1/37 | 0.035093 | 0.068878 | 0.03017 | ADRB2 | 1 |
| BP | GO:2000647 | negative regulation of stem cell proliferation | 1/37 | 0.035093 | 0.068878 | 0.03017 | TP53 | 1 |
| BP | GO:0008277 | regulation of G protein-coupled receptor signaling pathway | 2/37 | 0.035116 | 0.068878 | 0.03017 | ADRB2/PRKCA | 2 |
| BP | GO:0048592 | eye morphogenesis | 2/37 | 0.035116 | 0.068878 | 0.03017 | BCL2/BAX | 2 |
| BP | GO:0051092 | positive regulation of NF-kappaB transcription factor activity | 2/37 | 0.035116 | 0.068878 | 0.03017 | AR/IL1B | 2 |
| BP | GO:0018212 | peptidyl-tyrosine modification | 3/37 | 0.035524 | 0.06946 | 0.030425 | KDR/TP53/PRKCD | 3 |
| BP | GO:0150063 | visual system development | 3/37 | 0.035524 | 0.06946 | 0.030425 | JUN/BCL2/BAX | 3 |
| BP | GO:0007338 | single fertilization | 2/37 | 0.035547 | 0.06946 | 0.030425 | AR/CDK1 | 2 |
| BP | GO:0016331 | morphogenesis of embryonic epithelium | 2/37 | 0.035547 | 0.06946 | 0.030425 | AR/CASP3 | 2 |
| BP | GO:0072525 | pyridine-containing compound biosynthetic process | 2/37 | 0.035547 | 0.06946 | 0.030425 | PTGS2/TP53 | 2 |
| BP | GO:0016999 | antibiotic metabolic process | 2/37 | 0.03598 | 0.0702 | 0.030749 | AKR1B1/DPEP1 | 2 |
| BP | GO:1903169 | regulation of calcium ion transmembrane transport | 2/37 | 0.03598 | 0.0702 | 0.030749 | DRD1/BAX | 2 |
| BP | GO:0030198 | extracellular matrix organization | 3/37 | 0.036015 | 0.070216 | 0.030756 | DPP4/PRSS1/KDR | 3 |
| BP | GO:0006909 | phagocytosis | 3/37 | 0.036262 | 0.070404 | 0.030838 | IL1B/PRKCD/PPARG | 3 |
| BP | GO:0000187 | activation of MAPK activity | 2/37 | 0.036415 | 0.070404 | 0.030838 | CDK1/IL1B | 2 |
| BP | GO:0048489 | synaptic vesicle transport | 2/37 | 0.036415 | 0.070404 | 0.030838 | DRD1/CHRM2 | 2 |
| BP | GO:0097480 | establishment of synaptic vesicle localization | 2/37 | 0.036415 | 0.070404 | 0.030838 | DRD1/CHRM2 | 2 |
| BP | GO:0048880 | sensory system development | 3/37 | 0.036758 | 0.070404 | 0.030838 | JUN/BCL2/BAX | 3 |
| BP | GO:0030168 | platelet activation | 2/37 | 0.036852 | 0.070404 | 0.030838 | PRKCA/PRKCD | 2 |
| BP | GO:0034250 | positive regulation of cellular amide metabolic process | 2/37 | 0.036852 | 0.070404 | 0.030838 | CASP3/PRKCD | 2 |
| BP | GO:0045807 | positive regulation of endocytosis | 2/37 | 0.036852 | 0.070404 | 0.030838 | IL1B/PPARG | 2 |
| BP | GO:0050729 | positive regulation of inflammatory response | 2/37 | 0.036852 | 0.070404 | 0.030838 | PTGS2/IL1B | 2 |
| BP | GO:0002363 | alpha-beta T cell lineage commitment | 1/37 | 0.037007 | 0.070404 | 0.030838 | BCL2 | 1 |
| BP | GO:0002726 | positive regulation of T cell cytokine production | 1/37 | 0.037007 | 0.070404 | 0.030838 | IL1B | 1 |
| BP | GO:0002922 | positive regulation of humoral immune response | 1/37 | 0.037007 | 0.070404 | 0.030838 | IL1B | 1 |
| BP | GO:0003085 | negative regulation of systemic arterial blood pressure | 1/37 | 0.037007 | 0.070404 | 0.030838 | ADRB2 | 1 |
| BP | GO:0007213 | G protein-coupled acetylcholine receptor signaling pathway | 1/37 | 0.037007 | 0.070404 | 0.030838 | CHRM2 | 1 |
| BP | GO:0007625 | grooming behavior | 1/37 | 0.037007 | 0.070404 | 0.030838 | DRD1 | 1 |
| BP | GO:0010523 | negative regulation of calcium ion transport into cytosol | 1/37 | 0.037007 | 0.070404 | 0.030838 | BCL2 | 1 |
| BP | GO:0010544 | negative regulation of platelet activation | 1/37 | 0.037007 | 0.070404 | 0.030838 | PRKCD | 1 |
| BP | GO:0010663 | positive regulation of striated muscle cell apoptotic process | 1/37 | 0.037007 | 0.070404 | 0.030838 | TP53 | 1 |
| BP | GO:0010666 | positive regulation of cardiac muscle cell apoptotic process | 1/37 | 0.037007 | 0.070404 | 0.030838 | TP53 | 1 |
| BP | GO:0015669 | gas transport | 1/37 | 0.037007 | 0.070404 | 0.030838 | MYC | 1 |
| BP | GO:0030949 | positive regulation of vascular endothelial growth factor receptor signaling pathway | 1/37 | 0.037007 | 0.070404 | 0.030838 | IL1B | 1 |
| BP | GO:0032303 | regulation of icosanoid secretion | 1/37 | 0.037007 | 0.070404 | 0.030838 | IL1B | 1 |
| BP | GO:0032930 | positive regulation of superoxide anion generation | 1/37 | 0.037007 | 0.070404 | 0.030838 | PRKCD | 1 |
| BP | GO:0034138 | toll-like receptor 3 signaling pathway | 1/37 | 0.037007 | 0.070404 | 0.030838 | CASP8 | 1 |
| BP | GO:0045076 | regulation of interleukin-2 biosynthetic process | 1/37 | 0.037007 | 0.070404 | 0.030838 | IL1B | 1 |
| BP | GO:0051546 | keratinocyte migration | 1/37 | 0.037007 | 0.070404 | 0.030838 | PPARD | 1 |
| BP | GO:0060252 | positive regulation of glial cell proliferation | 1/37 | 0.037007 | 0.070404 | 0.030838 | IL1B | 1 |
| BP | GO:0071731 | response to nitric oxide | 1/37 | 0.037007 | 0.070404 | 0.030838 | DPEP1 | 1 |
| BP | GO:0072074 | kidney mesenchyme development | 1/37 | 0.037007 | 0.070404 | 0.030838 | MYC | 1 |
| BP | GO:1902170 | cellular response to reactive nitrogen species | 1/37 | 0.037007 | 0.070404 | 0.030838 | DPEP1 | 1 |
| BP | GO:1902644 | tertiary alcohol metabolic process | 1/37 | 0.037007 | 0.070404 | 0.030838 | AKR1B1 | 1 |
| BP | GO:1905288 | vascular associated smooth muscle cell apoptotic process | 1/37 | 0.037007 | 0.070404 | 0.030838 | PPARG | 1 |
| BP | GO:1905459 | regulation of vascular associated smooth muscle cell apoptotic process | 1/37 | 0.037007 | 0.070404 | 0.030838 | PPARG | 1 |
| BP | GO:0017156 | calcium ion regulated exocytosis | 2/37 | 0.037291 | 0.070891 | 0.031052 | DRD1/CHRM2 | 2 |
| BP | GO:0009123 | nucleoside monophosphate metabolic process | 3/37 | 0.03776 | 0.07173 | 0.031419 | TP53/CDK1/CCNB1 | 3 |
| BP | GO:0042129 | regulation of T cell proliferation | 2/37 | 0.038175 | 0.07234 | 0.031686 | CASP3/IL1B | 2 |
| BP | GO:0051053 | negative regulation of DNA metabolic process | 2/37 | 0.038175 | 0.07234 | 0.031686 | TP53/PPARG | 2 |
| BP | GO:0001764 | neuron migration | 2/37 | 0.038621 | 0.07234 | 0.031686 | DRD1/BAX | 2 |
| BP | GO:0043271 | negative regulation of ion transport | 2/37 | 0.038621 | 0.07234 | 0.031686 | PTGS2/BCL2 | 2 |
| BP | GO:0002320 | lymphoid progenitor cell differentiation | 1/37 | 0.038918 | 0.07234 | 0.031686 | BCL2 | 1 |
| BP | GO:0002827 | positive regulation of T-helper 1 type immune response | 1/37 | 0.038918 | 0.07234 | 0.031686 | IL1B | 1 |
| BP | GO:0006271 | DNA strand elongation involved in DNA replication | 1/37 | 0.038918 | 0.07234 | 0.031686 | PCNA | 1 |
| BP | GO:0006525 | arginine metabolic process | 1/37 | 0.038918 | 0.07234 | 0.031686 | NOS2 | 1 |
| BP | GO:0006677 | glycosylceramide metabolic process | 1/37 | 0.038918 | 0.07234 | 0.031686 | PRKCD | 1 |
| BP | GO:0007095 | mitotic G2 DNA damage checkpoint | 1/37 | 0.038918 | 0.07234 | 0.031686 | CDK1 | 1 |
| BP | GO:0007620 | copulation | 1/37 | 0.038918 | 0.07234 | 0.031686 | SLC6A4 | 1 |
| BP | GO:0010042 | response to manganese ion | 1/37 | 0.038918 | 0.07234 | 0.031686 | PTGS2 | 1 |
| BP | GO:0010738 | regulation of protein kinase A signaling | 1/37 | 0.038918 | 0.07234 | 0.031686 | ADRB2 | 1 |
| BP | GO:0016137 | glycoside metabolic process | 1/37 | 0.038918 | 0.07234 | 0.031686 | AKR1B1 | 1 |
| BP | GO:0032042 | mitochondrial DNA metabolic process | 1/37 | 0.038918 | 0.07234 | 0.031686 | TP53 | 1 |
| BP | GO:0033194 | response to hydroperoxide | 1/37 | 0.038918 | 0.07234 | 0.031686 | PRKCD | 1 |
| BP | GO:0034393 | positive regulation of smooth muscle cell apoptotic process | 1/37 | 0.038918 | 0.07234 | 0.031686 | PPARG | 1 |
| BP | GO:0042053 | regulation of dopamine metabolic process | 1/37 | 0.038918 | 0.07234 | 0.031686 | DRD1 | 1 |
| BP | GO:0042069 | regulation of catecholamine metabolic process | 1/37 | 0.038918 | 0.07234 | 0.031686 | DRD1 | 1 |
| BP | GO:0042276 | error-prone translesion synthesis | 1/37 | 0.038918 | 0.07234 | 0.031686 | PCNA | 1 |
| BP | GO:0043369 | CD4-positive or CD8-positive, alpha-beta T cell lineage commitment | 1/37 | 0.038918 | 0.07234 | 0.031686 | BCL2 | 1 |
| BP | GO:0046514 | ceramide catabolic process | 1/37 | 0.038918 | 0.07234 | 0.031686 | PRKCD | 1 |
| BP | GO:0048148 | behavioral response to cocaine | 1/37 | 0.038918 | 0.07234 | 0.031686 | DRD1 | 1 |
| BP | GO:0051580 | regulation of neurotransmitter uptake | 1/37 | 0.038918 | 0.07234 | 0.031686 | DRD1 | 1 |
| BP | GO:0051767 | nitric-oxide synthase biosynthetic process | 1/37 | 0.038918 | 0.07234 | 0.031686 | KDR | 1 |
| BP | GO:0051769 | regulation of nitric-oxide synthase biosynthetic process | 1/37 | 0.038918 | 0.07234 | 0.031686 | KDR | 1 |
| BP | GO:0060602 | branch elongation of an epithelium | 1/37 | 0.038918 | 0.07234 | 0.031686 | ESR1 | 1 |
| BP | GO:0070987 | error-free translesion synthesis | 1/37 | 0.038918 | 0.07234 | 0.031686 | PCNA | 1 |
| BP | GO:0071636 | positive regulation of transforming growth factor beta production | 1/37 | 0.038918 | 0.07234 | 0.031686 | PTGS2 | 1 |
| BP | GO:1902993 | positive regulation of amyloid precursor protein catabolic process | 1/37 | 0.038918 | 0.07234 | 0.031686 | CASP3 | 1 |
| BP | GO:0030900 | forebrain development | 3/37 | 0.03929 | 0.072927 | 0.031944 | DRD1/BAX/CASP3 | 3 |
| BP | GO:0045089 | positive regulation of innate immune response | 3/37 | 0.03929 | 0.072927 | 0.031944 | ESR1/CASP8/PRKCD | 3 |
| BP | GO:0006665 | sphingolipid metabolic process | 2/37 | 0.039517 | 0.073296 | 0.032105 | BAX/PRKCD | 2 |
| BP | GO:1903305 | regulation of regulated secretory pathway | 2/37 | 0.039968 | 0.074079 | 0.032448 | DRD1/CHRM2 | 2 |
| BP | GO:0032635 | interleukin-6 production | 2/37 | 0.040421 | 0.074758 | 0.032746 | NOS2/IL1B | 2 |
| BP | GO:0002029 | desensitization of G protein-coupled receptor signaling pathway | 1/37 | 0.040824 | 0.074758 | 0.032746 | ADRB2 | 1 |
| BP | GO:0010829 | negative regulation of glucose transmembrane transport | 1/37 | 0.040824 | 0.074758 | 0.032746 | IL1B | 1 |
| BP | GO:0010893 | positive regulation of steroid biosynthetic process | 1/37 | 0.040824 | 0.074758 | 0.032746 | IL1B | 1 |
| BP | GO:0014821 | phasic smooth muscle contraction | 1/37 | 0.040824 | 0.074758 | 0.032746 | DRD1 | 1 |
| BP | GO:0022401 | negative adaptation of signaling pathway | 1/37 | 0.040824 | 0.074758 | 0.032746 | ADRB2 | 1 |
| BP | GO:0032986 | protein-DNA complex disassembly | 1/37 | 0.040824 | 0.074758 | 0.032746 | MYC | 1 |
| BP | GO:0035162 | embryonic hemopoiesis | 1/37 | 0.040824 | 0.074758 | 0.032746 | KDR | 1 |
| BP | GO:0035357 | peroxisome proliferator activated receptor signaling pathway | 1/37 | 0.040824 | 0.074758 | 0.032746 | PPARG | 1 |
| BP | GO:0035743 | CD4-positive, alpha-beta T cell cytokine production | 1/37 | 0.040824 | 0.074758 | 0.032746 | IL1B | 1 |
| BP | GO:0043153 | entrainment of circadian clock by photoperiod | 1/37 | 0.040824 | 0.074758 | 0.032746 | TP53 | 1 |
| BP | GO:0051412 | response to corticosterone | 1/37 | 0.040824 | 0.074758 | 0.032746 | CDKN1A | 1 |
| BP | GO:0070584 | mitochondrion morphogenesis | 1/37 | 0.040824 | 0.074758 | 0.032746 | BAX | 1 |
| BP | GO:0071459 | protein localization to chromosome, centromeric region | 1/37 | 0.040824 | 0.074758 | 0.032746 | CDK1 | 1 |
| BP | GO:0071498 | cellular response to fluid shear stress | 1/37 | 0.040824 | 0.074758 | 0.032746 | PTGS2 | 1 |
| BP | GO:0072111 | cell proliferation involved in kidney development | 1/37 | 0.040824 | 0.074758 | 0.032746 | MYC | 1 |
| BP | GO:2000269 | regulation of fibroblast apoptotic process | 1/37 | 0.040824 | 0.074758 | 0.032746 | TP53 | 1 |
| BP | GO:0000723 | telomere maintenance | 2/37 | 0.040876 | 0.0748 | 0.032764 | PCNA/MYC | 2 |
| BP | GO:0031214 | biomineral tissue development | 2/37 | 0.041333 | 0.07553 | 0.033084 | PTGS2/ADRB2 | 2 |
| BP | GO:0097479 | synaptic vesicle localization | 2/37 | 0.041333 | 0.07553 | 0.033084 | DRD1/CHRM2 | 2 |
| BP | GO:0014706 | striated muscle tissue development | 3/37 | 0.041644 | 0.076044 | 0.033309 | BCL2/CDK1/CCNB1 | 3 |
| BP | GO:0007088 | regulation of mitotic nuclear division | 2/37 | 0.041792 | 0.076261 | 0.033404 | IL1B/CCNB1 | 2 |
| BP | GO:0002244 | hematopoietic progenitor cell differentiation | 2/37 | 0.042716 | 0.076726 | 0.033608 | BCL2/TP53 | 2 |
| BP | GO:0000423 | mitophagy | 1/37 | 0.042728 | 0.076726 | 0.033608 | TP53 | 1 |
| BP | GO:0001759 | organ induction | 1/37 | 0.042728 | 0.076726 | 0.033608 | AR | 1 |
| BP | GO:0006582 | melanin metabolic process | 1/37 | 0.042728 | 0.076726 | 0.033608 | BCL2 | 1 |
| BP | GO:0008053 | mitochondrial fusion | 1/37 | 0.042728 | 0.076726 | 0.033608 | BAX | 1 |
| BP | GO:0009235 | cobalamin metabolic process | 1/37 | 0.042728 | 0.076726 | 0.033608 | PRSS1 | 1 |
| BP | GO:0010226 | response to lithium ion | 1/37 | 0.042728 | 0.076726 | 0.033608 | PTGS2 | 1 |
| BP | GO:0010869 | regulation of receptor biosynthetic process | 1/37 | 0.042728 | 0.076726 | 0.033608 | PPARG | 1 |
| BP | GO:0021854 | hypothalamus development | 1/37 | 0.042728 | 0.076726 | 0.033608 | BAX | 1 |
| BP | GO:0023058 | adaptation of signaling pathway | 1/37 | 0.042728 | 0.076726 | 0.033608 | ADRB2 | 1 |
| BP | GO:0031281 | positive regulation of cyclase activity | 1/37 | 0.042728 | 0.076726 | 0.033608 | NOS2 | 1 |
| BP | GO:0032928 | regulation of superoxide anion generation | 1/37 | 0.042728 | 0.076726 | 0.033608 | PRKCD | 1 |
| BP | GO:0035584 | calcium-mediated signaling using intracellular calcium source | 1/37 | 0.042728 | 0.076726 | 0.033608 | KDR | 1 |
| BP | GO:0035809 | regulation of urine volume | 1/37 | 0.042728 | 0.076726 | 0.033608 | AKR1B1 | 1 |
| BP | GO:0042094 | interleukin-2 biosynthetic process | 1/37 | 0.042728 | 0.076726 | 0.033608 | IL1B | 1 |
| BP | GO:0042359 | vitamin D metabolic process | 1/37 | 0.042728 | 0.076726 | 0.033608 | IL1B | 1 |
| BP | GO:0051195 | negative regulation of cofactor metabolic process | 1/37 | 0.042728 | 0.076726 | 0.033608 | TP53 | 1 |
| BP | GO:0060065 | uterus development | 1/37 | 0.042728 | 0.076726 | 0.033608 | ESR1 | 1 |
| BP | GO:0060575 | intestinal epithelial cell differentiation | 1/37 | 0.042728 | 0.076726 | 0.033608 | CDKN1A | 1 |
| BP | GO:0071379 | cellular response to prostaglandin stimulus | 1/37 | 0.042728 | 0.076726 | 0.033608 | PPARG | 1 |
| BP | GO:0071404 | cellular response to low-density lipoprotein particle stimulus | 1/37 | 0.042728 | 0.076726 | 0.033608 | PPARG | 1 |
| BP | GO:0090312 | positive regulation of protein deacetylation | 1/37 | 0.042728 | 0.076726 | 0.033608 | TP53 | 1 |
| BP | GO:1901522 | positive regulation of transcription from RNA polymerase II promoter involved in cellular response to chemical stimulus | 1/37 | 0.042728 | 0.076726 | 0.033608 | TP53 | 1 |
| BP | GO:0010639 | negative regulation of organelle organization | 3/37 | 0.042982 | 0.07713 | 0.033785 | TP53/PRKCD/CCNB1 | 3 |
| BP | GO:0035051 | cardiocyte differentiation | 2/37 | 0.043181 | 0.077433 | 0.033917 | CDK1/CCNB1 | 2 |
| BP | GO:0051302 | regulation of cell division | 2/37 | 0.043648 | 0.078216 | 0.03426 | MYC/IL1B | 2 |
| BP | GO:1903532 | positive regulation of secretion by cell | 3/37 | 0.044069 | 0.078453 | 0.034364 | PPARD/SLC6A4/IL1B | 3 |
| BP | GO:0007269 | neurotransmitter secretion | 2/37 | 0.044116 | 0.078453 | 0.034364 | DRD1/CHRM2 | 2 |
| BP | GO:0099643 | signal release from synapse | 2/37 | 0.044587 | 0.078453 | 0.034364 | DRD1/CHRM2 | 2 |
| BP | GO:0006297 | nucleotide-excision repair, DNA gap filling | 1/37 | 0.044627 | 0.078453 | 0.034364 | PCNA | 1 |
| BP | GO:0006359 | regulation of transcription by RNA polymerase III | 1/37 | 0.044627 | 0.078453 | 0.034364 | AR | 1 |
| BP | GO:0007413 | axonal fasciculation | 1/37 | 0.044627 | 0.078453 | 0.034364 | CASP3 | 1 |
| BP | GO:0007530 | sex determination | 1/37 | 0.044627 | 0.078453 | 0.034364 | AR | 1 |
| BP | GO:0007617 | mating behavior | 1/37 | 0.044627 | 0.078453 | 0.034364 | DRD1 | 1 |
| BP | GO:0019400 | alditol metabolic process | 1/37 | 0.044627 | 0.078453 | 0.034364 | AKR1B1 | 1 |
| BP | GO:0030539 | male genitalia development | 1/37 | 0.044627 | 0.078453 | 0.034364 | AR | 1 |
| BP | GO:0032373 | positive regulation of sterol transport | 1/37 | 0.044627 | 0.078453 | 0.034364 | PON1 | 1 |
| BP | GO:0032376 | positive regulation of cholesterol transport | 1/37 | 0.044627 | 0.078453 | 0.034364 | PON1 | 1 |
| BP | GO:0032799 | low-density lipoprotein receptor particle metabolic process | 1/37 | 0.044627 | 0.078453 | 0.034364 | PPARG | 1 |
| BP | GO:0034695 | response to prostaglandin E | 1/37 | 0.044627 | 0.078453 | 0.034364 | PPARG | 1 |
| BP | GO:0036037 | CD8-positive, alpha-beta T cell activation | 1/37 | 0.044627 | 0.078453 | 0.034364 | BCL2 | 1 |
| BP | GO:0043950 | positive regulation of cAMP-mediated signaling | 1/37 | 0.044627 | 0.078453 | 0.034364 | PRKCA | 1 |
| BP | GO:0048714 | positive regulation of oligodendrocyte differentiation | 1/37 | 0.044627 | 0.078453 | 0.034364 | PPARG | 1 |
| BP | GO:0050995 | negative regulation of lipid catabolic process | 1/37 | 0.044627 | 0.078453 | 0.034364 | IL1B | 1 |
| BP | GO:0051043 | regulation of membrane protein ectodomain proteolysis | 1/37 | 0.044627 | 0.078453 | 0.034364 | IL1B | 1 |
| BP | GO:0051349 | positive regulation of lyase activity | 1/37 | 0.044627 | 0.078453 | 0.034364 | NOS2 | 1 |
| BP | GO:0072215 | regulation of metanephros development | 1/37 | 0.044627 | 0.078453 | 0.034364 | MYC | 1 |
| BP | GO:0090140 | regulation of mitochondrial fission | 1/37 | 0.044627 | 0.078453 | 0.034364 | KDR | 1 |
| BP | GO:0090343 | positive regulation of cell aging | 1/37 | 0.044627 | 0.078453 | 0.034364 | TP53 | 1 |
| BP | GO:0106030 | neuron projection fasciculation | 1/37 | 0.044627 | 0.078453 | 0.034364 | CASP3 | 1 |
| BP | GO:1902254 | negative regulation of intrinsic apoptotic signaling pathway by p53 class mediator | 1/37 | 0.044627 | 0.078453 | 0.034364 | BCL2 | 1 |
| BP | GO:1903429 | regulation of cell maturation | 1/37 | 0.044627 | 0.078453 | 0.034364 | BCL2 | 1 |
| BP | GO:2000291 | regulation of myoblast proliferation | 1/37 | 0.044627 | 0.078453 | 0.034364 | PPARD | 1 |
| BP | GO:2000637 | positive regulation of gene silencing by miRNA | 1/37 | 0.044627 | 0.078453 | 0.034364 | TP53 | 1 |
| BP | GO:0006732 | coenzyme metabolic process | 3/37 | 0.045169 | 0.079351 | 0.034758 | PTGS2/TP53/FASN | 3 |
| BP | GO:0001783 | B cell apoptotic process | 1/37 | 0.046523 | 0.081125 | 0.035535 | BAX | 1 |
| BP | GO:0002227 | innate immune response in mucosa | 1/37 | 0.046523 | 0.081125 | 0.035535 | NOS2 | 1 |
| BP | GO:0009110 | vitamin biosynthetic process | 1/37 | 0.046523 | 0.081125 | 0.035535 | IL1B | 1 |
| BP | GO:0010288 | response to lead ion | 1/37 | 0.046523 | 0.081125 | 0.035535 | PTGS2 | 1 |
| BP | GO:0010640 | regulation of platelet-derived growth factor receptor signaling pathway | 1/37 | 0.046523 | 0.081125 | 0.035535 | F7 | 1 |
| BP | GO:0021544 | subpallium development | 1/37 | 0.046523 | 0.081125 | 0.035535 | DRD1 | 1 |
| BP | GO:0021895 | cerebral cortex neuron differentiation | 1/37 | 0.046523 | 0.081125 | 0.035535 | DRD1 | 1 |
| BP | GO:0032897 | negative regulation of viral transcription | 1/37 | 0.046523 | 0.081125 | 0.035535 | JUN | 1 |
| BP | GO:0060148 | positive regulation of posttranscriptional gene silencing | 1/37 | 0.046523 | 0.081125 | 0.035535 | TP53 | 1 |
| BP | GO:2000209 | regulation of anoikis | 1/37 | 0.046523 | 0.081125 | 0.035535 | BCL2 | 1 |
| BP | GO:2001026 | regulation of endothelial cell chemotaxis | 1/37 | 0.046523 | 0.081125 | 0.035535 | KDR | 1 |
| BP | GO:0060537 | muscle tissue development | 3/37 | 0.046563 | 0.081142 | 0.035542 | BCL2/CDK1/CCNB1 | 3 |
| BP | GO:0032200 | telomere organization | 2/37 | 0.046968 | 0.081791 | 0.035826 | PCNA/MYC | 2 |
| BP | GO:0007517 | muscle organ development | 3/37 | 0.047127 | 0.082014 | 0.035924 | BCL2/CDK1/CCNB1 | 3 |
| BP | GO:0009408 | response to heat | 2/37 | 0.047449 | 0.082464 | 0.036121 | PTGS2/CDKN1A | 2 |
| BP | GO:0051897 | positive regulation of protein kinase B signaling | 2/37 | 0.047449 | 0.082464 | 0.036121 | F7/ESR1 | 2 |
| BP | GO:0001959 | regulation of cytokine-mediated signaling pathway | 2/37 | 0.047933 | 0.083193 | 0.03644 | CASP8/PPARG | 2 |
| BP | GO:0043488 | regulation of mRNA stability | 2/37 | 0.047933 | 0.083193 | 0.03644 | PRKCA/PRKCD | 2 |
| BP | GO:0002053 | positive regulation of mesenchymal cell proliferation | 1/37 | 0.048415 | 0.083249 | 0.036465 | MYC | 1 |
| BP | GO:0009648 | photoperiodism | 1/37 | 0.048415 | 0.083249 | 0.036465 | TP53 | 1 |
| BP | GO:0019054 | modulation by virus of host process | 1/37 | 0.048415 | 0.083249 | 0.036465 | CASP8 | 1 |
| BP | GO:0031069 | hair follicle morphogenesis | 1/37 | 0.048415 | 0.083249 | 0.036465 | BCL2 | 1 |
| BP | GO:0031664 | regulation of lipopolysaccharide-mediated signaling pathway | 1/37 | 0.048415 | 0.083249 | 0.036465 | PRKCA | 1 |
| BP | GO:0045662 | negative regulation of myoblast differentiation | 1/37 | 0.048415 | 0.083249 | 0.036465 | PPARD | 1 |
| BP | GO:0051894 | positive regulation of focal adhesion assembly | 1/37 | 0.048415 | 0.083249 | 0.036465 | KDR | 1 |
| BP | GO:0060330 | regulation of response to interferon-gamma | 1/37 | 0.048415 | 0.083249 | 0.036465 | PPARG | 1 |
| BP | GO:0060334 | regulation of interferon-gamma-mediated signaling pathway | 1/37 | 0.048415 | 0.083249 | 0.036465 | PPARG | 1 |
| BP | GO:0060561 | apoptotic process involved in morphogenesis | 1/37 | 0.048415 | 0.083249 | 0.036465 | BAX | 1 |
| BP | GO:0060571 | morphogenesis of an epithelial fold | 1/37 | 0.048415 | 0.083249 | 0.036465 | AR | 1 |
| BP | GO:1900101 | regulation of endoplasmic reticulum unfolded protein response | 1/37 | 0.048415 | 0.083249 | 0.036465 | BAX | 1 |
| BP | GO:1904385 | cellular response to angiotensin | 1/37 | 0.048415 | 0.083249 | 0.036465 | PRKCD | 1 |
| BP | GO:2000311 | regulation of AMPA receptor activity | 1/37 | 0.048415 | 0.083249 | 0.036465 | ADRB2 | 1 |
| BP | GO:0006611 | protein export from nucleus | 2/37 | 0.048906 | 0.083981 | 0.036786 | TP53/IL1B | 2 |
| BP | GO:0051099 | positive regulation of binding | 2/37 | 0.048906 | 0.083981 | 0.036786 | PON1/PPARG | 2 |
| BP | GO:1901796 | regulation of signal transduction by p53 class mediator | 2/37 | 0.049395 | 0.084709 | 0.037104 | BCL2/TP53 | 2 |
| BP | GO:1905330 | regulation of morphogenesis of an epithelium | 2/37 | 0.049395 | 0.084709 | 0.037104 | AR/ESR1 | 2 |
| BP | GO:0022408 | negative regulation of cell-cell adhesion | 2/37 | 0.049885 | 0.085419 | 0.037416 | CASP3/PRKCD | 2 |
| BP | GO:0048015 | phosphatidylinositol-mediated signaling | 2/37 | 0.049885 | 0.085419 | 0.037416 | KDR/PPARD | 2 |
| CC | GO:0000307 | cyclin-dependent protein kinase holoenzyme complex | 4/37 | 1.12E-06 | 0.000165 | 0.000108 | CDKN1A/CDK1/PCNA/CCNB1 | 4 |
| CC | GO:1902554 | serine/threonine protein kinase complex | 4/37 | 2.19E-05 | 0.000902 | 0.00059 | CDKN1A/CDK1/PCNA/CCNB1 | 4 |
| CC | GO:0045121 | membrane raft | 6/37 | 2.43E-05 | 0.000902 | 0.00059 | PTGS2/DPP4/KDR/SLC6A4/CASP3/CASP8 | 6 |
| CC | GO:0098857 | membrane microdomain | 6/37 | 2.47E-05 | 0.000902 | 0.00059 | PTGS2/DPP4/KDR/SLC6A4/CASP3/CASP8 | 6 |
| CC | GO:0098589 | membrane region | 6/37 | 3.05E-05 | 0.000902 | 0.00059 | PTGS2/DPP4/KDR/SLC6A4/CASP3/CASP8 | 6 |
| CC | GO:1902911 | protein kinase complex | 4/37 | 5.07E-05 | 0.001251 | 0.000818 | CDKN1A/CDK1/PCNA/CCNB1 | 4 |
| CC | GO:0000790 | nuclear chromatin | 6/37 | 6.61E-05 | 0.001398 | 0.000915 | AR/PPARD/JUN/ESR1/TP53/MYC | 6 |
| CC | GO:0061695 | transferase complex, transferring phosphorus-containing groups | 5/37 | 0.000116 | 0.002152 | 0.001408 | CDKN1A/TP53/CDK1/PCNA/CCNB1 | 5 |
| CC | GO:0090575 | RNA polymerase II transcription factor complex | 4/37 | 0.00024 | 0.003952 | 0.002586 | PPARD/JUN/TP53/PPARG | 4 |
| CC | GO:0005741 | mitochondrial outer membrane | 4/37 | 0.000336 | 0.00465 | 0.003043 | PGR/BCL2/BAX/CASP8 | 4 |
| CC | GO:0099056 | integral component of presynaptic membrane | 3/37 | 0.000346 | 0.00465 | 0.003043 | DRD1/CHRM2/SLC6A4 | 3 |
| CC | GO:0098889 | intrinsic component of presynaptic membrane | 3/37 | 0.000486 | 0.005448 | 0.003565 | DRD1/CHRM2/SLC6A4 | 3 |
| CC | GO:0031968 | organelle outer membrane | 4/37 | 0.000532 | 0.005448 | 0.003565 | PGR/BCL2/BAX/CASP8 | 4 |
| CC | GO:0044798 | nuclear transcription factor complex | 4/37 | 0.000532 | 0.005448 | 0.003565 | PPARD/JUN/TP53/PPARG | 4 |
| CC | GO:0019867 | outer membrane | 4/37 | 0.000552 | 0.005448 | 0.003565 | PGR/BCL2/BAX/CASP8 | 4 |
| CC | GO:0046930 | pore complex | 2/37 | 0.000846 | 0.007822 | 0.005118 | BCL2/BAX | 2 |
| CC | GO:0099055 | integral component of postsynaptic membrane | 3/37 | 0.001366 | 0.011891 | 0.007781 | DRD1/CHRM2/SLC6A4 | 3 |
| CC | GO:0098936 | intrinsic component of postsynaptic membrane | 3/37 | 0.00154 | 0.012664 | 0.008287 | DRD1/CHRM2/SLC6A4 | 3 |
| CC | GO:0034399 | nuclear periphery | 3/37 | 0.001888 | 0.014708 | 0.009624 | TP53/PCNA/PRKCD | 3 |
| CC | GO:0099699 | integral component of synaptic membrane | 3/37 | 0.00288 | 0.021313 | 0.013946 | DRD1/CHRM2/SLC6A4 | 3 |
| CC | GO:0042734 | presynaptic membrane | 3/37 | 0.003387 | 0.023871 | 0.01562 | DRD1/CHRM2/SLC6A4 | 3 |
| CC | GO:0099240 | intrinsic component of synaptic membrane | 3/37 | 0.003567 | 0.023999 | 0.015704 | DRD1/CHRM2/SLC6A4 | 3 |
| CC | GO:0043209 | myelin sheath | 2/37 | 0.003812 | 0.024529 | 0.01605 | AKR1B1/BCL2 | 2 |
| CC | GO:0005667 | transcription factor complex | 4/37 | 0.004711 | 0.029053 | 0.019011 | PPARD/JUN/TP53/PPARG | 4 |
| CC | GO:0005657 | replication fork | 2/37 | 0.007637 | 0.045211 | 0.029583 | TP53/PCNA | 2 |
| CC | GO:0005902 | microvillus | 2/37 | 0.010598 | 0.060328 | 0.039475 | AKR1B1/DPEP1 | 2 |
| MF | GO:0004879 | nuclear receptor activity | 6/37 | 5.51E-10 | 7.05E-08 | 3.36E-08 | AR/ESR2/PPARD/ESR1/PGR/PPARG | 6 |
| MF | GO:0098531 | transcription factor activity, direct ligand regulated sequence-specific DNA binding | 6/37 | 5.51E-10 | 7.05E-08 | 3.36E-08 | AR/ESR2/PPARD/ESR1/PGR/PPARG | 6 |
| MF | GO:0003707 | steroid hormone receptor activity | 6/37 | 1.64E-09 | 1.40E-07 | 6.69E-08 | AR/ESR2/PPARD/ESR1/PGR/PPARG | 6 |
| MF | GO:0001085 | RNA polymerase II transcription factor binding | 6/37 | 7.61E-07 | 4.87E-05 | 2.32E-05 | AR/PPARD/JUN/ESR1/TP53/PPARG | 6 |
| MF | GO:0097153 | cysteine-type endopeptidase activity involved in apoptotic process | 3/37 | 3.76E-06 | 0.000193 | 9.19E-05 | CASP9/CASP3/CASP8 | 3 |
| MF | GO:0035173 | histone kinase activity | 3/37 | 5.61E-06 | 0.000239 | 0.000114 | PRKCA/CDK1/CCNB1 | 3 |
| MF | GO:0001091 | RNA polymerase II basal transcription factor binding | 3/37 | 9.36E-06 | 0.000342 | 0.000163 | AR/ESR1/TP53 | 3 |
| MF | GO:0044389 | ubiquitin-like protein ligase binding | 6/37 | 3.91E-05 | 0.00125 | 0.000596 | JUN/BCL2/CASP8/CDKN1A/TP53/CCNB1 | 6 |
| MF | GO:0005496 | steroid binding | 4/37 | 4.49E-05 | 0.001278 | 0.00061 | AR/ESR2/ESR1/PGR | 4 |
| MF | GO:0030331 | estrogen receptor binding | 3/37 | 9.13E-05 | 0.002337 | 0.001115 | ESR1/PCNA/PPARG | 3 |
| MF | GO:0002020 | protease binding | 4/37 | 0.000143 | 0.003095 | 0.001476 | DPP4/BCL2/CASP3/TP53 | 4 |
| MF | GO:0016538 | cyclin-dependent protein serine/threonine kinase regulator activity | 3/37 | 0.000145 | 0.003095 | 0.001476 | CASP3/CDKN1A/CCNB1 | 3 |
| MF | GO:0051400 | BH domain binding | 2/37 | 0.000189 | 0.003463 | 0.001652 | BCL2/BAX | 2 |
| MF | GO:0070513 | death domain binding | 2/37 | 0.000189 | 0.003463 | 0.001652 | BCL2/BAX | 2 |
| MF | GO:0004175 | endopeptidase activity | 6/37 | 0.000235 | 0.003733 | 0.001781 | F7/DPP4/PRSS1/CASP9/CASP3/CASP8 | 6 |
| MF | GO:0001228 | DNA-binding transcription activator activity, RNA polymerase II-specific | 6/37 | 0.000273 | 0.003733 | 0.001781 | AR/JUN/ESR1/PGR/TP53/MYC | 6 |
| MF | GO:0004861 | cyclin-dependent protein serine/threonine kinase inhibitor activity | 2/37 | 0.000277 | 0.003733 | 0.001781 | CASP3/CDKN1A | 2 |
| MF | GO:0008239 | dipeptidyl-peptidase activity | 2/37 | 0.000277 | 0.003733 | 0.001781 | DPP4/DPEP1 | 2 |
| MF | GO:0035257 | nuclear hormone receptor binding | 4/37 | 0.000277 | 0.003733 | 0.001781 | NCOA2/ESR1/PCNA/PPARG | 4 |
| MF | GO:0031625 | ubiquitin protein ligase binding | 5/37 | 0.000324 | 0.004145 | 0.001977 | JUN/BCL2/CASP8/CDKN1A/TP53 | 5 |
| MF | GO:0030374 | nuclear receptor transcription coactivator activity | 3/37 | 0.000368 | 0.004433 | 0.002115 | NCOA2/PPARD/PPARG | 3 |
| MF | GO:0036041 | long-chain fatty acid binding | 2/37 | 0.000381 | 0.004433 | 0.002115 | PPARD/PPARG | 2 |
| MF | GO:0070491 | repressing transcription factor binding | 3/37 | 0.000436 | 0.004652 | 0.002219 | PPARD/MYC/PPARG | 3 |
| MF | GO:0001098 | basal transcription machinery binding | 3/37 | 0.000454 | 0.004652 | 0.002219 | AR/ESR1/TP53 | 3 |
| MF | GO:0001099 | basal RNA polymerase II transcription machinery binding | 3/37 | 0.000454 | 0.004652 | 0.002219 | AR/ESR1/TP53 | 3 |
| MF | GO:0003713 | transcription coactivator activity | 5/37 | 0.000501 | 0.004751 | 0.002266 | ESR2/NCOA2/PPARD/JUN/PPARG | 5 |
| MF | GO:0004697 | protein kinase C activity | 2/37 | 0.000501 | 0.004751 | 0.002266 | PRKCA/PRKCD | 2 |
| MF | GO:0005123 | death receptor binding | 2/37 | 0.000567 | 0.005147 | 0.002455 | CASP3/CASP8 | 2 |
| MF | GO:0051427 | hormone receptor binding | 4/37 | 0.000583 | 0.005147 | 0.002455 | NCOA2/ESR1/PCNA/PPARG | 4 |
| MF | GO:0051117 | ATPase binding | 3/37 | 0.000619 | 0.00528 | 0.002518 | AR/ESR1/PGR | 3 |
| MF | GO:0033613 | activating transcription factor binding | 3/37 | 0.000738 | 0.006098 | 0.002909 | JUN/MYC/PPARG | 3 |
| MF | GO:1901338 | catecholamine binding | 2/37 | 0.000789 | 0.006313 | 0.003011 | DRD1/ADRB2 | 2 |
| MF | GO:0035258 | steroid hormone receptor binding | 3/37 | 0.00093 | 0.007211 | 0.003439 | ESR1/PCNA/PPARG | 3 |
| MF | GO:0035035 | histone acetyltransferase binding | 2/37 | 0.001667 | 0.012548 | 0.005985 | TP53/PCNA | 2 |
| MF | GO:0030332 | cyclin binding | 2/37 | 0.001783 | 0.012889 | 0.006148 | CDKN1A/CDK1 | 2 |
| MF | GO:0004197 | cysteine-type endopeptidase activity | 3/37 | 0.001813 | 0.012889 | 0.006148 | CASP9/CASP3/CASP8 | 3 |
| MF | GO:0031072 | heat shock protein binding | 3/37 | 0.00195 | 0.013311 | 0.006349 | KDR/BAX/CDK1 | 3 |
| MF | GO:0030291 | protein serine/threonine kinase inhibitor activity | 2/37 | 0.002028 | 0.013311 | 0.006349 | CASP3/CDKN1A | 2 |
| MF | GO:0051721 | protein phosphatase 2A binding | 2/37 | 0.002028 | 0.013311 | 0.006349 | BCL2/TP53 | 2 |
| MF | GO:0005504 | fatty acid binding | 2/37 | 0.002288 | 0.014641 | 0.006983 | PPARD/PPARG | 2 |
| MF | GO:0001103 | RNA polymerase II repressing transcription factor binding | 2/37 | 0.002423 | 0.01513 | 0.007216 | PPARD/PPARG | 2 |
| MF | GO:0005178 | integrin binding | 3/37 | 0.002619 | 0.015961 | 0.007613 | KDR/PRKCA/IL1B | 3 |
| MF | GO:0020037 | heme binding | 3/37 | 0.002791 | 0.016614 | 0.007924 | NOS2/PTGS2/PTGS1 | 3 |
| MF | GO:0019903 | protein phosphatase binding | 3/37 | 0.003093 | 0.017993 | 0.008582 | BCL2/TP53/PPARG | 3 |
| MF | GO:0046906 | tetrapyrrole binding | 3/37 | 0.003414 | 0.019335 | 0.009222 | NOS2/PTGS2/PTGS1 | 3 |
| MF | GO:0030544 | Hsp70 protein binding | 2/37 | 0.003474 | 0.019335 | 0.009222 | BAX/CDK1 | 2 |
| MF | GO:0032813 | tumor necrosis factor receptor superfamily binding | 2/37 | 0.004155 | 0.022629 | 0.010793 | CASP3/CASP8 | 2 |
| MF | GO:0016705 | oxidoreductase activity, acting on paired donors, with incorporation or reduction of molecular oxygen | 3/37 | 0.00442 | 0.023122 | 0.011028 | NOS2/PTGS2/PTGS1 | 3 |
| MF | GO:0004252 | serine-type endopeptidase activity | 3/37 | 0.004498 | 0.023122 | 0.011028 | F7/DPP4/PRSS1 | 3 |
| MF | GO:0004177 | aminopeptidase activity | 2/37 | 0.004516 | 0.023122 | 0.011028 | DPP4/DPEP1 | 2 |
| MF | GO:0070888 | E-box binding | 2/37 | 0.004891 | 0.024553 | 0.011711 | MYC/PPARG | 2 |
| MF | GO:0004601 | peroxidase activity | 2/37 | 0.005281 | 0.025508 | 0.012167 | PTGS2/PTGS1 | 2 |
| MF | GO:0050661 | NADP binding | 2/37 | 0.005281 | 0.025508 | 0.012167 | NOS2/FASN | 2 |
| MF | GO:0001047 | core promoter binding | 2/37 | 0.005891 | 0.027891 | 0.013303 | TP53/MYC | 2 |
| MF | GO:0016684 | oxidoreductase activity, acting on peroxide as acceptor | 2/37 | 0.006101 | 0.027891 | 0.013303 | PTGS2/PTGS1 | 2 |
| MF | GO:0043621 | protein self-association | 2/37 | 0.006101 | 0.027891 | 0.013303 | TP53/PPARG | 2 |
| MF | GO:0019887 | protein kinase regulator activity | 3/37 | 0.006237 | 0.028014 | 0.013362 | CASP3/CDKN1A/CCNB1 | 3 |
| MF | GO:0008236 | serine-type peptidase activity | 3/37 | 0.00643 | 0.02819 | 0.013446 | F7/DPP4/PRSS1 | 3 |
| MF | GO:0008234 | cysteine-type peptidase activity | 3/37 | 0.006627 | 0.02819 | 0.013446 | CASP9/CASP3/CASP8 | 3 |
| MF | GO:0019902 | phosphatase binding | 3/37 | 0.006727 | 0.02819 | 0.013446 | BCL2/TP53/PPARG | 3 |
| MF | GO:0008227 | G protein-coupled amine receptor activity | 2/37 | 0.006752 | 0.02819 | 0.013446 | CHRM2/ADRB2 | 2 |
| MF | GO:0017171 | serine hydrolase activity | 3/37 | 0.006827 | 0.02819 | 0.013446 | F7/DPP4/PRSS1 | 3 |
| MF | GO:0031406 | carboxylic acid binding | 3/37 | 0.007556 | 0.030667 | 0.014627 | NOS2/PPARD/PPARG | 3 |
| MF | GO:0004860 | protein kinase inhibitor activity | 2/37 | 0.007667 | 0.030667 | 0.014627 | CASP3/CDKN1A | 2 |
| MF | GO:0033293 | monocarboxylic acid binding | 2/37 | 0.007904 | 0.031128 | 0.014847 | PPARD/PPARG | 2 |
| MF | GO:0019210 | kinase inhibitor activity | 2/37 | 0.008634 | 0.03349 | 0.015974 | CASP3/CDKN1A | 2 |
| MF | GO:0043177 | organic acid binding | 3/37 | 0.008909 | 0.03394 | 0.016188 | NOS2/PPARD/PPARG | 3 |
| MF | GO:0030971 | receptor tyrosine kinase binding | 2/37 | 0.009137 | 0.03394 | 0.016188 | TP53/PCNA | 2 |
| MF | GO:0019207 | kinase regulator activity | 3/37 | 0.009148 | 0.03394 | 0.016188 | CASP3/CDKN1A/CCNB1 | 3 |
| MF | GO:0001618 | virus receptor activity | 2/37 | 0.010451 | 0.037684 | 0.017974 | DPP4/CDK1 | 2 |
| MF | GO:0104005 | hijacked molecular function | 2/37 | 0.010451 | 0.037684 | 0.017974 | DPP4/CDK1 | 2 |
| MF | GO:0070405 | ammonium ion binding | 2/37 | 0.010724 | 0.038129 | 0.018186 | DRD1/SLC6A4 | 2 |
| MF | GO:0030295 | protein kinase activator activity | 2/37 | 0.012132 | 0.042545 | 0.020293 | CDKN1A/CCNB1 | 2 |
| MF | GO:0008013 | beta-catenin binding | 2/37 | 0.012717 | 0.043993 | 0.020984 | AR/ESR1 | 2 |
| MF | GO:0016209 | antioxidant activity | 2/37 | 0.013923 | 0.046331 | 0.022099 | PTGS2/PTGS1 | 2 |
| MF | GO:0019209 | kinase activator activity | 2/37 | 0.013923 | 0.046331 | 0.022099 | CDKN1A/CCNB1 | 2 |
| MF | GO:0001227 | DNA-binding transcription repressor activity, RNA polymerase II-specific | 3/37 | 0.013935 | 0.046331 | 0.022099 | PPARD/MYC/PPARG | 3 |
| MF | GO:0072341 | modified amino acid binding | 2/37 | 0.014859 | 0.048767 | 0.023261 | FASN/DPEP1 | 2 |
| MF | GO:0051213 | dioxygenase activity | 2/37 | 0.015498 | 0.05022 | 0.023953 | PTGS2/PTGS1 | 2 |
| MF | GO:1990782 | protein tyrosine kinase binding | 2/37 | 0.016148 | 0.051673 | 0.024647 | TP53/PCNA | 2 |
| MF | GO:0000980 | RNA polymerase II distal enhancer sequence-specific DNA binding | 2/37 | 0.018168 | 0.05742 | 0.027388 | JUN/TP53 | 2 |
| MF | GO:0051087 | chaperone binding | 2/37 | 0.019216 | 0.059992 | 0.028615 | BAX/TP53 | 2 |
| MF | GO:0050839 | cell adhesion molecule binding | 4/37 | 0.019778 | 0.060268 | 0.028746 | KDR/PRKCA/FASN/IL1B | 4 |
| MF | GO:0004952 | dopamine neurotransmitter receptor activity | 1/37 | 0.020717 | 0.060268 | 0.028746 | DRD1 | 1 |
| MF | GO:0004955 | prostaglandin receptor activity | 1/37 | 0.020717 | 0.060268 | 0.028746 | PPARG | 1 |
| MF | GO:0015378 | sodium:chloride symporter activity | 1/37 | 0.020717 | 0.060268 | 0.028746 | SLC6A4 | 1 |
| MF | GO:0043176 | amine binding | 1/37 | 0.020717 | 0.060268 | 0.028746 | SLC6A4 | 1 |
| MF | GO:0051378 | serotonin binding | 1/37 | 0.020717 | 0.060268 | 0.028746 | SLC6A4 | 1 |
| MF | GO:0005126 | cytokine receptor binding | 3/37 | 0.02165 | 0.061348 | 0.029261 | CASP3/CASP8/IL1B | 3 |
| MF | GO:0001094 | TFIID-class transcription factor complex binding | 1/37 | 0.022766 | 0.061348 | 0.029261 | TP53 | 1 |
| MF | GO:0004312 | fatty acid synthase activity | 1/37 | 0.022766 | 0.061348 | 0.029261 | FASN | 1 |
| MF | GO:0004954 | prostanoid receptor activity | 1/37 | 0.022766 | 0.061348 | 0.029261 | PPARG | 1 |
| MF | GO:0032404 | mismatch repair complex binding | 1/37 | 0.022766 | 0.061348 | 0.029261 | PCNA | 1 |
| MF | GO:0043560 | insulin receptor substrate binding | 1/37 | 0.022766 | 0.061348 | 0.029261 | PRKCD | 1 |
| MF | GO:0052650 | NADP-retinol dehydrogenase activity | 1/37 | 0.022766 | 0.061348 | 0.029261 | AKR1B1 | 1 |
| MF | GO:0008238 | exopeptidase activity | 2/37 | 0.023272 | 0.062057 | 0.0296 | DPP4/DPEP1 | 2 |
| MF | GO:0016004 | phospholipase activator activity | 1/37 | 0.02481 | 0.064853 | 0.030933 | CASP3 | 1 |
| MF | GO:0030594 | neurotransmitter receptor activity | 2/37 | 0.024826 | 0.064853 | 0.030933 | DRD1/CHRM2 | 2 |
| MF | GO:0001158 | enhancer sequence-specific DNA binding | 2/37 | 0.025619 | 0.065464 | 0.031225 | JUN/TP53 | 2 |
| MF | GO:0016616 | oxidoreductase activity, acting on the CH-OH group of donors, NAD or NADP as acceptor | 2/37 | 0.025619 | 0.065464 | 0.031225 | AKR1B1/FASN | 2 |
| MF | GO:0004032 | alditol:NADP+ 1-oxidoreductase activity | 1/37 | 0.02685 | 0.065464 | 0.031225 | AKR1B1 | 1 |
| MF | GO:0008179 | adenylate cyclase binding | 1/37 | 0.02685 | 0.065464 | 0.031225 | ADRB2 | 1 |
| MF | GO:0015373 | anion:sodium symporter activity | 1/37 | 0.02685 | 0.065464 | 0.031225 | SLC6A4 | 1 |
| MF | GO:0016653 | oxidoreductase activity, acting on NAD(P)H, heme protein as acceptor | 1/37 | 0.02685 | 0.065464 | 0.031225 | NOS2 | 1 |
| MF | GO:0035240 | dopamine binding | 1/37 | 0.02685 | 0.065464 | 0.031225 | DRD1 | 1 |
| MF | GO:0001162 | RNA polymerase II intronic transcription regulatory region sequence-specific DNA binding | 1/37 | 0.028886 | 0.066621 | 0.031777 | NCOA2 | 1 |
| MF | GO:0019104 | DNA N-glycosylase activity | 1/37 | 0.028886 | 0.066621 | 0.031777 | PCNA | 1 |
| MF | GO:0030983 | mismatched DNA binding | 1/37 | 0.028886 | 0.066621 | 0.031777 | PCNA | 1 |
| MF | GO:0035497 | cAMP response element binding | 1/37 | 0.028886 | 0.066621 | 0.031777 | JUN | 1 |
| MF | GO:0050998 | nitric-oxide synthase binding | 1/37 | 0.028886 | 0.066621 | 0.031777 | SLC6A4 | 1 |
| MF | GO:0060229 | lipase activator activity | 1/37 | 0.028886 | 0.066621 | 0.031777 | CASP3 | 1 |
| MF | GO:0016614 | oxidoreductase activity, acting on CH-OH group of donors | 2/37 | 0.029314 | 0.067004 | 0.031959 | AKR1B1/FASN | 2 |
| MF | GO:0004953 | icosanoid receptor activity | 1/37 | 0.030918 | 0.069431 | 0.033117 | PPARG | 1 |
| MF | GO:0016805 | dipeptidase activity | 1/37 | 0.030918 | 0.069431 | 0.033117 | DPEP1 | 1 |
| MF | GO:0035326 | enhancer binding | 2/37 | 0.031454 | 0.069705 | 0.033247 | JUN/TP53 | 2 |
| MF | GO:0004713 | protein tyrosine kinase activity | 2/37 | 0.031889 | 0.069705 | 0.033247 | KDR/PRKCD | 2 |
| MF | GO:0005149 | interleukin-1 receptor binding | 1/37 | 0.032946 | 0.069705 | 0.033247 | IL1B | 1 |
| MF | GO:0010181 | FMN binding | 1/37 | 0.032946 | 0.069705 | 0.033247 | NOS2 | 1 |
| MF | GO:0015377 | cation:chloride symporter activity | 1/37 | 0.032946 | 0.069705 | 0.033247 | SLC6A4 | 1 |
| MF | GO:0070402 | NADPH binding | 1/37 | 0.032946 | 0.069705 | 0.033247 | FASN | 1 |
| MF | GO:0071837 | HMG box domain binding | 1/37 | 0.032946 | 0.069705 | 0.033247 | JUN | 1 |
| MF | GO:0001161 | intronic transcription regulatory region sequence-specific DNA binding | 1/37 | 0.03497 | 0.071619 | 0.03416 | NCOA2 | 1 |
| MF | GO:0004935 | adrenergic receptor activity | 1/37 | 0.03497 | 0.071619 | 0.03416 | ADRB2 | 1 |
| MF | GO:0044213 | intronic transcription regulatory region DNA binding | 1/37 | 0.03497 | 0.071619 | 0.03416 | NCOA2 | 1 |
| MF | GO:0046965 | retinoid X receptor binding | 1/37 | 0.03497 | 0.071619 | 0.03416 | PPARG | 1 |
| MF | GO:0016247 | channel regulator activity | 2/37 | 0.03637 | 0.073895 | 0.035246 | ADRB2/BCL2 | 2 |
| MF | GO:0008353 | RNA polymerase II CTD heptapeptide repeat kinase activity | 1/37 | 0.03699 | 0.074562 | 0.035564 | CDK1 | 1 |
| MF | GO:0033218 | amide binding | 3/37 | 0.037896 | 0.075792 | 0.036151 | ADRB2/FASN/PPARG | 3 |
| MF | GO:0015296 | anion:cation symporter activity | 1/37 | 0.039005 | 0.07681 | 0.036636 | SLC6A4 | 1 |
| MF | GO:0070182 | DNA polymerase binding | 1/37 | 0.039005 | 0.07681 | 0.036636 | PCNA | 1 |
| MF | GO:0016922 | nuclear receptor binding | 1/37 | 0.041016 | 0.080154 | 0.038231 | NCOA2 | 1 |
| MF | GO:0008106 | alcohol dehydrogenase (NADP+) activity | 1/37 | 0.043024 | 0.081806 | 0.039019 | AKR1B1 | 1 |
| MF | GO:0016799 | hydrolase activity, hydrolyzing N-glycosyl compounds | 1/37 | 0.043024 | 0.081806 | 0.039019 | PCNA | 1 |
| MF | GO:0051861 | glycolipid binding | 1/37 | 0.043024 | 0.081806 | 0.039019 | DPEP1 | 1 |
| MF | GO:0004857 | enzyme inhibitor activity | 3/37 | 0.04314 | 0.081806 | 0.039019 | CASP3/CDKN1A/DPEP1 | 3 |
| MF | GO:0017025 | TBP-class protein binding | 1/37 | 0.045027 | 0.084756 | 0.040427 | ESR1 | 1 |
| MF | GO:0001223 | transcription coactivator binding | 1/37 | 0.047026 | 0.086609 | 0.04131 | ESR1 | 1 |
| MF | GO:0015464 | acetylcholine receptor activity | 1/37 | 0.047026 | 0.086609 | 0.04131 | CHRM2 | 1 |
| MF | GO:0070412 | R-SMAD binding | 1/37 | 0.047026 | 0.086609 | 0.04131 | JUN | 1 |
| MF | GO:0001965 | G-protein alpha-subunit binding | 1/37 | 0.049021 | 0.089638 | 0.042755 | DRD1 | 1 |
