# Supplementary material for: Network pharmacology and molecular docking technology-based predictive study of the active ingredients and potential targets of rhubarb for the treatment of diabetic nephropathy
Source: BMC Complement Med Ther. 2022 Aug 6;22:210. doi: 10.1186/s12906-022-03662-6 (PMC9356435; doi:10.1186/s12906-022-03662-6)
Supplement: Supplementary file 6 — Additional file 6. [file 12906_2022_3662_MOESM6_ESM.docx]

| **ID** | **Description** | **GeneRatio** | **BgRatio** | **pvalue** | **p.adjust** | **qvalue** | **geneID** | **Count** |
| --- | --- | --- | --- | --- | --- | --- | --- | --- |
| hsa04115 | p53 signaling pathway | 9/35 | 73/8062 | 1.44E-11 | 2.80E-09 | 1.33E-09 | BCL2/BAX/CASP9/CASP3/CASP8/CDKN1A/TP53/CDK1/CCNB1 | 9 |
| hsa05161 | Hepatitis B | 11/35 | 162/8062 | 4.22E-11 | 4.10E-09 | 1.96E-09 | JUN/BCL2/BAX/CASP9/CASP3/CASP8/PRKCA/CDKN1A/TP53/PCNA/MYC | 11 |
| hsa05222 | Small cell lung cancer | 9/35 | 92/8062 | 1.22E-10 | 7.89E-09 | 3.77E-09 | NOS2/PTGS2/BCL2/BAX/CASP9/CASP3/CDKN1A/TP53/MYC | 9 |
| hsa05210 | Colorectal cancer | 8/35 | 86/8062 | 2.24E-09 | 1.09E-07 | 5.19E-08 | JUN/BCL2/BAX/CASP9/CASP3/CDKN1A/TP53/MYC | 8 |
| hsa01524 | Platinum drug resistance | 7/35 | 73/8062 | 2.04E-08 | 7.56E-07 | 3.61E-07 | BCL2/BAX/CASP9/CASP3/CASP8/CDKN1A/TP53 | 7 |
| hsa05163 | Human cytomegalovirus infection | 10/35 | 225/8062 | 2.34E-08 | 7.56E-07 | 3.61E-07 | PTGS2/BAX/CASP9/CASP3/CASP8/PRKCA/CDKN1A/TP53/MYC/IL1B | 10 |
| hsa05167 | Kaposi sarcoma-associated herpesvirus infection | 9/35 | 193/8062 | 8.84E-08 | 2.45E-06 | 1.17E-06 | PTGS2/JUN/BAX/CASP9/CASP3/CASP8/CDKN1A/TP53/MYC | 9 |
| hsa05162 | Measles | 8/35 | 139/8062 | 1.01E-07 | 2.46E-06 | 1.17E-06 | JUN/BCL2/BAX/CASP9/CASP3/CASP8/TP53/IL1B | 8 |
| hsa05169 | Epstein-Barr virus infection | 9/35 | 202/8062 | 1.31E-07 | 2.82E-06 | 1.35E-06 | JUN/BCL2/BAX/CASP9/CASP3/CASP8/CDKN1A/TP53/MYC | 9 |
| hsa05224 | Breast cancer | 8/35 | 147/8062 | 1.57E-07 | 2.84E-06 | 1.35E-06 | ESR2/JUN/ESR1/PGR/BAX/CDKN1A/TP53/MYC | 8 |
| hsa01522 | Endocrine resistance | 7/35 | 98/8062 | 1.61E-07 | 2.84E-06 | 1.35E-06 | ESR2/JUN/ESR1/BCL2/BAX/CDKN1A/TP53 | 7 |
| hsa04933 | AGE-RAGE signaling pathway in diabetic complications | 7/35 | 100/8062 | 1.85E-07 | 2.94E-06 | 1.40E-06 | JUN/BCL2/BAX/CASP3/PRKCA/IL1B/PRKCD | 7 |
| hsa05170 | Human immunodeficiency virus 1 infection | 9/35 | 212/8062 | 1.98E-07 | 2.94E-06 | 1.40E-06 | JUN/BCL2/BAX/CASP9/CASP3/CASP8/PRKCA/CDK1/CCNB1 | 9 |
| hsa04215 | Apoptosis - multiple species | 5/35 | 32/8062 | 2.12E-07 | 2.94E-06 | 1.40E-06 | BCL2/BAX/CASP9/CASP3/CASP8 | 5 |
| hsa05216 | Thyroid cancer | 5/35 | 37/8062 | 4.52E-07 | 5.85E-06 | 2.79E-06 | BAX/CDKN1A/TP53/MYC/PPARG | 5 |
| hsa04210 | Apoptosis | 7/35 | 136/8062 | 1.51E-06 | 1.83E-05 | 8.74E-06 | JUN/BCL2/BAX/CASP9/CASP3/CASP8/TP53 | 7 |
| hsa04915 | Estrogen signaling pathway | 7/35 | 138/8062 | 1.67E-06 | 1.90E-05 | 9.08E-06 | ESR2/NCOA2/JUN/ESR1/PGR/BCL2/PRKCD | 7 |
| hsa05160 | Hepatitis C | 7/35 | 157/8062 | 3.95E-06 | 4.26E-05 | 2.03E-05 | BAX/CASP9/CASP3/CASP8/CDKN1A/TP53/MYC | 7 |
| hsa05213 | Endometrial cancer | 5/35 | 58/8062 | 4.45E-06 | 4.54E-05 | 2.17E-05 | BAX/CASP9/CDKN1A/TP53/MYC | 5 |
| hsa05164 | Influenza A | 7/35 | 171/8062 | 6.96E-06 | 6.75E-05 | 3.22E-05 | PRSS1/BAX/CASP9/CASP3/CASP8/PRKCA/IL1B | 7 |
| hsa05152 | Tuberculosis | 7/35 | 180/8062 | 9.76E-06 | 8.65E-05 | 4.13E-05 | NOS2/BCL2/BAX/CASP9/CASP3/CASP8/IL1B | 7 |
| hsa05223 | Non-small cell lung cancer | 5/35 | 68/8062 | 9.81E-06 | 8.65E-05 | 4.13E-05 | BAX/CASP9/PRKCA/CDKN1A/TP53 | 5 |
| hsa04919 | Thyroid hormone signaling pathway | 6/35 | 121/8062 | 1.15E-05 | 9.68E-05 | 4.62E-05 | NCOA2/ESR1/CASP9/PRKCA/TP53/MYC | 6 |
| hsa04110 | Cell cycle | 6/35 | 124/8062 | 1.32E-05 | 0.000107 | 5.10E-05 | CDKN1A/TP53/CDK1/PCNA/MYC/CCNB1 | 6 |
| hsa05203 | Viral carcinogenesis | 7/35 | 204/8062 | 2.21E-05 | 0.00017 | 8.12E-05 | JUN/BAX/CASP3/CASP8/CDKN1A/TP53/CDK1 | 7 |
| hsa05205 | Proteoglycans in cancer | 7/35 | 205/8062 | 2.28E-05 | 0.00017 | 8.12E-05 | KDR/ESR1/CASP3/PRKCA/CDKN1A/TP53/MYC | 7 |
| hsa05132 | Salmonella infection | 7/35 | 213/8062 | 2.92E-05 | 0.00021 | 0.0001 | JUN/BCL2/BAX/CASP3/CASP8/MYC/IL1B | 7 |
| hsa04657 | IL-17 signaling pathway | 5/35 | 94/8062 | 4.77E-05 | 0.00033 | 0.000158 | PTGS2/JUN/CASP3/CASP8/IL1B | 5 |
| hsa05215 | Prostate cancer | 5/35 | 97/8062 | 5.54E-05 | 0.000371 | 0.000177 | AR/BCL2/CASP9/CDKN1A/TP53 | 5 |
| hsa04625 | C-type lectin receptor signaling pathway | 5/35 | 104/8062 | 7.74E-05 | 0.000501 | 0.000239 | PTGS2/JUN/CASP8/IL1B/PRKCD | 5 |
| hsa05134 | Legionellosis | 4/35 | 57/8062 | 9.99E-05 | 0.000625 | 0.000298 | CASP9/CASP3/CASP8/IL1B | 4 |
| hsa04151 | PI3K-Akt signaling pathway | 8/35 | 354/8062 | 0.000105946 | 0.000628 | 0.0003 | KDR/CHRM2/BCL2/CASP9/PRKCA/CDKN1A/TP53/MYC | 8 |
| hsa04668 | TNF signaling pathway | 5/35 | 112/8062 | 0.000110148 | 0.000628 | 0.0003 | PTGS2/JUN/CASP3/CASP8/IL1B | 5 |
| hsa05145 | Toxoplasmosis | 5/35 | 112/8062 | 0.000110148 | 0.000628 | 0.0003 | NOS2/BCL2/CASP9/CASP3/CASP8 | 5 |
| hsa04370 | VEGF signaling pathway | 4/35 | 59/8062 | 0.000114373 | 0.000634 | 0.000303 | PTGS2/KDR/CASP9/PRKCA | 4 |
| hsa04726 | Serotonergic synapse | 5/35 | 115/8062 | 0.000124842 | 0.000673 | 0.000321 | PTGS2/PTGS1/SLC6A4/CASP3/PRKCA | 5 |
| hsa04722 | Neurotrophin signaling pathway | 5/35 | 119/8062 | 0.000146724 | 0.000769 | 0.000367 | JUN/BCL2/BAX/TP53/PRKCD | 5 |
| hsa05130 | Pathogenic Escherichia coli infection | 6/35 | 193/8062 | 0.000158435 | 0.000809 | 0.000386 | JUN/BAX/CASP9/CASP3/CASP8/IL1B | 6 |
| hsa04010 | MAPK signaling pathway | 7/35 | 294/8062 | 0.000222839 | 0.001108 | 0.000529 | KDR/JUN/CASP3/PRKCA/TP53/MYC/IL1B | 7 |
| hsa05214 | Glioma | 4/35 | 75/8062 | 0.000290769 | 0.00133 | 0.000635 | BAX/PRKCA/CDKN1A/TP53 | 4 |
| hsa05418 | Fluid shear stress and atherosclerosis | 5/35 | 139/8062 | 0.000303518 | 0.00133 | 0.000635 | KDR/JUN/BCL2/TP53/IL1B | 5 |
| hsa05133 | Pertussis | 4/35 | 76/8062 | 0.00030598 | 0.00133 | 0.000635 | NOS2/JUN/CASP3/IL1B | 4 |
| hsa05212 | Pancreatic cancer | 4/35 | 76/8062 | 0.00030598 | 0.00133 | 0.000635 | BAX/CASP9/CDKN1A/TP53 | 4 |
| hsa05220 | Chronic myeloid leukemia | 4/35 | 76/8062 | 0.00030598 | 0.00133 | 0.000635 | BAX/CDKN1A/TP53/MYC | 4 |
| hsa05206 | MicroRNAs in cancer | 7/35 | 310/8062 | 0.000308456 | 0.00133 | 0.000635 | PTGS2/BCL2/CASP3/PRKCA/CDKN1A/TP53/MYC | 7 |
| hsa05140 | Leishmaniasis | 4/35 | 77/8062 | 0.000321755 | 0.001357 | 0.000648 | NOS2/PTGS2/JUN/IL1B | 4 |
| hsa01521 | EGFR tyrosine kinase inhibitor resistance | 4/35 | 79/8062 | 0.000355044 | 0.001466 | 0.0007 | KDR/BCL2/BAX/PRKCA | 4 |
| hsa05226 | Gastric cancer | 5/35 | 149/8062 | 0.000418471 | 0.001691 | 0.000808 | BCL2/BAX/CDKN1A/TP53/MYC | 5 |
| hsa04932 | Non-alcoholic fatty liver disease | 5/35 | 150/8062 | 0.000431555 | 0.001709 | 0.000816 | JUN/BAX/CASP3/CASP8/IL1B | 5 |
| hsa04012 | ErbB signaling pathway | 4/35 | 85/8062 | 0.000469714 | 0.001822 | 0.00087 | JUN/PRKCA/CDKN1A/MYC | 4 |
| hsa04218 | Cellular senescence | 5/35 | 156/8062 | 0.000516687 | 0.001965 | 0.000938 | CDKN1A/TP53/CDK1/MYC/CCNB1 | 5 |
| hsa05131 | Shigellosis | 6/35 | 242/8062 | 0.000537104 | 0.002004 | 0.000957 | JUN/BCL2/BAX/TP53/IL1B/PRKCD | 6 |
| hsa04310 | Wnt signaling pathway | 5/35 | 160/8062 | 0.000580105 | 0.002123 | 0.001014 | PPARD/JUN/PRKCA/TP53/MYC | 5 |
| hsa05219 | Bladder cancer | 3/35 | 41/8062 | 0.000713714 | 0.002555 | 0.00122 | CDKN1A/TP53/MYC | 3 |
| hsa05225 | Hepatocellular carcinoma | 5/35 | 168/8062 | 0.00072436 | 0.002555 | 0.00122 | BAX/PRKCA/CDKN1A/TP53/MYC | 5 |
| hsa05142 | Chagas disease | 4/35 | 102/8062 | 0.000935634 | 0.003184 | 0.001521 | NOS2/JUN/CASP8/IL1B | 4 |
| hsa05146 | Amoebiasis | 4/35 | 102/8062 | 0.000935634 | 0.003184 | 0.001521 | NOS2/CASP3/PRKCA/IL1B | 4 |
| hsa04621 | NOD-like receptor signaling pathway | 5/35 | 181/8062 | 0.00101418 | 0.003392 | 0.00162 | JUN/BCL2/CASP8/IL1B/PRKCD | 5 |
| hsa04066 | HIF-1 signaling pathway | 4/35 | 109/8062 | 0.001198806 | 0.003942 | 0.001882 | NOS2/BCL2/PRKCA/CDKN1A | 4 |
| hsa05202 | Transcriptional misregulation in cancer | 5/35 | 192/8062 | 0.001320547 | 0.00427 | 0.002039 | BAX/CDKN1A/TP53/MYC/PPARG | 5 |
| hsa04020 | Calcium signaling pathway | 5/35 | 201/8062 | 0.00161841 | 0.005147 | 0.002458 | NOS2/DRD1/CHRM2/ADRB2/PRKCA | 5 |
| hsa04071 | Sphingolipid signaling pathway | 4/35 | 119/8062 | 0.001659369 | 0.005192 | 0.002479 | BCL2/BAX/PRKCA/TP53 | 4 |
| hsa04923 | Regulation of lipolysis in adipocytes | 3/35 | 56/8062 | 0.0017751 | 0.005466 | 0.00261 | PTGS2/PTGS1/ADRB2 | 3 |
| hsa05016 | Huntington disease | 6/35 | 306/8062 | 0.001821671 | 0.005522 | 0.002637 | BAX/CASP9/CASP3/CASP8/TP53/PPARG | 6 |
| hsa05416 | Viral myocarditis | 3/35 | 60/8062 | 0.002165501 | 0.006374 | 0.003044 | CASP9/CASP3/CASP8 | 3 |
| hsa04114 | Oocyte meiosis | 4/35 | 128/8062 | 0.002168524 | 0.006374 | 0.003044 | AR/PGR/CDK1/CCNB1 | 4 |
| hsa05166 | Human T-cell leukemia virus 1 infection | 5/35 | 219/8062 | 0.002359366 | 0.006832 | 0.003262 | JUN/BAX/CDKN1A/TP53/MYC | 5 |
| hsa05217 | Basal cell carcinoma | 3/35 | 63/8062 | 0.002490734 | 0.007106 | 0.003393 | BAX/CDKN1A/TP53 | 3 |
| hsa05165 | Human papillomavirus infection | 6/35 | 331/8062 | 0.002709047 | 0.007617 | 0.003637 | PTGS2/BAX/CASP3/CASP8/CDKN1A/TP53 | 6 |
| hsa05031 | Amphetamine addiction | 3/35 | 69/8062 | 0.003228333 | 0.008947 | 0.004272 | JUN/DRD1/PRKCA | 3 |
| hsa05218 | Melanoma | 3/35 | 72/8062 | 0.003642306 | 0.009952 | 0.004752 | BAX/CDKN1A/TP53 | 3 |
| hsa05012 | Parkinson disease | 5/35 | 249/8062 | 0.004106161 | 0.011064 | 0.005283 | DRD1/BAX/CASP9/CASP3/TP53 | 5 |
| hsa04921 | Oxytocin signaling pathway | 4/35 | 154/8062 | 0.004229235 | 0.011239 | 0.005367 | PTGS2/JUN/PRKCA/CDKN1A | 4 |
| hsa05014 | Amyotrophic lateral sclerosis | 6/35 | 364/8062 | 0.004339669 | 0.011377 | 0.005432 | NOS2/BCL2/BAX/CASP9/CASP3/TP53 | 6 |
| hsa05168 | Herpes simplex virus 1 infection | 7/35 | 490/8062 | 0.004453839 | 0.011521 | 0.005501 | BCL2/BAX/CASP9/CASP3/CASP8/TP53/IL1B | 7 |
| hsa05010 | Alzheimer disease | 6/35 | 369/8062 | 0.004639384 | 0.011843 | 0.005655 | NOS2/PTGS2/CASP9/CASP3/CASP8/IL1B | 6 |
| hsa04217 | Necroptosis | 4/35 | 159/8062 | 0.004738606 | 0.011939 | 0.005701 | BCL2/BAX/CASP8/IL1B | 4 |
| hsa05020 | Prion disease | 5/35 | 273/8062 | 0.006058481 | 0.015069 | 0.007195 | BAX/CASP9/CASP3/IL1B/PRKCD | 5 |
| hsa04540 | Gap junction | 3/35 | 88/8062 | 0.006391976 | 0.015697 | 0.007495 | DRD1/PRKCA/CDK1 | 3 |
| hsa04211 | Longevity regulating pathway | 3/35 | 89/8062 | 0.006595435 | 0.015994 | 0.007637 | BAX/TP53/PPARG | 3 |
| hsa04912 | GnRH signaling pathway | 3/35 | 93/8062 | 0.007447872 | 0.017838 | 0.008517 | JUN/PRKCA/PRKCD | 3 |
| hsa04914 | Progesterone-mediated oocyte maturation | 3/35 | 99/8062 | 0.008844238 | 0.020924 | 0.009991 | PGR/CDK1/CCNB1 | 3 |
| hsa04750 | Inflammatory mediator regulation of TRP channels | 3/35 | 100/8062 | 0.009090905 | 0.021249 | 0.010146 | PRKCA/IL1B/PRKCD | 3 |
| hsa04064 | NF-kappa B signaling pathway | 3/35 | 104/8062 | 0.010117936 | 0.023093 | 0.011026 | PTGS2/BCL2/IL1B | 3 |
| hsa04620 | Toll-like receptor signaling pathway | 3/35 | 104/8062 | 0.010117936 | 0.023093 | 0.011026 | JUN/CASP8/IL1B | 3 |
| hsa04928 | Parathyroid hormone synthesis, secretion and action | 3/35 | 106/8062 | 0.010655855 | 0.023919 | 0.011421 | BCL2/PRKCA/CDKN1A | 3 |
| hsa04510 | Focal adhesion | 4/35 | 201/8062 | 0.010726618 | 0.023919 | 0.011421 | KDR/JUN/BCL2/PRKCA | 4 |
| hsa05143 | African trypanosomiasis | 2/35 | 37/8062 | 0.011087198 | 0.024442 | 0.011671 | PRKCA/IL1B | 2 |
| hsa04725 | Cholinergic synapse | 3/35 | 113/8062 | 0.012668332 | 0.027614 | 0.013185 | CHRM2/BCL2/PRKCA | 3 |
| hsa04024 | cAMP signaling pathway | 4/35 | 216/8062 | 0.013692614 | 0.029515 | 0.014093 | JUN/DRD1/CHRM2/ADRB2 | 4 |
| hsa04380 | Osteoclast differentiation | 3/35 | 128/8062 | 0.017673039 | 0.037677 | 0.01799 | JUN/IL1B/PPARG | 3 |
| hsa04926 | Relaxin signaling pathway | 3/35 | 129/8062 | 0.018040706 | 0.038042 | 0.018165 | NOS2/JUN/PRKCA | 3 |
| hsa05030 | Cocaine addiction | 2/35 | 49/8062 | 0.018952132 | 0.039535 | 0.018877 | JUN/DRD1 | 2 |
| hsa04961 | Endocrine and other factor-regulated calcium reabsorption | 2/35 | 53/8062 | 0.021969037 | 0.04534 | 0.021649 | ESR1/PRKCA | 2 |
| hsa04261 | Adrenergic signaling in cardiomyocytes | 3/35 | 149/8062 | 0.026297394 | 0.053702 | 0.025642 | ADRB2/BCL2/PRKCA | 3 |
| hsa00590 | Arachidonic acid metabolism | 2/35 | 63/8062 | 0.030308456 | 0.061248 | 0.029245 | PTGS2/PTGS1 | 2 |
| hsa04929 | GnRH secretion | 2/35 | 64/8062 | 0.0312021 | 0.062404 | 0.029797 | ESR2/PRKCA | 2 |
| hsa05321 | Inflammatory bowel disease | 2/35 | 65/8062 | 0.032106183 | 0.063557 | 0.030347 | JUN/IL1B | 2 |
| hsa04630 | JAK-STAT signaling pathway | 3/35 | 162/8062 | 0.032588778 | 0.063861 | 0.030492 | BCL2/CDKN1A/MYC | 3 |
| hsa05221 | Acute myeloid leukemia | 2/35 | 67/8062 | 0.033945259 | 0.065854 | 0.031444 | PPARD/MYC | 2 |
| hsa04137 | Mitophagy - animal | 2/35 | 68/8062 | 0.034880051 | 0.066997 | 0.03199 | JUN/TP53 | 2 |
| hsa05211 | Renal cell carcinoma | 2/35 | 69/8062 | 0.035824879 | 0.067955 | 0.032447 | JUN/CDKN1A | 2 |
| hsa04917 | Prolactin signaling pathway | 2/35 | 70/8062 | 0.036779642 | 0.067955 | 0.032447 | ESR2/ESR1 | 2 |
| hsa05120 | Epithelial cell signaling in Helicobacter pylori infection | 2/35 | 70/8062 | 0.036779642 | 0.067955 | 0.032447 | JUN/CASP3 | 2 |
| hsa05230 | Central carbon metabolism in cancer | 2/35 | 70/8062 | 0.036779642 | 0.067955 | 0.032447 | TP53/MYC | 2 |
| hsa03320 | PPAR signaling pathway | 2/35 | 77/8062 | 0.043732997 | 0.08004 | 0.038218 | PPARD/PPARG | 2 |
